# Associations between per-and polyfluoroalkyl substances (PFAS) and county-level cancer incidence between 2016 and 2021 and incident cancer burden attributable to PFAS in drinking water in the United States

**DOI:** 10.1038/s41370-024-00742-2

**Published:** 2025-01-09

**Authors:** Shiwen Li, Paulina Oliva, Lu Zhang, Jesse A. Goodrich, Rob McConnell, David V. Conti, Lida Chatzi, Max Aung

**Affiliations:** 1 Department of Population and Public Health Sciences, Keck School of Medicine, University of Southern California, Los Angeles, CA USA; 2 Department of Economics, Dornsife College of Letters, Arts and Sciences, University of Southern California, Los Angeles, CA USA

**Keywords:** PFAS, Cancer, Drinking Water

## Abstract

**Background:**

Exposure to per- and polyfluoroalkyl substances (PFAS) has been linked with various cancers. Assessment of PFAS in drinking water and cancers can help inform biomonitoring and prevention efforts.

**Objective:**

To screen for incident cancer (2016–2021) and assess associations with PFAS contamination in drinking water in the US.

**Methods:**

We obtained county-level age-adjusted cancer incidence (2016–2021) from the Surveillance, Epidemiology, and End Results (SEER) Program. Data on PFAS levels in public drinking water systems were obtained from the Third (UCMR3; 2013–2015) and Fifth (UCMR5; 2023–2024) Unregulated Contaminant Monitoring Rule. UCMR3 measured PFOS, PFOA, PFNA, PFHxS, PFHpA, and PFBS. UCMR5 expanded measurements to include PFBA, PFHxA, PFPeA, and PFPeS. We created indicators of PFAS detection and, for UCMR5, concentrations above Maximum Contaminant Levels (MCLs). MCLs for PFOA and PFOS are 4 ng/L, and for PFNA and PFHxS are 10 ng/L. We used Poisson regression models to assess associations between PFAS detection or MCL violation and cancer incidence, adjusting for potential confounders. We estimated the number of attributable cancer cases.

**Results:**

PFAS in drinking water was associated with increased cancer incidence in the digestive, endocrine, oral cavity/pharynx, and respiratory systems. Incidence rate ratios (IRRs) ranged from 1.02 to 1.33. The strongest association was observed between PFBS and oral cavity/pharynx cancers (IRR: 1.33 [1.04, 1.71]). Among males, PFAS was associated with cancers in the urinary, brain, leukemia, and soft tissues. Among females, PFAS was associated with cancers in the thyroid, oral cavity/pharynx, and soft tissue. PFAS in drinking water is estimated to contribute to 4626 [95% CI: 1,377, 8046] incident cancer cases per year based on UCMR3 data and 6864 [95% CI: 991, 12,804] based on UCMR5.

**Impact statement:**

The ecological study examined the associations between PFAS in drinking water measured in two waves (2013–2015 and 2023–2024) and cancer incidence between 2016 and 2021. We found that PFAS in drinking water was associated with cancers in the organ system including the oral cavity/pharynx, lung, digestive system, brain, urinary system, soft tissue, and thyroid. Some cancers have not been widely studied for their associations with PFAS. We also observed sex differences in the associations between PFAS and cancer risks. This is the first ecological study that examined PFAS exposure in drinking water and various cancer risks.

## Introduction

Per- and polyfluoroalkyl substances (PFAS) are synthetic chemicals that have been widely used in consumer products and have accumulated in the environment since the 1940s due to their resistance to degradation [1]. Drinking water is a major source of exposure for the general population [2]. One recent study suggests that one or more types of PFAS were detected in at least 45% of drinking water across the US near urban areas and potential PFAS sources [3]. Smalling et al. found that 40% of the public water had PFAS detected compared to 20% of the private-well samples although the concentrations of individual PFAS were not different between public and private-well water samples. On April 10, 2024, the US Environmental Protection Agency (EPA) announced the final National Primary Drinking Water Regulation which sets the Maximum Contaminant levels (MCL), a legally enforceable level of contaminants in drinking water, for 6 PFAS [4]. The unprecedented rulings regulating PFAS in drinking water demonstrate the importance of assessing the health risks of PFAS from drinking water.

It is estimated that there were 1.9 million new cancer cases and more than 600,000 deaths from cancers in the US in 2023 [5]. Endocrine-disrupting chemicals like PFAS have been proposed as environmental risk factors for cancers through multiple mechanisms, such as *alterations in male and female reproduction, epigenetic changes, and changes in neuroendocrinology, behavior, and metabolism which can lead to the development of cancers* [6, 7].

PFAS have been linked to various health outcomes including cancers [8] and the International Agency for Research on Cancer (IARC) has listed PFOA as carcinogenic to humans (Group 1) and PFOS as possibly carcinogenic to humans (Group 2B) [9]. A systematic review and meta-analysis identified associations between PFAS and kidney and testicular cancers [10]. In addition, the National Academies of Sciences, Engineering, and Medicine (NASEM) conducted reviews of evidence for health risks of PFAS and concluded sufficient causal evidence existed for the associations between PFAS and kidney cancer, suggestive evidence for associations between PFAS and breast and testicular cancer [11].

Several mechanisms were identified including endocrine disruption, lipid metabolism, epigenetic change, oxidative stress, chronic inflammation, and immunosuppression [12]. For example, PFAS can bind to nuclear receptors such as peroxisome proliferator-activated receptors which regulate the lipid metabolism although evidence is less clear for the direct binding to estrogen and androgen receptors [6]. Disruption in hormone homeostasis is implicated in the mechanisms causing liver, thyroid, prostate, and breast cancers [12]. PFAS have also been linked to both hyper- and hypomethylation of DNA and functional analysis of the differentially methylated probes or regions showed the involvement of cancer and reproductive disease [12, 13]. Therefore, there is strong biological plausibility to link PFAS with cancers.

In 2022, approximately half of the new cancer cases were cancers of the prostate, breast, lung, colon, and rectum [14] but existing epidemiological studies were limited to the associations between PFAS and these cancers. In addition, fewer studies focused on the source-specific effect of PFAS, and most of these studies were based in European countries [15] except for studies from the C8 Health Project [16, 17], one study examining the correlation between PFAS in water and thyroid cancer in the US [18] and the associations between PFOA in water and cancer risks in the Mid-Ohio River Valley [16]. Identifying the contribution to cancer risk from PFAS in drinking water would help prioritize and target drinking water quality to reduce risk. Thus, there is an urgent need for more health assessment of cancer risks from PFAS, especially from drinking water.

We hypothesized that there are cancers caused by PFAS in drinking water that have not been identified previously due to the limited sample size of cancer cases and lack of PFAS quality data. We aimed to screen for associations between PFAS in drinking water and county-level cancer incidence in an ecological study that would help direct future epidemiological and experimental research on PFAS and cancer. We used data from the Third Unregulated Contaminant Monitoring Rule (UCMR3) [19], and the Fifth Unregulated Contaminant Monitoring Rule (UCMR5) [20] in public drinking water systems (PWS), and cancer incidence data from the Surveillance, Epidemiology, and End Results (SEER) Program between 2016 and 2021. To better understand the cancer burden due to PFAS and provide a policy-relevant perspective, we additionally estimated the number of cancer incidence cases attributable to PFAS in drinking water.

## Method

### Cancer incidence data

We obtained cancer incidence data from the U.S. National Cancer Institute’s (NCI) Surveillance, Epidemiology, and End Results (SEER) program [21]. The geographic coverage of the SEER database, which includes 22 cancer registries, is approximately half of the US population. The geographic area coverage included Connecticut, Hawaii, Iowa, New Mexico, Seattle, Utah, Atlanta, Alaska Natives, Georgia, California, Kentucky, Louisiana, New Jersey, Idaho, New York, and Texas. The data are publicly available and can be obtained through the SEER*Stat program [21].

County-level age-adjusted incidence rates between 2016 and 2021 (patients with diagnosis of cancers between 2016 and 2021) were calculated using the SEER*Stat program using “Incidence – SEER Research Limited-Field Data, 22 Registries, Nov 2023 (2000-2021)” database [21]. The crude rate was age-adjusted according to the 2000 US population. We obtained yearly, age-adjusted cancer incidence rates per 100,000 for each county and each cancer site. The cancer site was based on the ICD-O-3/WHO Classification of Tumours of Haematopoietic and Lymphoid Tissues (2008), grouped as follows: oral cavity and pharynx, digestive system, respiratory system, bones and joints, soft tissue including heart, skin excluding basal and squamous (excluding non-melanoma cases), breast, female genital system, male genital system, urinary system, brain and other nervous system, endocrine system, lymphoma, myeloma, leukemia. We excluded cancers in the eye and orbit, mesothelioma, and Kaposi sarcoma due to the limited number of cases. We assessed the associations between PFAS and cancers in the male/female genital system in stratified analyses (excluded from the main analysis since sex-specific incidence rate is a more appropriate outcome).

### PFAS in drinking water

We obtained two different sets of PFAS data: (1) US EPA’s Third Unregulated Contaminant Monitoring Rule (UCMR3) data (2013 to 2015) [19] and (2) US EPA’s Fifth Unregulated Contaminant Monitoring Rule (UCMR5) data (2023 to March 2024) [20].

UCMR3 was conducted by the US EPA between January 2013 and December 2015 under the Safe Drinking Water Act and monitored unregulated contaminants in PWS including 6 PFAS: perfluorooctanesulfonic acid (PFOS), perfluorooctanoic acid (PFOA), perfluorononanoic acid (PFNA), perfluorohexanesulfonic acid (PFHxS), perfluoroheptanoic acid (PFHpA), and perfluorobutanesulfonic acid (PFBS). UCMR3 tested PFAS in all PWSs serving more than 10,000 people and 800 representative PWSs serving 10,000 or fewer people across all states in the US. The minimum reporting levels (MRLs) for PFOS, PFOA, PFNA, PFHxS, PFHpA, and PFBS were 0.04, 0.02, 0.02, 0.03, 0.01, and 0.09 µg/L, respectively. The detection rates for PFOS, PFOA, PFNA, PFHxS, PFHpA, and PFBS across all samples were 0.79%, 1.03%, 0.05%, 0.56%, 0.64%, and 0.05%.

UCMR5 started in 2023 and the most recent release of data was up to March 2024 [20]. 29 PFAS were tested by UCMR5 and MRL for these PFAS ranges from 2 to 20 ng/L. We included PFAS that were commonly present in PWS including 6 PFAS mentioned above plus perfluorobutanoic acid (PFBA), perfluorohexanoic acid (PFHxA), perfluoropentanoic acid (PFPeA) and perfluoropentane sulfonic acid (PFPeS) because these additional PFAS were detected in the water systems. We hypothesize that UCMR5 was a valid measure of exposure because of the lower detection of limit for each PFAS, potentially correcting the exposure misclassification in UCMR3.

We downloaded the geographic boundaries of PWSs for the United States [22] and linked each of the PFAS data to the PWS boundaries. Briefly, SimpleLab used three approaches to identify boundaries of water systems: (1) use water service boundaries provided by each state, (2) matching algorithm to match water systems to the boundary of a town or city, (3) use a statistical model to estimate the reasonable radium of a water service boundary. Therefore, misclassification of water boundaries is likely for the second and third approaches. We calculated the average PFAS concentrations detected for each PWS and each PFAS chemical by different data sources.

Since no detection of PFAS could mean either a water system had no PFAS or had PFAS concentrations below the detection limit and we had to average across values across multiple samples collected for the same water system and for each county, using the continuously measured concentrations for each PFAS would not be meaningful. In addition, detection limits for UCMR3 are much higher and a wide range of values of concentrations was possible. Thus, imputation is not appropriate.

For UCMR3 data, we created a binary variable indicating the detection of PFAS in the drinking water for each county.

We created another binary variable indicating the violation of Maximum Contaminant Levels (MCLs), which are legally enforceable levels of contaminants in drinking water. MCLs for PFOA and PFOS are 4 ng/L and MCLs for PFNA and PFHxS are 10 ng/L. Since there were no individual MCLs for other PFAS, we only created detected/not detected variables for other PFAS. We only created this variable for UCMR5 not for UCMR3 because the MRL in UCMR3 was much higher than MCL.

We additionally created a binary variable indicating at least 1 PFAS was detected in drinking water separately for UCMR3 and UCMR5, and another binary variable indicating each of the PFAS was detected/above MCL in both UCMR3 and UCMR5.

### Covariates

We obtained 5-year estimates of American Community Survey data from 2013 to 2017. We calculated the following county-level socioeconomic status (SES) variables: percent of people of color (including any race with Hispanic ethnicity)/non-White, percent of the population (25 years and over) with education below high school, percent of the population whose income was below the federal poverty line, and percent of the population in the workforce that was unemployed.

We obtained additional potential confounders including air pollution, obesity prevalence, smoking rate, and urbanicity. We included air pollution as a proxy for co-occurring environmental pollutants with PFAS contamination in drinking water since areas with high industrial activities would have potentially high air pollution levels due to traffic [23–26]. The data source for each covariate is described below.

We downloaded 2018 annual average fine particulate matter (PM2.5; unit: μg/m^3^, resolution: 0.01° × 0.01°) data [27, 28] and calculated county-level average PM2.5. PM2.5 was estimated using Aerosol Optical Depth (AOD) retrievals from the NASA MODIS, MISR, and SeaWIFS instruments with the GEOS-Chem chemical transport model, and subsequently calibrated to regional ground-based observations of both total and compositional mass using Geographically Weighted Regression (GWR).

Urbanicity was classified based on the 2013 NCHS Urban-Rural Classification Scheme for Counties which classified counties into the large central metro, large fringe metro, medium metro, micropolitan, and noncore [29]. The metropolitan area includes a large metro (with a metropolitan statistical area (MSA) population of 1 million or more; divided into large central metro, large fringe metro); medium metro (with MSA population between 250,000 and 999,999), small metro (with MSA population less than 250,000). Non-metropolitan areas include micropolitan (with urban cluster population between 10,000 and 49,999) and noncore.

Obesity prevalence and prevalence of smokers in 2018 (% of the population that was obese) were obtained from the CDC’s Behavioral Risk Factor Surveillance System (BRFSS) [30].

### Statistical methods

#### Association study

We assessed the associations between individual PFAS and county-level cancer incidence rates using negative binomial regression adjusting for county-level SES variables, urbanicity, smoking rate, obesity, and air pollution. We chose the negative binomial model due to overdispersion which violated the assumption of the Poisson model. We calculated incidence rate ratios (IRR) and 95% confidence interval. We conducted the following analysis:

1. Detection of PFAS (2013–2015) and county-level annual average cancer incidence rate per 100,000 (2016–2021)
2. Detection or Violations of MCLs of PFAS (2023–2024) and county-level annual average cancer incidence rate per 100,000 (2016–2021)

$$\log \left({Y}_{i}\right)={\beta }_{0}+{\beta }_{1}{{PFAS}}_{i}+{\beta }_{c}{C}_{i}+\log ({{Population}}_{i})$$

Where Y was the incidence rate of cancer,

PFAS was the detection of or MCL violation of PFAS in drinking water,

C was the potential confounders described above,

Population is the population size for each county

We adjusted for multiple tests using the false discovery rate (FDR) method across major cancer systems for each PFAS. We used a significance threshold of 0.05 for FDR-adjusted *p*-values. However, given the exploratory nature of the study, we presented all findings at the crude *p*-value of 0.05.

To reduce the number of false positive associations, we only evaluated and presented the associations between PFAS and subtypes of cancers within each major organ system when PFAS was associated with the cancers of that major organ system at a crude *p*-value of 0.05. However, full summary statistics were provided for reference across all-cancer subtypes. We did not further conduct multiple testing corrections among subtypes within a major organ system because some of the subtypes are the primary driver of the overall cancer incidence in that major organ system (i.e. lung cancer in respiratory) and some of the categories contain overlapping organs (i.e. cancer in liver and intrahepatic bile duct contains both liver and intrahepatic bile duct cancers).

To account for potential outcome clustering, we additionally conducted a sensitivity analysis using a generalized mixed-effect model by including a random intercept at the state level.

$$\begin{array}{c}\log \left({Y}_{{ij}}\right)={\beta }_{0}+{\beta }_{1}{{PFAS}}_{{ij}}+{\beta }_{c}{C}_{{ij}}+\log \left({{Population}}_{{ij}}\right)+{e}_{j}\\ {e}_{j} \sim N(0,{\sigma }_{e}^{2})\end{array}$$

Where i was each county and j was each state.

We conducted two additional sensitivity analyses (1) assessing the association between detection/MCL violation of at least 1 PFAS in drinking water and cancer incidence separately for UCMR3 and UCMR5 and (2) detection/MCL violation of PFAS in both UCMR3 and UCMR5 and cancer incidence. By collapsing different PFAS exposures, we would greatly reduce the exposure misclassification and when we assessed the continuous detection of PFAS in water systems, we can both limit the exposure misclassification and identify long-term PFAS contamination.

Additional sensitivity analyses were done to assess the influence of covariates in the model including (1) removing air pollution from the model, (2) removing obesity from the model, (3) adding the number of potential PFAS-polluting industrial sites in each county-based data obtained from the PFAS Analytic Tools [31]. Briefly, the EPA developed a dataset from various sources to show which industries may handle and/or release PFAS in their facilities, but the data does not mean that the included facilities were actively manufacturing, processing, using, or releasing PFAS.

#### Incident cancer case attributable to PFAS

We calculated attributable cancer cases if the associations were statistically significant based on a crude *p*-value cutoff of 0.05. We first calculated population attributable fraction (PAF) based on IRR:

$${PAF}=\frac{p\times ({IRR}-1)}{1+p\times ({IRR}-1)}$$

Where:

*P* is the proportion of the population exposed to PFAS.

IRR is the incidence rate ratio for the association between PFAS and cancer incidence.

The proportion of the population exposed to PFAS was calculated by the following:

$$p=\frac{\mathop{\sum }_{i=1}^{n}{{pop}}_{i}|\left({{Detection\; or\; MCL\; violation\; of\; PFAS}}_{i}=1\right)}{\mathop{\sum }_{i=1}^{n}{{pop}}_{i}}$$

Where:

*N* is the total number of counties.

Pop is the population for each county.

Detection or MCL violation of PFAS is the binary variable indicating the county had PFAS detected in their drinking water or the county’s PFAS concentration was above the MCL (1 is yes and 0 is no).

Lastly, we calculated the number of incident cancer cases attributable to PFAS:

$${Attributable\; Cases}=\frac{{Incidence\; Rate\; per}100,000\times {Total\; US\; Population}}{100,000}\times {PAF}$$

Where:

The total US population in 2023 was *n* = 339,996,563.

Incidence Rate per 100,000 was calculated as the average incidence rate for all counties.

We estimated 95% CI using the 95% CI of the effect estimates from the association study.

We then summed all-cancer cases attributable to each PFAS.

$${All\; Attributable\; Cases}=\sum {{Attributable\; Cases}}_{{PFAS}}$$

Where *Attributable Cases*_*PFAS*_ is the estimated attributable cancer cases by each PFAS.

#### Stratification by sex

Lastly, we obtained sex-specific age-adjusted yearly cancer incidence between 2016 and 2021. We then assessed the association between PFAS from UCMR3 and UCMR5 and cancer incidence rate by sex using a negative binomial model.

## Results

Figure 1 shows an example of the distribution of PFOA in UCMR3 and UCMR5 and respiratory and endocrine cancer incidence. Distribution of other cancer incidence can be found in Supplemental Figs. 1–14. Table 1 shows that we included data from 1080 counties, encompassing a population of approximately 156.1 million (roughly half of the US population). PFAS data were available for 686 counties based on UCMR3 and 663 or 665 counties based on UCMR5.

**Fig. 1:**
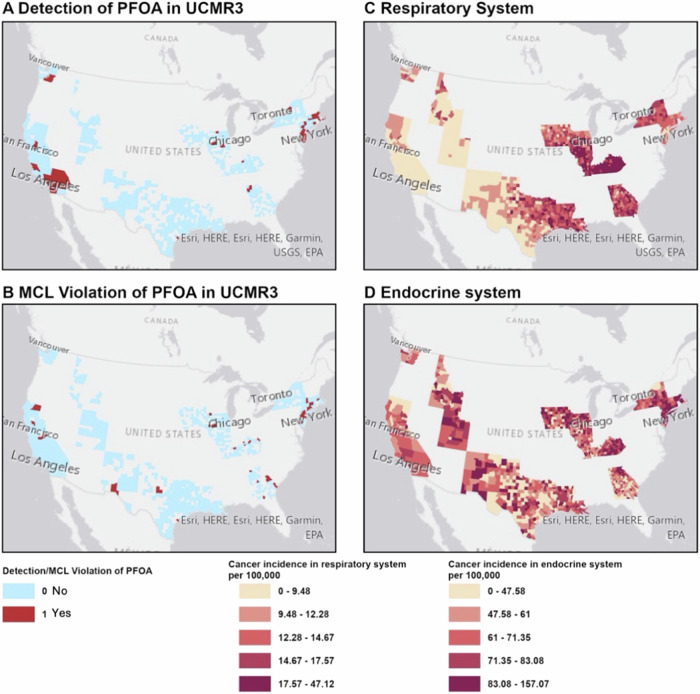
Illustration of the distribution of PFAS in drinking water and annual average cancer incidence between 2016 and 2021. **A** Detection of PFOA in drinking water based on UCMR3, **B** Maximum Contaminant Level (MCL) violation of PFOA based on UCMR5, **C** Annual average cancer incidence rate per 100,000 (between 2016 and 2021) for cancers in the respiratory system, and **D** Annual average cancer incidence rate per 100,000 (between 2016 and 2021) for cancers in the endocrine system.

**Table 1:** Characteristics of counties included in the study.

Characteristic		*N* = 1080
% People of Color	Mean (SD)	18% (15%)
% Population with Education below High School	Mean (SD)	16% (7%)
% Population below Federal Poverty Line	Mean (SD)	17% (7%)
% Population that were unemployed	Mean (SD)	6.6% (2.8%)
Mean Air Pollution level in 2019 (μg/m^3^)	Mean (SD)	6.43 (1.53)
Urbanicity	*n* (%)	
Large central metro		32 (3.0%)
Large fringe metro		137 (13%)
Medium metro		141 (13%)
Small metro		127 (12%)
Micropolitan		213 (20%)
Noncore		430 (40%)
% Population Currently Smoke	Mean (SD)	25.3% (4.1%)
Missing		2
% Population Obese	Mean (SD)	32% (6%)
Missing		21
**UCMR3**	*n* (%)	
Detection of PFOA		51 (7.4%)
Missing		394
Detection of PFOS		38 (5.5%)
Missing		394
Detection of PFHpA		38 (5.5%)
Missing		394
Detection of PFHxS		30 (4.4%)
Missing		394
Detection of PFNA		7 (1.0%)
Missing		394
Detection of PFBS		4 (0.6%)
Missing		394
**UCMR5**	*n* (%)	
Detection of PFBA		260 (39%)
Missing		415
Detection of PFHXA		194 (29%)
Missing		417
Detection of PFBS		187 (28%)
Missing		417
Detection of PFPEA		221 (33%)
Missing		417
Detection of PFHpA		78 (12%)
Missing		417
**UCMR5**	*n* (%)	
MCL violation of PFOA		51 (7.7%)
Missing		417
MCL violation of PFOS		47 (7.1%)
Missing		417
MCL violation of PFHxS		14 (2.1%)
Missing		415
MCL violation of PFNA		0 (0%)
Missing		415

At the water system level, when we looked at the six overlapping PFAS (PFOA, PFOS, PFHpA, PFHxS, PFNA, and PFBS) and their detection based on UCMR3 and UCMR5 across 2351 water systems, the majority of the PFAS were not detected in both UCMR3 and UCMR5 (87%) and roughly 1% had detection both in UCMR3 and 5. 12% were only detected by UCMR5 and about 1% only by UCMR3. At the water system levels, data from UCMR3 and UCMR5 were fairly correlated (Pearson’s correlation coefficient: 0.16, *p*-value < 2.2e-16).

At the county level, the most detected PFAS in drinking water based on UCMR3 were PFOA and PFOS, followed by PFHpA, PFHxS, PFNA, and PFBS (7.4%, 5.5%, 5.5%, 4.4%, 1%, and <1%, respectively). Based on UCMR5, violations of the MCL for PFOA, PFOS, and PFNA were 7.7%, 7.7%, and 2.1%, respectively, while detections of PFBA, PFPeA, PFHxA, PFBS, and PFHpA were 33%, 29%, 28%, 33%, and 12%.

### Associations between PFAS and cancers

**Fig. 2:**
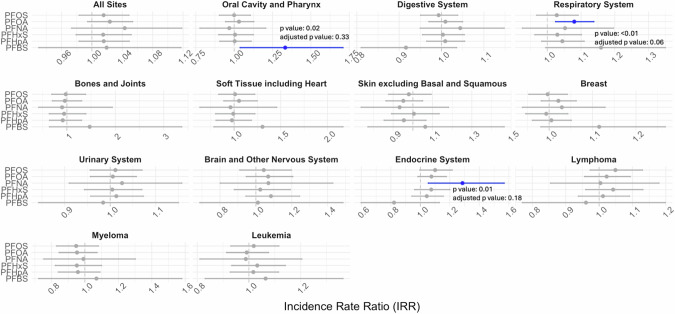
Associations between annual average county-level age-adjusted cancer incidence rate (per 100,000 persons per year) between 2016 and 2021 and detection or Maximum Contaminant Level (MCL) violation of per- and polyfluoroalkyl substances (PFAS) in drinking water based on UCMR3. For UCMR3, all PFAS were categorized into detected/not detected. The lines for statistically significant results are shown in blue.

**Fig. 3:**
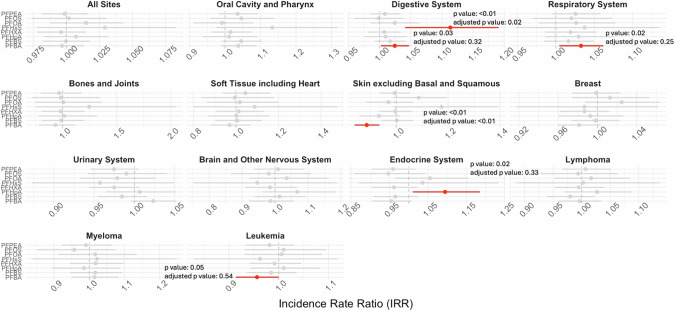
Associations between annual average county-level age-adjusted cancer incidence rate (per 100,000 persons per year) between 2016 and 2021 and detection or Maximum Contaminant Level (MCL) violation of per- and polyfluoroalkyl substances (PFAS) in drinking water based on UCMR5. For UCMR5, all PFAS were categorized into Detected/Not Detected except for PFOS, PFOA, PFHxS, and PFNA which were categorized into above Maximum Contaminant Level or Not (4 ng/L for PFOS and PFOA and 10 ng/L for PFHxS and PFNA). The lines for statistically significant results are shown in red.

Figures 2 and 3 show the associations between PFAS detection in drinking water based on UCMR3 (exposure period: 2013–2015) and UCMR5 (exposure period: 2023–2024) and cancer incidence rates (2016–2021). Overall, we found four types of cancers were positively associated with the detection of or an MCL violation of at least one PFAS based on UCMR3 or UCMR5 data including the digestive system, endocrine system, oral cavity and pharynx, and respiratory system (IRR ranges: 1.02–1.33; see Supplemental Table 1–3).

The largest effect was observed between the detection of PFBS and cancers in the oral cavity and pharynx (IRR: 1.33 [1.04, 1.71], *p*-value: 0.02, FDR-adjusted *p*-value: 0.33). This was followed by the detection of PFNA in UCMR3 and PFHpA in UCMR5, which were associated with cancers in the endocrine system, mainly thyroid cancers (IRRs: 1.28 [1.04, 1.57], *p*-value: 0.01, FDR-adjusted *p*-value: 0.18 and 1.10 [1.01, 1.19], *p*-value: 0.02, FDR-adjusted *p*-value: 0.33).

Detection of PFBA and MCL violations of PFHxS in UCMR5 were positively associated with digestive system cancers. Specifically, these cancers were located in the large intestine (IRR: 1.20 [1.07, 1.34]) and liver (IRR: 1.10 [1.03, 1.18]) for PFBA. For PFHxS, the cancers were located in the esophagus (IRR: 1.37 [1.09, 1.72]), colon and rectum (IRR: 1.12 [1.03, 1.21]), rectosigmoid junction (IRR: 1.36 [1.00, 1.83]), and gallbladder (IRR: 1.60 [1.06, 2.41]; see Supplemental Tables 4–6).

Additionally, the detection of PFOA in UCMR3 and PFBA in UCMR5 was associated with respiratory system cancers, mainly lung cancers (IRRs: 1.08 [1.02, 1.14] and 1.03 [1.01, 1.06]).

Lastly, the detection of PFBA was associated with a reduced risk of skin cancer and leukemia (IRRs: 0.89 [0.84, 0.93], *p*-value: <0.01, FDR-adjusted *p*-value: <0.01 and 0.96 [0.91, 1.00], *p*-value: 0.05, FDR-adjusted *p*-value: 0.54). Full summary statistics are available in Supplemental Table 1–6.

### Cancer cases attributable to PFAS

As shown in Table 2, we estimated that the detection of PFBS, PFNA, and PFOA would contribute to 4626 [95% CI: 1377, 8046] incident cancer cases each year based on UCMR3 data while based on UCMR5 data, detection of PFBA and PFHpA and MCL violation of PFHxS would contribute to 6864 [95% CI: 991, 12,804] incident cancer cases each year.

**Table 2:** Attributable cancer incidence per year due to detection of PFAS in drinking water based on UCMR3 and UCMR5 data.

PFAS^a,b^	Period	Cancers	Attributable Cases [95% CI]
Detection of PFBS	UCMR3	Oral Cavity and Pharynx	28 [3,60]
Detection of PFNA	UCMR3	Endocrine System	567 [108, 1114]
Detection of PFOA	UCMR3	Respiratory System	4031 [1266, 6872]
**Total**	**UCMR3**		**4626 [1377, 8046]**
Detection of PFBA	UCMR5	Digestive System	2668 [310, 5036]
Detection of PFBA	UCMR5	Respiratory System	2786 [448, 5137]
Detection of PFHpA	UCMR5	Endocrine System	1213 [160, 2299]
MCL Violation of PFHxS	UCMR5	Digestive System	198 [73, 332]
**Total**	**UCMR5**		**6864 [991, 12804]**

Total cancer cases were bolded.

^a^Total US population in 2023 was 339,996,563, the baseline all-cancer incident rate was 446.247 per 100,000 and the total cancer cases were 1,517,225.

^b^Skin cancers were excluded from the calculations of the total cancer cases.

### Mixed-effect model

When we conducted a generalized linear mixed model with a random intercept at the state level (as a sensitivity analysis), the detection of PFOA in UCMR3 was associated with an increased risk of lung cancers (IRR: 1.06 [1.01, 1.11]). In UCMR5, we observed associations between PFHxS and an increased risk of digestive system cancers (IRR: 1.12 [1.05, 1.19]), PFHpA and endocrine system cancers (IRR: 1.10 [1.02, 1.19]), and both PFPeA and PFHpA with respiratory system cancers (IRRs: 1.03 [1.00, 1.06] and 1.04 [1.00, 1.08]). Overall, the results were fairly similar to our main analysis. See Supplemental Tables 7–9 for all summary statistics.

### Sensitivity analysis

As shown in Supplemental Tables 10 and 11, when we collapsed detection/MCL violation of PFAS in drinking water into one variable (at least 1 PFAS detected/had MCL violation), detection of at least 1 PFAS in drinking water was associated with cancers in the respiratory system based on both UCMR3 and 5. However, none of the associations were significant at an FDR-adjusted *p*-value threshold of 0.05. As shown in Supplemental Table 12, only detection/MCL violation of PFOA in both UCMR3 and 5 was associated with increased risk of cancers in all-cancer incidence and cancer incidence in the endocrine system and reduced risk of skin cancer.

When we removed air pollution from the model, we found that the detection of PFOA in UCMR3 was positively associated with cancers in the respiratory system, and the detection of PFBA was negatively associated with skin cancer at FDR-adjusted *p*-value of 0.05 (Supplemental Table 13). When we removed the prevalence of obesity, we found that MCL violation of PFHxS in UCMR5 was positively associated with cancers in the digestive system, detection of PFBA in UCMR5 was negatively associated with cancers of the skin and endocrine system, detection of PFOA in UCMR3 was positively associated with cancers in the respiratory system at FDR-adjusted *p*-value of 0.05 (Supplemental Table 14).

When we additionally controlled for the number of potential PFAS-polluting facilities in the county, MCL violation of PFHxS in UCMR5 was positively associated with the digestive system, detection of PFBA in UCMR5 was negatively associated with skin cancer, and detection of PFOA in UCMR3 was positively associated with respiratory cancer at FDR-adjusted *p*-value of 0.05 (Supplemental Table 15).

### Sex-stratified analysis

Among males, we found four types of cancers were positively associated with the detection or an MCL violation of PFAS based on UCMR3 or UCMR5 data including cancers in the urinary system, brain and other nervous system, leukemia, and soft tissues. Among females, we found three types of cancers were positively associated with the detection or an MCL violation of PFAS based on UCMR3 or UCMR5 data including cancers in the endocrine system, oral cavity and pharynx, and soft tissue.

Based on UCMR3, we found significant associations between the detection of PFHpA and increased risk of brain cancers (IRR: 1.22 [1.02, 1.47]), the detections of PFHxS and PFOS and increased risk of cancers in the urinary system (IRRs: 1.11 [1.00, 1.22], 1.10 [1.01, 1.20]), the detection of PFOS and reduced risk of myeloma (IRR: 0.8 [0.70, 0.99]) among males. In females, we found associations between the detection of PFOA and increased risk of cancers in the oral cavity and pharynx as well as reduced risk of skin cancer (IRR: 1.20 [1.03, 1.39], 0.85 [0.73, 0.98]) (see Supplemental Table 16).

Based on UCMR5, MCL violations of PFOS, and PFOA and increased risk of cancers in soft tissue in both male and female groups (male: 1.66 [1.33, 2.06], 1.75 [1.42, 2.16]; female: 1.56 [1.24, 1.97], 1.32 [1.05, 1.67]).

Among males, we found associations between MCL violation of PFOA and detection of PFHpA and increased risk of brain cancers (IRRs: 1.20 [1.02, 1.40] and 1.15 [1.00, 1.32]), MCL violations of PFHxS and PFOS and detection of PFPeA and increased risk of leukemia (1.86 [1.50, 2.29], 1.19 [1.05, 1.34], and 1.09 [1.01, 1.17]), detections of PFBS, PFPeA, PFHxA, and MCL violation of PFHxS and increased risk of cancers in soft tissues (IRRs: 1.19 [1.02, 1.37], 1.24 [1.07, 1.43], 1.28 [1.10, 1.48], 4.13 [2.93, 5.82]), and MCL violation of PFHxS and increased risk of cancers in urinary system (IRR: 1.28 [1.12, 1.47]). We also found that MCL violation of PFHxS was associated with a reduced risk of lymphoma and cancers in the male genital system among males (IRRs: 0.75 [0.63, 0.91] and 0.86 [0.77, 0.96]).

Among females, we found detections of PFHpA, PFHxA, and PFPeA were associated with endocrine cancers (IRRs: 1.15 [1.03, 1.29], 1.11 [1.01, 1.21] and 1.09 [1.00, 1.19]) and MCL violation of PFOS and cancers in the oral cavity and pharynx (IRR: 1.25 [1.07, 1.46]) (see Supplemental Tables 17 and 18).

## Discussion

Our study was the first ecological study to assess cancer-wide associations with PFAS exposure in drinking water using cancer incidence data from SERR between 2016 and 2021 and PFAS data in drinking water from UCMR3 (2013 to 2015) and UCMR5 (2023 to 2024). We found significant associations between PFAS in drinking water including PFOS, PFBS, PFHxS, PFOA, PFNA, PFBA, and PFHpA, and increased incidence rates of cancers in oral and pharynx, digestive, respiratory, and endocrine systems. In the sex-stratified analysis, we found that in males PFAS were associated with cancers in the urinary system, brain and other nervous system, leukemia, and soft tissues, and in females, PFAS were associated with the endocrine system, oral cavity and pharynx, and soft tissue. Some of the cancers identified in our study have not been studied for their associations with PFAS. In addition, based on UCMR3 and 5, we estimated that 4626 [95% CI: 1377, 8046] and 6864 [95% CI: 991, 12,804] cases were attributed to PFAS in drinking water.

Drinking water is a significant route of PFAS exposure for the general population [2]. Recent studies further corroborate that PFAS levels in the blood are driven by drinking water exposure using matched tap water and plasma samples [32, 33]. Existing literature provides robust evidence linking PFAS to kidney cancer and suggestive evidence for breast and testicular cancers [11, 34]. However, most studies have focused on PFOS and PFOA, with limited research on other PFAS and their associations with cancers, and there is limited research assessing source-specific health effects of PFAS, especially from drinking water [15–17, 35, 36].

### Digestive system

Our study also found that detection of PFBA and MCL violation of PFHxS in drinking water were associated with increased risks of cancers in the digestive system, including the esophagus (esophageal cancer), colon and rectum (colorectal cancer), rectosigmoid junction, liver (liver cancer/hepatocellular carcinoma), and gallbladder cancers. Although previous studies did not identify associations between PFHxS and PFBA with esophageal cancer, a recent National Health and Nutrition Examination Survey (NHANES) study using data from 2003 to 2018 suggested increased risks of esophageal cancer with PFOA and PFOS exposure [37]. Since PFAS-contaminated drinking water would directly expose tissues in the esophagus, the associations with PFBA and PFHxS are thus plausible and deserve further investigation.

For liver cancer, consistent evidence from both rodent and human observational studies suggests that PFAS can cause liver injury [38], leading to a higher risk of liver cancer [39]. In a mouse study, exposure to PFBA showed increased liver weight and changes in gene expression in the liver [40]. One previous study also suggested that PFBA was preferentially accumulated in liver and lung cancers [41]. Several studies have also demonstrated that PFAS can disrupt lipid metabolism, which plays a crucial role in the development and progression of liver cancer [42–45]. Our study adds evidence that the detection of PFBA was associated with an increased risk of liver cancer.

We found that violation of MCL of PFHxS was associated with colorectal cancers specifically in males. NASEM identified an association between PFAS exposure and ulcerative colitis, a chronic inflammatory bowel disease (IBD) [11]. Patients with IBD are at increased risk of colorectal cancer and intestinal lymphoma [46]. Experimental studies in mice have shown that ingestion of PFOA-treated water may induce alterations in epigenetic and tight junction genes in the small intestine and colon, as well as changes in the gut microbiome [47–49]. The mucosal barrier and gut microbiota collectively maintain the homeostasis of the gastrointestinal (GI) tract, and damage to the intestinal barrier can lead to colorectal cancer [50, 51]. However, human observational studies of PFAS and colorectal cancer found mixed results. One study in the mid-Ohio Valley showed an inverse association between PFOS and colorectal cancer [52]. In a Swedish cohort, residents living in highly contaminated areas had elevated cancer risk in the rectum [15]. More studies are needed to evaluate colorectal cancer risk due to PFAS in both human and animal studies.

### Respiratory system

We found that detections of PFOA and PFBA were associated with an increased risk of lung cancer. In sex-specific analysis, we found several PFAS associated with cancers in the lung including pleura and trachea in both males and females, although these PFAS were not associated with cancer in the entire respiratory system. Moon and Mun (2024) found that PFOS and PFNA were associated with an increased risk of lung cancer (ORs: 2.62 [1.24–5.83] and 2.38 [1.00–5.52]). In the Swedish cohort, living in areas contaminated with PFAS was associated with lung cancer risk among men but not among women [15]. In a study among lung cancer patients, PFAS including PFOA in blood was associated with multiple clinical indicators related to immune and liver functions [53]. Exposure to PFAS, through both inhaled and non-inhaled pathways, can target the lungs and modify lung functions and inflammatory responses. A study exposing human bronchial epithelial cells to PFOA and PFOS showed activated inflammasome, altered membrane properties of cells, and effects on inflammation- and immune-related genes [54]. In addition, PFBA was detected at a higher concentration in liver and lung tissues in humans [41]. Therefore, it is plausible that PFOA and PFBA cause lung cancer development.

### Endocrine system

We observed that detections of PFNA and PFHpA were associated with an increased risk of thyroid cancer and the associations were primarily in females. PFAS are also considered endocrine-disrupting chemicals (EDCs) and can therefore increase the cancer risk of hormone-sensitive organs, including the thyroid [55]. PFAS may disrupt the thyroid hormone system based on in vitro and animal studies, leading to impaired thyroid function and an increased risk of thyroid cancer [56, 57]. Other studies also found that exposure to PFOA and PFOS was associated with disrupted thyroid hormones and alterations in thyroid gene expression [58, 59]. However, epidemiological evidence for the associations between PFAS and thyroid cancer is inconsistent. One case-control study in China found that PFNA, PFDA, and PFUnDA were associated with a lower risk of thyroid cancer [60]. Similar inverse associations were found in several studies, including a Finnish study [61] and another study in China [62]. Conversely, a recent study in the US found positive associations between PFOS and thyroid cancer [63]. Our study further suggests that there is a potential interaction between female sex and PFAS associations with thyroid cancer development. Since thyroid cancer is more common in females than males, it is plausible that sex hormone plays a role in the development of thyroid cancer and thus can interact with PFAS leading to greater thyroid cancer risk [64].

### Urinary system

In males, we also found that PFOS and PFHxS were associated with cancers in the urinary system including kidney cancer and bladder cancer. A recent systematic review identified an overall association between PFOA and PFOS and kidney cancer [10]. Although we did not observe associations between kidney cancer and PFOA in our main analysis, in sex-stratified analysis the IRR [95% CI] between PFOA and kidney cancer was 1.09 [0.99, 1.19], *p*-value: 0.08 in UCMR3, comparable to the C8 study associations (HR: 1.10; 95% CI: 0.98, 1.24) [16, 17]. Studies included in the systematic review also found sex-specific associations between PFAS and kidney cancers in males only [10].

Previous evidence suggests that the kidney is a major pathway for PFAS elimination from the human body and PFAS can cause damage to kidney function over time leading to possible kidney cancer [65]. Future studies should consider evaluating PFAS other than PFOA and PFOS to assess nephrotoxicity and sex-specific effects of these PFAS on kidneys.

### Hematologic system (blood and lymphatic)

We found inverse associations between the detection of PFBA and leukemia, but sex-specific effect estimates suggest that MCL violations of PFHxS and PFOS and the detection of PFPeA were positively associated with leukemia in males. Two epidemiological studies found PFAS were associated with leukemia in childhood or adulthood [35, 66] and an Italian study also found higher leukemia in males only [35].

### Nervous system

In the male group, we also found several PFAS including detection of PFHpA and violation of MCL of PFOA were associated with brain cancer. Growing evidence suggests that PFAS have neurotoxicity. Previous experimental studies show that PFAS may cross the blood-brain barrier to cause damage to brain cells and disrupt neurotransmission [67]. PFAS was found in 96% of the samples of brain tissues from brain cancer patients [68]. Moon and Sun (2024) also found that PFHxS was associated with increased brain cancer in NHANES.

### Skin and soft tissue

We found inverse associations between the detection of PFBA and skin cancer, which are not plausible. Dermal exposure to consumer products contains high concentrations of PFAS (eg. 22–10,500 ng/g product weight in cosmetics found in North America) [69]. PFAS has been detected in almost all cosmetics and personal care products [70]. In addition, the SEER database did not cover some states with higher PFAS contamination. More studies are needed to evaluate the effects of PFAS on skin cancer.

We also found that PFAS was associated with cancer in soft tissues. Few previous studies have identified a link between PFAS and soft tissue [71–73]. Therefore, further study is needed on the effects of PAS on specific soft tissue.

### Head and neck

Our study identified significant associations between the detection of PFBS in drinking water and an increased risk of oral and pharynx cancer. Among females, detection of PFOA was associated with oral and pharyngeal cancers. EPA’s toxicity assessment of PFBS through evidence synthesis of existing experimental and epidemiological studies and calculation of toxicity levels suggested that PFBS is less toxic than PFOA and PFOS and in humans, only asthma and serum cholesterol levels were associated with PFBS [74]. Previous studies often reported null associations between PFBS as well as other PFAS and oral/pharynx cancers [75], perhaps due to small sample sizes since oral and pharyngeal cancers are relatively rare, accounting for about 3% of all-cancer cases [76]. Diet and drinking water are the primary routes of PFAS exposure [77], leading to direct contact with PFAS in the mouth and pharynx. Mechanistic studies on PFAS and oral/pharynx cancer are limited. However, PFAS may induce cancer through mechanisms such as oxidative stress and DNA damage in squamous cells [6] or by increasing permeability and damaging the epithelial cells lining the oral cavity and pharynx, creating an environment for cancer development [78]. More research is needed to confirm our findings, as no previous studies have specifically examined PFAS effects on oral or pharyngeal cells.

### Eye and orbit

Although we were not able to examine the associations between PFAS and cancers in the eye and orbit, there has been a recent study on PFAS measured in metabolomics and retinoblastoma in childhood [79] and they found that PFOS was associated with retinoblastoma and PFOA was associated with retinoblastoma only in children of US-born mothers. Therefore, future studies can further assess the associations between PFAS and retinoblastoma in adulthood to strengthen the findings.

### Inconsistency of results between UCMR3 and UCMR5

We observed differences in the results when using UCMR3 and UCMR5. First, since we observed that more water systems had detection of PFAS in UCMR5, it is unlikely that those water systems recently increased in PFAS contamination due to increased attention to PFAS contamination and the recent announcement of EPA’s rule on MCL of six PFAS [4]. The more likely explanation is the improved quantification which resulted in much lower detection limits in UCMR5 than UCMR3 and thus UCMR5 might be a more accurate measure of PFAS levels in drinking water. However, it is also possible that exposure in UCMR5 is misclassified which leads to differences in the results (described below in the limitation).

### Sex-specific PFAS and cancer associations

We found that different cancers were associated with PFAS by sex. PFAS have been previously found to have sex-specific effects on various health outcomes including cardiovascular diseases, cholesterol metabolism, liver disease, and neurodevelopment [80–82]. In PFAS and cancer research, in an analysis in NHANES, PFAS were only associated with melanoma among women but not among men [83]. In addition, in another prospective study of cancer, they found that PFOA was associated with renal cell carcinoma among women while PFHxS was associated with leukemia among men [84]. There have been studies suggesting that half-lives of PFAS were different in males and females which were not explained by reproductive factors [85, 86]. In addition, there have been studies suggesting sex-specific effects of PFAS on biological mechanisms such as cholesterol metabolism, liver injury, and inflammatory biomarkers [80, 87, 88]. Therefore, future studies should focus on the sex-specific effects of PFAS on cancers.

### Strength and limitations

By focusing on this source-specific PFAS exposure, we provide actionable targets for interventions, such as water quality, to policymakers. We categorized PFAS levels according to the new EPA regulatory standards (final MCLs announced in 2024). By using these new MCLs as cutoffs, our analysis gains increased policy relevance. We controlled for numerous area-level confounders and explored various modeling approaches. In our main analysis, we controlled for age and conducted sex-stratified analyses to account for confounding by sex.

Our study had several limitations. It was an ecological study analyzed at the county level, and thus we were not able to control individual-level confounders except for age and sex. We were also unable to control for potential confounders specific to each cancer type.

Although SEER covers a significant portion of the U.S. population, it is not representative of the entire country, as several states are not included in the database. In addition, SEER may not adequately capture areas with the highest levels of PFAS contamination in drinking water, which could limit the generalizability of our findings to communities most affected by PFAS. This limitation might partly explain the lack of observed associations in our study for cancers such as kidney and testicular cancers found in previous studies [36], as areas with the most severe contamination, such as Michigan [89], and potential health effects could be underrepresented. Future studies incorporating broader population-level data sources are necessary to further elucidate the relationship between PFAS exposure and cancer incidence.

In addition, we were unable to control for the lack of lagging between PFAS exposure and cancer incidence. It was particularly an issue for UCMR5 data since UCMR5 was conducted after cancer diagnosis. If we assume UCMR3 was a valid measure of PFAS exposure through water, there would be significant exposure misclassification when using UCMR5. Approximately half of the exposed water systems from UCMR3 would be misclassified as not exposed by UCMR5 while 11% of the unexposed water systems would be classified as exposed by UCMR5. However, the exposure classification would not be driven by cancer incidence and thus, it could result in bias in our effect estimates towards null.

Moreover, mitigation efforts may have been made towards water systems that have been previously detected with PFAS based on UCMR3 data and the mitigation effort may not have been made systematically resulting in further misclassification of PFAS exposure based on UCMR5.

In addition, we are unable to account for the various latency periods of cancers in our study. We are unable to assess whether PFAS monitoring data from both UCMR3 and UCMR5 was indeed a valid measure of historical levels of PFAS water contamination. Therefore, our results should be interpreted with caution.

We also did not account for multiple comparisons when conducting analyses of sub-organ systems because the cancer rate in major cancer systems may be driven primarily by one cancer (i.e. lung cancer for the respiratory system). Therefore, false positive associations were still likely.

In regions where PFAS contamination or water contamination is prevalent, people may not drink tap water. We did not have individual-level water drinking behavior data and therefore, nondifferential exposure misclassification of PFAS exposure from drinking water is possible and would bias our effect estimates towards null.

## Conclusions

This cancer-wide ecological study presents evidence linking PFAS exposure through drinking water to increased cancer risks. The significant associations identified between PFAS in drinking water and various cancers, including those of the endocrine, digestive, oral cavity, pharynx, skin, and respiratory systems, underscore the urgent need for more comprehensive research. Given the recent regulation of PFAS in drinking water by the US EPA, our findings highlight the critical importance of developing effective strategies to mitigate cancer risks from exposure to PFAS through drinking water.

## Supplementary information

Supplemental Figures

Supplemental Table 1

Supplemental Table 2

Supplemental Table 3

Supplemental Table 4

## Data Availability

Data were publicly available and downloaded from US EPA and National Cancer Institute websites. Codes were available upon request.

## Code availability

Codes were available upon request.
