# Supplementary material for: Associations between per-and polyfluoroalkyl substances (PFAS) and county-level cancer incidence between 2016 and 2021 and incident cancer burden attributable to PFAS in drinking water in the United States
Source: J Expo Sci Environ Epidemiol. 2025 Jan 9;35(3):425–36. doi: 10.1038/s41370-024-00742-2 (PMC12069088; doi:10.1038/s41370-024-00742-2)

**Supplemental Figure 1.** All cancer incidence per 100,000 between 2016 and 2021.


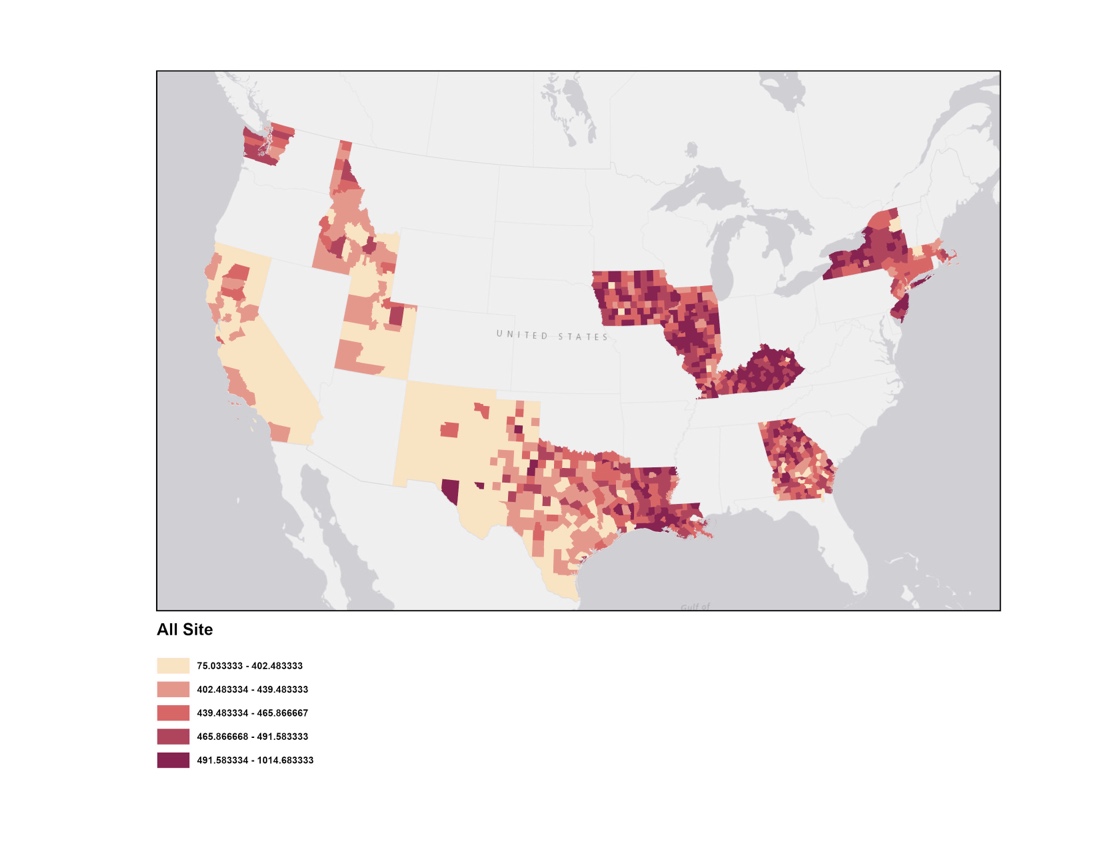


**Supplemental Figure 2.** Soft tissue cancer incidence per 100,000 between 2016 and 2021.


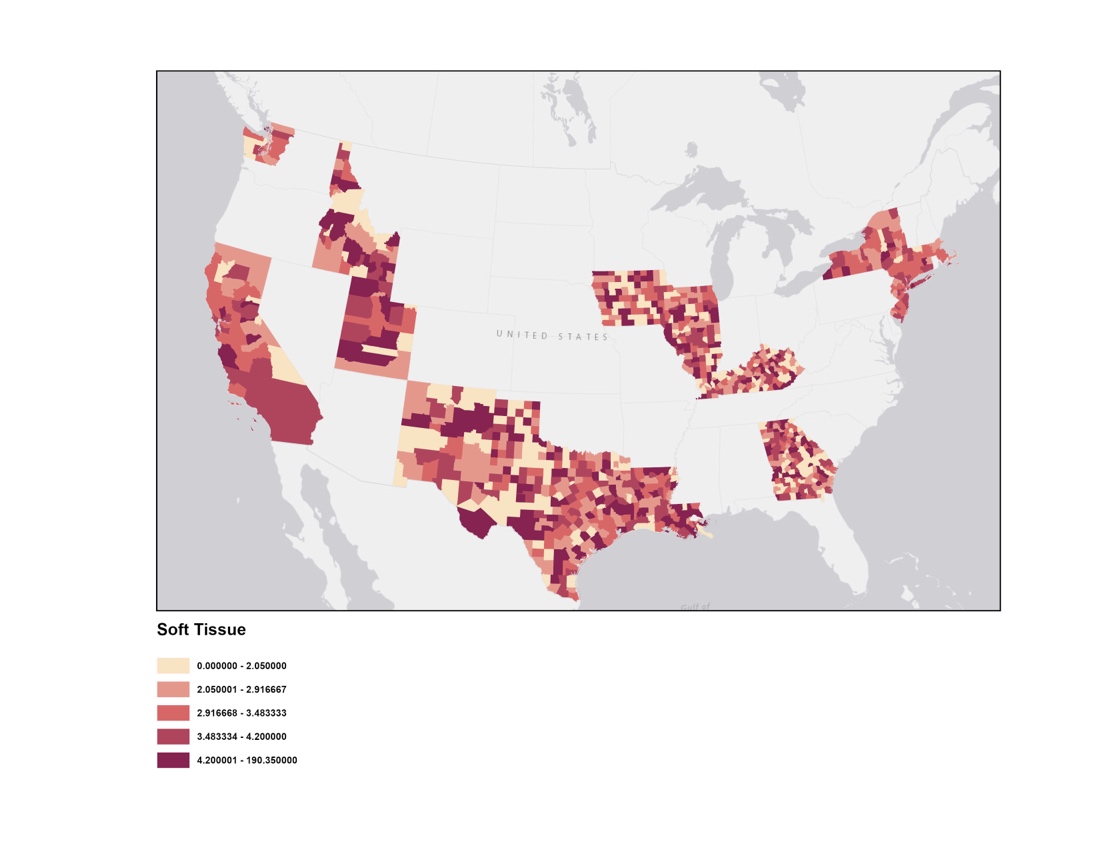


**Supplemental Figure 3.** Myeloma cancer incidence per 100,000 between 2016 and 2021.


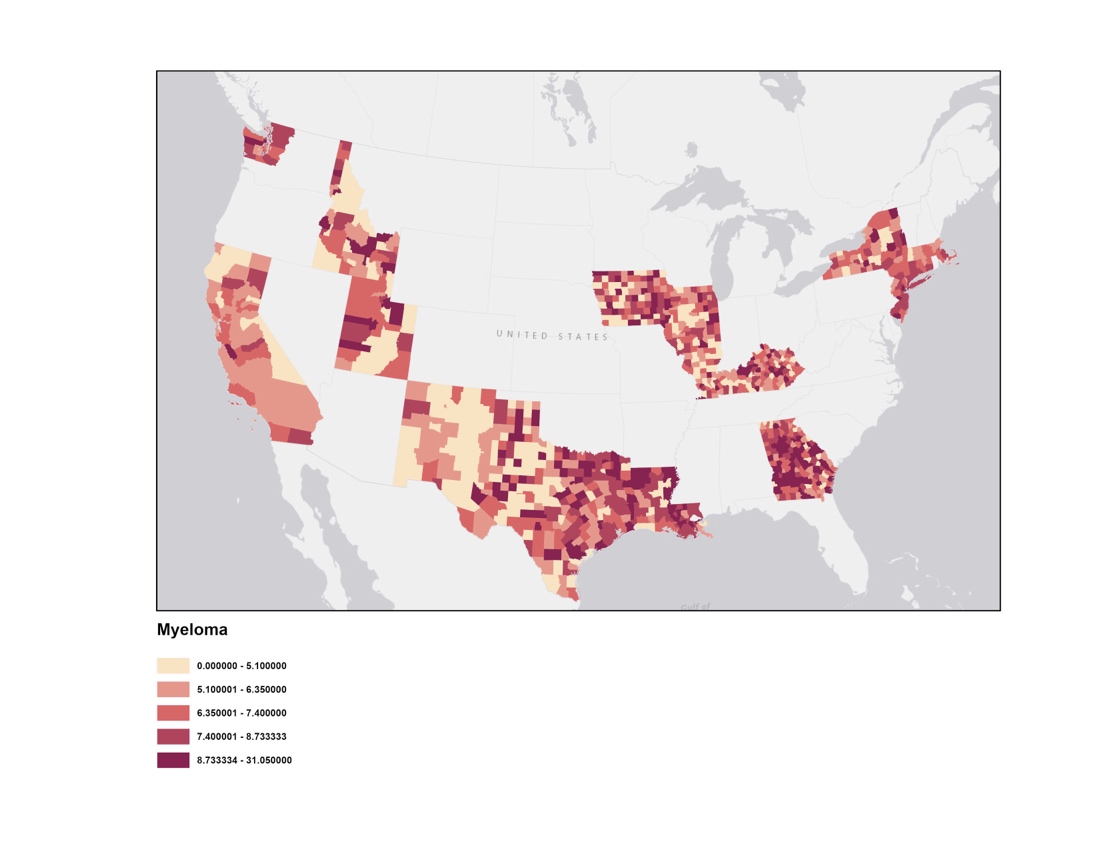


**Supplemental Figure 4.** Urinary system cancer incidence per 100,000 between 2016 and 2021.


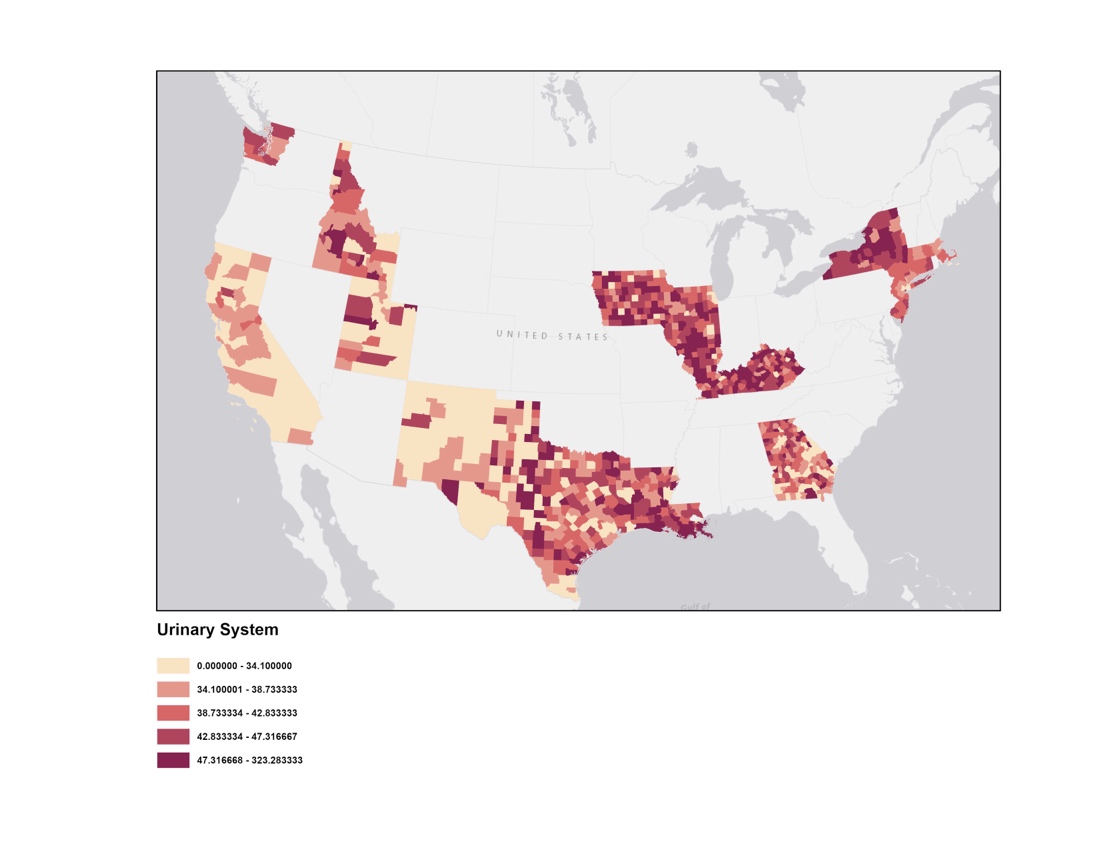


**Supplemental Figure 5.** Breast cancer incidence per 100,000 between 2016 and 2021.


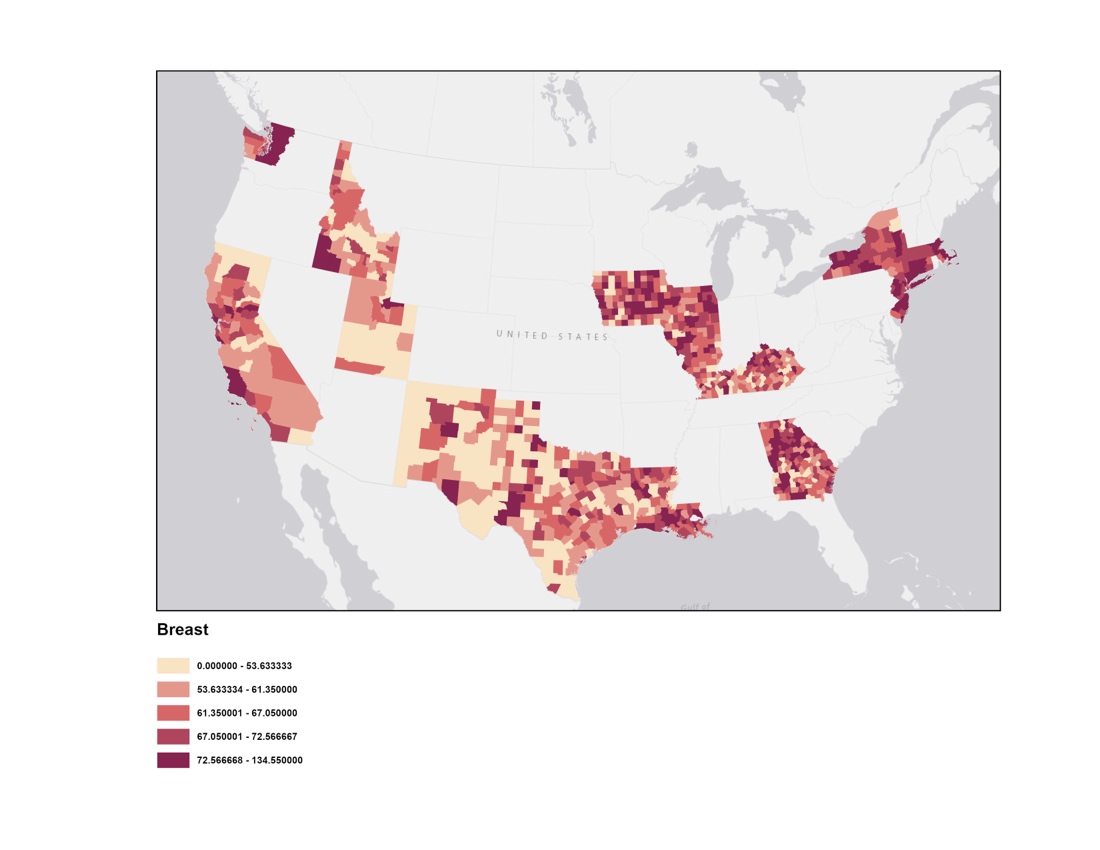


**Supplemental Figure 6.** Leukemia cancer incidence per 100,000 between 2016 and 2021.


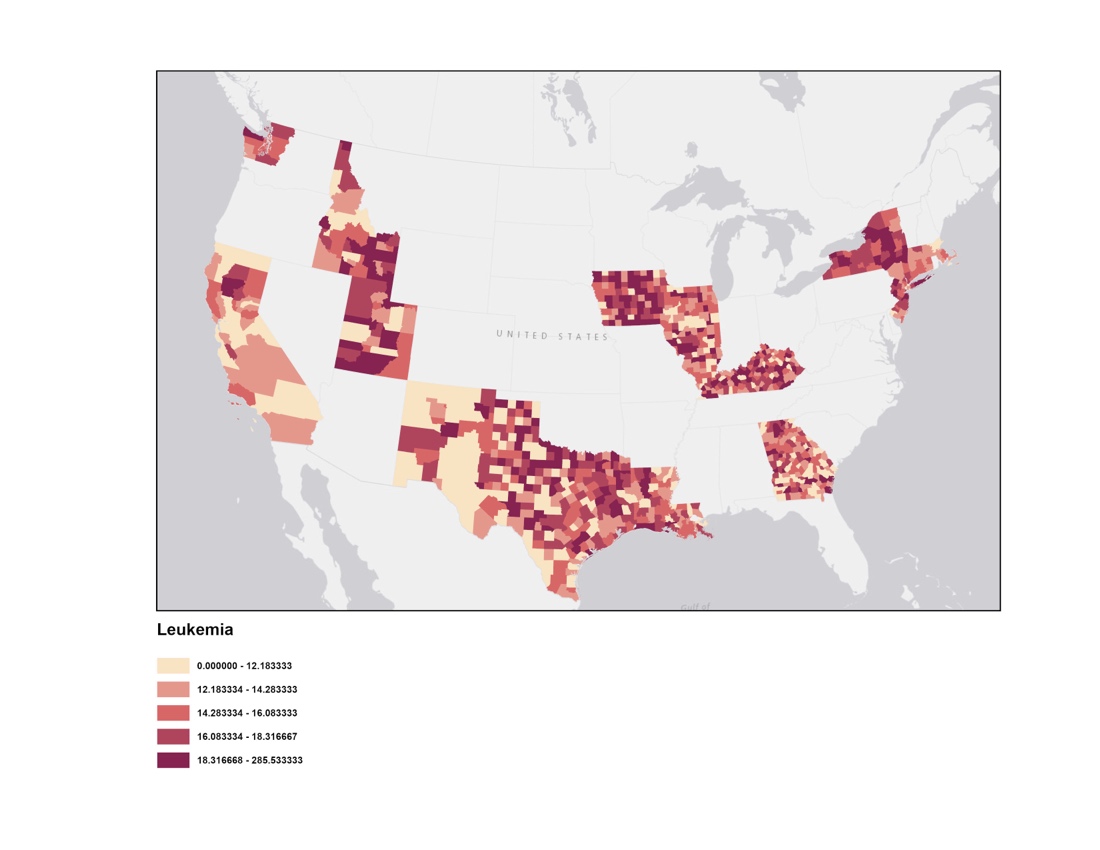


**Supplemental Figure 7.** Oral cavity and pharynx cancer incidence per 100,000 between 2016 and 2021.


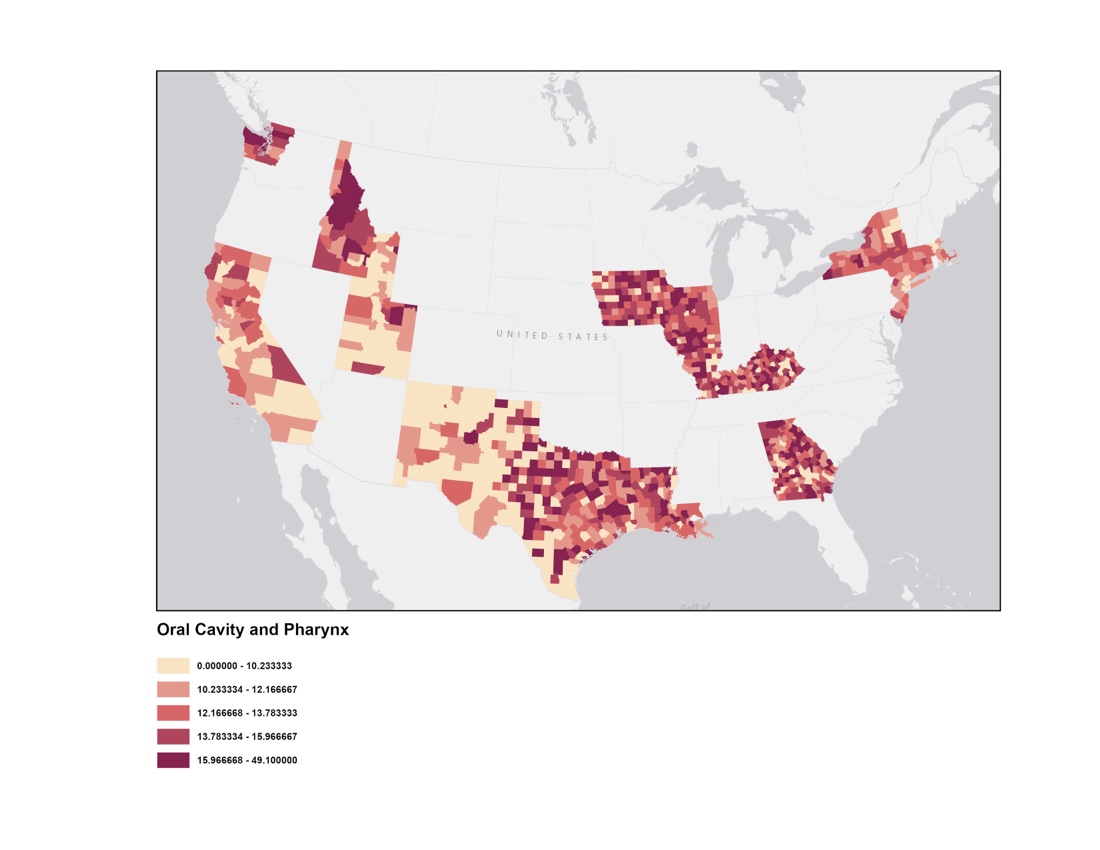


**Supplemental Figure 8.** Skin cancer incidence per 100,000 between 2016 and 2021.


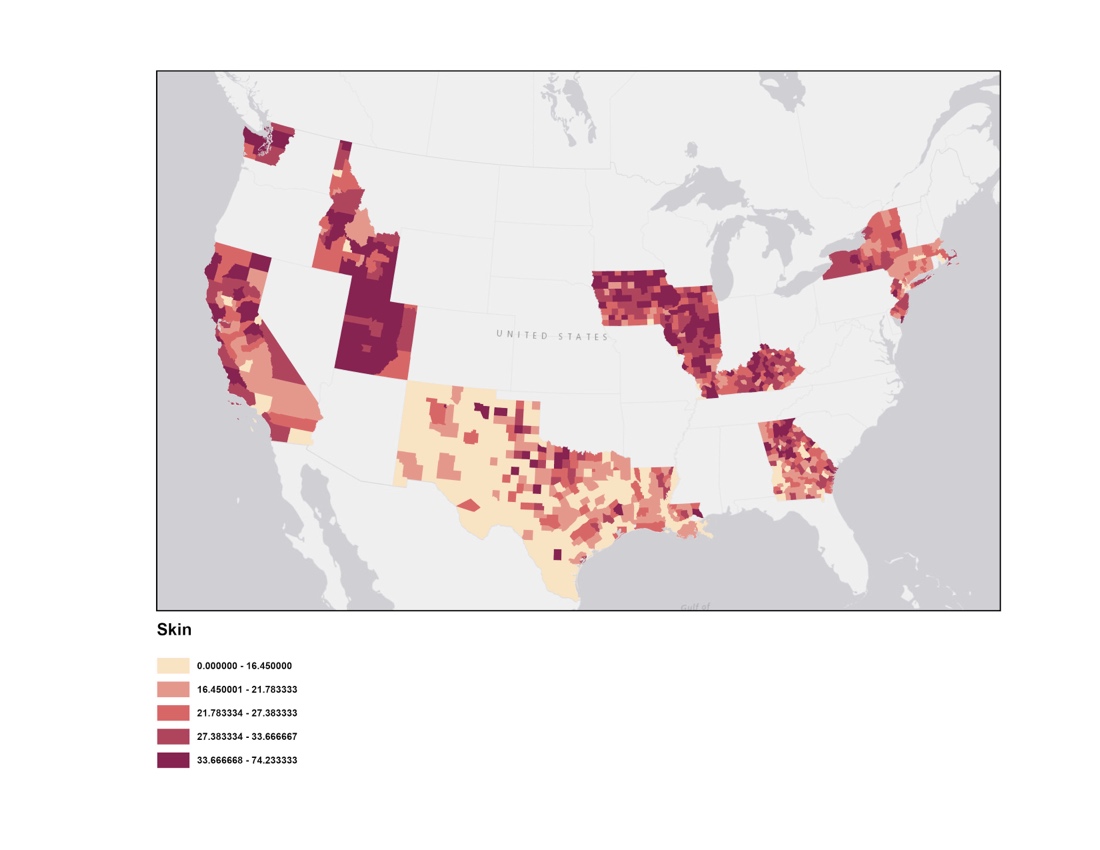


**Supplemental Figure 9.** Digestive cancer incidence per 100,000 between 2016 and 2021.


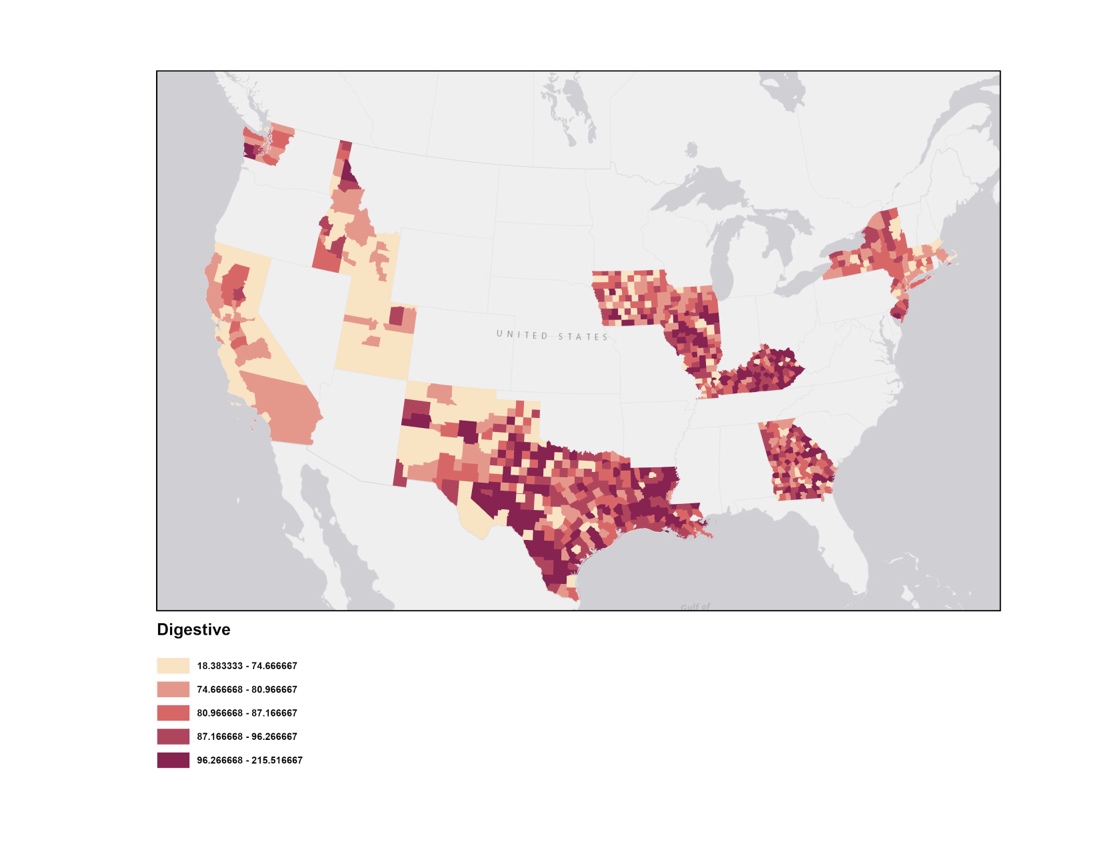


**Supplemental Figure 10.** Lymphoma cancer incidence per 100,000 between 2016 and 2021.


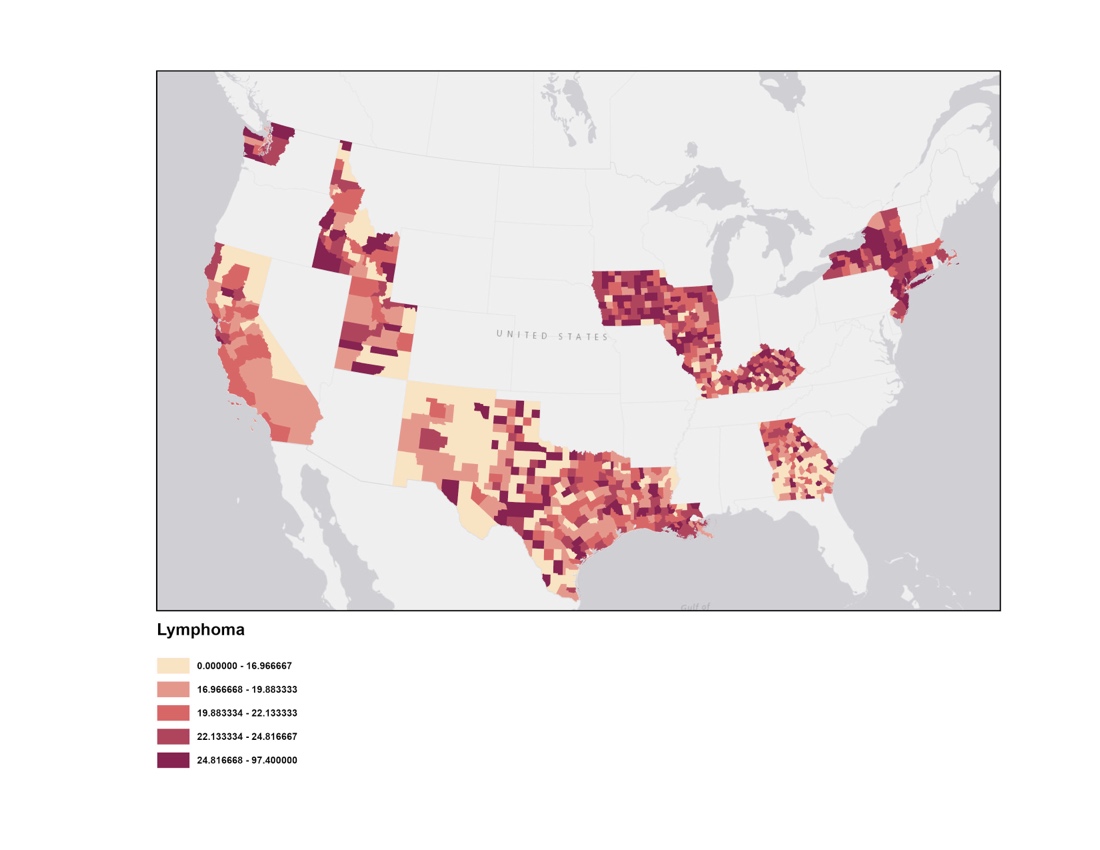


**Supplemental Figure 11.** Bones and joints cancer incidence per 100,000 between 2016 and 2021.


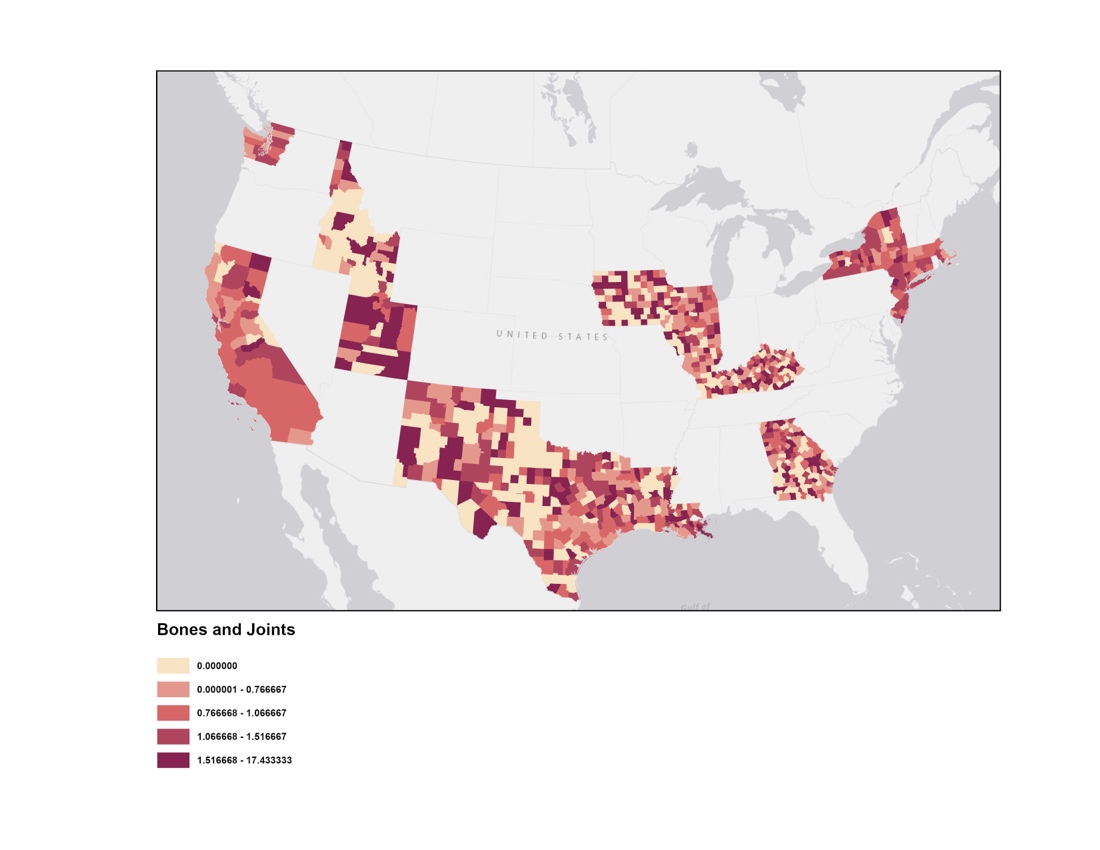


**Supplemental Figure 12.** Brain and other nervous system cancer incidence per 100,000 between 2016 and 2021.


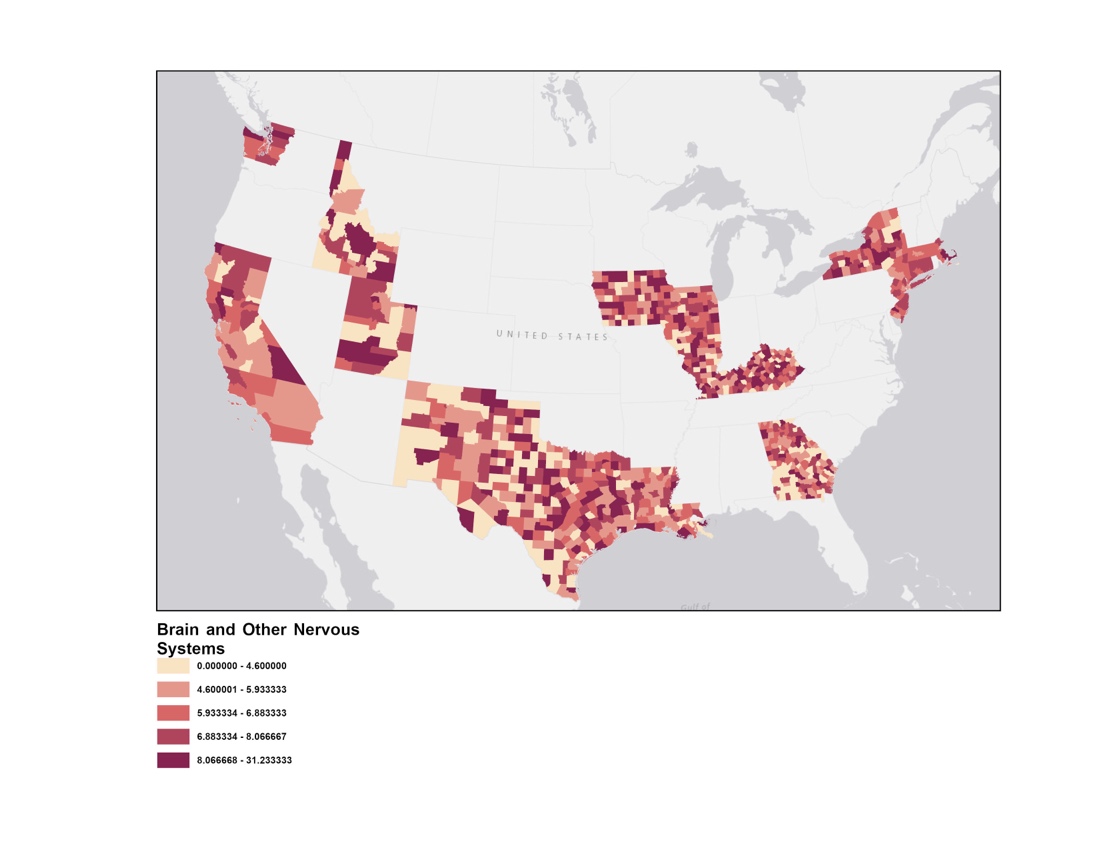


**Supplemental Figure 13.** Male genital system cancer incidence per 100,000 between 2016 and 2021.

**
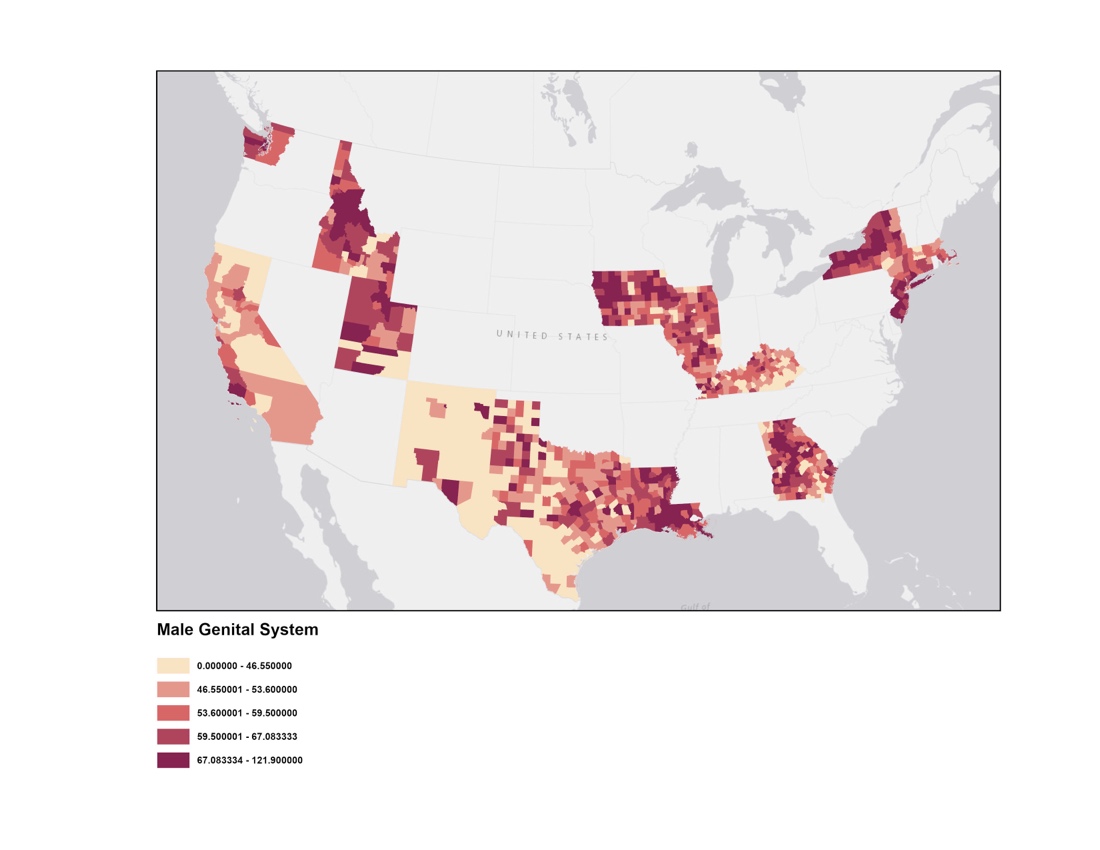
**

**Supplemental Figure 14.** Female genital system cancer incidence per 100,000 between 2016 and 2021.


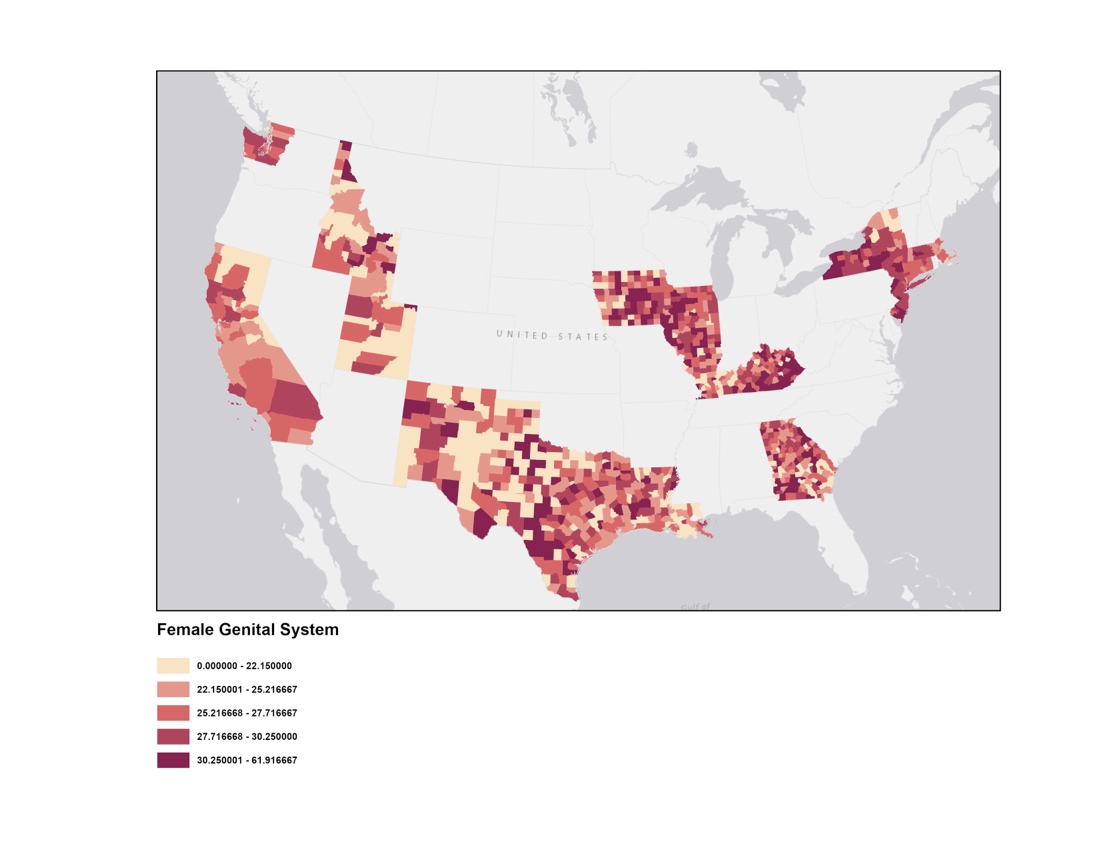

Supplement: Supplementary file 1 — Supplemental Figures [file 41370_2024_742_MOESM1_ESM.docx]
