# Supplementary material for: Associations between per-and polyfluoroalkyl substances (PFAS) and county-level cancer incidence between 2016 and 2021 and incident cancer burden attributable to PFAS in drinking water in the United States
Source: J Expo Sci Environ Epidemiol. 2025 Jan 9;35(3):425–36. doi: 10.1038/s41370-024-00742-2 (PMC12069088; doi:10.1038/s41370-024-00742-2)
Supplement: Supplementary file 2 — Supplemental Table 1 [file 41370_2024_742_MOESM2_ESM.docx]

| **Supplemental Table 1.** Association between detection of PFAS based on UCMR3 data and county-level cancer incidence. | | | | |
| --- | --- | --- | --- | --- |
| **Cancers** | **Exposures** | **IRR [95% CI]** | **p value** | **adjusted p value^1,2,3^** |
| Respiratory System | PFOA | 1.08 [1.02, 1.14] | 0.00 | 0.06 |
| Endocrine System | PFNA | 1.29 [1.05, 1.57] | 0.01 | 0.18 |
| Oral Cavity and Pharynx | PFBS | 1.33 [1.04, 1.71] | 0.02 | 0.33 |
| Respiratory System | PFBS | 1.16 [1, 1.35] | 0.06 | 0.75 |
| Endocrine System | PFOS | 1.1 [1, 1.22] | 0.06 | 0.82 |
| Breast | PFBS | 1.12 [0.98, 1.27] | 0.10 | 1.00 |
| Endocrine System | PFOA | 1.08 [0.98, 1.18] | 0.11 | 1.00 |
| All Sites | PFOA | 1.02 [0.99, 1.05] | 0.11 | 1.00 |
| Respiratory System | PFHpA | 1.04 [0.98, 1.11] | 0.16 | 1.00 |
| Digestive System | PFBS | 0.92 [0.81, 1.04] | 0.16 | 1.00 |
| Endocrine System | PFHxS | 1.07 [0.96, 1.2] | 0.20 | 1.00 |
| Lymphoma | PFOS | 1.05 [0.97, 1.13] | 0.21 | 1.00 |
| Endocrine System | PFBS | 0.82 [0.6, 1.13] | 0.23 | 1.00 |
| All Sites | PFNA | 1.04 [0.97, 1.12] | 0.23 | 1.00 |
| Brain and Other Nervous System | PFHpA | 1.08 [0.94, 1.24] | 0.25 | 1.00 |
| Brain and Other Nervous System | PFOA | 1.07 [0.95, 1.21] | 0.28 | 1.00 |
| Breast | PFOA | 1.02 [0.98, 1.06] | 0.30 | 1.00 |
| Soft Tissue including Heart | PFBS | 1.3 [0.79, 2.17] | 0.30 | 1.00 |
| Skin excluding Basal and Squamous | PFOA | 0.95 [0.86, 1.05] | 0.32 | 1.00 |
| Lymphoma | PFHxS | 1.04 [0.96, 1.13] | 0.33 | 1.00 |
| All Sites | PFHpA | 1.02 [0.98, 1.05] | 0.35 | 1.00 |
| All Sites | PFOS | 1.02 [0.98, 1.05] | 0.35 | 1.00 |
| Bones and Joints | PFBS | 1.47 [0.64, 3.37] | 0.36 | 1.00 |
| Respiratory System | PFOS | 1.03 [0.97, 1.09] | 0.36 | 1.00 |
| Digestive System | PFNA | 1.04 [0.95, 1.15] | 0.37 | 1.00 |
| Respiratory System | PFHxS | 1.03 [0.96, 1.1] | 0.39 | 1.00 |
| Skin excluding Basal and Squamous | PFHpA | 0.95 [0.85, 1.07] | 0.41 | 1.00 |
| Endocrine System | PFHpA | 1.04 [0.94, 1.16] | 0.41 | 1.00 |
| All Sites | PFHxS | 1.01 [0.98, 1.05] | 0.42 | 1.00 |
| Myeloma | PFOS | 0.95 [0.83, 1.08] | 0.42 | 1.00 |
| Myeloma | PFOA | 0.95 [0.85, 1.07] | 0.42 | 1.00 |
| Respiratory System | PFNA | 1.05 [0.93, 1.2] | 0.43 | 1.00 |
| Oral Cavity and Pharynx | PFOA | 1.03 [0.94, 1.13] | 0.49 | 1.00 |
| Myeloma | PFHxS | 0.95 [0.82, 1.1] | 0.50 | 1.00 |
| Lymphoma | PFOA | 1.02 [0.96, 1.09] | 0.51 | 1.00 |
| Myeloma | PFHpA | 0.96 [0.84, 1.09] | 0.51 | 1.00 |
| Leukemia | PFHxS | 1.03 [0.93, 1.14] | 0.54 | 1.00 |
| Brain and Other Nervous System | PFOS | 1.04 [0.91, 1.2] | 0.54 | 1.00 |
| Soft Tissue including Heart | PFOA | 1.05 [0.89, 1.25] | 0.54 | 1.00 |
| Breast | PFNA | 1.03 [0.94, 1.13] | 0.54 | 1.00 |
| Skin excluding Basal and Squamous | PFNA | 0.93 [0.74, 1.18] | 0.56 | 1.00 |
| Leukemia | PFBS | 1.06 [0.83, 1.36] | 0.62 | 1.00 |
| Bones and Joints | PFHpA | 0.92 [0.64, 1.3] | 0.62 | 1.00 |
| Urinary System | PFHpA | 1.01 [0.96, 1.07] | 0.64 | 1.00 |
| Brain and Other Nervous System | PFNA | 1.07 [0.8, 1.43] | 0.65 | 1.00 |
| Digestive System | PFOA | 1.01 [0.97, 1.05] | 0.65 | 1.00 |
| Urinary System | PFNA | 1.03 [0.91, 1.16] | 0.67 | 1.00 |
| All Sites | PFBS | 1.02 [0.93, 1.12] | 0.68 | 1.00 |
| Leukemia | PFOS | 1.02 [0.93, 1.12] | 0.69 | 1.00 |
| Urinary System | PFOS | 1.01 [0.96, 1.07] | 0.69 | 1.00 |
| Digestive System | PFHpA | 1.01 [0.96, 1.05] | 0.70 | 1.00 |
| Skin excluding Basal and Squamous | PFOS | 0.98 [0.88, 1.09] | 0.71 | 1.00 |
| Skin excluding Basal and Squamous | PFBS | 1.06 [0.77, 1.46] | 0.71 | 1.00 |
| Leukemia | PFHpA | 1.02 [0.93, 1.11] | 0.72 | 1.00 |
| Lymphoma | PFBS | 0.96 [0.77, 1.2] | 0.72 | 1.00 |
| Myeloma | PFBS | 1.07 [0.72, 1.58] | 0.74 | 1.00 |
| Brain and Other Nervous System | PFHxS | 1.02 [0.88, 1.19] | 0.76 | 1.00 |
| Digestive System | PFOS | 0.99 [0.95, 1.04] | 0.76 | 1.00 |
| Lymphoma | PFHpA | 1.01 [0.94, 1.09] | 0.76 | 1.00 |
| Bones and Joints | PFHxS | 0.94 [0.64, 1.39] | 0.77 | 1.00 |
| Oral Cavity and Pharynx | PFNA | 0.97 [0.78, 1.21] | 0.77 | 1.00 |
| Bones and Joints | PFOA | 0.96 [0.7, 1.31] | 0.78 | 1.00 |
| Breast | PFHxS | 0.99 [0.95, 1.04] | 0.78 | 1.00 |
| Bones and Joints | PFNA | 0.9 [0.42, 1.94] | 0.79 | 1.00 |
| Soft Tissue including Heart | PFHpA | 0.98 [0.81, 1.18] | 0.81 | 1.00 |
| Urinary System | PFOA | 1.01 [0.96, 1.06] | 0.82 | 1.00 |
| Breast | PFHpA | 1.01 [0.96, 1.05] | 0.82 | 1.00 |
| Leukemia | PFOA | 0.99 [0.91, 1.08] | 0.84 | 1.00 |
| Urinary System | PFBS | 0.98 [0.84, 1.15] | 0.84 | 1.00 |
| Soft Tissue including Heart | PFNA | 0.96 [0.63, 1.46] | 0.85 | 1.00 |
| Breast | PFOS | 1 [0.95, 1.04] | 0.87 | 1.00 |
| Urinary System | PFHxS | 1 [0.94, 1.07] | 0.89 | 1.00 |
| Digestive System | PFHxS | 1 [0.96, 1.05] | 0.90 | 1.00 |
| Bones and Joints | PFOS | 0.98 [0.69, 1.39] | 0.90 | 1.00 |
| Leukemia | PFNA | 0.99 [0.81, 1.2] | 0.91 | 1.00 |
| Soft Tissue including Heart | PFOS | 1.01 [0.84, 1.22] | 0.91 | 1.00 |
| Oral Cavity and Pharynx | PFHxS | 1.01 [0.9, 1.12] | 0.92 | 1.00 |
| Soft Tissue including Heart | PFHxS | 0.99 [0.8, 1.22] | 0.93 | 1.00 |
| Skin excluding Basal and Squamous | PFHxS | 1.01 [0.89, 1.13] | 0.93 | 1.00 |
| Myeloma | PFNA | 0.99 [0.75, 1.3] | 0.95 | 1.00 |
| Oral Cavity and Pharynx | PFHpA | 1 [0.91, 1.11] | 0.95 | 1.00 |
| Brain and Other Nervous System | PFBS | 1.01 [0.69, 1.49] | 0.95 | 1.00 |
| Lymphoma | PFNA | 1 [0.85, 1.18] | 0.96 | 1.00 |
| Oral Cavity and Pharynx | PFOS | 1 [0.9, 1.11] | 0.99 | 1.00 |
| 1. All models were adjusted for county-level SES variables, urbanicity, smoking rate, obesity, and air pollution. | | | | |
| 2. p values were adjusted using false discovery rate method and were adjusted by PFAS chemical. | | | | |
| 3. cells were highlighted if crude or adjusted p values were less than 0.05. | | | | |

| **Supplemental Table 2.** Association between detection or violation of MCL of PFAS based on UCMR5 data and county-level cancer incidence. | | | | |
| --- | --- | --- | --- | --- |
| **Cancers** | **Exposures** | **IRR [95% CI]** | **p value** | **adjusted p value^1,2,3^** |
| Skin excluding Basal and Squamous | PFBA | 0.89 [0.84, 0.93] | 0.00 | 0.00 |
| Respiratory System | PFBA | 1.03 [1.01, 1.06] | 0.02 | 0.25 |
| Endocrine System | PFHpA | 1.09 [1.01, 1.18] | 0.02 | 0.33 |
| Digestive System | PFBA | 1.02 [1, 1.05] | 0.03 | 0.32 |
| Leukemia | PFBA | 0.96 [0.91, 1] | 0.05 | 0.54 |
| Endocrine System | PFBA | 0.96 [0.91, 1.01] | 0.09 | 0.88 |
| Breast | PFBA | 0.98 [0.96, 1] | 0.09 | 0.88 |
| Urinary System | PFBA | 1.02 [1, 1.05] | 0.09 | 0.88 |
| Urinary System | PFPEA | 0.97 [0.95, 1] | 0.09 | 1.00 |
| Skin excluding Basal and Squamous | PFHpA | 0.93 [0.86, 1.01] | 0.10 | 1.00 |
| Respiratory System | PFPEA | 1.03 [1, 1.06] | 0.10 | 1.00 |
| Oral Cavity and Pharynx | PFBS | 1.05 [0.99, 1.1] | 0.10 | 1.00 |
| Urinary System | PFHXA | 0.97 [0.94, 1.01] | 0.11 | 1.00 |
| Oral Cavity and Pharynx | PFBA | 1.04 [0.99, 1.09] | 0.13 | 0.88 |
| Endocrine System | PFPEA | 0.96 [0.91, 1.02] | 0.16 | 1.00 |
| Digestive System | PFBS | 1.02 [0.99, 1.04] | 0.17 | 1.00 |
| Respiratory System | PFHpA | 1.03 [0.99, 1.07] | 0.18 | 1.00 |
| Oral Cavity and Pharynx | PFPEA | 1.04 [0.98, 1.09] | 0.19 | 1.00 |
| Endocrine System | PFHXA | 0.96 [0.9, 1.02] | 0.20 | 1.00 |
| Brain and Other Nervous System | PFHpA | 1.07 [0.96, 1.18] | 0.21 | 1.00 |
| Endocrine System | PFBS | 0.97 [0.91, 1.02] | 0.24 | 1.00 |
| Respiratory System | PFHXA | 1.02 [0.99, 1.05] | 0.24 | 1.00 |
| Respiratory System | PFBS | 1.02 [0.99, 1.05] | 0.30 | 1.00 |
| Breast | PFHXA | 0.99 [0.96, 1.01] | 0.30 | 1.00 |
| Urinary System | PFBS | 0.98 [0.96, 1.01] | 0.32 | 1.00 |
| Lymphoma | PFBS | 0.98 [0.94, 1.02] | 0.34 | 1.00 |
| Lymphoma | PFHpA | 1.03 [0.97, 1.09] | 0.35 | 1.00 |
| Soft Tissue including Heart | PFPEA | 1.04 [0.94, 1.16] | 0.42 | 1.00 |
| Bones and Joints | PFBA | 0.93 [0.78, 1.11] | 0.44 | 1.00 |
| Leukemia | PFPEA | 0.98 [0.93, 1.03] | 0.45 | 1.00 |
| Digestive System | PFPEA | 1.01 [0.98, 1.03] | 0.49 | 1.00 |
| Soft Tissue including Heart | PFBA | 0.97 [0.88, 1.07] | 0.53 | 1.00 |
| Leukemia | PFBS | 0.98 [0.94, 1.03] | 0.54 | 1.00 |
| All Sites | PFHpA | 1.01 [0.98, 1.03] | 0.55 | 1.00 |
| Digestive System | PFHXA | 1.01 [0.98, 1.03] | 0.56 | 1.00 |
| Digestive System | PFHpA | 1.01 [0.98, 1.04] | 0.57 | 1.00 |
| Oral Cavity and Pharynx | PFHXA | 1.02 [0.96, 1.07] | 0.58 | 1.00 |
| Myeloma | PFHXA | 1.02 [0.95, 1.1] | 0.64 | 1.00 |
| Myeloma | PFBS | 1.02 [0.95, 1.09] | 0.65 | 1.00 |
| Myeloma | PFBA | 1.01 [0.95, 1.08] | 0.66 | 1.00 |
| Breast | PFHpA | 0.99 [0.96, 1.03] | 0.67 | 1.00 |
| All Sites | PFHXA | 1 [0.98, 1.01] | 0.67 | 1.00 |
| Brain and Other Nervous System | PFBA | 0.99 [0.92, 1.05] | 0.69 | 1.00 |
| All Sites | PFBA | 1 [0.98, 1.01] | 0.690 | 1.00 |
| Brain and Other Nervous System | PFHXA | 0.98 [0.91, 1.06] | 0.70 | 1.00 |
| Brain and Other Nervous System | PFBS | 1.01 [0.94, 1.09] | 0.73 | 1.00 |
| Lymphoma | PFPEA | 1.01 [0.97, 1.05] | 0.74 | 1.00 |
| Bones and Joints | PFPEA | 0.97 [0.8, 1.17] | 0.74 | 1.00 |
| Leukemia | PFHXA | 0.99 [0.94, 1.04] | 0.75 | 1.00 |
| Myeloma | PFHpA | 0.98 [0.89, 1.09] | 0.76 | 1.00 |
| Urinary System | PFHpA | 1.01 [0.97, 1.05] | 0.76 | 1.00 |
| Oral Cavity and Pharynx | PFHpA | 1.01 [0.94, 1.09] | 0.77 | 1.00 |
| Leukemia | PFHpA | 1.01 [0.94, 1.08] | 0.77 | 1.00 |
| Myeloma | PFPEA | 0.99 [0.92, 1.06] | 0.79 | 1.00 |
| Soft Tissue including Heart | PFHXA | 1.01 [0.91, 1.13] | 0.80 | 1.00 |
| Brain and Other Nervous System | PFPEA | 1.01 [0.94, 1.09] | 0.81 | 1.00 |
| Lymphoma | PFHXA | 0.99 [0.95, 1.04] | 0.81 | 1.00 |
| Skin excluding Basal and Squamous | PFPEA | 0.99 [0.94, 1.05] | 0.82 | 1.00 |
| Lymphoma | PFBA | 1 [0.96, 1.03] | 0.84 | 1.00 |
| Bones and Joints | PFHXA | 0.98 [0.81, 1.2] | 0.85 | 1.00 |
| Breast | PFBS | 1 [0.97, 1.02] | 0.86 | 1.00 |
| All Sites | PFPEA | 1 [0.98, 1.02] | 0.87 | 1.00 |
| Bones and Joints | PFBS | 0.99 [0.81, 1.19] | 0.88 | 1.00 |
| Skin excluding Basal and Squamous | PFHXA | 1 [0.94, 1.06] | 0.88 | 1.00 |
| Breast | PFPEA | 1 [0.97, 1.02] | 0.90 | 1.00 |
| Soft Tissue including Heart | PFBS | 1.01 [0.91, 1.12] | 0.90 | 1.00 |
| Bones and Joints | PFHpA | 1.01 [0.78, 1.31] | 0.91 | 1.00 |
| Skin excluding Basal and Squamous | PFBS | 1 [0.95, 1.06] | 0.93 | 1.00 |
| Soft Tissue including Heart | PFHpA | 1 [0.87, 1.16] | 0.97 | 1.00 |
| All Sites | PFBS | 1 [0.98, 1.02] | 0.98 | 1.00 |
| 1. All models were adjusted for county-level SES variables, urbanicity, smoking rate, obesity, and air pollution. | | | | |
| 2. p values were adjusted using false discovery rate method and were adjusted by PFAS chemical. | | | | |
| 3. cells were highlighted if crude or adjusted p values were less than 0.05. | | | | |

| **Supplemental Table 3.** Association between PFAS based on UCMR5 data and county-level cancer incidence. | | | | |
| --- | --- | --- | --- | --- |
| **Cancers** | **Exposures** | **IRR [95% CI]** | **p value** | **adjusted p value^1,2,3^** |
| Digestive System | PFHxS | 1.11 [1.04, 1.19] | 0.00 | 0.02 |
| Skin excluding Basal and Squamous | PFHxS | 1.17 [0.99, 1.39] | 0.06 | 0.83 |
| All Sites | PFHxS | 1.03 [0.98, 1.08] | 0.27 | 1.00 |
| Oral Cavity and Pharynx | PFHxS | 1.13 [0.98, 1.32] | 0.10 | 1.00 |
| Respiratory System | PFHxS | 1.04 [0.95, 1.13] | 0.42 | 1.00 |
| Bones and Joints | PFHxS | 1.25 [0.76, 2.04] | 0.39 | 1.00 |
| Soft Tissue including Heart | PFHxS | 1.09 [0.8, 1.47] | 0.59 | 1.00 |
| Breast | PFHxS | 0.99 [0.92, 1.06] | 0.72 | 1.00 |
| Urinary System | PFHxS | 0.96 [0.87, 1.05] | 0.35 | 1.00 |
| Brain and Other Nervous System | PFHxS | 0.95 [0.75, 1.19] | 0.63 | 1.00 |
| Endocrine System | PFHxS | 1.04 [0.87, 1.23] | 0.69 | 1.00 |
| Lymphoma | PFHxS | 1 [0.89, 1.14] | 0.96 | 1.00 |
| Myeloma | PFHxS | 1.02 [0.84, 1.25] | 0.83 | 1.00 |
| Leukemia | PFHxS | 0.96 [0.83, 1.12] | 0.61 | 1.00 |
| All Sites | PFOA | 1.01 [0.99, 1.04] | 0.28 | 1.00 |
| Oral Cavity and Pharynx | PFOA | 0.99 [0.91, 1.08] | 0.84 | 1.00 |
| Digestive System | PFOA | 1.02 [0.99, 1.06] | 0.21 | 1.00 |
| Respiratory System | PFOA | 1.03 [0.98, 1.08] | 0.28 | 1.00 |
| Bones and Joints | PFOA | 1.01 [0.75, 1.36] | 0.95 | 1.00 |
| Soft Tissue including Heart | PFOA | 1.02 [0.87, 1.2] | 0.83 | 1.00 |
| Skin excluding Basal and Squamous | PFOA | 0.97 [0.89, 1.06] | 0.51 | 1.00 |
| Breast | PFOA | 1.02 [0.99, 1.06] | 0.19 | 1.00 |
| Urinary System | PFOA | 0.98 [0.93, 1.03] | 0.37 | 1.00 |
| Brain and Other Nervous System | PFOA | 1.04 [0.92, 1.16] | 0.56 | 1.00 |
| Endocrine System | PFOA | 1.06 [0.97, 1.15] | 0.23 | 1.00 |
| Lymphoma | PFOA | 1.02 [0.95, 1.08] | 0.61 | 1.00 |
| Myeloma | PFOA | 1.02 [0.91, 1.13] | 0.77 | 1.00 |
| Leukemia | PFOA | 1.01 [0.93, 1.09] | 0.88 | 1.00 |
| All Sites | PFOS | 1 [0.97, 1.03] | 0.89 | 1.00 |
| Oral Cavity and Pharynx | PFOS | 1.05 [0.96, 1.14] | 0.30 | 1.00 |
| Digestive System | PFOS | 1 [0.96, 1.04] | 0.93 | 1.00 |
| Respiratory System | PFOS | 1.02 [0.97, 1.07] | 0.43 | 1.00 |
| Bones and Joints | PFOS | 0.98 [0.72, 1.34] | 0.91 | 1.00 |
| Soft Tissue including Heart | PFOS | 0.99 [0.84, 1.18] | 0.94 | 1.00 |
| Skin excluding Basal and Squamous | PFOS | 1.09 [0.99, 1.19] | 0.08 | 1.00 |
| Breast | PFOS | 1.01 [0.97, 1.05] | 0.52 | 1.00 |
| Urinary System | PFOS | 0.99 [0.94, 1.04] | 0.69 | 1.00 |
| Brain and Other Nervous System | PFOS | 0.98 [0.87, 1.11] | 0.76 | 1.00 |
| Endocrine System | PFOS | 0.95 [0.86, 1.04] | 0.26 | 1.00 |
| Lymphoma | PFOS | 0.99 [0.93, 1.06] | 0.86 | 1.00 |
| Myeloma | PFOS | 0.96 [0.85, 1.07] | 0.45 | 1.00 |
| Leukemia | PFOS | 1.01 [0.93, 1.09] | 0.81 | 1.00 |
| 1. All models were adjusted for county-level SES variables, urbanicity, smoking rate, obesity, and air pollution. | | | | |
| 2. p values were adjusted using false discovery rate method and were adjusted by PFAS chemical. | | | | |
| 3. cells were highlighted if crude or adjusted p values were less than 0.05. | | | | |

| **Supplemental Table 4.** Association between detection of PFAS based on UCMR3 data and county-level cancer incidence for each sub-type. | | | |
| --- | --- | --- | --- |
| **Cancers** | **Exposures** | **IRR [95% CI]** | **p value^1,2^** |
| **All Sites** | PFOA | 1.02 [0.99, 1.05] | 0.11 |
| Oral Cavity and Pharynx | PFOA | 1.03 [0.94, 1.13] | 0.49 |
| Lip | PFOA | 1.07 [0.69, 1.65] | 0.76 |
| Tongue | PFOA | 1.02 [0.87, 1.19] | 0.83 |
| Salivary Gland | PFOA | 1.07 [0.82, 1.39] | 0.63 |
| Floor of Mouth | PFOA | 0.98 [0.61, 1.58] | 0.94 |
| Gum and Other Mouth | PFOA | 1.04 [0.81, 1.32] | 0.77 |
| Nasopharynx | PFOA | 1 [0.65, 1.55] | 0.99 |
| Tonsil | PFOA | 1.05 [0.86, 1.29] | 0.61 |
| Oropharynx | PFOA | 0.96 [0.66, 1.39] | 0.82 |
| Hypopharynx | PFOA | 1.07 [0.7, 1.65] | 0.74 |
| Other Oral Cavity and Pharynx | PFOA | 0.9 [0.46, 1.76] | 0.75 |
| **Digestive System** | PFOA | 1.01 [0.97, 1.05] | 0.65 |
| Esophagus | PFOA | 1.08 [0.93, 1.24] | 0.32 |
| Stomach | PFOA | 1.13 [1.01, 1.28] | 0.04 |
| Small Intestine | PFOA | 0.99 [0.82, 1.2] | 0.94 |
| Colon and Rectum | PFOA | 1 [0.95, 1.06] | 0.89 |
| Colon excluding Rectum | PFOA | 1.01 [0.95, 1.07] | 0.81 |
| Cecum | PFOA | 0.96 [0.83, 1.1] | 0.52 |
| Appendix | PFOA | 0.99 [0.79, 1.25] | 0.96 |
| Ascending Colon | PFOA | 1.06 [0.92, 1.22] | 0.40 |
| Hepatic Flexure | PFOA | 1.07 [0.8, 1.43] | 0.63 |
| Transverse Colon | PFOA | 1.07 [0.88, 1.3] | 0.51 |
| Splenic Flexure | PFOA | 1.04 [0.72, 1.5] | 0.83 |
| Descending Colon | PFOA | 0.95 [0.73, 1.23] | 0.68 |
| Sigmoid Colon | PFOA | 1 [0.88, 1.13] | 0.99 |
| Large Intestine NOS | PFOA | 0.96 [0.77, 1.2] | 0.73 |
| Rectum and Rectosigmoid Junction | PFOA | 0.99 [0.91, 1.09] | 0.90 |
| Rectosigmoid Junction | PFOA | 0.98 [0.81, 1.19] | 0.85 |
| Rectum | PFOA | 1 [0.9, 1.11] | 0.95 |
| Anus Anal Canal and Anorectum | PFOA | 0.99 [0.79, 1.23] | 0.91 |
| Liver and Intrahepatic Bile Duct | PFOA | 0.91 [0.82, 1.02] | 0.10 |
| Liver | PFOA | 0.89 [0.78, 1] | 0.06 |
| Intrahepatic Bile Duct | PFOA | 1.04 [0.82, 1.32] | 0.76 |
| Gallbladder | PFOA | 0.95 [0.7, 1.28] | 0.71 |
| Other Biliary | PFOA | 1.05 [0.83, 1.33] | 0.68 |
| Pancreas | PFOA | 1.02 [0.94, 1.11] | 0.63 |
| Retroperitoneum | PFOA | 0.96 [0.59, 1.56] | 0.86 |
| Peritoneum Omentum and Mesentery | PFOA | 0.93 [0.58, 1.49] | 0.76 |
| Other Digestive Organs | PFOA | 1.02 [0.72, 1.45] | 0.90 |
| **Respiratory System** | PFOA | 1.08 [1.02, 1.14] | 0.00 |
| Nose Nasal Cavity and Middle Ear | PFOA | 1.03 [0.7, 1.51] | 0.89 |
| Larynx | PFOA | 1.1 [0.92, 1.32] | 0.29 |
| Lung and Bronchus | PFOA | 1.08 [1.02, 1.14] | 0.01 |
| Pleura | PFOA | 1.38 [0.2, 9.37] | 0.74 |
| Trachea Mediastinum and Other Respiratory Organs | PFOA | 1.45 [0.72, 2.89] | 0.30 |
| Bones and Joints | PFOA | 0.96 [0.7, 1.31] | 0.78 |
| Soft Tissue including Heart | PFOA | 1.05 [0.89, 1.25] | 0.54 |
| Skin excluding Basal and Squamous | PFOA | 0.95 [0.86, 1.05] | 0.32 |
| Melanoma of the Skin | PFOA | 0.95 [0.86, 1.05] | 0.33 |
| Other Non Epithelial Skin | PFOA | 0.97 [0.77, 1.22] | 0.76 |
| **Breast** | PFOA | 1.02 [0.98, 1.06] | 0.30 |
| **Urinary System** | PFOA | 1.01 [0.96, 1.06] | 0.82 |
| Urinary Bladder | PFOA | 1.06 [0.99, 1.14] | 0.10 |
| Kidney and Renal Pelvis | PFOA | 0.94 [0.87, 1.01] | 0.10 |
| Ureter | PFOA | 1.19 [0.79, 1.79] | 0.41 |
| Other Urinary Organs | PFOA | 0.96 [0.59, 1.58] | 0.89 |
| **Brain and Other Nervous System** | PFOA | 1.07 [0.95, 1.21] | 0.28 |
| Brain | PFOA | 1.08 [0.95, 1.22] | 0.22 |
| Cranial Nerves Other Nervous System | PFOA | 0.88 [0.52, 1.48] | 0.62 |
| **Endocrine System** | PFOA | 1.08 [0.98, 1.18] | 0.11 |
| Thyroid | PFOA | 1.07 [0.97, 1.17] | 0.17 |
| Other Endocrine including Thymus | PFOA | 1.19 [0.86, 1.64] | 0.29 |
| **Lymphoma** | PFOA | 1.02 [0.96, 1.09] | 0.51 |
| **Hodgkin Lymphoma** | PFOA | 1.05 [0.86, 1.27] | 0.65 |
| Hodgkin Nodal | PFOA | 1.05 [0.87, 1.28] | 0.60 |
| Hodgkin Extranodal | PFOA | 0.68 [0.12, 3.96] | 0.67 |
| Non Hodgkin Lymphoma | PFOA | 1.02 [0.95, 1.1] | 0.59 |
| NHL Nodal | PFOA | 1 [0.92, 1.09] | 0.96 |
| NHL Extranodal | PFOA | 1.06 [0.93, 1.19] | 0.39 |
| **Myeloma** | PFOA | 0.95 [0.85, 1.07] | 0.42 |
| **Leukemia** | PFOA | 0.99 [0.91, 1.08] | 0.84 |
| Lymphocytic Leukemia | PFOA | 0.97 [0.87, 1.09] | 0.64 |
| Acute Lymphocytic Leukemia | PFOA | 0.95 [0.75, 1.21] | 0.70 |
| Chronic Lymphocytic Leukemia | PFOA | 0.96 [0.84, 1.11] | 0.61 |
| Other Lymphocytic Leukemia | PFOA | 1.2 [0.75, 1.92] | 0.45 |
| Myeloid and Monocytic Leukemia | PFOA | 1.03 [0.91, 1.16] | 0.61 |
| Acute Myeloid Leukemia | PFOA | 1.01 [0.87, 1.18] | 0.88 |
| Acute Monocytic Leukemia | PFOA | 1.37 [0.68, 2.73] | 0.38 |
| Chronic Myeloid Leukemia | PFOA | 1.02 [0.82, 1.27] | 0.83 |
| Other Myeloid Monocytic Leukemia | PFOA | 1.52 [0.69, 3.35] | 0.30 |
| Other Leukemia | PFOA | 0.83 [0.58, 1.19] | 0.31 |
| Other Acute Leukemia | PFOA | 0.73 [0.36, 1.47] | 0.38 |
| Aleukemic Subleukemic and NOS | PFOA | 0.87 [0.57, 1.32] | 0.51 |
| **All Sites** | PFOS | 1.02 [0.98, 1.05] | 0.35 |
| Oral Cavity and Pharynx | PFOS | 1 [0.9, 1.11] | 0.99 |
| Lip | PFOS | 1.06 [0.64, 1.76] | 0.82 |
| Tongue | PFOS | 0.97 [0.81, 1.16] | 0.74 |
| Salivary Gland | PFOS | 1.08 [0.8, 1.45] | 0.61 |
| Floor of Mouth | PFOS | 0.98 [0.57, 1.69] | 0.96 |
| Gum and Other Mouth | PFOS | 1.02 [0.78, 1.34] | 0.87 |
| Nasopharynx | PFOS | 0.83 [0.5, 1.37] | 0.46 |
| Tonsil | PFOS | 1 [0.79, 1.26] | 0.98 |
| Oropharynx | PFOS | 0.95 [0.62, 1.44] | 0.80 |
| Hypopharynx | PFOS | 1.03 [0.64, 1.66] | 0.91 |
| Other Oral Cavity and Pharynx | PFOS | 1.12 [0.56, 2.25] | 0.75 |
| **Digestive System** | PFOS | 0.99 [0.95, 1.04] | 0.76 |
| Esophagus | PFOS | 1.01 [0.86, 1.2] | 0.87 |
| Stomach | PFOS | 1.05 [0.92, 1.19] | 0.51 |
| Small Intestine | PFOS | 0.98 [0.79, 1.22] | 0.88 |
| Colon and Rectum | PFOS | 1 [0.95, 1.07] | 0.91 |
| Colon excluding Rectum | PFOS | 0.99 [0.93, 1.06] | 0.87 |
| Cecum | PFOS | 0.95 [0.81, 1.11] | 0.51 |
| Appendix | PFOS | 1.04 [0.81, 1.34] | 0.75 |
| Ascending Colon | PFOS | 1.03 [0.88, 1.2] | 0.75 |
| Hepatic Flexure | PFOS | 1.01 [0.73, 1.4] | 0.96 |
| Transverse Colon | PFOS | 1.09 [0.87, 1.35] | 0.45 |
| Splenic Flexure | PFOS | 1.01 [0.67, 1.52] | 0.96 |
| Descending Colon | PFOS | 0.94 [0.7, 1.25] | 0.65 |
| Sigmoid Colon | PFOS | 0.99 [0.86, 1.13] | 0.84 |
| Large Intestine NOS | PFOS | 0.94 [0.73, 1.21] | 0.62 |
| Rectum and Rectosigmoid Junction | PFOS | 1.02 [0.92, 1.13] | 0.67 |
| Rectosigmoid Junction | PFOS | 1.06 [0.86, 1.32] | 0.57 |
| Rectum | PFOS | 1.01 [0.9, 1.14] | 0.86 |
| Anus Anal Canal and Anorectum | PFOS | 0.95 [0.74, 1.23] | 0.71 |
| Liver and Intrahepatic Bile Duct | PFOS | 0.87 [0.77, 0.98] | 0.02 |
| Liver | PFOS | 0.86 [0.74, 0.98] | 0.03 |
| Intrahepatic Bile Duct | PFOS | 0.93 [0.71, 1.23] | 0.63 |
| Gallbladder | PFOS | 0.95 [0.69, 1.33] | 0.78 |
| Other Biliary | PFOS | 1.11 [0.87, 1.44] | 0.40 |
| Pancreas | PFOS | 1.02 [0.92, 1.12] | 0.75 |
| Retroperitoneum | PFOS | 0.98 [0.57, 1.68] | 0.94 |
| Peritoneum Omentum and Mesentery | PFOS | 0.95 [0.56, 1.62] | 0.85 |
| Other Digestive Organs | PFOS | 1.05 [0.72, 1.54] | 0.80 |
| **Respiratory System** | PFOS | 1.03 [0.97, 1.09] | 0.36 |
| Nose Nasal Cavity and Middle Ear | PFOS | 0.99 [0.64, 1.53] | 0.98 |
| Larynx | PFOS | 1.05 [0.86, 1.29] | 0.63 |
| Lung and Bronchus | PFOS | 1.03 [0.97, 1.09] | 0.40 |
| Pleura | PFOS | 1.24 [0.15, 10.68] | 0.84 |
| Trachea Mediastinum and Other Respiratory Organs | PFOS | 1.35 [0.62, 2.92] | 0.45 |
| Bones and Joints | PFOS | 0.98 [0.69, 1.39] | 0.90 |
| Soft Tissue including Heart | PFOS | 1.01 [0.84, 1.22] | 0.91 |
| Skin excluding Basal and Squamous | PFOS | 0.98 [0.88, 1.09] | 0.71 |
| Melanoma of the Skin | PFOS | 0.98 [0.88, 1.1] | 0.75 |
| Other Non Epithelial Skin | PFOS | 0.96 [0.74, 1.24] | 0.74 |
| **Breast** | PFOS | 1 [0.95, 1.04] | 0.87 |
| **Urinary System** | PFOS | 1.01 [0.96, 1.07] | 0.69 |
| Urinary Bladder | PFOS | 1.06 [0.98, 1.15] | 0.12 |
| Kidney and Renal Pelvis | PFOS | 0.96 [0.88, 1.04] | 0.34 |
| Ureter | PFOS | 1 [0.61, 1.63] | 1.00 |
| Other Urinary Organs | PFOS | 0.89 [0.5, 1.58] | 0.68 |
| **Brain and Other Nervous System** | PFOS | 1.04 [0.91, 1.2] | 0.54 |
| Brain | PFOS | 1.04 [0.9, 1.19] | 0.63 |
| Cranial Nerves Other Nervous System | PFOS | 1.16 [0.69, 1.98] | 0.57 |
| **Endocrine System** | PFOS | 1.1 [1, 1.22] | 0.06 |
| Thyroid | PFOS | 1.1 [0.99, 1.22] | 0.08 |
| Other Endocrine including Thymus | PFOS | 1.16 [0.82, 1.65] | 0.40 |
| **Lymphoma** | PFOS | 1.05 [0.97, 1.13] | 0.21 |
| **Hodgkin Lymphoma** | PFOS | 1.12 [0.91, 1.38] | 0.29 |
| Hodgkin Nodal | PFOS | 1.13 [0.91, 1.39] | 0.27 |
| Hodgkin Extranodal | PFOS | 0.83 [0.13, 5.31] | 0.85 |
| Non Hodgkin Lymphoma | PFOS | 1.04 [0.96, 1.13] | 0.34 |
| NHL Nodal | PFOS | 1.02 [0.93, 1.13] | 0.64 |
| NHL Extranodal | PFOS | 1.07 [0.93, 1.23] | 0.33 |
| **Myeloma** | PFOS | 0.95 [0.83, 1.08] | 0.42 |
| **Leukemia** | PFOS | 1.02 [0.93, 1.12] | 0.69 |
| Lymphocytic Leukemia | PFOS | 1.02 [0.89, 1.16] | 0.79 |
| Acute Lymphocytic Leukemia | PFOS | 0.89 [0.68, 1.17] | 0.41 |
| Chronic Lymphocytic Leukemia | PFOS | 1.05 [0.9, 1.22] | 0.54 |
| Other Lymphocytic Leukemia | PFOS | 1.24 [0.73, 2.09] | 0.42 |
| Myeloid and Monocytic Leukemia | PFOS | 1.03 [0.9, 1.18] | 0.62 |
| Acute Myeloid Leukemia | PFOS | 1.01 [0.85, 1.2] | 0.92 |
| Acute Monocytic Leukemia | PFOS | 1.41 [0.67, 2.95] | 0.37 |
| Chronic Myeloid Leukemia | PFOS | 1.04 [0.82, 1.33] | 0.75 |
| Other Myeloid Monocytic Leukemia | PFOS | 1.35 [0.54, 3.41] | 0.52 |
| Other Leukemia | PFOS | 0.9 [0.61, 1.34] | 0.61 |
| Other Acute Leukemia | PFOS | 0.79 [0.37, 1.7] | 0.55 |
| Aleukemic Subleukemic and NOS | PFOS | 0.94 [0.59, 1.49] | 0.80 |
| **All Sites** | PFHpA | 1.02 [0.98, 1.05] | 0.35 |
| Oral Cavity and Pharynx | PFHpA | 1 [0.91, 1.11] | 0.95 |
| Lip | PFHpA | 1.08 [0.65, 1.79] | 0.77 |
| Tongue | PFHpA | 0.93 [0.78, 1.12] | 0.45 |
| Salivary Gland | PFHpA | 1.11 [0.82, 1.49] | 0.50 |
| Floor of Mouth | PFHpA | 1 [0.58, 1.7] | 0.99 |
| Gum and Other Mouth | PFHpA | 1.08 [0.83, 1.42] | 0.57 |
| Nasopharynx | PFHpA | 1.05 [0.65, 1.68] | 0.85 |
| Tonsil | PFHpA | 1 [0.79, 1.26] | 0.98 |
| Oropharynx | PFHpA | 0.99 [0.65, 1.5] | 0.96 |
| Hypopharynx | PFHpA | 0.93 [0.57, 1.53] | 0.78 |
| Other Oral Cavity and Pharynx | PFHpA | 0.98 [0.47, 2.08] | 0.97 |
| **Digestive System** | PFHpA | 1.01 [0.96, 1.05] | 0.70 |
| Esophagus | PFHpA | 1.06 [0.9, 1.25] | 0.46 |
| Stomach | PFHpA | 1.05 [0.92, 1.2] | 0.45 |
| Small Intestine | PFHpA | 0.96 [0.77, 1.19] | 0.68 |
| Colon and Rectum | PFHpA | 1.02 [0.96, 1.09] | 0.45 |
| Colon excluding Rectum | PFHpA | 1.03 [0.96, 1.1] | 0.45 |
| Cecum | PFHpA | 0.98 [0.84, 1.15] | 0.81 |
| Appendix | PFHpA | 0.95 [0.73, 1.24] | 0.72 |
| Ascending Colon | PFHpA | 1.07 [0.91, 1.25] | 0.41 |
| Hepatic Flexure | PFHpA | 1.01 [0.72, 1.43] | 0.94 |
| Transverse Colon | PFHpA | 1.17 [0.94, 1.46] | 0.15 |
| Splenic Flexure | PFHpA | 0.96 [0.63, 1.47] | 0.86 |
| Descending Colon | PFHpA | 1.01 [0.76, 1.34] | 0.96 |
| Sigmoid Colon | PFHpA | 1.01 [0.88, 1.16] | 0.92 |
| Large Intestine NOS | PFHpA | 1.05 [0.82, 1.33] | 0.72 |
| Rectum and Rectosigmoid Junction | PFHpA | 1.02 [0.91, 1.13] | 0.78 |
| Rectosigmoid Junction | PFHpA | 1.08 [0.87, 1.34] | 0.47 |
| Rectum | PFHpA | 1 [0.88, 1.12] | 0.94 |
| Anus Anal Canal and Anorectum | PFHpA | 0.96 [0.74, 1.24] | 0.74 |
| Liver and Intrahepatic Bile Duct | PFHpA | 0.92 [0.81, 1.04] | 0.16 |
| Liver | PFHpA | 0.89 [0.78, 1.03] | 0.11 |
| Intrahepatic Bile Duct | PFHpA | 1.03 [0.79, 1.35] | 0.82 |
| Gallbladder | PFHpA | 0.97 [0.7, 1.34] | 0.86 |
| Other Biliary | PFHpA | 1.03 [0.79, 1.34] | 0.83 |
| Pancreas | PFHpA | 1.01 [0.92, 1.11] | 0.77 |
| Retroperitoneum | PFHpA | 0.9 [0.52, 1.57] | 0.71 |
| Peritoneum Omentum and Mesentery | PFHpA | 0.96 [0.57, 1.64] | 0.89 |
| Other Digestive Organs | PFHpA | 0.99 [0.67, 1.46] | 0.95 |
| **Respiratory System** | PFHpA | 1.04 [0.98, 1.11] | 0.16 |
| Nose Nasal Cavity and Middle Ear | PFHpA | 1.06 [0.69, 1.62] | 0.80 |
| Larynx | PFHpA | 1.01 [0.82, 1.24] | 0.94 |
| Lung and Bronchus | PFHpA | 1.04 [0.98, 1.11] | 0.16 |
| Pleura | PFHpA | 2.01 [0.3, 13.21] | 0.47 |
| Trachea Mediastinum and Other Respiratory Organs | PFHpA | 1.19 [0.52, 2.72] | 0.67 |
| Bones and Joints | PFHpA | 0.92 [0.64, 1.3] | 0.62 |
| Soft Tissue including Heart | PFHpA | 0.98 [0.81, 1.18] | 0.81 |
| Skin excluding Basal and Squamous | PFHpA | 0.95 [0.85, 1.07] | 0.41 |
| Melanoma of the Skin | PFHpA | 0.95 [0.85, 1.07] | 0.42 |
| Other Non Epithelial Skin | PFHpA | 0.97 [0.74, 1.25] | 0.79 |
| **Breast** | PFHpA | 1.01 [0.96, 1.05] | 0.82 |
| **Urinary System** | PFHpA | 1.01 [0.96, 1.07] | 0.64 |
| Urinary Bladder | PFHpA | 1.07 [0.99, 1.15] | 0.11 |
| Kidney and Renal Pelvis | PFHpA | 0.95 [0.87, 1.04] | 0.26 |
| Ureter | PFHpA | 1.18 [0.74, 1.88] | 0.49 |
| Other Urinary Organs | PFHpA | 0.92 [0.53, 1.61] | 0.78 |
| **Brain and Other Nervous System** | PFHpA | 1.08 [0.94, 1.24] | 0.25 |
| Brain | PFHpA | 1.08 [0.94, 1.24] | 0.29 |
| Cranial Nerves Other Nervous System | PFHpA | 1.16 [0.67, 2] | 0.59 |
| **Endocrine System** | PFHpA | 1.04 [0.94, 1.16] | 0.41 |
| Thyroid | PFHpA | 1.03 [0.93, 1.15] | 0.58 |
| Other Endocrine including Thymus | PFHpA | 1.23 [0.87, 1.74] | 0.25 |
| **Lymphoma** | PFHpA | 1.01 [0.94, 1.09] | 0.76 |
| **Hodgkin Lymphoma** | PFHpA | 1.01 [0.81, 1.26] | 0.90 |
| Hodgkin Nodal | PFHpA | 1.02 [0.82, 1.27] | 0.88 |
| Hodgkin Extranodal | PFHpA | 0.78 [0.13, 4.72] | 0.79 |
| Non Hodgkin Lymphoma | PFHpA | 1.01 [0.93, 1.1] | 0.77 |
| NHL Nodal | PFHpA | 0.99 [0.9, 1.09] | 0.85 |
| NHL Extranodal | PFHpA | 1.05 [0.92, 1.21] | 0.46 |
| **Myeloma** | PFHpA | 0.96 [0.84, 1.09] | 0.51 |
| **Leukemia** | PFHpA | 1.02 [0.93, 1.11] | 0.72 |
| Lymphocytic Leukemia | PFHpA | 1 [0.87, 1.14] | 0.95 |
| Acute Lymphocytic Leukemia | PFHpA | 0.94 [0.72, 1.23] | 0.66 |
| Chronic Lymphocytic Leukemia | PFHpA | 1.01 [0.87, 1.18] | 0.87 |
| Other Lymphocytic Leukemia | PFHpA | 1.04 [0.6, 1.8] | 0.90 |
| Myeloid and Monocytic Leukemia | PFHpA | 1.04 [0.9, 1.19] | 0.61 |
| Acute Myeloid Leukemia | PFHpA | 1.03 [0.87, 1.22] | 0.72 |
| Acute Monocytic Leukemia | PFHpA | 1.35 [0.61, 2.98] | 0.46 |
| Chronic Myeloid Leukemia | PFHpA | 1.02 [0.8, 1.3] | 0.89 |
| Other Myeloid Monocytic Leukemia | PFHpA | 1.16 [0.44, 3.04] | 0.76 |
| Other Leukemia | PFHpA | 1.03 [0.7, 1.51] | 0.88 |
| Other Acute Leukemia | PFHpA | 1.01 [0.49, 2.1] | 0.98 |
| Aleukemic Subleukemic and NOS | PFHpA | 1.03 [0.66, 1.62] | 0.90 |
| **All Sites** | PFHxS | 1.01 [0.98, 1.05] | 0.42 |
| Oral Cavity and Pharynx | PFHxS | 1.01 [0.9, 1.12] | 0.92 |
| Lip | PFHxS | 1.19 [0.69, 2.02] | 0.53 |
| Tongue | PFHxS | 0.95 [0.78, 1.15] | 0.59 |
| Salivary Gland | PFHxS | 1.12 [0.81, 1.54] | 0.50 |
| Floor of Mouth | PFHxS | 1.08 [0.6, 1.92] | 0.81 |
| Gum and Other Mouth | PFHxS | 1.01 [0.75, 1.37] | 0.93 |
| Nasopharynx | PFHxS | 0.91 [0.53, 1.57] | 0.74 |
| Tonsil | PFHxS | 1.01 [0.78, 1.32] | 0.91 |
| Oropharynx | PFHxS | 0.97 [0.61, 1.55] | 0.90 |
| Hypopharynx | PFHxS | 1.02 [0.59, 1.75] | 0.95 |
| Other Oral Cavity and Pharynx | PFHxS | 0.98 [0.43, 2.24] | 0.97 |
| **Digestive System** | PFHxS | 1 [0.96, 1.05] | 0.90 |
| Esophagus | PFHxS | 1.05 [0.87, 1.26] | 0.62 |
| Stomach | PFHxS | 1.04 [0.9, 1.2] | 0.62 |
| Small Intestine | PFHxS | 0.95 [0.74, 1.21] | 0.65 |
| Colon and Rectum | PFHxS | 1.01 [0.95, 1.08] | 0.67 |
| Colon excluding Rectum | PFHxS | 1.01 [0.94, 1.09] | 0.75 |
| Cecum | PFHxS | 0.96 [0.8, 1.14] | 0.61 |
| Appendix | PFHxS | 0.92 [0.69, 1.23] | 0.57 |
| Ascending Colon | PFHxS | 1.04 [0.87, 1.23] | 0.68 |
| Hepatic Flexure | PFHxS | 1.02 [0.71, 1.47] | 0.91 |
| Transverse Colon | PFHxS | 1.17 [0.93, 1.49] | 0.19 |
| Splenic Flexure | PFHxS | 0.92 [0.57, 1.48] | 0.73 |
| Descending Colon | PFHxS | 1.01 [0.74, 1.38] | 0.96 |
| Sigmoid Colon | PFHxS | 1.01 [0.87, 1.18] | 0.86 |
| Large Intestine NOS | PFHxS | 1.01 [0.77, 1.33] | 0.94 |
| Rectum and Rectosigmoid Junction | PFHxS | 1.02 [0.91, 1.14] | 0.76 |
| Rectosigmoid Junction | PFHxS | 1.12 [0.89, 1.42] | 0.34 |
| Rectum | PFHxS | 0.99 [0.87, 1.13] | 0.87 |
| Anus Anal Canal and Anorectum | PFHxS | 0.96 [0.73, 1.27] | 0.80 |
| Liver and Intrahepatic Bile Duct | PFHxS | 0.89 [0.78, 1.02] | 0.10 |
| Liver | PFHxS | 0.89 [0.76, 1.04] | 0.15 |
| Intrahepatic Bile Duct | PFHxS | 0.9 [0.66, 1.22] | 0.50 |
| Gallbladder | PFHxS | 0.99 [0.69, 1.42] | 0.94 |
| Other Biliary | PFHxS | 1.12 [0.85, 1.48] | 0.44 |
| Pancreas | PFHxS | 1.03 [0.93, 1.14] | 0.61 |
| Retroperitoneum | PFHxS | 0.95 [0.52, 1.73] | 0.85 |
| Peritoneum Omentum and Mesentery | PFHxS | 0.93 [0.51, 1.67] | 0.80 |
| Other Digestive Organs | PFHxS | 1.04 [0.68, 1.58] | 0.86 |
| **Respiratory System** | PFHxS | 1.03 [0.96, 1.1] | 0.39 |
| Nose Nasal Cavity and Middle Ear | PFHxS | 1.01 [0.63, 1.63] | 0.97 |
| Larynx | PFHxS | 0.97 [0.76, 1.23] | 0.81 |
| Lung and Bronchus | PFHxS | 1.03 [0.97, 1.1] | 0.36 |
| Pleura | PFHxS | 1.3 [0.11, 14.67] | 0.83 |
| Trachea Mediastinum and Other Respiratory Organs | PFHxS | 1.18 [0.48, 2.93] | 0.72 |
| Bones and Joints | PFHxS | 0.94 [0.64, 1.39] | 0.77 |
| Soft Tissue including Heart | PFHxS | 0.99 [0.8, 1.22] | 0.93 |
| Skin excluding Basal and Squamous | PFHxS | 1.01 [0.89, 1.13] | 0.93 |
| Melanoma of the Skin | PFHxS | 1.01 [0.89, 1.14] | 0.87 |
| Other Non Epithelial Skin | PFHxS | 0.96 [0.72, 1.28] | 0.77 |
| **Breast** | PFHxS | 0.99 [0.95, 1.04] | 0.78 |
| **Urinary System** | PFHxS | 1 [0.94, 1.07] | 0.89 |
| Urinary Bladder | PFHxS | 1.06 [0.97, 1.16] | 0.19 |
| Kidney and Renal Pelvis | PFHxS | 0.94 [0.86, 1.04] | 0.23 |
| Ureter | PFHxS | 1.13 [0.67, 1.9] | 0.65 |
| Other Urinary Organs | PFHxS | 0.81 [0.42, 1.56] | 0.53 |
| **Brain and Other Nervous System** | PFHxS | 1.02 [0.88, 1.19] | 0.76 |
| Brain | PFHxS | 1.02 [0.87, 1.19] | 0.84 |
| Cranial Nerves Other Nervous System | PFHxS | 1.14 [0.63, 2.06] | 0.66 |
| **Endocrine System** | PFHxS | 1.07 [0.96, 1.2] | 0.20 |
| Thyroid | PFHxS | 1.07 [0.95, 1.2] | 0.25 |
| Other Endocrine including Thymus | PFHxS | 1.13 [0.77, 1.68] | 0.53 |
| **Lymphoma** | PFHxS | 1.04 [0.96, 1.13] | 0.33 |
| **Hodgkin Lymphoma** | PFHxS | 1.03 [0.81, 1.3] | 0.84 |
| Hodgkin Nodal | PFHxS | 1.03 [0.81, 1.31] | 0.81 |
| Hodgkin Extranodal | PFHxS | 0.78 [0.09, 6.93] | 0.83 |
| Non Hodgkin Lymphoma | PFHxS | 1.04 [0.96, 1.14] | 0.33 |
| NHL Nodal | PFHxS | 1.02 [0.92, 1.14] | 0.70 |
| NHL Extranodal | PFHxS | 1.09 [0.94, 1.26] | 0.25 |
| **Myeloma** | PFHxS | 0.95 [0.82, 1.1] | 0.50 |
| **Leukemia** | PFHxS | 1.03 [0.93, 1.14] | 0.54 |
| Lymphocytic Leukemia | PFHxS | 1.04 [0.9, 1.2] | 0.57 |
| Acute Lymphocytic Leukemia | PFHxS | 0.9 [0.67, 1.22] | 0.51 |
| Chronic Lymphocytic Leukemia | PFHxS | 1.1 [0.93, 1.3] | 0.27 |
| Other Lymphocytic Leukemia | PFHxS | 0.99 [0.54, 1.83] | 0.99 |
| Myeloid and Monocytic Leukemia | PFHxS | 1.03 [0.89, 1.2] | 0.69 |
| Acute Myeloid Leukemia | PFHxS | 1.04 [0.86, 1.25] | 0.68 |
| Acute Monocytic Leukemia | PFHxS | 0.9 [0.34, 2.42] | 0.83 |
| Chronic Myeloid Leukemia | PFHxS | 1 [0.76, 1.32] | 0.99 |
| Other Myeloid Monocytic Leukemia | PFHxS | 1.44 [0.53, 3.86] | 0.47 |
| Other Leukemia | PFHxS | 0.94 [0.61, 1.45] | 0.78 |
| Other Acute Leukemia | PFHxS | 0.85 [0.37, 1.97] | 0.71 |
| Aleukemic Subleukemic and NOS | PFHxS | 0.97 [0.58, 1.61] | 0.90 |
| **All Sites** | PFNA | 1.04 [0.97, 1.12] | 0.23 |
| Oral Cavity and Pharynx | PFNA | 0.97 [0.78, 1.21] | 0.77 |
| Lip | PFNA | 1.13 [0.39, 3.26] | 0.82 |
| Tongue | PFNA | 0.87 [0.58, 1.29] | 0.48 |
| Salivary Gland | PFNA | 1.04 [0.55, 1.97] | 0.90 |
| Floor of Mouth | PFNA | 0.9 [0.27, 2.99] | 0.86 |
| Gum and Other Mouth | PFNA | 1.01 [0.56, 1.81] | 0.98 |
| Nasopharynx | PFNA | 1.09 [0.41, 2.88] | 0.86 |
| Tonsil | PFNA | 0.99 [0.6, 1.65] | 0.98 |
| Oropharynx | PFNA | 0.91 [0.36, 2.3] | 0.84 |
| Hypopharynx | PFNA | 0.94 [0.3, 2.93] | 0.92 |
| Other Oral Cavity and Pharynx | PFNA | 1.31 [0.31, 5.52] | 0.71 |
| **Digestive System** | PFNA | 1.04 [0.95, 1.15] | 0.37 |
| Esophagus | PFNA | 0.96 [0.66, 1.38] | 0.82 |
| Stomach | PFNA | 1.13 [0.85, 1.49] | 0.40 |
| Small Intestine | PFNA | 0.95 [0.6, 1.51] | 0.82 |
| Colon and Rectum | PFNA | 1.06 [0.94, 1.21] | 0.35 |
| Colon excluding Rectum | PFNA | 1.07 [0.92, 1.24] | 0.38 |
| Cecum | PFNA | 1.05 [0.75, 1.46] | 0.78 |
| Appendix | PFNA | 1.01 [0.59, 1.72] | 0.98 |
| Ascending Colon | PFNA | 1.03 [0.73, 1.46] | 0.85 |
| Hepatic Flexure | PFNA | 1.22 [0.63, 2.38] | 0.56 |
| Transverse Colon | PFNA | 1.01 [0.62, 1.67] | 0.95 |
| Splenic Flexure | PFNA | 1.19 [0.51, 2.78] | 0.69 |
| Descending Colon | PFNA | 1.08 [0.59, 1.96] | 0.81 |
| Sigmoid Colon | PFNA | 1.12 [0.84, 1.5] | 0.44 |
| Large Intestine NOS | PFNA | 0.96 [0.54, 1.7] | 0.89 |
| Rectum and Rectosigmoid Junction | PFNA | 1.05 [0.84, 1.32] | 0.64 |
| Rectosigmoid Junction | PFNA | 1.12 [0.7, 1.77] | 0.64 |
| Rectum | PFNA | 1.04 [0.8, 1.34] | 0.78 |
| Anus Anal Canal and Anorectum | PFNA | 0.92 [0.54, 1.6] | 0.78 |
| Liver and Intrahepatic Bile Duct | PFNA | 0.89 [0.68, 1.18] | 0.43 |
| Liver | PFNA | 0.87 [0.63, 1.19] | 0.39 |
| Intrahepatic Bile Duct | PFNA | 0.99 [0.56, 1.76] | 0.98 |
| Gallbladder | PFNA | 0.98 [0.49, 1.99] | 0.96 |
| Other Biliary | PFNA | 1.18 [0.69, 2.01] | 0.54 |
| Pancreas | PFNA | 1.07 [0.88, 1.3] | 0.49 |
| Retroperitoneum | PFNA | 1.05 [0.34, 3.23] | 0.93 |
| Peritoneum Omentum and Mesentery | PFNA | 0.93 [0.29, 2.93] | 0.90 |
| Other Digestive Organs | PFNA | 1.43 [0.71, 2.89] | 0.32 |
| **Respiratory System** | PFNA | 1.05 [0.93, 1.2] | 0.43 |
| Nose Nasal Cavity and Middle Ear | PFNA | 1.15 [0.49, 2.74] | 0.75 |
| Larynx | PFNA | 1 [0.63, 1.59] | 0.99 |
| Lung and Bronchus | PFNA | 1.05 [0.93, 1.2] | 0.43 |
| Pleura | PFNA | 0.85 [0, 288.62] | 0.96 |
| Trachea Mediastinum and Other Respiratory Organs | PFNA | 0.87 [0.11, 6.62] | 0.89 |
| Bones and Joints | PFNA | 0.9 [0.42, 1.94] | 0.79 |
| Soft Tissue including Heart | PFNA | 0.96 [0.63, 1.46] | 0.85 |
| Skin excluding Basal and Squamous | PFNA | 0.93 [0.74, 1.18] | 0.56 |
| Melanoma of the Skin | PFNA | 0.93 [0.73, 1.18] | 0.55 |
| Other Non Epithelial Skin | PFNA | 1.01 [0.59, 1.72] | 0.99 |
| **Breast** | PFNA | 1.03 [0.94, 1.13] | 0.54 |
| **Urinary System** | PFNA | 1.03 [0.91, 1.16] | 0.67 |
| Urinary Bladder | PFNA | 1.06 [0.9, 1.25] | 0.48 |
| Kidney and Renal Pelvis | PFNA | 0.97 [0.8, 1.16] | 0.72 |
| Ureter | PFNA | 1.26 [0.49, 3.22] | 0.63 |
| Other Urinary Organs | PFNA | 0.96 [0.3, 3.1] | 0.94 |
| **Brain and Other Nervous System** | PFNA | 1.07 [0.8, 1.43] | 0.65 |
| Brain | PFNA | 1.07 [0.8, 1.45] | 0.64 |
| Cranial Nerves Other Nervous System | PFNA | 1.01 [0.3, 3.42] | 0.99 |
| **Endocrine System** | PFNA | 1.29 [1.05, 1.57] | 0.01 |
| Thyroid | PFNA | 1.28 [1.04, 1.57] | 0.02 |
| Other Endocrine including Thymus | PFNA | 1.42 [0.71, 2.83] | 0.32 |
| **Lymphoma** | PFNA | 1 [0.85, 1.18] | 0.96 |
| **Hodgkin Lymphoma** | PFNA | 0.95 [0.59, 1.53] | 0.84 |
| Hodgkin Nodal | PFNA | 0.95 [0.59, 1.54] | 0.85 |
| Hodgkin Extranodal | PFNA | 0.6 [0.01, 63.06] | 0.83 |
| Non Hodgkin Lymphoma | PFNA | 1.01 [0.85, 1.2] | 0.90 |
| NHL Nodal | PFNA | 0.99 [0.8, 1.23] | 0.94 |
| NHL Extranodal | PFNA | 1.05 [0.79, 1.4] | 0.75 |
| **Myeloma** | PFNA | 0.99 [0.75, 1.3] | 0.95 |
| **Leukemia** | PFNA | 0.99 [0.81, 1.2] | 0.91 |
| Lymphocytic Leukemia | PFNA | 0.97 [0.74, 1.29] | 0.84 |
| Acute Lymphocytic Leukemia | PFNA | 1.03 [0.59, 1.81] | 0.91 |
| Chronic Lymphocytic Leukemia | PFNA | 0.95 [0.68, 1.32] | 0.75 |
| Other Lymphocytic Leukemia | PFNA | 1.02 [0.33, 3.19] | 0.97 |
| Myeloid and Monocytic Leukemia | PFNA | 1.02 [0.76, 1.37] | 0.91 |
| Acute Myeloid Leukemia | PFNA | 0.98 [0.68, 1.42] | 0.92 |
| Acute Monocytic Leukemia | PFNA | 1.05 [0.18, 6.28] | 0.96 |
| Chronic Myeloid Leukemia | PFNA | 1.08 [0.64, 1.8] | 0.78 |
| Other Myeloid Monocytic Leukemia | PFNA | 1.31 [0.18, 9.68] | 0.79 |
| Other Leukemia | PFNA | 0.84 [0.33, 2.13] | 0.71 |
| Other Acute Leukemia | PFNA | 0.58 [0.07, 4.52] | 0.60 |
| Aleukemic Subleukemic and NOS | PFNA | 0.96 [0.34, 2.7] | 0.94 |
| **All Sites** | PFBS | 1.02 [0.93, 1.12] | 0.68 |
| Oral Cavity and Pharynx | PFBS | 1.33 [1.04, 1.71] | 0.02 |
| Lip | PFBS | 0.53 [0.08, 3.63] | 0.52 |
| Tongue | PFBS | 1.5 [0.97, 2.34] | 0.07 |
| Salivary Gland | PFBS | 1.62 [0.81, 3.24] | 0.18 |
| Floor of Mouth | PFBS | 1.14 [0.28, 4.62] | 0.86 |
| Gum and Other Mouth | PFBS | 1.58 [0.83, 3.02] | 0.17 |
| Nasopharynx | PFBS | 0.84 [0.17, 4.12] | 0.83 |
| Tonsil | PFBS | 1.2 [0.69, 2.09] | 0.51 |
| Oropharynx | PFBS | 1.33 [0.49, 3.62] | 0.58 |
| Hypopharynx | PFBS | 1.22 [0.4, 3.76] | 0.73 |
| Other Oral Cavity and Pharynx | PFBS | 0.86 [0.13, 5.64] | 0.88 |
| **Digestive System** | PFBS | 0.92 [0.81, 1.04] | 0.16 |
| Esophagus | PFBS | 1.17 [0.77, 1.77] | 0.47 |
| Stomach | PFBS | 0.85 [0.58, 1.26] | 0.43 |
| Small Intestine | PFBS | 1.08 [0.58, 2.02] | 0.80 |
| Colon and Rectum | PFBS | 0.87 [0.74, 1.02] | 0.09 |
| Colon excluding Rectum | PFBS | 0.87 [0.72, 1.05] | 0.15 |
| Cecum | PFBS | 0.86 [0.56, 1.33] | 0.50 |
| Appendix | PFBS | 1.01 [0.48, 2.13] | 0.97 |
| Ascending Colon | PFBS | 1.19 [0.81, 1.74] | 0.38 |
| Hepatic Flexure | PFBS | 0.67 [0.25, 1.84] | 0.44 |
| Transverse Colon | PFBS | 0.99 [0.55, 1.79] | 0.98 |
| Splenic Flexure | PFBS | 0.82 [0.25, 2.68] | 0.74 |
| Descending Colon | PFBS | 0.72 [0.3, 1.73] | 0.46 |
| Sigmoid Colon | PFBS | 0.78 [0.53, 1.15] | 0.21 |
| Large Intestine NOS | PFBS | 0.59 [0.28, 1.24] | 0.17 |
| Rectum and Rectosigmoid Junction | PFBS | 0.86 [0.65, 1.15] | 0.31 |
| Rectosigmoid Junction | PFBS | 0.79 [0.43, 1.42] | 0.43 |
| Rectum | PFBS | 0.89 [0.64, 1.23] | 0.47 |
| Anus Anal Canal and Anorectum | PFBS | 1.27 [0.67, 2.38] | 0.46 |
| Liver and Intrahepatic Bile Duct | PFBS | 0.89 [0.65, 1.24] | 0.50 |
| Liver | PFBS | 0.82 [0.57, 1.19] | 0.30 |
| Intrahepatic Bile Duct | PFBS | 1.38 [0.7, 2.72] | 0.35 |
| Gallbladder | PFBS | 0.36 [0.09, 1.53] | 0.17 |
| Other Biliary | PFBS | 1.07 [0.52, 2.19] | 0.86 |
| Pancreas | PFBS | 1.05 [0.81, 1.37] | 0.71 |
| Retroperitoneum | PFBS | 0.82 [0.16, 4.19] | 0.81 |
| Peritoneum Omentum and Mesentery | PFBS | 0.8 [0.14, 4.42] | 0.80 |
| Other Digestive Organs | PFBS | 0.78 [0.24, 2.57] | 0.68 |
| **Respiratory System** | PFBS | 1.16 [1, 1.35] | 0.06 |
| Nose Nasal Cavity and Middle Ear | PFBS | 1.26 [0.41, 3.87] | 0.68 |
| Larynx | PFBS | 1.26 [0.81, 1.95] | 0.31 |
| Lung and Bronchus | PFBS | 1.15 [0.99, 1.34] | 0.07 |
| Pleura | PFBS | #VALUE! | NA |
| Trachea Mediastinum and Other Respiratory Organs | PFBS | 1.72 [0.32, 9.18] | 0.53 |
| Bones and Joints | PFBS | 1.47 [0.64, 3.37] | 0.36 |
| Soft Tissue including Heart | PFBS | 1.3 [0.79, 2.17] | 0.30 |
| Skin excluding Basal and Squamous | PFBS | 1.06 [0.77, 1.46] | 0.71 |
| Melanoma of the Skin | PFBS | 1.06 [0.76, 1.47] | 0.75 |
| Other Non Epithelial Skin | PFBS | 1.15 [0.54, 2.45] | 0.72 |
| **Breast** | PFBS | 1.12 [0.98, 1.27] | 0.10 |
| **Urinary System** | PFBS | 0.98 [0.84, 1.15] | 0.84 |
| Urinary Bladder | PFBS | 1.15 [0.93, 1.43] | 0.21 |
| Kidney and Renal Pelvis | PFBS | 0.87 [0.69, 1.09] | 0.22 |
| Ureter | PFBS | 0.86 [0.19, 3.75] | 0.84 |
| Other Urinary Organs | PFBS | 0.83 [0.13, 5.23] | 0.84 |
| **Brain and Other Nervous System** | PFBS | 1.01 [0.69, 1.49] | 0.95 |
| Brain | PFBS | 1.07 [0.72, 1.57] | 0.75 |
| Cranial Nerves Other Nervous System | PFBS | 0.3 [0.02, 3.87] | 0.36 |
| **Endocrine System** | PFBS | 0.82 [0.6, 1.13] | 0.23 |
| Thyroid | PFBS | 0.81 [0.58, 1.13] | 0.21 |
| Other Endocrine including Thymus | PFBS | 1.01 [0.34, 2.99] | 0.99 |
| **Lymphoma** | PFBS | 0.96 [0.77, 1.2] | 0.72 |
| **Hodgkin Lymphoma** | PFBS | 0.91 [0.47, 1.76] | 0.78 |
| Hodgkin Nodal | PFBS | 0.92 [0.47, 1.77] | 0.79 |
| Hodgkin Extranodal | PFBS | 0.71 [0.01, 63.47] | 0.88 |
| Non Hodgkin Lymphoma | PFBS | 0.97 [0.77, 1.22] | 0.79 |
| NHL Nodal | PFBS | 0.99 [0.75, 1.31] | 0.96 |
| NHL Extranodal | PFBS | 0.92 [0.59, 1.42] | 0.69 |
| **Myeloma** | PFBS | 1.07 [0.72, 1.58] | 0.74 |
| **Leukemia** | PFBS | 1.06 [0.83, 1.36] | 0.62 |
| Lymphocytic Leukemia | PFBS | 1.12 [0.79, 1.61] | 0.52 |
| Acute Lymphocytic Leukemia | PFBS | 0.81 [0.36, 1.81] | 0.60 |
| Chronic Lymphocytic Leukemia | PFBS | 1.23 [0.81, 1.86] | 0.34 |
| Other Lymphocytic Leukemia | PFBS | 1.8 [0.46, 7] | 0.40 |
| Myeloid and Monocytic Leukemia | PFBS | 1.02 [0.71, 1.48] | 0.91 |
| Acute Myeloid Leukemia | PFBS | 0.86 [0.52, 1.44] | 0.57 |
| Acute Monocytic Leukemia | PFBS | 1.45 [0.24, 8.9] | 0.69 |
| Chronic Myeloid Leukemia | PFBS | 1.33 [0.76, 2.33] | 0.32 |
| Other Myeloid Monocytic Leukemia | PFBS | 0.25 [0, 40.01] | 0.59 |
| Other Leukemia | PFBS | 0.99 [0.39, 2.55] | 0.99 |
| Other Acute Leukemia | PFBS | 0.4 [0.03, 5.37] | 0.49 |
| Aleukemic Subleukemic and NOS | PFBS | 1.25 [0.45, 3.45] | 0.67 |
| 1. All models were adjusted for county-level SES variables, urbanicity, smoking rate, obesity, and air pollution. | | | |
| 2. cells were highlighted if crude p values were less than 0.05. | | | |

| **Supplemental Table 5.** Association between detection of PFAS based on UCMR5 and county-level cancer incidence for each sub-type. | | | |
| --- | --- | --- | --- |
| **Cancers** | **Exposures** | **IRR [95% CI]** | **p value^1,2^** |
| **All Sites** | PFBS | 1 [0.98, 1.02] | 0.98 |
| Oral Cavity and Pharynx | PFBS | 1.05 [0.99, 1.1] | 0.10 |
| Lip | PFBS | 0.98 [0.77, 1.25] | 0.88 |
| Tongue | PFBS | 1.06 [0.96, 1.16] | 0.27 |
| Salivary Gland | PFBS | 1.1 [0.94, 1.3] | 0.24 |
| Floor of Mouth | PFBS | 1.01 [0.77, 1.34] | 0.92 |
| Gum and Other Mouth | PFBS | 0.99 [0.86, 1.15] | 0.94 |
| Nasopharynx | PFBS | 0.95 [0.72, 1.26] | 0.71 |
| Tonsil | PFBS | 1.09 [0.96, 1.23] | 0.19 |
| Oropharynx | PFBS | 0.96 [0.77, 1.2] | 0.74 |
| Hypopharynx | PFBS | 1.04 [0.81, 1.34] | 0.76 |
| Other Oral Cavity and Pharynx | PFBS | 1.33 [0.93, 1.91] | 0.12 |
| **Digestive System** | PFBS | 1.02 [0.99, 1.04] | 0.17 |
| Esophagus | PFBS | 1.04 [0.96, 1.14] | 0.33 |
| Stomach | PFBS | 1.04 [0.96, 1.12] | 0.36 |
| Small Intestine | PFBS | 0.98 [0.87, 1.1] | 0.75 |
| Colon and Rectum | PFBS | 1.02 [0.98, 1.05] | 0.32 |
| Colon excluding Rectum | PFBS | 1.02 [0.99, 1.06] | 0.25 |
| Cecum | PFBS | 1.06 [0.98, 1.15] | 0.14 |
| Appendix | PFBS | 0.96 [0.83, 1.1] | 0.54 |
| Ascending Colon | PFBS | 0.99 [0.91, 1.07] | 0.75 |
| Hepatic Flexure | PFBS | 1.03 [0.87, 1.23] | 0.69 |
| Transverse Colon | PFBS | 1.03 [0.92, 1.16] | 0.57 |
| Splenic Flexure | PFBS | 1 [0.81, 1.25] | 0.97 |
| Descending Colon | PFBS | 0.98 [0.84, 1.14] | 0.78 |
| Sigmoid Colon | PFBS | 1.02 [0.95, 1.1] | 0.56 |
| Large Intestine NOS | PFBS | 1.08 [0.95, 1.23] | 0.23 |
| Rectum and Rectosigmoid Junction | PFBS | 1 [0.95, 1.06] | 0.90 |
| Rectosigmoid Junction | PFBS | 1.07 [0.95, 1.2] | 0.29 |
| Rectum | PFBS | 0.99 [0.93, 1.05] | 0.70 |
| Anus Anal Canal and Anorectum | PFBS | 1.03 [0.9, 1.17] | 0.71 |
| Liver and Intrahepatic Bile Duct | PFBS | 1.04 [0.97, 1.11] | 0.26 |
| Liver | PFBS | 1.04 [0.97, 1.12] | 0.30 |
| Intrahepatic Bile Duct | PFBS | 1.04 [0.9, 1.21] | 0.58 |
| Gallbladder | PFBS | 1.06 [0.88, 1.27] | 0.55 |
| Other Biliary | PFBS | 1.05 [0.9, 1.21] | 0.55 |
| Pancreas | PFBS | 0.99 [0.94, 1.04] | 0.60 |
| Retroperitoneum | PFBS | 1.01 [0.75, 1.36] | 0.94 |
| Peritoneum Omentum and Mesentery | PFBS | 1.06 [0.79, 1.42] | 0.68 |
| Other Digestive Organs | PFBS | 0.98 [0.79, 1.21] | 0.82 |
| **Respiratory System** | PFBS | 1.02 [0.99, 1.05] | 0.30 |
| Nose Nasal Cavity and Middle Ear | PFBS | 0.99 [0.79, 1.26] | 0.97 |
| Larynx | PFBS | 0.98 [0.88, 1.08] | 0.67 |
| Lung and Bronchus | PFBS | 1.02 [0.99, 1.05] | 0.26 |
| Pleura | PFBS | 0.53 [0.15, 1.96] | 0.34 |
| Trachea Mediastinum and Other Respiratory Organs | PFBS | 1.17 [0.77, 1.79] | 0.46 |
| Bones and Joints | PFBS | 0.99 [0.81, 1.19] | 0.88 |
| Soft Tissue including Heart | PFBS | 1.01 [0.91, 1.12] | 0.90 |
| Skin excluding Basal and Squamous | PFBS | 1 [0.95, 1.06] | 0.93 |
| Melanoma of the Skin | PFBS | 1.02 [0.96, 1.08] | 0.56 |
| Other Non Epithelial Skin | PFBS | 0.83 [0.72, 0.96] | 0.01 |
| **Breast** | PFBS | 1 [0.97, 1.02] | 0.86 |
| **Urinary System** | PFBS | 0.98 [0.96, 1.01] | 0.32 |
| Urinary Bladder | PFBS | 0.97 [0.93, 1.02] | 0.23 |
| Kidney and Renal Pelvis | PFBS | 1 [0.95, 1.04] | 0.84 |
| Ureter | PFBS | 0.92 [0.71, 1.21] | 0.56 |
| Other Urinary Organs | PFBS | 0.92 [0.69, 1.24] | 0.60 |
| **Brain and Other Nervous System** | PFBS | 1.01 [0.94, 1.09] | 0.73 |
| Brain | PFBS | 1.01 [0.94, 1.09] | 0.78 |
| Cranial Nerves Other Nervous System | PFBS | 1.02 [0.76, 1.39] | 0.88 |
| **Endocrine System** | PFBS | 0.97 [0.91, 1.02] | 0.24 |
| Thyroid | PFBS | 0.96 [0.9, 1.02] | 0.19 |
| Other Endocrine including Thymus | PFBS | 1.04 [0.85, 1.27] | 0.72 |
| **Lymphoma** | PFBS | 0.98 [0.94, 1.02] | 0.34 |
| **Hodgkin Lymphoma** | PFBS | 1.02 [0.91, 1.15] | 0.73 |
| Hodgkin Nodal | PFBS | 1.03 [0.91, 1.16] | 0.63 |
| Hodgkin Extranodal | PFBS | 0.57 [0.19, 1.7] | 0.32 |
| Non Hodgkin Lymphoma | PFBS | 0.97 [0.93, 1.02] | 0.25 |
| NHL Nodal | PFBS | 0.97 [0.91, 1.02] | 0.21 |
| NHL Extranodal | PFBS | 0.99 [0.92, 1.07] | 0.83 |
| **Myeloma** | PFBS | 1.02 [0.95, 1.09] | 0.65 |
| **Leukemia** | PFBS | 0.98 [0.94, 1.03] | 0.54 |
| Lymphocytic Leukemia | PFBS | 0.98 [0.92, 1.06] | 0.65 |
| Acute Lymphocytic Leukemia | PFBS | 0.98 [0.84, 1.14] | 0.78 |
| Chronic Lymphocytic Leukemia | PFBS | 1 [0.92, 1.09] | 0.99 |
| Other Lymphocytic Leukemia | PFBS | 0.79 [0.58, 1.09] | 0.16 |
| Myeloid and Monocytic Leukemia | PFBS | 0.96 [0.9, 1.04] | 0.34 |
| Acute Myeloid Leukemia | PFBS | 0.95 [0.87, 1.05] | 0.31 |
| Acute Monocytic Leukemia | PFBS | 1.07 [0.68, 1.67] | 0.77 |
| Chronic Myeloid Leukemia | PFBS | 0.98 [0.86, 1.12] | 0.75 |
| Other Myeloid Monocytic Leukemia | PFBS | 0.98 [0.57, 1.69] | 0.94 |
| Other Leukemia | PFBS | 1.16 [0.95, 1.42] | 0.14 |
| Other Acute Leukemia | PFBS | 1.22 [0.84, 1.78] | 0.29 |
| Aleukemic Subleukemic and NOS | PFBS | 1.14 [0.9, 1.44] | 0.27 |
| **All Sites** | PFHpA | 1.01 [0.98, 1.03] | 0.55 |
| Oral Cavity and Pharynx | PFHpA | 1.01 [0.94, 1.09] | 0.77 |
| Lip | PFHpA | 1.04 [0.73, 1.49] | 0.81 |
| Tongue | PFHpA | 1.02 [0.89, 1.16] | 0.81 |
| Salivary Gland | PFHpA | 1.02 [0.82, 1.28] | 0.84 |
| Floor of Mouth | PFHpA | 0.89 [0.6, 1.33] | 0.58 |
| Gum and Other Mouth | PFHpA | 1.04 [0.85, 1.27] | 0.71 |
| Nasopharynx | PFHpA | 1.04 [0.72, 1.51] | 0.83 |
| Tonsil | PFHpA | 1.01 [0.85, 1.2] | 0.89 |
| Oropharynx | PFHpA | 0.92 [0.68, 1.26] | 0.62 |
| Hypopharynx | PFHpA | 0.95 [0.66, 1.35] | 0.77 |
| Other Oral Cavity and Pharynx | PFHpA | 1.23 [0.74, 2.06] | 0.42 |
| **Digestive System** | PFHpA | 1.01 [0.98, 1.04] | 0.57 |
| Esophagus | PFHpA | 1 [0.88, 1.13] | 0.99 |
| Stomach | PFHpA | 1.03 [0.93, 1.14] | 0.58 |
| Small Intestine | PFHpA | 0.9 [0.76, 1.06] | 0.21 |
| Colon and Rectum | PFHpA | 1.03 [0.98, 1.08] | 0.21 |
| Colon excluding Rectum | PFHpA | 1.02 [0.97, 1.08] | 0.37 |
| Cecum | PFHpA | 1.05 [0.93, 1.17] | 0.44 |
| Appendix | PFHpA | 0.99 [0.82, 1.2] | 0.95 |
| Ascending Colon | PFHpA | 0.99 [0.88, 1.11] | 0.84 |
| Hepatic Flexure | PFHpA | 1.12 [0.88, 1.41] | 0.36 |
| Transverse Colon | PFHpA | 1.09 [0.93, 1.29] | 0.27 |
| Splenic Flexure | PFHpA | 1.02 [0.76, 1.38] | 0.88 |
| Descending Colon | PFHpA | 1.05 [0.85, 1.29] | 0.67 |
| Sigmoid Colon | PFHpA | 1 [0.91, 1.11] | 0.93 |
| Large Intestine NOS | PFHpA | 1 [0.84, 1.21] | 0.97 |
| Rectum and Rectosigmoid Junction | PFHpA | 1.04 [0.97, 1.13] | 0.27 |
| Rectosigmoid Junction | PFHpA | 1.07 [0.91, 1.25] | 0.44 |
| Rectum | PFHpA | 1.04 [0.95, 1.13] | 0.41 |
| Anus Anal Canal and Anorectum | PFHpA | 0.96 [0.8, 1.16] | 0.66 |
| Liver and Intrahepatic Bile Duct | PFHpA | 0.96 [0.87, 1.05] | 0.35 |
| Liver | PFHpA | 0.95 [0.85, 1.05] | 0.31 |
| Intrahepatic Bile Duct | PFHpA | 1.01 [0.82, 1.24] | 0.92 |
| Gallbladder | PFHpA | 1.04 [0.82, 1.34] | 0.73 |
| Other Biliary | PFHpA | 1.02 [0.83, 1.24] | 0.87 |
| Pancreas | PFHpA | 1.01 [0.94, 1.08] | 0.81 |
| Retroperitoneum | PFHpA | 0.98 [0.65, 1.48] | 0.93 |
| Peritoneum Omentum and Mesentery | PFHpA | 1.07 [0.73, 1.59] | 0.72 |
| Other Digestive Organs | PFHpA | 0.93 [0.69, 1.26] | 0.65 |
| **Respiratory System** | PFHpA | 1.03 [0.99, 1.07] | 0.18 |
| Nose Nasal Cavity and Middle Ear | PFHpA | 0.97 [0.7, 1.34] | 0.86 |
| Larynx | PFHpA | 0.94 [0.81, 1.09] | 0.43 |
| Lung and Bronchus | PFHpA | 1.04 [0.99, 1.08] | 0.12 |
| Pleura | PFHpA | 1.15 [0.23, 5.7] | 0.86 |
| Trachea Mediastinum and Other Respiratory Organs | PFHpA | 0.94 [0.5, 1.76] | 0.84 |
| Bones and Joints | PFHpA | 1.01 [0.78, 1.31] | 0.91 |
| Soft Tissue including Heart | PFHpA | 1 [0.87, 1.16] | 0.97 |
| Skin excluding Basal and Squamous | PFHpA | 0.93 [0.86, 1.01] | 0.10 |
| Melanoma of the Skin | PFHpA | 0.94 [0.86, 1.02] | 0.15 |
| Other Non Epithelial Skin | PFHpA | 0.87 [0.71, 1.06] | 0.16 |
| **Breast** | PFHpA | 0.99 [0.96, 1.03] | 0.67 |
| **Urinary System** | PFHpA | 1.01 [0.97, 1.05] | 0.76 |
| Urinary Bladder | PFHpA | 1.01 [0.95, 1.07] | 0.79 |
| Kidney and Renal Pelvis | PFHpA | 1.01 [0.95, 1.07] | 0.75 |
| Ureter | PFHpA | 1 [0.7, 1.44] | 0.98 |
| Other Urinary Organs | PFHpA | 0.79 [0.51, 1.22] | 0.29 |
| **Brain and Other Nervous System** | PFHpA | 1.07 [0.96, 1.18] | 0.21 |
| Brain | PFHpA | 1.05 [0.95, 1.17] | 0.32 |
| Cranial Nerves Other Nervous System | PFHpA | 1.25 [0.83, 1.89] | 0.29 |
| **Endocrine System** | PFHpA | 1.09 [1.01, 1.18] | 0.02 |
| Thyroid | PFHpA | 1.1 [1.01, 1.19] | 0.02 |
| Other Endocrine including Thymus | PFHpA | 1.04 [0.8, 1.37] | 0.75 |
| **Lymphoma** | PFHpA | 1.03 [0.97, 1.09] | 0.35 |
| **Hodgkin Lymphoma** | PFHpA | 1.09 [0.93, 1.28] | 0.29 |
| Hodgkin Nodal | PFHpA | 1.1 [0.93, 1.29] | 0.25 |
| Hodgkin Extranodal | PFHpA | 0.7 [0.15, 3.2] | 0.64 |
| Non Hodgkin Lymphoma | PFHpA | 1.02 [0.96, 1.08] | 0.56 |
| NHL Nodal | PFHpA | 1.02 [0.95, 1.1] | 0.61 |
| NHL Extranodal | PFHpA | 1.01 [0.91, 1.13] | 0.79 |
| **Myeloma** | PFHpA | 0.98 [0.89, 1.09] | 0.76 |
| **Leukemia** | PFHpA | 1.01 [0.94, 1.08] | 0.77 |
| Lymphocytic Leukemia | PFHpA | 1.02 [0.93, 1.13] | 0.64 |
| Acute Lymphocytic Leukemia | PFHpA | 0.99 [0.81, 1.21] | 0.91 |
| Chronic Lymphocytic Leukemia | PFHpA | 1.04 [0.93, 1.17] | 0.47 |
| Other Lymphocytic Leukemia | PFHpA | 0.93 [0.61, 1.41] | 0.72 |
| Myeloid and Monocytic Leukemia | PFHpA | 0.99 [0.9, 1.1] | 0.91 |
| Acute Myeloid Leukemia | PFHpA | 0.97 [0.86, 1.11] | 0.68 |
| Acute Monocytic Leukemia | PFHpA | 1.12 [0.61, 2.05] | 0.71 |
| Chronic Myeloid Leukemia | PFHpA | 1.01 [0.84, 1.21] | 0.92 |
| Other Myeloid Monocytic Leukemia | PFHpA | 1.33 [0.66, 2.7] | 0.42 |
| Other Leukemia | PFHpA | 1.02 [0.77, 1.36] | 0.87 |
| Other Acute Leukemia | PFHpA | 1.27 [0.76, 2.12] | 0.36 |
| Aleukemic Subleukemic and NOS | PFHpA | 0.94 [0.67, 1.31] | 0.70 |
| **All Sites** | PFBA | 1 [0.98, 1.01] | 0.69 |
| Oral Cavity and Pharynx | PFBA | 1.04 [0.99, 1.09] | 0.13 |
| Lip | PFBA | 0.71 [0.57, 0.89] | 0.00 |
| Tongue | PFBA | 1.1 [1.02, 1.2] | 0.02 |
| Salivary Gland | PFBA | 1.06 [0.91, 1.22] | 0.45 |
| Floor of Mouth | PFBA | 0.92 [0.72, 1.17] | 0.48 |
| Gum and Other Mouth | PFBA | 1.03 [0.9, 1.17] | 0.67 |
| Nasopharynx | PFBA | 0.99 [0.77, 1.28] | 0.95 |
| Tonsil | PFBA | 1.04 [0.94, 1.16] | 0.46 |
| Oropharynx | PFBA | 1.1 [0.91, 1.33] | 0.33 |
| Hypopharynx | PFBA | 0.93 [0.74, 1.16] | 0.52 |
| Other Oral Cavity and Pharynx | PFBA | 1.38 [1.01, 1.91] | 0.05 |
| **Digestive System** | PFBA | 1.02 [1, 1.05] | 0.03 |
| Esophagus | PFBA | 1.03 [0.96, 1.12] | 0.40 |
| Stomach | PFBA | 1.04 [0.97, 1.11] | 0.28 |
| Small Intestine | PFBA | 0.98 [0.88, 1.08] | 0.64 |
| Colon and Rectum | PFBA | 1.02 [0.99, 1.05] | 0.24 |
| Colon excluding Rectum | PFBA | 1.02 [0.99, 1.05] | 0.21 |
| Cecum | PFBA | 1.02 [0.95, 1.1] | 0.57 |
| Appendix | PFBA | 0.96 [0.84, 1.08] | 0.48 |
| Ascending Colon | PFBA | 0.98 [0.91, 1.05] | 0.52 |
| Hepatic Flexure | PFBA | 1.04 [0.89, 1.21] | 0.62 |
| Transverse Colon | PFBA | 1.06 [0.95, 1.17] | 0.29 |
| Splenic Flexure | PFBA | 1.11 [0.92, 1.35] | 0.27 |
| Descending Colon | PFBA | 0.94 [0.82, 1.07] | 0.36 |
| Sigmoid Colon | PFBA | 1.01 [0.95, 1.08] | 0.69 |
| Large Intestine NOS | PFBA | 1.2 [1.07, 1.34] | 0.00 |
| Rectum and Rectosigmoid Junction | PFBA | 1.01 [0.96, 1.06] | 0.67 |
| Rectosigmoid Junction | PFBA | 1.05 [0.94, 1.16] | 0.39 |
| Rectum | PFBA | 1 [0.95, 1.06] | 0.96 |
| Anus Anal Canal and Anorectum | PFBA | 1.01 [0.9, 1.14] | 0.86 |
| Liver and Intrahepatic Bile Duct | PFBA | 1.09 [1.03, 1.16] | 0.00 |
| Liver | PFBA | 1.1 [1.03, 1.18] | 0.00 |
| Intrahepatic Bile Duct | PFBA | 1.02 [0.89, 1.17] | 0.74 |
| Gallbladder | PFBA | 1.05 [0.89, 1.24] | 0.58 |
| Other Biliary | PFBA | 1.06 [0.93, 1.21] | 0.36 |
| Pancreas | PFBA | 1.01 [0.96, 1.05] | 0.82 |
| Retroperitoneum | PFBA | 0.98 [0.75, 1.28] | 0.89 |
| Peritoneum Omentum and Mesentery | PFBA | 0.94 [0.72, 1.23] | 0.64 |
| Other Digestive Organs | PFBA | 1.01 [0.83, 1.22] | 0.93 |
| **Respiratory System** | PFBA | 1.03 [1.01, 1.06] | 0.02 |
| Nose Nasal Cavity and Middle Ear | PFBA | 1.07 [0.87, 1.32] | 0.52 |
| Larynx | PFBA | 1 [0.92, 1.1] | 0.93 |
| Lung and Bronchus | PFBA | 1.03 [1.01, 1.06] | 0.02 |
| Pleura | PFBA | 0.62 [0.22, 1.79] | 0.38 |
| Trachea Mediastinum and Other Respiratory Organs | PFBA | 0.8 [0.54, 1.19] | 0.28 |
| Bones and Joints | PFBA | 0.93 [0.78, 1.11] | 0.44 |
| Soft Tissue including Heart | PFBA | 0.97 [0.88, 1.07] | 0.53 |
| Skin excluding Basal and Squamous | PFBA | 0.89 [0.84, 0.93] | 0.00 |
| Melanoma of the Skin | PFBA | 0.89 [0.84, 0.94] | 0.00 |
| Other Non Epithelial Skin | PFBA | 0.86 [0.76, 0.97] | 0.02 |
| **Breast** | PFBA | 0.98 [0.96, 1] | 0.09 |
| **Urinary System** | PFBA | 1.02 [1, 1.05] | 0.09 |
| Urinary Bladder | PFBA | 1.01 [0.97, 1.05] | 0.77 |
| Kidney and Renal Pelvis | PFBA | 1.04 [1.01, 1.09] | 0.02 |
| Ureter | PFBA | 1 [0.79, 1.27] | 0.98 |
| Other Urinary Organs | PFBA | 0.91 [0.7, 1.19] | 0.50 |
| **Brain and Other Nervous System** | PFBA | 0.99 [0.92, 1.05] | 0.69 |
| Brain | PFBA | 0.96 [0.89, 1.03] | 0.23 |
| Cranial Nerves Other Nervous System | PFBA | 1.4 [1.08, 1.82] | 0.01 |
| **Endocrine System** | PFBA | 0.96 [0.91, 1.01] | 0.09 |
| Thyroid | PFBA | 0.95 [0.89, 1] | 0.04 |
| Other Endocrine including Thymus | PFBA | 1.12 [0.93, 1.34] | 0.23 |
| **Lymphoma** | PFBA | 1 [0.96, 1.03] | 0.84 |
| **Hodgkin Lymphoma** | PFBA | 1.04 [0.94, 1.16] | 0.42 |
| Hodgkin Nodal | PFBA | 1.04 [0.94, 1.16] | 0.45 |
| Hodgkin Extranodal | PFBA | 1.18 [0.52, 2.69] | 0.69 |
| Non Hodgkin Lymphoma | PFBA | 0.99 [0.95, 1.03] | 0.60 |
| NHL Nodal | PFBA | 1 [0.96, 1.05] | 0.90 |
| NHL Extranodal | PFBA | 0.96 [0.89, 1.03] | 0.26 |
| **Myeloma** | PFBA | 1.01 [0.95, 1.08] | 0.66 |
| **Leukemia** | PFBA | 0.96 [0.91, 1] | 0.05 |
| Lymphocytic Leukemia | PFBA | 0.98 [0.92, 1.05] | 0.61 |
| Acute Lymphocytic Leukemia | PFBA | 0.98 [0.86, 1.12] | 0.75 |
| Chronic Lymphocytic Leukemia | PFBA | 0.98 [0.91, 1.05] | 0.57 |
| Other Lymphocytic Leukemia | PFBA | 1.08 [0.82, 1.43] | 0.56 |
| Myeloid and Monocytic Leukemia | PFBA | 0.91 [0.85, 0.97] | 0.00 |
| Acute Myeloid Leukemia | PFBA | 0.89 [0.81, 0.96] | 0.00 |
| Acute Monocytic Leukemia | PFBA | 0.81 [0.54, 1.21] | 0.30 |
| Chronic Myeloid Leukemia | PFBA | 0.94 [0.83, 1.05] | 0.28 |
| Other Myeloid Monocytic Leukemia | PFBA | 1.29 [0.8, 2.09] | 0.29 |
| Other Leukemia | PFBA | 1.13 [0.95, 1.35] | 0.17 |
| Other Acute Leukemia | PFBA | 1.16 [0.83, 1.62] | 0.37 |
| Aleukemic Subleukemic and NOS | PFBA | 1.12 [0.91, 1.38] | 0.27 |
| **All Sites** | PFHXA | 1 [0.98, 1.01] | 0.67 |
| Oral Cavity and Pharynx | PFHXA | 1.02 [0.96, 1.07] | 0.58 |
| Lip | PFHXA | 1.06 [0.83, 1.36] | 0.62 |
| Tongue | PFHXA | 1.02 [0.93, 1.13] | 0.64 |
| Salivary Gland | PFHXA | 1.02 [0.86, 1.2] | 0.85 |
| Floor of Mouth | PFHXA | 0.91 [0.68, 1.21] | 0.51 |
| Gum and Other Mouth | PFHXA | 1.03 [0.89, 1.2] | 0.65 |
| Nasopharynx | PFHXA | 0.85 [0.63, 1.14] | 0.27 |
| Tonsil | PFHXA | 1.07 [0.94, 1.21] | 0.30 |
| Oropharynx | PFHXA | 0.95 [0.76, 1.2] | 0.69 |
| Hypopharynx | PFHXA | 0.85 [0.65, 1.11] | 0.24 |
| Other Oral Cavity and Pharynx | PFHXA | 1.33 [0.91, 1.94] | 0.13 |
| **Digestive System** | PFHXA | 1.01 [0.98, 1.03] | 0.56 |
| Esophagus | PFHXA | 1.02 [0.93, 1.11] | 0.73 |
| Stomach | PFHXA | 1.03 [0.95, 1.11] | 0.52 |
| Small Intestine | PFHXA | 0.98 [0.87, 1.11] | 0.76 |
| Colon and Rectum | PFHXA | 1 [0.96, 1.03] | 0.89 |
| Colon excluding Rectum | PFHXA | 1 [0.96, 1.04] | 0.90 |
| Cecum | PFHXA | 0.99 [0.91, 1.08] | 0.82 |
| Appendix | PFHXA | 0.94 [0.81, 1.09] | 0.41 |
| Ascending Colon | PFHXA | 0.99 [0.91, 1.08] | 0.84 |
| Hepatic Flexure | PFHXA | 0.86 [0.72, 1.04] | 0.11 |
| Transverse Colon | PFHXA | 1.03 [0.91, 1.16] | 0.65 |
| Splenic Flexure | PFHXA | 0.97 [0.77, 1.22] | 0.78 |
| Descending Colon | PFHXA | 0.93 [0.8, 1.09] | 0.38 |
| Sigmoid Colon | PFHXA | 0.99 [0.91, 1.06] | 0.71 |
| Large Intestine NOS | PFHXA | 1.24 [1.09, 1.41] | 0.00 |
| Rectum and Rectosigmoid Junction | PFHXA | 1 [0.94, 1.06] | 0.93 |
| Rectosigmoid Junction | PFHXA | 1.08 [0.95, 1.22] | 0.24 |
| Rectum | PFHXA | 0.98 [0.92, 1.04] | 0.49 |
| Anus Anal Canal and Anorectum | PFHXA | 1.1 [0.96, 1.26] | 0.19 |
| Liver and Intrahepatic Bile Duct | PFHXA | 1.03 [0.96, 1.1] | 0.44 |
| Liver | PFHXA | 1.03 [0.96, 1.12] | 0.39 |
| Intrahepatic Bile Duct | PFHXA | 1.01 [0.87, 1.18] | 0.89 |
| Gallbladder | PFHXA | 0.99 [0.81, 1.19] | 0.89 |
| Other Biliary | PFHXA | 0.98 [0.85, 1.15] | 0.84 |
| Pancreas | PFHXA | 1.02 [0.97, 1.08] | 0.49 |
| Retroperitoneum | PFHXA | 0.92 [0.68, 1.26] | 0.60 |
| Peritoneum Omentum and Mesentery | PFHXA | 1 [0.73, 1.35] | 0.98 |
| Other Digestive Organs | PFHXA | 0.99 [0.79, 1.24] | 0.93 |
| **Respiratory System** | PFHXA | 1.02 [0.99, 1.05] | 0.24 |
| Nose Nasal Cavity and Middle Ear | PFHXA | 1.1 [0.87, 1.4] | 0.43 |
| Larynx | PFHXA | 0.96 [0.86, 1.07] | 0.46 |
| Lung and Bronchus | PFHXA | 1.02 [0.99, 1.06] | 0.20 |
| Pleura | PFHXA | 0.84 [0.24, 2.88] | 0.78 |
| Trachea Mediastinum and Other Respiratory Organs | PFHXA | 1.02 [0.65, 1.59] | 0.94 |
| Bones and Joints | PFHXA | 0.98 [0.81, 1.2] | 0.85 |
| Soft Tissue including Heart | PFHXA | 1.01 [0.91, 1.13] | 0.80 |
| Skin excluding Basal and Squamous | PFHXA | 1 [0.94, 1.06] | 0.88 |
| Melanoma of the Skin | PFHXA | 1.01 [0.94, 1.07] | 0.87 |
| Other Non Epithelial Skin | PFHXA | 0.88 [0.76, 1.02] | 0.09 |
| **Breast** | PFHXA | 0.99 [0.96, 1.01] | 0.30 |
| **Urinary System** | PFHXA | 0.97 [0.94, 1.01] | 0.11 |
| Urinary Bladder | PFHXA | 0.97 [0.92, 1.01] | 0.15 |
| Kidney and Renal Pelvis | PFHXA | 0.98 [0.94, 1.03] | 0.40 |
| Ureter | PFHXA | 1.06 [0.81, 1.4] | 0.67 |
| Other Urinary Organs | PFHXA | 0.91 [0.67, 1.24] | 0.55 |
| **Brain and Other Nervous System** | PFHXA | 0.98 [0.91, 1.06] | 0.70 |
| Brain | PFHXA | 0.98 [0.91, 1.07] | 0.68 |
| Cranial Nerves Other Nervous System | PFHXA | 1 [0.73, 1.37] | 0.99 |
| **Endocrine System** | PFHXA | 0.96 [0.9, 1.02] | 0.20 |
| Thyroid | PFHXA | 0.96 [0.9, 1.02] | 0.20 |
| Other Endocrine including Thymus | PFHXA | 1 [0.81, 1.23] | 0.97 |
| **Lymphoma** | PFHXA | 0.99 [0.95, 1.04] | 0.81 |
| **Hodgkin Lymphoma** | PFHXA | 1.03 [0.91, 1.17] | 0.63 |
| Hodgkin Nodal | PFHXA | 1.04 [0.92, 1.17] | 0.58 |
| Hodgkin Extranodal | PFHXA | 0.75 [0.26, 2.18] | 0.60 |
| Non Hodgkin Lymphoma | PFHXA | 0.99 [0.95, 1.04] | 0.67 |
| NHL Nodal | PFHXA | 0.99 [0.93, 1.04] | 0.67 |
| NHL Extranodal | PFHXA | 0.99 [0.92, 1.08] | 0.89 |
| **Myeloma** | PFHXA | 1.02 [0.95, 1.1] | 0.64 |
| **Leukemia** | PFHXA | 0.99 [0.94, 1.04] | 0.75 |
| Lymphocytic Leukemia | PFHXA | 0.99 [0.92, 1.07] | 0.84 |
| Acute Lymphocytic Leukemia | PFHXA | 0.97 [0.83, 1.13] | 0.70 |
| Chronic Lymphocytic Leukemia | PFHXA | 1.01 [0.92, 1.1] | 0.87 |
| Other Lymphocytic Leukemia | PFHXA | 0.91 [0.66, 1.26] | 0.58 |
| Myeloid and Monocytic Leukemia | PFHXA | 0.96 [0.89, 1.03] | 0.27 |
| Acute Myeloid Leukemia | PFHXA | 0.92 [0.84, 1.01] | 0.10 |
| Acute Monocytic Leukemia | PFHXA | 0.9 [0.56, 1.44] | 0.66 |
| Chronic Myeloid Leukemia | PFHXA | 1.01 [0.89, 1.16] | 0.83 |
| Other Myeloid Monocytic Leukemia | PFHXA | 1.4 [0.82, 2.41] | 0.22 |
| Other Leukemia | PFHXA | 1.27 [1.04, 1.55] | 0.02 |
| Other Acute Leukemia | PFHXA | 1.61 [1.1, 2.36] | 0.01 |
| Aleukemic Subleukemic and NOS | PFHXA | 1.16 [0.91, 1.48] | 0.23 |
| **All Sites** | PFPEA | 1 [0.98, 1.02] | 0.87 |
| Oral Cavity and Pharynx | PFPEA | 1.04 [0.98, 1.09] | 0.19 |
| Lip | PFPEA | 0.96 [0.76, 1.22] | 0.74 |
| Tongue | PFPEA | 1.03 [0.94, 1.13] | 0.51 |
| Salivary Gland | PFPEA | 1.03 [0.88, 1.21] | 0.70 |
| Floor of Mouth | PFPEA | 0.95 [0.73, 1.25] | 0.73 |
| Gum and Other Mouth | PFPEA | 1.03 [0.89, 1.19] | 0.70 |
| Nasopharynx | PFPEA | 1 [0.76, 1.32] | 1.00 |
| Tonsil | PFPEA | 1.09 [0.97, 1.23] | 0.15 |
| Oropharynx | PFPEA | 1.06 [0.85, 1.31] | 0.62 |
| Hypopharynx | PFPEA | 0.94 [0.73, 1.21] | 0.65 |
| Other Oral Cavity and Pharynx | PFPEA | 1.31 [0.92, 1.88] | 0.14 |
| **Digestive System** | PFPEA | 1.01 [0.98, 1.03] | 0.49 |
| Esophagus | PFPEA | 0.98 [0.9, 1.07] | 0.64 |
| Stomach | PFPEA | 1.01 [0.93, 1.09] | 0.88 |
| Small Intestine | PFPEA | 0.97 [0.86, 1.09] | 0.58 |
| Colon and Rectum | PFPEA | 1.01 [0.97, 1.04] | 0.71 |
| Colon excluding Rectum | PFPEA | 1.01 [0.97, 1.05] | 0.56 |
| Cecum | PFPEA | 1.03 [0.95, 1.12] | 0.43 |
| Appendix | PFPEA | 0.98 [0.85, 1.12] | 0.77 |
| Ascending Colon | PFPEA | 0.97 [0.9, 1.06] | 0.54 |
| Hepatic Flexure | PFPEA | 0.98 [0.83, 1.16] | 0.81 |
| Transverse Colon | PFPEA | 1.05 [0.94, 1.18] | 0.41 |
| Splenic Flexure | PFPEA | 1 [0.81, 1.24] | 0.99 |
| Descending Colon | PFPEA | 0.96 [0.83, 1.11] | 0.59 |
| Sigmoid Colon | PFPEA | 0.98 [0.92, 1.06] | 0.65 |
| Large Intestine NOS | PFPEA | 1.2 [1.06, 1.36] | 0.01 |
| Rectum and Rectosigmoid Junction | PFPEA | 1 [0.94, 1.05] | 0.89 |
| Rectosigmoid Junction | PFPEA | 1.03 [0.91, 1.16] | 0.63 |
| Rectum | PFPEA | 0.99 [0.93, 1.05] | 0.71 |
| Anus Anal Canal and Anorectum | PFPEA | 1.04 [0.91, 1.19] | 0.56 |
| Liver and Intrahepatic Bile Duct | PFPEA | 1.05 [0.98, 1.12] | 0.14 |
| Liver | PFPEA | 1.07 [1, 1.16] | 0.06 |
| Intrahepatic Bile Duct | PFPEA | 0.96 [0.83, 1.11] | 0.58 |
| Gallbladder | PFPEA | 0.97 [0.81, 1.17] | 0.75 |
| Other Biliary | PFPEA | 1.01 [0.87, 1.17] | 0.92 |
| Pancreas | PFPEA | 1.01 [0.96, 1.07] | 0.64 |
| Retroperitoneum | PFPEA | 1.06 [0.79, 1.42] | 0.71 |
| Peritoneum Omentum and Mesentery | PFPEA | 0.99 [0.74, 1.33] | 0.94 |
| Other Digestive Organs | PFPEA | 0.98 [0.79, 1.22] | 0.87 |
| **Respiratory System** | PFPEA | 1.03 [1, 1.06] | 0.10 |
| Nose Nasal Cavity and Middle Ear | PFPEA | 1.1 [0.88, 1.39] | 0.39 |
| Larynx | PFPEA | 0.96 [0.87, 1.07] | 0.48 |
| Lung and Bronchus | PFPEA | 1.03 [1, 1.06] | 0.07 |
| Pleura | PFPEA | 1 [0.33, 3.04] | 0.99 |
| Trachea Mediastinum and Other Respiratory Organs | PFPEA | 0.84 [0.54, 1.31] | 0.44 |
| Bones and Joints | PFPEA | 0.97 [0.8, 1.17] | 0.74 |
| Soft Tissue including Heart | PFPEA | 1.04 [0.94, 1.16] | 0.42 |
| Skin excluding Basal and Squamous | PFPEA | 0.99 [0.94, 1.05] | 0.82 |
| Melanoma of the Skin | PFPEA | 1.01 [0.95, 1.07] | 0.86 |
| Other Non Epithelial Skin | PFPEA | 0.85 [0.74, 0.98] | 0.02 |
| **Breast** | PFPEA | 1 [0.97, 1.02] | 0.90 |
| **Urinary System** | PFPEA | 0.97 [0.95, 1] | 0.09 |
| Urinary Bladder | PFPEA | 0.95 [0.91, 0.99] | 0.02 |
| Kidney and Renal Pelvis | PFPEA | 1 [0.96, 1.04] | 0.96 |
| Ureter | PFPEA | 0.99 [0.76, 1.29] | 0.95 |
| Other Urinary Organs | PFPEA | 0.93 [0.7, 1.25] | 0.65 |
| **Brain and Other Nervous System** | PFPEA | 1.01 [0.94, 1.09] | 0.81 |
| Brain | PFPEA | 1 [0.93, 1.08] | 0.94 |
| Cranial Nerves Other Nervous System | PFPEA | 1.1 [0.82, 1.49] | 0.53 |
| **Endocrine System** | PFPEA | 0.96 [0.91, 1.02] | 0.16 |
| Thyroid | PFPEA | 0.95 [0.9, 1.01] | 0.13 |
| Other Endocrine including Thymus | PFPEA | 1.03 [0.85, 1.26] | 0.74 |
| **Lymphoma** | PFPEA | 1.01 [0.97, 1.05] | 0.74 |
| **Hodgkin Lymphoma** | PFPEA | 1.08 [0.96, 1.21] | 0.20 |
| Hodgkin Nodal | PFPEA | 1.08 [0.96, 1.22] | 0.20 |
| Hodgkin Extranodal | PFPEA | 0.96 [0.36, 2.54] | 0.93 |
| Non Hodgkin Lymphoma | PFPEA | 1 [0.95, 1.04] | 0.91 |
| NHL Nodal | PFPEA | 1.02 [0.96, 1.07] | 0.52 |
| NHL Extranodal | PFPEA | 0.96 [0.88, 1.03] | 0.25 |
| **Myeloma** | PFPEA | 0.99 [0.92, 1.06] | 0.79 |
| **Leukemia** | PFPEA | 0.98 [0.93, 1.03] | 0.45 |
| Lymphocytic Leukemia | PFPEA | 0.96 [0.89, 1.03] | 0.24 |
| Acute Lymphocytic Leukemia | PFPEA | 0.93 [0.81, 1.08] | 0.37 |
| Chronic Lymphocytic Leukemia | PFPEA | 0.98 [0.9, 1.06] | 0.57 |
| Other Lymphocytic Leukemia | PFPEA | 0.86 [0.63, 1.17] | 0.33 |
| Myeloid and Monocytic Leukemia | PFPEA | 0.97 [0.91, 1.05] | 0.48 |
| Acute Myeloid Leukemia | PFPEA | 0.94 [0.85, 1.03] | 0.17 |
| Acute Monocytic Leukemia | PFPEA | 0.99 [0.64, 1.55] | 0.98 |
| Chronic Myeloid Leukemia | PFPEA | 1.03 [0.91, 1.18] | 0.62 |
| Other Myeloid Monocytic Leukemia | PFPEA | 1.28 [0.75, 2.17] | 0.36 |
| Other Leukemia | PFPEA | 1.25 [1.03, 1.52] | 0.02 |
| Other Acute Leukemia | PFPEA | 1.56 [1.09, 2.26] | 0.02 |
| Aleukemic Subleukemic and NOS | PFPEA | 1.15 [0.91, 1.45] | 0.24 |
| 1. All models were adjusted for county-level SES variables, urbanicity, smoking rate, obesity, and air pollution. | | | |
| 2. cells were highlighted if crude p values were less than 0.05. | | | |
