# Supplementary material for: Associations between per-and polyfluoroalkyl substances (PFAS) and county-level cancer incidence between 2016 and 2021 and incident cancer burden attributable to PFAS in drinking water in the United States
Source: J Expo Sci Environ Epidemiol. 2025 Jan 9;35(3):425–36. doi: 10.1038/s41370-024-00742-2 (PMC12069088; doi:10.1038/s41370-024-00742-2)
Supplement: Supplementary file 4 — Supplemental Table 3 [file 41370_2024_742_MOESM4_ESM.docx]

| **Supplemental Table 11.** Sensitivity analysis for the association between detection/MCL violation of at least one PFAS in drinking water based on UCMR5 and cancer incidence. | | |
| --- | --- | --- |
| **Cancers** | **IRR [95% CI]** | **p value^1,2^** |
| **All Sites** | 1 [0.99, 1.02] | 0.75 |
| Oral Cavity and Pharynx | 1.05 [1, 1.1] | 0.05 |
| Lip | 0.83 [0.68, 1.02] | 0.08 |
| Tongue | 1.08 [0.99, 1.17] | 0.07 |
| Salivary Gland | 1.11 [0.96, 1.29] | 0.15 |
| Floor of Mouth | 0.97 [0.77, 1.23] | 0.82 |
| Gum and Other Mouth | 1.03 [0.9, 1.16] | 0.69 |
| Nasopharynx | 1.03 [0.8, 1.32] | 0.84 |
| Tonsil | 1.08 [0.97, 1.2] | 0.15 |
| Oropharynx | 0.99 [0.82, 1.19] | 0.89 |
| Hypopharynx | 0.96 [0.77, 1.2] | 0.72 |
| Other Oral Cavity and Pharynx | 1.36 [0.99, 1.88] | 0.06 |
| **Digestive System** | 1.02 [1, 1.04] | 0.13 |
| Esophagus | 1.01 [0.94, 1.09] | 0.74 |
| Stomach | 1.03 [0.96, 1.1] | 0.44 |
| Small Intestine | 0.99 [0.89, 1.1] | 0.88 |
| Colon and Rectum | 1.01 [0.98, 1.04] | 0.39 |
| Colon excluding Rectum | 1.01 [0.98, 1.05] | 0.42 |
| Cecum | 1.01 [0.94, 1.09] | 0.72 |
| Appendix | 1 [0.88, 1.13] | 0.95 |
| Ascending Colon | 0.98 [0.91, 1.05] | 0.54 |
| Hepatic Flexure | 1.02 [0.88, 1.18] | 0.82 |
| Transverse Colon | 1.03 [0.93, 1.14] | 0.59 |
| Splenic Flexure | 1.08 [0.9, 1.31] | 0.40 |
| Descending Colon | 0.91 [0.8, 1.04] | 0.17 |
| Sigmoid Colon | 1.02 [0.96, 1.09] | 0.50 |
| Large Intestine NOS | 1.12 [1, 1.26] | 0.04 |
| Rectum and Rectosigmoid Junction | 1.01 [0.96, 1.06] | 0.64 |
| Rectosigmoid Junction | 1.04 [0.93, 1.15] | 0.51 |
| Rectum | 1.01 [0.95, 1.06] | 0.83 |
| Anus Anal Canal and Anorectum | 1.05 [0.93, 1.18] | 0.42 |
| Liver and Intrahepatic Bile Duct | 1.07 [1.01, 1.13] | 0.03 |
| Liver | 1.09 [1.02, 1.17] | 0.01 |
| Intrahepatic Bile Duct | 0.98 [0.86, 1.12] | 0.79 |
| Gallbladder | 1.04 [0.88, 1.23] | 0.64 |
| Other Biliary | 1.03 [0.9, 1.17] | 0.70 |
| Pancreas | 1 [0.95, 1.05] | 0.94 |
| Retroperitoneum | 1.05 [0.8, 1.37] | 0.74 |
| Peritoneum Omentum and Mesentery | 0.92 [0.71, 1.2] | 0.55 |
| Other Digestive Organs | 0.95 [0.79, 1.16] | 0.64 |
| **Respiratory System** | 1.03 [1.01, 1.06] | 0.02 |
| Nose Nasal Cavity and Middle Ear | 1.03 [0.84, 1.26] | 0.80 |
| Larynx | 0.99 [0.9, 1.07] | 0.74 |
| Lung and Bronchus | 1.04 [1.01, 1.06] | 0.01 |
| Pleura | 0.62 [0.23, 1.65] | 0.34 |
| Trachea Mediastinum and Other Respiratory Organs | 0.83 [0.56, 1.21] | 0.33 |
| Bones and Joints | 0.93 [0.78, 1.11] | 0.42 |
| Soft Tissue including Heart | 1 [0.91, 1.09] | 0.92 |
| Skin excluding Basal and Squamous | 0.99 [0.94, 1.04] | 0.58 |
| Melanoma of the Skin | 1 [0.94, 1.05] | 0.88 |
| Other Non Epithelial Skin | 0.87 [0.77, 0.98] | 0.02 |
| **Breast** | 0.99 [0.97, 1.01] | 0.46 |
| **Urinary System** | 1 [0.97, 1.03] | 0.95 |
| Urinary Bladder | 0.99 [0.95, 1.03] | 0.55 |
| Kidney and Renal Pelvis | 1.02 [0.98, 1.05] | 0.43 |
| Ureter | 0.97 [0.77, 1.23] | 0.83 |
| Other Urinary Organs | 0.89 [0.69, 1.15] | 0.38 |
| **Brain and Other Nervous System** | 0.99 [0.93, 1.06] | 0.80 |
| Brain | 0.97 [0.91, 1.04] | 0.39 |
| Cranial Nerves Other Nervous System | 1.29 [0.99, 1.68] | 0.06 |
| **Endocrine System** | 0.96 [0.91, 1.01] | 0.11 |
| Thyroid | 0.95 [0.9, 1] | 0.06 |
| Other Endocrine including Thymus | 1.1 [0.92, 1.32] | 0.31 |
| **Lymphoma** | 1.01 [0.97, 1.05] | 0.60 |
| **Hodgkin Lymphoma** | 1.04 [0.93, 1.15] | 0.48 |
| Hodgkin Nodal | 1.04 [0.94, 1.16] | 0.47 |
| Hodgkin Extranodal | 0.96 [0.42, 2.2] | 0.93 |
| Non Hodgkin Lymphoma | 1.01 [0.97, 1.05] | 0.77 |
| NHL Nodal | 1.01 [0.96, 1.06] | 0.71 |
| NHL Extranodal | 1 [0.93, 1.07] | 0.97 |
| **Myeloma** | 1.01 [0.95, 1.08] | 0.78 |
| **Leukemia** | 0.97 [0.93, 1.01] | 0.16 |
| Lymphocytic Leukemia | 0.99 [0.93, 1.05] | 0.72 |
| Acute Lymphocytic Leukemia | 0.93 [0.81, 1.06] | 0.26 |
| Chronic Lymphocytic Leukemia | 1 [0.93, 1.08] | 0.91 |
| Other Lymphocytic Leukemia | 1.04 [0.79, 1.37] | 0.77 |
| Myeloid and Monocytic Leukemia | 0.93 [0.87, 0.99] | 0.02 |
| Acute Myeloid Leukemia | 0.91 [0.84, 0.99] | 0.02 |
| Acute Monocytic Leukemia | 0.74 [0.5, 1.1] | 0.13 |
| Chronic Myeloid Leukemia | 0.97 [0.87, 1.09] | 0.62 |
| Other Myeloid Monocytic Leukemia | 1.19 [0.74, 1.92] | 0.47 |
| Other Leukemia | 1.14 [0.96, 1.36] | 0.15 |
| Other Acute Leukemia | 1.29 [0.92, 1.8] | 0.14 |
| Aleukemic Subleukemic and NOS | 1.09 [0.88, 1.34] | 0.43 |
| 1. All models were adjusted for county-level SES variables, urbanicity, smoking rate, obesity, and air pollution. | | |
| 2. cells were highlighted if crude p values were less than 0.05. | | |

| **Supplemental Table 12.** Sensitivity analysis of detection/MCL violation of PFAS based on both UCMR3 and 5 and cancer incidence. | | | |
| --- | --- | --- | --- |
| **Cancers** | **Exposures** | **IRR [95% CI]** | **p value^1,2,3^** |
| **All Sites** | PFOA | 1.04 [1, 1.08] | 0.05 |
| Oral Cavity and Pharynx | PFOA | 0.99 [0.87, 1.14] | 0.93 |
| Lip | PFOA | 0.91 [0.46, 1.84] | 0.80 |
| Tongue | PFOA | 1.01 [0.8, 1.28] | 0.93 |
| Salivary Gland | PFOA | 1.06 [0.7, 1.6] | 0.77 |
| Floor of Mouth | PFOA | 0.79 [0.36, 1.74] | 0.56 |
| Gum and Other Mouth | PFOA | 1 [0.69, 1.45] | 0.99 |
| Nasopharynx | PFOA | 1 [0.52, 1.93] | 1.00 |
| Tonsil | PFOA | 0.98 [0.71, 1.35] | 0.89 |
| Oropharynx | PFOA | 1.01 [0.58, 1.78] | 0.97 |
| Hypopharynx | PFOA | 0.95 [0.48, 1.87] | 0.87 |
| Other Oral Cavity and Pharynx | PFOA | 1.06 [0.39, 2.85] | 0.91 |
| **Digestive System** | PFOA | 1.05 [0.99, 1.11] | 0.08 |
| Esophagus | PFOA | 0.99 [0.79, 1.24] | 0.94 |
| Stomach | PFOA | 1.28 [1.08, 1.52] | 0.00 |
| Small Intestine | PFOA | 1.06 [0.79, 1.41] | 0.70 |
| Colon and Rectum | PFOA | 1.06 [0.99, 1.15] | 0.11 |
| Colon excluding Rectum | PFOA | 1.07 [0.98, 1.17] | 0.15 |
| Cecum | PFOA | 1.03 [0.83, 1.27] | 0.81 |
| Appendix | PFOA | 1.1 [0.78, 1.54] | 0.59 |
| Ascending Colon | PFOA | 1.05 [0.85, 1.3] | 0.63 |
| Hepatic Flexure | PFOA | 1.28 [0.85, 1.93] | 0.24 |
| Transverse Colon | PFOA | 1.08 [0.8, 1.47] | 0.60 |
| Splenic Flexure | PFOA | 1.13 [0.66, 1.94] | 0.66 |
| Descending Colon | PFOA | 1.08 [0.74, 1.58] | 0.67 |
| Sigmoid Colon | PFOA | 1.07 [0.89, 1.28] | 0.50 |
| Large Intestine NOS | PFOA | 1.03 [0.74, 1.44] | 0.85 |
| Rectum and Rectosigmoid Junction | PFOA | 1.05 [0.92, 1.21] | 0.46 |
| Rectosigmoid Junction | PFOA | 1.1 [0.82, 1.48] | 0.53 |
| Rectum | PFOA | 1.04 [0.89, 1.22] | 0.62 |
| Anus Anal Canal and Anorectum | PFOA | 0.9 [0.63, 1.29] | 0.57 |
| Liver and Intrahepatic Bile Duct | PFOA | 0.94 [0.8, 1.11] | 0.46 |
| Liver | PFOA | 0.92 [0.76, 1.11] | 0.38 |
| Intrahepatic Bile Duct | PFOA | 1.03 [0.71, 1.49] | 0.87 |
| Gallbladder | PFOA | 1.05 [0.67, 1.63] | 0.84 |
| Other Biliary | PFOA | 1.03 [0.72, 1.47] | 0.89 |
| Pancreas | PFOA | 1.04 [0.92, 1.18] | 0.53 |
| Retroperitoneum | PFOA | 0.96 [0.44, 2.06] | 0.91 |
| Peritoneum Omentum and Mesentery | PFOA | 0.88 [0.4, 1.92] | 0.75 |
| Other Digestive Organs | PFOA | 0.92 [0.53, 1.58] | 0.76 |
| **Respiratory System** | PFOA | 1.07 [0.99, 1.15] | 0.09 |
| Nose Nasal Cavity and Middle Ear | PFOA | 1 [0.55, 1.81] | 1.00 |
| Larynx | PFOA | 1.05 [0.79, 1.38] | 0.75 |
| Lung and Bronchus | PFOA | 1.07 [0.99, 1.15] | 0.09 |
| Pleura | PFOA | 1.05 [0.05, 23.78] | 0.98 |
| Trachea Mediastinum and Other Respiratory Organs | PFOA | 1.08 [0.35, 3.31] | 0.90 |
| Bones and Joints | PFOA | 1.06 [0.66, 1.71] | 0.80 |
| Soft Tissue including Heart | PFOA | 1.1 [0.86, 1.42] | 0.45 |
| Skin excluding Basal and Squamous | PFOA | 0.85 [0.73, 0.99] | 0.03 |
| Melanoma of the Skin | PFOA | 0.85 [0.73, 0.99] | 0.04 |
| Other Non Epithelial Skin | PFOA | 0.87 [0.6, 1.26] | 0.45 |
| **Breast** | PFOA | 1.04 [0.98, 1.1] | 0.19 |
| **Urinary System** | PFOA | 1.01 [0.94, 1.09] | 0.75 |
| Urinary Bladder | PFOA | 1.08 [0.97, 1.2] | 0.14 |
| Kidney and Renal Pelvis | PFOA | 0.94 [0.83, 1.05] | 0.26 |
| Ureter | PFOA | 1.31 [0.71, 2.4] | 0.38 |
| Other Urinary Organs | PFOA | 0.93 [0.43, 2.03] | 0.86 |
| **Brain and Other Nervous System** | PFOA | 1.07 [0.89, 1.28] | 0.50 |
| Brain | PFOA | 1.1 [0.91, 1.33] | 0.31 |
| Cranial Nerves Other Nervous System | PFOA | 0.55 [0.2, 1.48] | 0.24 |
| **Endocrine System** | PFOA | 1.17 [1.04, 1.33] | 0.01 |
| Thyroid | PFOA | 1.18 [1.04, 1.34] | 0.01 |
| Other Endocrine including Thymus | PFOA | 1.11 [0.69, 1.78] | 0.67 |
| **Lymphoma** | PFOA | 1.05 [0.94, 1.16] | 0.39 |
| **Hodgkin Lymphoma** | PFOA | 1.09 [0.82, 1.46] | 0.55 |
| Hodgkin Nodal | PFOA | 1.1 [0.82, 1.47] | 0.52 |
| Hodgkin Extranodal | PFOA | 0.83 [0.05, 13.4] | 0.89 |
| Non Hodgkin Lymphoma | PFOA | 1.04 [0.93, 1.16] | 0.49 |
| NHL Nodal | PFOA | 1 [0.87, 1.14] | 0.96 |
| NHL Extranodal | PFOA | 1.13 [0.94, 1.35] | 0.20 |
| **Myeloma** | PFOA | 0.96 [0.81, 1.15] | 0.68 |
| **Leukemia** | PFOA | 1.06 [0.94, 1.2] | 0.35 |
| Lymphocytic Leukemia | PFOA | 1.07 [0.9, 1.27] | 0.47 |
| Acute Lymphocytic Leukemia | PFOA | 1.09 [0.77, 1.56] | 0.62 |
| Chronic Lymphocytic Leukemia | PFOA | 1.06 [0.86, 1.3] | 0.57 |
| Other Lymphocytic Leukemia | PFOA | 1.1 [0.53, 2.29] | 0.79 |
| Myeloid and Monocytic Leukemia | PFOA | 1.09 [0.91, 1.3] | 0.37 |
| Acute Myeloid Leukemia | PFOA | 1.04 [0.83, 1.31] | 0.71 |
| Acute Monocytic Leukemia | PFOA | 1.6 [0.61, 4.18] | 0.34 |
| Chronic Myeloid Leukemia | PFOA | 1.09 [0.79, 1.52] | 0.59 |
| Other Myeloid Monocytic Leukemia | PFOA | 1.8 [0.59, 5.51] | 0.30 |
| Other Leukemia | PFOA | 0.8 [0.45, 1.42] | 0.45 |
| Other Acute Leukemia | PFOA | 0.78 [0.26, 2.36] | 0.66 |
| Aleukemic Subleukemic and NOS | PFOA | 0.82 [0.42, 1.59] | 0.55 |
| **All Sites** | PFOS | 1.01 [0.96, 1.07] | 0.66 |
| Oral Cavity and Pharynx | PFOS | 1 [0.83, 1.21] | 0.98 |
| Lip | PFOS | 1.14 [0.49, 2.63] | 0.76 |
| Tongue | PFOS | 0.98 [0.71, 1.37] | 0.92 |
| Salivary Gland | PFOS | 1.16 [0.69, 1.95] | 0.57 |
| Floor of Mouth | PFOS | 0.83 [0.29, 2.41] | 0.74 |
| Gum and Other Mouth | PFOS | 1.08 [0.67, 1.76] | 0.75 |
| Nasopharynx | PFOS | 0.7 [0.25, 1.99] | 0.50 |
| Tonsil | PFOS | 0.94 [0.61, 1.47] | 0.80 |
| Oropharynx | PFOS | 1.05 [0.5, 2.21] | 0.91 |
| Hypopharynx | PFOS | 1.07 [0.45, 2.54] | 0.87 |
| Other Oral Cavity and Pharynx | PFOS | 0.9 [0.22, 3.73] | 0.89 |
| **Digestive System** | PFOS | 1.03 [0.96, 1.11] | 0.38 |
| Esophagus | PFOS | 1.13 [0.85, 1.51] | 0.39 |
| Stomach | PFOS | 1.04 [0.82, 1.32] | 0.75 |
| Small Intestine | PFOS | 1.08 [0.73, 1.59] | 0.70 |
| Colon and Rectum | PFOS | 1.03 [0.93, 1.15] | 0.52 |
| Colon excluding Rectum | PFOS | 1.02 [0.9, 1.16] | 0.71 |
| Cecum | PFOS | 0.95 [0.71, 1.28] | 0.74 |
| Appendix | PFOS | 1.07 [0.67, 1.7] | 0.78 |
| Ascending Colon | PFOS | 1.07 [0.81, 1.41] | 0.65 |
| Hepatic Flexure | PFOS | 0.99 [0.54, 1.83] | 0.98 |
| Transverse Colon | PFOS | 1.25 [0.86, 1.81] | 0.25 |
| Splenic Flexure | PFOS | 0.85 [0.38, 1.94] | 0.70 |
| Descending Colon | PFOS | 1.01 [0.6, 1.7] | 0.98 |
| Sigmoid Colon | PFOS | 1.03 [0.8, 1.31] | 0.83 |
| Large Intestine NOS | PFOS | 0.89 [0.56, 1.42] | 0.62 |
| Rectum and Rectosigmoid Junction | PFOS | 1.06 [0.88, 1.27] | 0.55 |
| Rectosigmoid Junction | PFOS | 1.27 [0.88, 1.83] | 0.21 |
| Rectum | PFOS | 1 [0.81, 1.24] | 0.98 |
| Anus Anal Canal and Anorectum | PFOS | 0.99 [0.62, 1.58] | 0.97 |
| Liver and Intrahepatic Bile Duct | PFOS | 0.96 [0.78, 1.19] | 0.72 |
| Liver | PFOS | 0.96 [0.76, 1.22] | 0.76 |
| Intrahepatic Bile Duct | PFOS | 0.96 [0.58, 1.59] | 0.88 |
| Gallbladder | PFOS | 1.17 [0.68, 2.02] | 0.57 |
| Other Biliary | PFOS | 1.21 [0.78, 1.87] | 0.39 |
| Pancreas | PFOS | 1.02 [0.86, 1.21] | 0.83 |
| Retroperitoneum | PFOS | 1.05 [0.4, 2.75] | 0.92 |
| Peritoneum Omentum and Mesentery | PFOS | 1.29 [0.53, 3.09] | 0.57 |
| Other Digestive Organs | PFOS | 0.84 [0.4, 1.79] | 0.66 |
| **Respiratory System** | PFOS | 0.99 [0.89, 1.1] | 0.87 |
| Nose Nasal Cavity and Middle Ear | PFOS | 0.8 [0.33, 1.93] | 0.62 |
| Larynx | PFOS | 0.98 [0.66, 1.45] | 0.92 |
| Lung and Bronchus | PFOS | 0.99 [0.89, 1.1] | 0.89 |
| Pleura | PFOS | NA | NA |
| Trachea Mediastinum and Other Respiratory Organs | PFOS | 1.27 [0.32, 4.98] | 0.73 |
| Bones and Joints | PFOS | 1 [0.53, 1.92] | 0.99 |
| Soft Tissue including Heart | PFOS | 0.95 [0.67, 1.36] | 0.80 |
| Skin excluding Basal and Squamous | PFOS | 0.99 [0.81, 1.2] | 0.90 |
| Melanoma of the Skin | PFOS | 1 [0.82, 1.22] | 0.99 |
| Other Non Epithelial Skin | PFOS | 0.87 [0.53, 1.43] | 0.57 |
| **Breast** | PFOS | 1.01 [0.93, 1.09] | 0.89 |
| **Urinary System** | PFOS | 1 [0.9, 1.11] | 0.95 |
| Urinary Bladder | PFOS | 1.06 [0.92, 1.23] | 0.41 |
| Kidney and Renal Pelvis | PFOS | 0.96 [0.82, 1.11] | 0.57 |
| Ureter | PFOS | 0.91 [0.34, 2.4] | 0.85 |
| Other Urinary Organs | PFOS | 0.72 [0.22, 2.38] | 0.59 |
| **Brain and Other Nervous System** | PFOS | 1.04 [0.81, 1.33] | 0.77 |
| Brain | PFOS | 1.02 [0.79, 1.32] | 0.86 |
| Cranial Nerves Other Nervous System | PFOS | 1.23 [0.49, 3.08] | 0.65 |
| **Endocrine System** | PFOS | 1.02 [0.86, 1.22] | 0.82 |
| Thyroid | PFOS | 1.02 [0.85, 1.23] | 0.81 |
| Other Endocrine including Thymus | PFOS | 0.99 [0.51, 1.91] | 0.97 |
| **Lymphoma** | PFOS | 1.08 [0.94, 1.23] | 0.27 |
| **Hodgkin Lymphoma** | PFOS | 1.08 [0.73, 1.58] | 0.71 |
| Hodgkin Nodal | PFOS | 1.08 [0.73, 1.59] | 0.69 |
| Hodgkin Extranodal | PFOS | NA | NA |
| Non Hodgkin Lymphoma | PFOS | 1.08 [0.94, 1.24] | 0.30 |
| NHL Nodal | PFOS | 1.03 [0.86, 1.23] | 0.73 |
| NHL Extranodal | PFOS | 1.17 [0.92, 1.49] | 0.19 |
| **Myeloma** | PFOS | 0.93 [0.73, 1.19] | 0.57 |
| **Leukemia** | PFOS | 1.07 [0.91, 1.25] | 0.43 |
| Lymphocytic Leukemia | PFOS | 1.03 [0.82, 1.31] | 0.78 |
| Acute Lymphocytic Leukemia | PFOS | 0.75 [0.44, 1.3] | 0.31 |
| Chronic Lymphocytic Leukemia | PFOS | 1.13 [0.86, 1.48] | 0.38 |
| Other Lymphocytic Leukemia | PFOS | 1.12 [0.43, 2.92] | 0.82 |
| Myeloid and Monocytic Leukemia | PFOS | 1.1 [0.86, 1.4] | 0.45 |
| Acute Myeloid Leukemia | PFOS | 1.08 [0.8, 1.46] | 0.63 |
| Acute Monocytic Leukemia | PFOS | 1.17 [0.28, 4.94] | 0.83 |
| Chronic Myeloid Leukemia | PFOS | 1.12 [0.73, 1.72] | 0.60 |
| Other Myeloid Monocytic Leukemia | PFOS | 1.36 [0.28, 6.57] | 0.71 |
| Other Leukemia | PFOS | 1.13 [0.59, 2.14] | 0.72 |
| Other Acute Leukemia | PFOS | 1.05 [0.3, 3.71] | 0.94 |
| Aleukemic Subleukemic and NOS | PFOS | 1.15 [0.54, 2.43] | 0.72 |
| **All Sites** | PFHxS | 1.04 [0.94, 1.14] | 0.47 |
| Oral Cavity and Pharynx | PFHxS | 1.25 [0.93, 1.68] | 0.14 |
| Lip | PFHxS | 1.55 [0.46, 5.22] | 0.48 |
| Tongue | PFHxS | 1.07 [0.6, 1.91] | 0.81 |
| Salivary Gland | PFHxS | 1.87 [0.88, 3.96] | 0.10 |
| Floor of Mouth | PFHxS | 1.06 [0.2, 5.58] | 0.95 |
| Gum and Other Mouth | PFHxS | 1.45 [0.68, 3.09] | 0.34 |
| Nasopharynx | PFHxS | 0.42 [0.04, 4.96] | 0.49 |
| Tonsil | PFHxS | 1.18 [0.59, 2.37] | 0.65 |
| Oropharynx | PFHxS | 1.56 [0.51, 4.82] | 0.44 |
| Hypopharynx | PFHxS | 1.24 [0.32, 4.87] | 0.75 |
| Other Oral Cavity and Pharynx | PFHxS | 1.24 [0.17, 8.87] | 0.83 |
| **Digestive System** | PFHxS | 1.06 [0.94, 1.21] | 0.35 |
| Esophagus | PFHxS | 1.49 [0.95, 2.33] | 0.08 |
| Stomach | PFHxS | 0.82 [0.52, 1.31] | 0.41 |
| Small Intestine | PFHxS | 1.05 [0.51, 2.17] | 0.90 |
| Colon and Rectum | PFHxS | 1.03 [0.86, 1.23] | 0.74 |
| Colon excluding Rectum | PFHxS | 0.97 [0.78, 1.21] | 0.80 |
| Cecum | PFHxS | 0.84 [0.49, 1.45] | 0.53 |
| Appendix | PFHxS | 0.67 [0.22, 2.01] | 0.47 |
| Ascending Colon | PFHxS | 1.19 [0.75, 1.88] | 0.46 |
| Hepatic Flexure | PFHxS | 0.83 [0.27, 2.61] | 0.75 |
| Transverse Colon | PFHxS | 1.5 [0.84, 2.69] | 0.17 |
| Splenic Flexure | PFHxS | 0.52 [0.08, 3.18] | 0.48 |
| Descending Colon | PFHxS | 1.06 [0.44, 2.55] | 0.90 |
| Sigmoid Colon | PFHxS | 0.96 [0.62, 1.48] | 0.85 |
| Large Intestine NOS | PFHxS | 0.68 [0.29, 1.59] | 0.38 |
| Rectum and Rectosigmoid Junction | PFHxS | 1.16 [0.86, 1.57] | 0.32 |
| Rectosigmoid Junction | PFHxS | 1.5 [0.84, 2.67] | 0.17 |
| Rectum | PFHxS | 1.08 [0.76, 1.53] | 0.68 |
| Anus Anal Canal and Anorectum | PFHxS | 0.97 [0.42, 2.28] | 0.95 |
| Liver and Intrahepatic Bile Duct | PFHxS | 1.21 [0.87, 1.67] | 0.26 |
| Liver | PFHxS | 1.25 [0.87, 1.8] | 0.22 |
| Intrahepatic Bile Duct | PFHxS | 0.92 [0.34, 2.48] | 0.87 |
| Gallbladder | PFHxS | 1.34 [0.57, 3.16] | 0.51 |
| Other Biliary | PFHxS | 1.52 [0.74, 3.12] | 0.25 |
| Pancreas | PFHxS | 1.06 [0.78, 1.44] | 0.72 |
| Retroperitoneum | PFHxS | 0.8 [0.12, 5.59] | 0.82 |
| Peritoneum Omentum and Mesentery | PFHxS | 1.54 [0.3, 7.8] | 0.60 |
| Other Digestive Organs | PFHxS | 0.3 [0.03, 2.75] | 0.29 |
| **Respiratory System** | PFHxS | 1.04 [0.87, 1.23] | 0.70 |
| Nose Nasal Cavity and Middle Ear | PFHxS | 0.69 [0.13, 3.67] | 0.67 |
| Larynx | PFHxS | 0.82 [0.41, 1.63] | 0.57 |
| Lung and Bronchus | PFHxS | 1.05 [0.88, 1.26] | 0.60 |
| Pleura | PFHxS | NA | NA |
| Trachea Mediastinum and Other Respiratory Organs | PFHxS | 1.85 [0.29, 11.83] | 0.52 |
| Bones and Joints | PFHxS | 1.05 [0.33, 3.31] | 0.93 |
| Soft Tissue including Heart | PFHxS | 1.07 [0.57, 2] | 0.84 |
| Skin excluding Basal and Squamous | PFHxS | 1.22 [0.86, 1.72] | 0.27 |
| Melanoma of the Skin | PFHxS | 1.27 [0.89, 1.81] | 0.20 |
| Other Non Epithelial Skin | PFHxS | 0.67 [0.23, 1.98] | 0.47 |
| **Breast** | PFHxS | 1 [0.86, 1.16] | 0.96 |
| **Urinary System** | PFHxS | 1.06 [0.89, 1.27] | 0.50 |
| Urinary Bladder | PFHxS | 1.09 [0.83, 1.43] | 0.53 |
| Kidney and Renal Pelvis | PFHxS | 1.07 [0.84, 1.36] | 0.59 |
| Ureter | PFHxS | 0.71 [0.09, 5.25] | 0.73 |
| Other Urinary Organs | PFHxS | 0.27 [0.01, 8.95] | 0.47 |
| **Brain and Other Nervous System** | PFHxS | 1.19 [0.78, 1.8] | 0.43 |
| Brain | PFHxS | 1.1 [0.7, 1.73] | 0.68 |
| Cranial Nerves Other Nervous System | PFHxS | 2.33 [0.74, 7.33] | 0.15 |
| **Endocrine System** | PFHxS | 0.84 [0.59, 1.21] | 0.35 |
| Thyroid | PFHxS | 0.84 [0.57, 1.23] | 0.36 |
| Other Endocrine including Thymus | PFHxS | 0.9 [0.24, 3.38] | 0.87 |
| **Lymphoma** | PFHxS | 1.13 [0.88, 1.44] | 0.33 |
| **Hodgkin Lymphoma** | PFHxS | 0.73 [0.32, 1.7] | 0.47 |
| Hodgkin Nodal | PFHxS | 0.74 [0.32, 1.72] | 0.49 |
| Hodgkin Extranodal | PFHxS | NA | NA |
| Non Hodgkin Lymphoma | PFHxS | 1.19 [0.92, 1.53] | 0.19 |
| NHL Nodal | PFHxS | 1.13 [0.82, 1.55] | 0.45 |
| NHL Extranodal | PFHxS | 1.32 [0.86, 2.05] | 0.21 |
| **Myeloma** | PFHxS | 0.95 [0.62, 1.47] | 0.83 |
| **Leukemia** | PFHxS | 1.17 [0.89, 1.55] | 0.26 |
| Lymphocytic Leukemia | PFHxS | 1.09 [0.72, 1.65] | 0.68 |
| Acute Lymphocytic Leukemia | PFHxS | 0.33 [0.07, 1.44] | 0.14 |
| Chronic Lymphocytic Leukemia | PFHxS | 1.39 [0.89, 2.16] | 0.15 |
| Other Lymphocytic Leukemia | PFHxS | 0.94 [0.13, 6.76] | 0.95 |
| Myeloid and Monocytic Leukemia | PFHxS | 1.18 [0.78, 1.8] | 0.43 |
| Acute Myeloid Leukemia | PFHxS | 1.24 [0.74, 2.08] | 0.42 |
| Acute Monocytic Leukemia | PFHxS | NA | NA |
| Chronic Myeloid Leukemia | PFHxS | 1.21 [0.59, 2.52] | 0.60 |
| Other Myeloid Monocytic Leukemia | PFHxS | NA | NA |
| Other Leukemia | PFHxS | 1.66 [0.68, 4.04] | 0.26 |
| Other Acute Leukemia | PFHxS | 1.01 [0.13, 8.1] | 0.99 |
| Aleukemic Subleukemic and NOS | PFHxS | 1.92 [0.72, 5.13] | 0.19 |
| **All Sites** | PFHpA | 1.01 [0.97, 1.05] | 0.61 |
| Oral Cavity and Pharynx | PFHpA | 0.98 [0.86, 1.11] | 0.70 |
| Lip | PFHpA | 1.02 [0.53, 1.93] | 0.96 |
| Tongue | PFHpA | 0.91 [0.72, 1.14] | 0.41 |
| Salivary Gland | PFHpA | 1.12 [0.78, 1.61] | 0.53 |
| Floor of Mouth | PFHpA | 0.85 [0.42, 1.7] | 0.64 |
| Gum and Other Mouth | PFHpA | 1.04 [0.75, 1.46] | 0.80 |
| Nasopharynx | PFHpA | 0.95 [0.53, 1.7] | 0.85 |
| Tonsil | PFHpA | 0.98 [0.73, 1.32] | 0.91 |
| Oropharynx | PFHpA | 0.96 [0.58, 1.61] | 0.88 |
| Hypopharynx | PFHpA | 0.86 [0.46, 1.61] | 0.64 |
| Other Oral Cavity and Pharynx | PFHpA | 1.22 [0.51, 2.93] | 0.66 |
| **Digestive System** | PFHpA | 1.02 [0.97, 1.07] | 0.55 |
| Esophagus | PFHpA | 1.01 [0.82, 1.24] | 0.91 |
| Stomach | PFHpA | 1.05 [0.9, 1.23] | 0.54 |
| Small Intestine | PFHpA | 0.96 [0.73, 1.25] | 0.75 |
| Colon and Rectum | PFHpA | 1.05 [0.98, 1.13] | 0.16 |
| Colon excluding Rectum | PFHpA | 1.05 [0.96, 1.14] | 0.29 |
| Cecum | PFHpA | 1.03 [0.85, 1.25] | 0.78 |
| Appendix | PFHpA | 0.94 [0.67, 1.3] | 0.69 |
| Ascending Colon | PFHpA | 1.01 [0.83, 1.23] | 0.89 |
| Hepatic Flexure | PFHpA | 1.11 [0.73, 1.67] | 0.63 |
| Transverse Colon | PFHpA | 1.2 [0.92, 1.57] | 0.19 |
| Splenic Flexure | PFHpA | 0.93 [0.55, 1.57] | 0.78 |
| Descending Colon | PFHpA | 1.09 [0.78, 1.54] | 0.61 |
| Sigmoid Colon | PFHpA | 1.05 [0.89, 1.24] | 0.58 |
| Large Intestine NOS | PFHpA | 1.08 [0.8, 1.45] | 0.61 |
| Rectum and Rectosigmoid Junction | PFHpA | 1.06 [0.93, 1.2] | 0.36 |
| Rectosigmoid Junction | PFHpA | 1.14 [0.88, 1.48] | 0.32 |
| Rectum | PFHpA | 1.04 [0.9, 1.2] | 0.64 |
| Anus Anal Canal and Anorectum | PFHpA | 0.91 [0.66, 1.25] | 0.56 |
| Liver and Intrahepatic Bile Duct | PFHpA | 0.89 [0.77, 1.03] | 0.13 |
| Liver | PFHpA | 0.88 [0.75, 1.04] | 0.13 |
| Intrahepatic Bile Duct | PFHpA | 0.96 [0.69, 1.34] | 0.81 |
| Gallbladder | PFHpA | 0.96 [0.65, 1.41] | 0.84 |
| Other Biliary | PFHpA | 1.07 [0.79, 1.47] | 0.65 |
| Pancreas | PFHpA | 1.02 [0.91, 1.14] | 0.76 |
| Retroperitoneum | PFHpA | 0.89 [0.45, 1.76] | 0.73 |
| Peritoneum Omentum and Mesentery | PFHpA | 0.99 [0.52, 1.89] | 0.98 |
| Other Digestive Organs | PFHpA | 0.98 [0.61, 1.56] | 0.93 |
| **Respiratory System** | PFHpA | 1.03 [0.96, 1.1] | 0.46 |
| Nose Nasal Cavity and Middle Ear | PFHpA | 1.06 [0.63, 1.77] | 0.82 |
| Larynx | PFHpA | 0.96 [0.73, 1.25] | 0.74 |
| Lung and Bronchus | PFHpA | 1.03 [0.96, 1.1] | 0.43 |
| Pleura | PFHpA | NA | NA |
| Trachea Mediastinum and Other Respiratory Organs | PFHpA | 0.92 [0.31, 2.73] | 0.88 |
| Bones and Joints | PFHpA | 0.94 [0.61, 1.44] | 0.77 |
| Soft Tissue including Heart | PFHpA | 1.01 [0.8, 1.27] | 0.96 |
| Skin excluding Basal and Squamous | PFHpA | 0.91 [0.8, 1.04] | 0.17 |
| Melanoma of the Skin | PFHpA | 0.91 [0.79, 1.05] | 0.19 |
| Other Non Epithelial Skin | PFHpA | 0.9 [0.65, 1.25] | 0.55 |
| **Breast** | PFHpA | 0.99 [0.94, 1.04] | 0.67 |
| **Urinary System** | PFHpA | 1.02 [0.95, 1.1] | 0.53 |
| Urinary Bladder | PFHpA | 1.06 [0.96, 1.16] | 0.28 |
| Kidney and Renal Pelvis | PFHpA | 0.99 [0.89, 1.09] | 0.79 |
| Ureter | PFHpA | 1.21 [0.69, 2.13] | 0.50 |
| Other Urinary Organs | PFHpA | 0.89 [0.44, 1.79] | 0.74 |
| **Brain and Other Nervous System** | PFHpA | 1.07 [0.9, 1.26] | 0.45 |
| Brain | PFHpA | 1.05 [0.89, 1.26] | 0.55 |
| Cranial Nerves Other Nervous System | PFHpA | 1.27 [0.67, 2.44] | 0.46 |
| **Endocrine System** | PFHpA | 1.08 [0.97, 1.22] | 0.16 |
| Thyroid | PFHpA | 1.08 [0.96, 1.22] | 0.20 |
| Other Endocrine including Thymus | PFHpA | 1.12 [0.73, 1.72] | 0.60 |
| **Lymphoma** | PFHpA | 1 [0.91, 1.1] | 0.96 |
| **Hodgkin Lymphoma** | PFHpA | 1.04 [0.79, 1.35] | 0.80 |
| Hodgkin Nodal | PFHpA | 1.04 [0.8, 1.36] | 0.77 |
| Hodgkin Extranodal | PFHpA | 0.73 [0.07, 7.94] | 0.80 |
| Non Hodgkin Lymphoma | PFHpA | 1 [0.9, 1.1] | 0.97 |
| NHL Nodal | PFHpA | 0.97 [0.86, 1.1] | 0.63 |
| NHL Extranodal | PFHpA | 1.05 [0.89, 1.24] | 0.56 |
| **Myeloma** | PFHpA | 0.96 [0.82, 1.12] | 0.62 |
| **Leukemia** | PFHpA | 1.01 [0.9, 1.13] | 0.83 |
| Lymphocytic Leukemia | PFHpA | 1.02 [0.87, 1.19] | 0.83 |
| Acute Lymphocytic Leukemia | PFHpA | 0.96 [0.7, 1.33] | 0.82 |
| Chronic Lymphocytic Leukemia | PFHpA | 1.04 [0.86, 1.25] | 0.70 |
| Other Lymphocytic Leukemia | PFHpA | 1.01 [0.52, 1.96] | 0.99 |
| Myeloid and Monocytic Leukemia | PFHpA | 1 [0.85, 1.19] | 0.96 |
| Acute Myeloid Leukemia | PFHpA | 0.98 [0.79, 1.21] | 0.82 |
| Acute Monocytic Leukemia | PFHpA | 1.35 [0.51, 3.55] | 0.55 |
| Chronic Myeloid Leukemia | PFHpA | 1.04 [0.77, 1.4] | 0.81 |
| Other Myeloid Monocytic Leukemia | PFHpA | 0.97 [0.3, 3.15] | 0.96 |
| Other Leukemia | PFHpA | 1.03 [0.64, 1.65] | 0.90 |
| Other Acute Leukemia | PFHpA | 1.04 [0.42, 2.59] | 0.93 |
| Aleukemic Subleukemic and NOS | PFHpA | 1.02 [0.59, 1.77] | 0.95 |
| **All Sites** | PFBS | 1.04 [0.88, 1.22] | 0.68 |
| Oral Cavity and Pharynx | PFBS | 1.22 [0.74, 2.01] | 0.44 |
| Lip | PFBS | NA | NA |
| Tongue | PFBS | 1.53 [0.67, 3.47] | 0.31 |
| Salivary Gland | PFBS | 1.19 [0.25, 5.67] | 0.83 |
| Floor of Mouth | PFBS | NA | NA |
| Gum and Other Mouth | PFBS | 1.07 [0.24, 4.84] | 0.93 |
| Nasopharynx | PFBS | NA | NA |
| Tonsil | PFBS | 1.07 [0.34, 3.36] | 0.91 |
| Oropharynx | PFBS | 1.79 [0.32, 9.96] | 0.51 |
| Hypopharynx | PFBS | NA | NA |
| Other Oral Cavity and Pharynx | PFBS | NA | NA |
| **Digestive System** | PFBS | 0.94 [0.75, 1.17] | 0.57 |
| Esophagus | PFBS | 0.86 [0.34, 2.2] | 0.75 |
| Stomach | PFBS | 0.99 [0.46, 2.1] | 0.97 |
| Small Intestine | PFBS | 0.96 [0.26, 3.56] | 0.95 |
| Colon and Rectum | PFBS | 0.9 [0.66, 1.23] | 0.51 |
| Colon excluding Rectum | PFBS | 0.88 [0.6, 1.27] | 0.49 |
| Cecum | PFBS | 0.93 [0.4, 2.19] | 0.87 |
| Appendix | PFBS | 1.17 [0.31, 4.41] | 0.82 |
| Ascending Colon | PFBS | 1.09 [0.5, 2.4] | 0.83 |
| Hepatic Flexure | PFBS | 1.04 [0.22, 4.86] | 0.96 |
| Transverse Colon | PFBS | 0.62 [0.15, 2.55] | 0.50 |
| Splenic Flexure | PFBS | 0.74 [0.07, 8.31] | 0.81 |
| Descending Colon | PFBS | 0.8 [0.15, 4.3] | 0.80 |
| Sigmoid Colon | PFBS | 0.83 [0.39, 1.76] | 0.62 |
| Large Intestine NOS | PFBS | 0.61 [0.14, 2.74] | 0.52 |
| Rectum and Rectosigmoid Junction | PFBS | 0.96 [0.56, 1.65] | 0.87 |
| Rectosigmoid Junction | PFBS | 1.41 [0.54, 3.67] | 0.48 |
| Rectum | PFBS | 0.83 [0.43, 1.61] | 0.58 |
| Anus Anal Canal and Anorectum | PFBS | 1.33 [0.42, 4.23] | 0.62 |
| Liver and Intrahepatic Bile Duct | PFBS | 0.96 [0.51, 1.79] | 0.89 |
| Liver | PFBS | 0.9 [0.44, 1.84] | 0.77 |
| Intrahepatic Bile Duct | PFBS | 1.35 [0.32, 5.63] | 0.68 |
| Gallbladder | PFBS | 0.24 [0.01, 9.67] | 0.45 |
| Other Biliary | PFBS | 0.97 [0.22, 4.39] | 0.97 |
| Pancreas | PFBS | 1.13 [0.68, 1.88] | 0.63 |
| Retroperitoneum | PFBS | NA | NA |
| Peritoneum Omentum and Mesentery | PFBS | NA | NA |
| Other Digestive Organs | PFBS | 0.73 [0.06, 8.72] | 0.81 |
| **Respiratory System** | PFBS | 1.23 [0.95, 1.6] | 0.12 |
| Nose Nasal Cavity and Middle Ear | PFBS | 1.05 [0.11, 10.23] | 0.97 |
| Larynx | PFBS | 1.08 [0.42, 2.76] | 0.87 |
| Lung and Bronchus | PFBS | 1.25 [0.95, 1.63] | 0.11 |
| Pleura | PFBS | NA | NA |
| Trachea Mediastinum and Other Respiratory Organs | PFBS | NA | NA |
| Bones and Joints | PFBS | 1.7 [0.37, 7.83] | 0.50 |
| Soft Tissue including Heart | PFBS | 1.17 [0.41, 3.33] | 0.77 |
| Skin excluding Basal and Squamous | PFBS | 1 [0.54, 1.84] | 0.99 |
| Melanoma of the Skin | PFBS | 1.02 [0.55, 1.92] | 0.94 |
| Other Non Epithelial Skin | PFBS | 0.67 [0.1, 4.34] | 0.68 |
| **Breast** | PFBS | 1.09 [0.85, 1.39] | 0.49 |
| **Urinary System** | PFBS | 1 [0.74, 1.36] | 0.97 |
| Urinary Bladder | PFBS | 1.21 [0.8, 1.82] | 0.37 |
| Kidney and Renal Pelvis | PFBS | 0.85 [0.54, 1.33] | 0.48 |
| Ureter | PFBS | NA | NA |
| Other Urinary Organs | PFBS | NA | NA |
| **Brain and Other Nervous System** | PFBS | 0.83 [0.37, 1.86] | 0.66 |
| Brain | PFBS | 0.9 [0.4, 2.01] | 0.80 |
| Cranial Nerves Other Nervous System | PFBS | NA | NA |
| **Endocrine System** | PFBS | 1.17 [0.69, 1.97] | 0.56 |
| Thyroid | PFBS | 1.15 [0.67, 2] | 0.61 |
| Other Endocrine including Thymus | PFBS | 1.41 [0.22, 8.94] | 0.71 |
| **Lymphoma** | PFBS | 0.96 [0.62, 1.49] | 0.85 |
| **Hodgkin Lymphoma** | PFBS | 0.68 [0.15, 3.07] | 0.61 |
| Hodgkin Nodal | PFBS | 0.68 [0.15, 3.09] | 0.62 |
| Hodgkin Extranodal | PFBS | NA | NA |
| Non Hodgkin Lymphoma | PFBS | 1 [0.63, 1.58] | 0.99 |
| NHL Nodal | PFBS | 0.94 [0.53, 1.65] | 0.82 |
| NHL Extranodal | PFBS | 1.14 [0.52, 2.5] | 0.74 |
| **Myeloma** | PFBS | 1.18 [0.57, 2.45] | 0.66 |
| **Leukemia** | PFBS | 1.12 [0.7, 1.78] | 0.65 |
| Lymphocytic Leukemia | PFBS | 1.15 [0.59, 2.25] | 0.68 |
| Acute Lymphocytic Leukemia | PFBS | 0.88 [0.19, 4.15] | 0.87 |
| Chronic Lymphocytic Leukemia | PFBS | 1.32 [0.62, 2.79] | 0.48 |
| Other Lymphocytic Leukemia | PFBS | NA | NA |
| Myeloid and Monocytic Leukemia | PFBS | 1.08 [0.53, 2.19] | 0.84 |
| Acute Myeloid Leukemia | PFBS | 0.88 [0.33, 2.39] | 0.80 |
| Acute Monocytic Leukemia | PFBS | NA | NA |
| Chronic Myeloid Leukemia | PFBS | 1.52 [0.54, 4.31] | 0.43 |
| Other Myeloid Monocytic Leukemia | PFBS | NA | NA |
| Other Leukemia | PFBS | 1.13 [0.2, 6.24] | 0.89 |
| Other Acute Leukemia | PFBS | NA | NA |
| Aleukemic Subleukemic and NOS | PFBS | 1.65 [0.3, 9.17] | 0.57 |
| 1. All models were adjusted for county-level SES variables, urbanicity, smoking rate, obesity, and air pollution. | | | |
| 2. cells were highlighted if crude p values were less than 0.05. | | | |
| 3. NAs were produced with limited sample size. | | | |

| **Supplemental Table 13.** Sensitivity analysis of removing air pollution from the model. | | | | | |
| --- | --- | --- | --- | --- | --- |
| **Cancers** | **Exposures^4^** | **IRR [95% CI]** | **p value^1,2,3^** | **adjusted p value^1,2,3^** | **UCMR** |
| Skin excluding Basal and Squamous | PFBA | 0.91 [0.86, 0.96] | 0.00 | 0.00 | UCMR5 |
| Respiratory System | PFOA | 1.09 [1.03, 1.15] | 0.00 | 0.03 | UCMR3 |
| Digestive System | PFHxS | 1.1 [1.03, 1.18] | 0.00 | 0.06 | UCMR5 |
| Endocrine System | PFBA | 0.93 [0.88, 0.98] | 0.01 | 0.07 | UCMR5 |
| Digestive System | PFBA | 1.03 [1.01, 1.05] | 0.01 | 0.07 | UCMR5 |
| Respiratory System | PFBA | 1.04 [1.01, 1.07] | 0.01 | 0.11 | UCMR5 |
| Endocrine System | PFNA | 1.3 [1.06, 1.58] | 0.01 | 0.15 | UCMR3 |
| Endocrine System | PFHpA | 1.11 [1.02, 1.21] | 0.02 | 0.21 | UCMR5 |
| Oral Cavity and Pharynx | PFBS | 1.34 [1.04, 1.71] | 0.02 | 0.32 | UCMR3 |
| Digestive System | PFBS | 1.03 [1, 1.05] | 0.03 | 0.43 | UCMR5 |
| Respiratory System | PFBS | 1.18 [1.01, 1.36] | 0.03 | 0.41 | UCMR3 |
| Endocrine System | PFOS | 1.13 [1.01, 1.26] | 0.04 | 0.50 | UCMR3 |
| All Sites | PFOA | 1.03 [1, 1.06] | 0.04 | 0.50 | UCMR3 |
| Skin excluding Basal and Squamous | PFHpA | 0.91 [0.83, 1] | 0.04 | 0.57 | UCMR5 |
| Endocrine System | PFOA | 1.1 [1, 1.22] | 0.05 | 0.57 | UCMR3 |
| Urinary System | PFBA | 1.03 [1, 1.06] | 0.05 | 0.49 | UCMR5 |
| Leukemia | PFBA | 0.96 [0.91, 1] | 0.06 | 0.52 | UCMR5 |
| Oral Cavity and Pharynx | PFBS | 1.06 [1, 1.12] | 0.06 | 0.76 | UCMR5 |
| Endocrine System | PFHxS | 1.13 [0.99, 1.28] | 0.06 | 0.91 | UCMR3 |
| Breast | PFOA | 1.04 [1, 1.08] | 0.07 | 0.74 | UCMR3 |
| Urinary System | PFHxS | 0.92 [0.83, 1.01] | 0.08 | 1.00 | UCMR5 |
| Skin excluding Basal and Squamous | PFOS | 1.09 [0.99, 1.21] | 0.09 | 1.00 | UCMR5 |
| Respiratory System | PFPEA | 1.03 [1, 1.06] | 0.09 | 1.00 | UCMR5 |
| Respiratory System | PFHpA | 1.04 [0.99, 1.09] | 0.09 | 1.00 | UCMR5 |
| Breast | PFBS | 1.11 [0.98, 1.26] | 0.10 | 1.00 | UCMR3 |
| Digestive System | PFBS | 0.9 [0.8, 1.02] | 0.10 | 1.00 | UCMR3 |
| Endocrine System | PFPEA | 0.95 [0.9, 1.01] | 0.12 | 1.00 | UCMR5 |
| Urinary System | PFPEA | 0.97 [0.94, 1.01] | 0.12 | 1.00 | UCMR5 |
| All Sites | PFOS | 1.03 [0.99, 1.06] | 0.12 | 1.00 | UCMR3 |
| Respiratory System | PFBS | 1.03 [0.99, 1.06] | 0.13 | 1.00 | UCMR5 |
| Breast | PFBA | 0.98 [0.96, 1.01] | 0.13 | 1.00 | UCMR5 |
| Respiratory System | PFHXA | 1.03 [0.99, 1.06] | 0.14 | 1.00 | UCMR5 |
| Endocrine System | PFBS | 0.79 [0.57, 1.08] | 0.14 | 1.00 | UCMR3 |
| Respiratory System | PFOS | 1.05 [0.98, 1.12] | 0.15 | 1.00 | UCMR3 |
| Oral Cavity and Pharynx | PFBA | 1.04 [0.99, 1.09] | 0.16 | 1.00 | UCMR5 |
| Endocrine System | PFBS | 0.96 [0.9, 1.02] | 0.16 | 1.00 | UCMR5 |
| Oral Cavity and Pharynx | PFPEA | 1.04 [0.98, 1.1] | 0.16 | 1.00 | UCMR5 |
| Oral Cavity and Pharynx | PFHxS | 1.11 [0.95, 1.3] | 0.17 | 1.00 | UCMR5 |
| All Sites | PFNA | 1.04 [0.98, 1.11] | 0.18 | 1.00 | UCMR3 |
| Skin excluding Basal and Squamous | PFHxS | 1.12 [0.94, 1.33] | 0.19 | 1.00 | UCMR5 |
| Respiratory System | PFHpA | 1.04 [0.98, 1.11] | 0.20 | 1.00 | UCMR3 |
| Urinary System | PFHXA | 0.98 [0.95, 1.01] | 0.20 | 1.00 | UCMR5 |
| Brain and Other Nervous System | PFHpA | 1.08 [0.96, 1.21] | 0.20 | 1.00 | UCMR5 |
| Endocrine System | PFOA | 1.06 [0.97, 1.17] | 0.22 | 1.00 | UCMR5 |
| Endocrine System | PFHpA | 1.07 [0.96, 1.19] | 0.22 | 1.00 | UCMR3 |
| Urinary System | PFOA | 0.97 [0.92, 1.02] | 0.22 | 1.00 | UCMR5 |
| Digestive System | PFHXA | 1.02 [0.99, 1.04] | 0.23 | 1.00 | UCMR5 |
| Digestive System | PFOA | 1.02 [0.98, 1.06] | 0.24 | 1.00 | UCMR5 |
| All Sites | PFHpA | 1.02 [0.99, 1.04] | 0.24 | 1.00 | UCMR5 |
| Oral Cavity and Pharynx | PFOS | 1.06 [0.96, 1.17] | 0.24 | 1.00 | UCMR5 |
| Lymphoma | PFHpA | 1.04 [0.98, 1.11] | 0.24 | 1.00 | UCMR5 |
| Brain and Other Nervous System | PFOA | 1.08 [0.95, 1.23] | 0.24 | 1.00 | UCMR3 |
| Lymphoma | PFOS | 1.05 [0.97, 1.14] | 0.26 | 1.00 | UCMR3 |
| Brain and Other Nervous System | PFHpA | 1.09 [0.94, 1.26] | 0.26 | 1.00 | UCMR3 |
| Endocrine System | PFHXA | 0.96 [0.9, 1.03] | 0.27 | 1.00 | UCMR5 |
| Leukemia | PFHxS | 0.92 [0.78, 1.07] | 0.27 | 1.00 | UCMR5 |
| Digestive System | PFHpA | 1.02 [0.98, 1.06] | 0.28 | 1.00 | UCMR5 |
| All Sites | PFHpA | 1.02 [0.99, 1.05] | 0.29 | 1.00 | UCMR3 |
| All Sites | PFHxS | 1.02 [0.98, 1.06] | 0.29 | 1.00 | UCMR3 |
| Respiratory System | PFHxS | 1.04 [0.97, 1.12] | 0.29 | 1.00 | UCMR3 |
| Breast | PFHxS | 0.96 [0.89, 1.04] | 0.29 | 1.00 | UCMR5 |
| Soft Tissue including Heart | PFBS | 1.31 [0.79, 2.18] | 0.29 | 1.00 | UCMR3 |
| Lymphoma | PFOA | 1.04 [0.97, 1.12] | 0.30 | 1.00 | UCMR3 |
| Digestive System | PFPEA | 1.01 [0.99, 1.04] | 0.31 | 1.00 | UCMR5 |
| Lymphoma | PFBS | 0.98 [0.93, 1.02] | 0.31 | 1.00 | UCMR5 |
| Bones and Joints | PFHxS | 1.3 [0.78, 2.16] | 0.31 | 1.00 | UCMR5 |
| Myeloma | PFHpA | 0.94 [0.82, 1.07] | 0.34 | 1.00 | UCMR3 |
| Endocrine System | PFOS | 0.95 [0.85, 1.06] | 0.34 | 1.00 | UCMR5 |
| Lymphoma | PFHxS | 0.94 [0.82, 1.07] | 0.34 | 1.00 | UCMR5 |
| Myeloma | PFOA | 0.94 [0.83, 1.07] | 0.35 | 1.00 | UCMR3 |
| Brain and Other Nervous System | PFHxS | 0.89 [0.7, 1.14] | 0.35 | 1.00 | UCMR5 |
| Leukemia | PFPEA | 0.98 [0.93, 1.03] | 0.35 | 1.00 | UCMR5 |
| All Sites | PFOA | 1.01 [0.99, 1.04] | 0.36 | 1.00 | UCMR5 |
| Skin excluding Basal and Squamous | PFBS | 1.15 [0.85, 1.57] | 0.36 | 1.00 | UCMR3 |
| Oral Cavity and Pharynx | PFHXA | 1.03 [0.97, 1.09] | 0.37 | 1.00 | UCMR5 |
| Breast | PFOA | 1.02 [0.98, 1.06] | 0.38 | 1.00 | UCMR5 |
| Urinary System | PFBS | 0.99 [0.95, 1.02] | 0.38 | 1.00 | UCMR5 |
| Myeloma | PFOS | 0.94 [0.81, 1.09] | 0.39 | 1.00 | UCMR3 |
| Soft Tissue including Heart | PFPEA | 1.05 [0.94, 1.17] | 0.40 | 1.00 | UCMR5 |
| Respiratory System | PFNA | 1.06 [0.93, 1.2] | 0.40 | 1.00 | UCMR3 |
| Breast | PFHXA | 0.99 [0.96, 1.02] | 0.40 | 1.00 | UCMR5 |
| Skin excluding Basal and Squamous | PFOA | 0.96 [0.86, 1.06] | 0.40 | 1.00 | UCMR3 |
| Myeloma | PFHxS | 0.93 [0.79, 1.1] | 0.42 | 1.00 | UCMR3 |
| Oral Cavity and Pharynx | PFOA | 1.04 [0.94, 1.15] | 0.43 | 1.00 | UCMR3 |
| Bones and Joints | PFBS | 1.39 [0.61, 3.19] | 0.44 | 1.00 | UCMR3 |
| Respiratory System | PFOA | 1.02 [0.97, 1.07] | 0.45 | 1.00 | UCMR5 |
| Brain and Other Nervous System | PFOS | 1.06 [0.91, 1.24] | 0.46 | 1.00 | UCMR3 |
| Myeloma | PFOS | 0.95 [0.84, 1.08] | 0.46 | 1.00 | UCMR5 |
| Skin excluding Basal and Squamous | PFBS | 1.02 [0.96, 1.09] | 0.48 | 1.00 | UCMR5 |
| Digestive System | PFNA | 1.03 [0.94, 1.13] | 0.48 | 1.00 | UCMR3 |
| All Sites | PFBS | 1.01 [0.99, 1.02] | 0.48 | 1.00 | UCMR5 |
| Breast | PFNA | 1.03 [0.94, 1.13] | 0.49 | 1.00 | UCMR3 |
| Lymphoma | PFBA | 0.99 [0.95, 1.03] | 0.49 | 1.00 | UCMR5 |
| Lymphoma | PFHxS | 1.03 [0.94, 1.14] | 0.49 | 1.00 | UCMR3 |
| Soft Tissue including Heart | PFOA | 1.07 [0.89, 1.28] | 0.50 | 1.00 | UCMR3 |
| Skin excluding Basal and Squamous | PFHxS | 0.95 [0.83, 1.09] | 0.50 | 1.00 | UCMR3 |
| Brain and Other Nervous System | PFBA | 0.98 [0.91, 1.05] | 0.51 | 1.00 | UCMR5 |
| Skin excluding Basal and Squamous | PFOS | 0.96 [0.85, 1.09] | 0.53 | 1.00 | UCMR3 |
| Leukemia | PFHpA | 1.02 [0.95, 1.1] | 0.55 | 1.00 | UCMR5 |
| Leukemia | PFBS | 0.98 [0.93, 1.04] | 0.55 | 1.00 | UCMR5 |
| Skin excluding Basal and Squamous | PFHXA | 1.02 [0.96, 1.09] | 0.56 | 1.00 | UCMR5 |
| Lymphoma | PFOS | 0.98 [0.9, 1.06] | 0.56 | 1.00 | UCMR5 |
| Urinary System | PFOS | 1.02 [0.96, 1.08] | 0.57 | 1.00 | UCMR3 |
| Bones and Joints | PFBA | 0.95 [0.79, 1.14] | 0.58 | 1.00 | UCMR5 |
| Breast | PFOS | 1.01 [0.97, 1.06] | 0.59 | 1.00 | UCMR5 |
| Soft Tissue including Heart | PFHxS | 1.09 [0.79, 1.49] | 0.60 | 1.00 | UCMR5 |
| Breast | PFHpA | 1.01 [0.97, 1.06] | 0.60 | 1.00 | UCMR3 |
| Myeloma | PFBA | 1.02 [0.95, 1.09] | 0.61 | 1.00 | UCMR5 |
| Lymphoma | PFHpA | 1.02 [0.94, 1.11] | 0.61 | 1.00 | UCMR3 |
| Brain and Other Nervous System | PFOA | 1.03 [0.91, 1.17] | 0.62 | 1.00 | UCMR5 |
| Leukemia | PFOS | 1.02 [0.93, 1.12] | 0.63 | 1.00 | UCMR5 |
| Brain and Other Nervous System | PFNA | 1.07 [0.8, 1.44] | 0.63 | 1.00 | UCMR3 |
| Soft Tissue including Heart | PFBS | 1.03 [0.92, 1.15] | 0.63 | 1.00 | UCMR5 |
| Lymphoma | PFHXA | 0.99 [0.94, 1.04] | 0.63 | 1.00 | UCMR5 |
| Urinary System | PFOS | 0.99 [0.93, 1.04] | 0.64 | 1.00 | UCMR5 |
| Soft Tissue including Heart | PFHXA | 1.03 [0.91, 1.16] | 0.65 | 1.00 | UCMR5 |
| Leukemia | PFHxS | 1.03 [0.92, 1.15] | 0.65 | 1.00 | UCMR3 |
| Lymphoma | PFOA | 1.02 [0.95, 1.09] | 0.66 | 1.00 | UCMR5 |
| Breast | PFOS | 1.01 [0.96, 1.06] | 0.66 | 1.00 | UCMR3 |
| Brain and Other Nervous System | PFOS | 0.97 [0.84, 1.12] | 0.66 | 1.00 | UCMR5 |
| Myeloma | PFPEA | 0.98 [0.91, 1.06] | 0.67 | 1.00 | UCMR5 |
| Lymphoma | PFBS | 0.95 [0.76, 1.19] | 0.67 | 1.00 | UCMR3 |
| Leukemia | PFHXA | 0.99 [0.94, 1.04] | 0.67 | 1.00 | UCMR5 |
| Leukemia | PFBS | 1.05 [0.82, 1.35] | 0.67 | 1.00 | UCMR3 |
| Breast | PFBS | 1.01 [0.98, 1.03] | 0.68 | 1.00 | UCMR5 |
| Brain and Other Nervous System | PFBS | 1.02 [0.94, 1.1] | 0.68 | 1.00 | UCMR5 |
| Digestive System | PFOS | 1.01 [0.97, 1.05] | 0.69 | 1.00 | UCMR5 |
| All Sites | PFBS | 1.02 [0.94, 1.1] | 0.69 | 1.00 | UCMR3 |
| Soft Tissue including Heart | PFOA | 1.04 [0.87, 1.24] | 0.69 | 1.00 | UCMR5 |
| Skin excluding Basal and Squamous | PFHpA | 0.98 [0.87, 1.1] | 0.69 | 1.00 | UCMR3 |
| Endocrine System | PFHxS | 1.04 [0.87, 1.24] | 0.70 | 1.00 | UCMR5 |
| Myeloma | PFHpA | 0.98 [0.88, 1.09] | 0.71 | 1.00 | UCMR5 |
| Brain and Other Nervous System | PFHxS | 1.03 [0.87, 1.23] | 0.73 | 1.00 | UCMR3 |
| Bones and Joints | PFHpA | 1.05 [0.79, 1.4] | 0.73 | 1.00 | UCMR5 |
| Skin excluding Basal and Squamous | PFPEA | 1.01 [0.95, 1.07] | 0.74 | 1.00 | UCMR5 |
| Myeloma | PFBS | 1.07 [0.72, 1.58] | 0.75 | 1.00 | UCMR3 |
| Myeloma | PFNA | 0.96 [0.73, 1.26] | 0.75 | 1.00 | UCMR3 |
| Leukemia | PFNA | 0.97 [0.79, 1.18] | 0.75 | 1.00 | UCMR3 |
| Digestive System | PFHxS | 1.01 [0.96, 1.07] | 0.75 | 1.00 | UCMR3 |
| Urinary System | PFNA | 1.02 [0.9, 1.15] | 0.75 | 1.00 | UCMR3 |
| Digestive System | PFOA | 1.01 [0.97, 1.05] | 0.76 | 1.00 | UCMR3 |
| Myeloma | PFBS | 1.01 [0.94, 1.09] | 0.76 | 1.00 | UCMR5 |
| Soft Tissue including Heart | PFHpA | 0.97 [0.79, 1.19] | 0.76 | 1.00 | UCMR3 |
| Oral Cavity and Pharynx | PFHpA | 1.01 [0.93, 1.1] | 0.77 | 1.00 | UCMR5 |
| Soft Tissue including Heart | PFOS | 1.03 [0.85, 1.25] | 0.78 | 1.00 | UCMR5 |
| Brain and Other Nervous System | PFPEA | 1.01 [0.93, 1.09] | 0.78 | 1.00 | UCMR5 |
| Urinary System | PFHxS | 1.01 [0.94, 1.08] | 0.79 | 1.00 | UCMR3 |
| Soft Tissue including Heart | PFBA | 0.99 [0.89, 1.09] | 0.79 | 1.00 | UCMR5 |
| Urinary System | PFHpA | 1.01 [0.96, 1.05] | 0.80 | 1.00 | UCMR5 |
| Bones and Joints | PFNA | 0.91 [0.42, 1.96] | 0.80 | 1.00 | UCMR3 |
| Brain and Other Nervous System | PFBS | 1.05 [0.71, 1.54] | 0.82 | 1.00 | UCMR3 |
| Bones and Joints | PFPEA | 0.98 [0.8, 1.19] | 0.82 | 1.00 | UCMR5 |
| Bones and Joints | PFHpA | 0.96 [0.66, 1.4] | 0.82 | 1.00 | UCMR3 |
| Bones and Joints | PFHxS | 0.95 [0.61, 1.49] | 0.82 | 1.00 | UCMR3 |
| Urinary System | PFBS | 0.98 [0.84, 1.15] | 0.83 | 1.00 | UCMR3 |
| Leukemia | PFOS | 1.01 [0.91, 1.12] | 0.83 | 1.00 | UCMR3 |
| Breast | PFPEA | 1 [0.97, 1.02] | 0.84 | 1.00 | UCMR5 |
| Soft Tissue including Heart | PFOS | 1.02 [0.82, 1.27] | 0.84 | 1.00 | UCMR3 |
| Oral Cavity and Pharynx | PFNA | 0.98 [0.78, 1.22] | 0.85 | 1.00 | UCMR3 |
| All Sites | PFOS | 1 [0.97, 1.03] | 0.85 | 1.00 | UCMR5 |
| Leukemia | PFHpA | 1.01 [0.92, 1.11] | 0.85 | 1.00 | UCMR3 |
| Oral Cavity and Pharynx | PFOA | 0.99 [0.91, 1.08] | 0.85 | 1.00 | UCMR5 |
| Digestive System | PFHpA | 1 [0.96, 1.05] | 0.86 | 1.00 | UCMR3 |
| Myeloma | PFHXA | 1.01 [0.93, 1.09] | 0.86 | 1.00 | UCMR5 |
| Skin excluding Basal and Squamous | PFOA | 1.01 [0.92, 1.11] | 0.86 | 1.00 | UCMR5 |
| Brain and Other Nervous System | PFHXA | 0.99 [0.91, 1.08] | 0.86 | 1.00 | UCMR5 |
| Lymphoma | PFPEA | 1 [0.96, 1.05] | 0.87 | 1.00 | UCMR5 |
| All Sites | PFHXA | 1 [0.98, 1.02] | 0.88 | 1.00 | UCMR5 |
| Myeloma | PFOA | 0.99 [0.88, 1.11] | 0.89 | 1.00 | UCMR5 |
| Respiratory System | PFHxS | 1.01 [0.92, 1.1] | 0.89 | 1.00 | UCMR5 |
| Oral Cavity and Pharynx | PFHpA | 1.01 [0.9, 1.12] | 0.89 | 1.00 | UCMR3 |
| Breast | PFHpA | 1 [0.96, 1.03] | 0.90 | 1.00 | UCMR5 |
| Bones and Joints | PFOA | 0.98 [0.7, 1.38] | 0.91 | 1.00 | UCMR3 |
| Skin excluding Basal and Squamous | PFNA | 1.01 [0.81, 1.27] | 0.91 | 1.00 | UCMR3 |
| Respiratory System | PFOS | 1 [0.95, 1.06] | 0.91 | 1.00 | UCMR5 |
| Bones and Joints | PFOA | 0.98 [0.71, 1.35] | 0.92 | 1.00 | UCMR5 |
| Bones and Joints | PFOS | 0.98 [0.66, 1.45] | 0.92 | 1.00 | UCMR3 |
| Urinary System | PFHpA | 1 [0.94, 1.07] | 0.92 | 1.00 | UCMR3 |
| Soft Tissue including Heart | PFHxS | 0.99 [0.77, 1.26] | 0.92 | 1.00 | UCMR3 |
| Oral Cavity and Pharynx | PFHxS | 0.99 [0.87, 1.13] | 0.93 | 1.00 | UCMR3 |
| Soft Tissue including Heart | PFHpA | 1.01 [0.86, 1.18] | 0.93 | 1.00 | UCMR5 |
| All Sites | PFHxS | 1 [0.95, 1.05] | 0.94 | 1.00 | UCMR5 |
| Soft Tissue including Heart | PFNA | 0.98 [0.65, 1.49] | 0.94 | 1.00 | UCMR3 |
| Bones and Joints | PFHXA | 1.01 [0.81, 1.25] | 0.95 | 1.00 | UCMR5 |
| All Sites | PFBA | 1 [0.99, 1.02] | 0.95 | 1.00 | UCMR5 |
| Leukemia | PFOA | 1 [0.92, 1.08] | 0.96 | 1.00 | UCMR5 |
| Breast | PFHxS | 1 [0.95, 1.06] | 0.96 | 1.00 | UCMR3 |
| Bones and Joints | PFBS | 1 [0.81, 1.22] | 0.96 | 1.00 | UCMR5 |
| Leukemia | PFOA | 1 [0.91, 1.09] | 0.96 | 1.00 | UCMR3 |
| Lymphoma | PFNA | 1 [0.85, 1.18] | 0.97 | 1.00 | UCMR3 |
| Oral Cavity and Pharynx | PFOS | 1 [0.89, 1.12] | 0.97 | 1.00 | UCMR3 |
| Bones and Joints | PFOS | 0.99 [0.7, 1.41] | 0.97 | 1.00 | UCMR5 |
| Urinary System | PFOA | 1 [0.95, 1.06] | 0.98 | 1.00 | UCMR3 |
| Digestive System | PFOS | 1 [0.95, 1.05] | 0.98 | 1.00 | UCMR3 |
| Myeloma | PFHxS | 1 [0.81, 1.23] | 1.00 | 1.00 | UCMR5 |
| All Sites | PFPEA | 1 [0.98, 1.02] | 1.00 | 1.00 | UCMR5 |
| 1. All models were adjusted for county-level SES variables, urbanicity, smoking rate, and obesity. | | | | | |
| 2. p values were adjusted using false discovery rate method and were adjusted by PFAS chemical. | | | | | |
| 3. cells were highlighted if crude or adjusted p values were less than 0.05. | | | | | |
| 4. All PFAS exposure in UCMR3 were categorized as detected/non-detected and in UCMR5, PFOA, PFOS, PFHxS were categorized as MCL violation and the rest of PFAS was categorized as detected/non-detected. | | | | | |

| **Supplemental Table 14.** Sensitivity analysis of removing obesity from the model. | | | | | |
| --- | --- | --- | --- | --- | --- |
| **Cancers** | **Exposures^4^** | **IRR [95% CI]** | **p value^1,2,3^** | **adjusted p value^1,2,3^** | **UCMR** |
| Digestive System | PFHxS | 1.14 [1.07, 1.21] | 0.00 | 0.00 | UCMR5 |
| Skin excluding Basal and Squamous | PFBA | 0.91 [0.86, 0.96] | 0.00 | 0.00 | UCMR5 |
| Respiratory System | PFOA | 1.1 [1.04, 1.16] | 0.00 | 0.01 | UCMR3 |
| Endocrine System | PFBA | 0.92 [0.87, 0.97] | 0.00 | 0.02 | UCMR5 |
| Endocrine System | PFNA | 1.31 [1.07, 1.6] | 0.01 | 0.13 | UCMR3 |
| Digestive System | PFBA | 1.03 [1.01, 1.05] | 0.01 | 0.14 | UCMR5 |
| Endocrine System | PFHpA | 1.11 [1.02, 1.2] | 0.02 | 0.29 | UCMR5 |
| Endocrine System | PFOS | 1.14 [1.02, 1.27] | 0.02 | 0.30 | UCMR3 |
| Breast | PFBA | 0.97 [0.95, 1] | 0.03 | 0.28 | UCMR5 |
| Digestive System | PFBS | 1.03 [1, 1.05] | 0.04 | 0.49 | UCMR5 |
| Oral Cavity and Pharynx | PFBS | 1.31 [1.02, 1.68] | 0.04 | 0.50 | UCMR3 |
| Oral Cavity and Pharynx | PFHxS | 1.17 [1.01, 1.36] | 0.04 | 0.48 | UCMR5 |
| Endocrine System | PFOA | 1.11 [1.01, 1.22] | 0.04 | 0.50 | UCMR3 |
| Digestive System | PFBS | 0.88 [0.79, 0.99] | 0.04 | 0.50 | UCMR3 |
| All Sites | PFOA | 1.03 [1, 1.06] | 0.04 | 0.50 | UCMR3 |
| Respiratory System | PFHpA | 1.07 [1, 1.13] | 0.04 | 0.62 | UCMR3 |
| Endocrine System | PFHxS | 1.14 [1, 1.29] | 0.05 | 0.67 | UCMR3 |
| Skin excluding Basal and Squamous | PFOS | 1.11 [1, 1.23] | 0.05 | 0.71 | UCMR5 |
| Skin excluding Basal and Squamous | PFHpA | 0.92 [0.84, 1] | 0.05 | 0.66 | UCMR5 |
| Respiratory System | PFHpA | 1.05 [1, 1.1] | 0.06 | 0.68 | UCMR5 |
| Breast | PFOA | 1.04 [1, 1.08] | 0.06 | 0.65 | UCMR3 |
| Oral Cavity and Pharynx | PFBS | 1.06 [1, 1.12] | 0.06 | 0.78 | UCMR5 |
| Urinary System | PFBA | 1.03 [1, 1.06] | 0.07 | 0.65 | UCMR5 |
| Leukemia | PFBA | 0.96 [0.91, 1] | 0.07 | 0.65 | UCMR5 |
| Skin excluding Basal and Squamous | PFHxS | 1.16 [0.98, 1.36] | 0.08 | 1.00 | UCMR5 |
| All Sites | PFHpA | 1.03 [1, 1.06] | 0.09 | 1.00 | UCMR3 |
| Respiratory System | PFBA | 1.02 [1, 1.05] | 0.10 | 0.77 | UCMR5 |
| Brain and Other Nervous System | PFHpA | 1.13 [0.98, 1.31] | 0.10 | 1.00 | UCMR3 |
| Respiratory System | PFBS | 1.14 [0.98, 1.32] | 0.10 | 1.00 | UCMR3 |
| Endocrine System | PFPEA | 0.95 [0.9, 1.01] | 0.11 | 1.00 | UCMR5 |
| Oral Cavity and Pharynx | PFBA | 1.04 [0.99, 1.09] | 0.11 | 0.77 | UCMR5 |
| Respiratory System | PFHXA | 1.03 [0.99, 1.06] | 0.12 | 1.00 | UCMR5 |
| Respiratory System | PFPEA | 1.03 [0.99, 1.06] | 0.12 | 1.00 | UCMR5 |
| All Sites | PFHxS | 1.04 [0.99, 1.09] | 0.12 | 1.00 | UCMR5 |
| Oral Cavity and Pharynx | PFOS | 1.08 [0.98, 1.19] | 0.12 | 1.00 | UCMR5 |
| Respiratory System | PFOS | 1.05 [0.99, 1.12] | 0.13 | 1.00 | UCMR3 |
| Endocrine System | PFOA | 1.08 [0.98, 1.18] | 0.13 | 1.00 | UCMR5 |
| Urinary System | PFPEA | 0.98 [0.95, 1.01] | 0.13 | 1.00 | UCMR5 |
| Brain and Other Nervous System | PFOA | 1.1 [0.97, 1.25] | 0.14 | 1.00 | UCMR3 |
| All Sites | PFOS | 1.02 [0.99, 1.06] | 0.14 | 1.00 | UCMR3 |
| Respiratory System | PFBS | 1.02 [0.99, 1.06] | 0.15 | 1.00 | UCMR5 |
| Respiratory System | PFHxS | 1.06 [0.98, 1.14] | 0.16 | 1.00 | UCMR3 |
| Oral Cavity and Pharynx | PFPEA | 1.04 [0.98, 1.1] | 0.16 | 1.00 | UCMR5 |
| Brain and Other Nervous System | PFHpA | 1.08 [0.97, 1.21] | 0.16 | 1.00 | UCMR5 |
| All Sites | PFHpA | 1.02 [0.99, 1.04] | 0.17 | 1.00 | UCMR5 |
| All Sites | PFNA | 1.04 [0.98, 1.11] | 0.17 | 1.00 | UCMR3 |
| Breast | PFBS | 1.09 [0.96, 1.24] | 0.18 | 1.00 | UCMR3 |
| Digestive System | PFHpA | 1.02 [0.99, 1.06] | 0.19 | 1.00 | UCMR5 |
| Endocrine System | PFBS | 0.81 [0.59, 1.11] | 0.19 | 1.00 | UCMR3 |
| Urinary System | PFHXA | 0.98 [0.95, 1.01] | 0.19 | 1.00 | UCMR5 |
| Endocrine System | PFBS | 0.96 [0.9, 1.02] | 0.20 | 1.00 | UCMR5 |
| All Sites | PFHxS | 1.02 [0.99, 1.06] | 0.20 | 1.00 | UCMR3 |
| Endocrine System | PFHpA | 1.07 [0.96, 1.2] | 0.21 | 1.00 | UCMR3 |
| Digestive System | PFHXA | 1.02 [0.99, 1.04] | 0.22 | 1.00 | UCMR5 |
| Lymphoma | PFOS | 1.05 [0.97, 1.14] | 0.23 | 1.00 | UCMR3 |
| All Sites | PFOA | 1.02 [0.99, 1.04] | 0.23 | 1.00 | UCMR5 |
| Digestive System | PFOA | 1.02 [0.98, 1.06] | 0.25 | 1.00 | UCMR5 |
| Urinary System | PFOA | 0.97 [0.92, 1.02] | 0.26 | 1.00 | UCMR5 |
| Soft Tissue including Heart | PFHxS | 1.19 [0.88, 1.6] | 0.27 | 1.00 | UCMR5 |
| Brain and Other Nervous System | PFOS | 1.09 [0.93, 1.27] | 0.28 | 1.00 | UCMR3 |
| All Sites | PFOS | 1.02 [0.99, 1.05] | 0.28 | 1.00 | UCMR5 |
| Brain and Other Nervous System | PFBA | 0.96 [0.9, 1.03] | 0.28 | 1.00 | UCMR5 |
| Digestive System | PFPEA | 1.01 [0.99, 1.04] | 0.29 | 1.00 | UCMR5 |
| Myeloma | PFOA | 0.93 [0.82, 1.06] | 0.29 | 1.00 | UCMR3 |
| Breast | PFOA | 1.02 [0.98, 1.06] | 0.29 | 1.00 | UCMR5 |
| Breast | PFOS | 1.02 [0.98, 1.07] | 0.30 | 1.00 | UCMR5 |
| Lymphoma | PFOA | 1.04 [0.97, 1.11] | 0.31 | 1.00 | UCMR3 |
| Lymphoma | PFHpA | 1.03 [0.97, 1.1] | 0.31 | 1.00 | UCMR5 |
| Lymphoma | PFBS | 0.98 [0.94, 1.02] | 0.33 | 1.00 | UCMR5 |
| Soft Tissue including Heart | PFBS | 1.29 [0.78, 2.15] | 0.33 | 1.00 | UCMR3 |
| Respiratory System | PFHxS | 1.05 [0.96, 1.14] | 0.33 | 1.00 | UCMR5 |
| Breast | PFHpA | 1.02 [0.98, 1.07] | 0.33 | 1.00 | UCMR3 |
| Endocrine System | PFHXA | 0.97 [0.91, 1.03] | 0.33 | 1.00 | UCMR5 |
| Digestive System | PFOS | 1.02 [0.98, 1.06] | 0.35 | 1.00 | UCMR5 |
| Skin excluding Basal and Squamous | PFBS | 1.16 [0.85, 1.58] | 0.35 | 1.00 | UCMR3 |
| Skin excluding Basal and Squamous | PFBS | 1.03 [0.97, 1.09] | 0.36 | 1.00 | UCMR5 |
| Urinary System | PFBS | 0.99 [0.95, 1.02] | 0.37 | 1.00 | UCMR5 |
| Myeloma | PFOS | 0.94 [0.81, 1.08] | 0.37 | 1.00 | UCMR3 |
| Respiratory System | PFNA | 1.06 [0.93, 1.2] | 0.38 | 1.00 | UCMR3 |
| Respiratory System | PFOA | 1.02 [0.97, 1.08] | 0.38 | 1.00 | UCMR5 |
| Bones and Joints | PFBS | 1.45 [0.63, 3.34] | 0.38 | 1.00 | UCMR3 |
| Oral Cavity and Pharynx | PFHXA | 1.03 [0.97, 1.09] | 0.39 | 1.00 | UCMR5 |
| Myeloma | PFHpA | 0.94 [0.82, 1.08] | 0.40 | 1.00 | UCMR3 |
| Leukemia | PFHxS | 0.94 [0.81, 1.09] | 0.40 | 1.00 | UCMR5 |
| Soft Tissue including Heart | PFPEA | 1.05 [0.94, 1.17] | 0.41 | 1.00 | UCMR5 |
| Skin excluding Basal and Squamous | PFHXA | 1.03 [0.96, 1.09] | 0.41 | 1.00 | UCMR5 |
| Myeloma | PFHxS | 0.93 [0.79, 1.1] | 0.42 | 1.00 | UCMR3 |
| Lymphoma | PFHxS | 1.04 [0.94, 1.14] | 0.43 | 1.00 | UCMR3 |
| Brain and Other Nervous System | PFHxS | 0.91 [0.72, 1.15] | 0.44 | 1.00 | UCMR5 |
| All Sites | PFBS | 1.01 [0.99, 1.02] | 0.45 | 1.00 | UCMR5 |
| Skin excluding Basal and Squamous | PFOA | 0.96 [0.87, 1.07] | 0.46 | 1.00 | UCMR3 |
| Skin excluding Basal and Squamous | PFHxS | 0.95 [0.83, 1.09] | 0.46 | 1.00 | UCMR3 |
| Brain and Other Nervous System | PFHxS | 1.07 [0.9, 1.27] | 0.46 | 1.00 | UCMR3 |
| All Sites | PFBA | 0.99 [0.98, 1.01] | 0.46 | 1.00 | UCMR5 |
| Endocrine System | PFOS | 0.96 [0.86, 1.07] | 0.47 | 1.00 | UCMR5 |
| Breast | PFNA | 1.03 [0.94, 1.13] | 0.47 | 1.00 | UCMR3 |
| Bones and Joints | PFHxS | 1.2 [0.73, 1.98] | 0.47 | 1.00 | UCMR5 |
| Breast | PFHXA | 0.99 [0.96, 1.02] | 0.48 | 1.00 | UCMR5 |
| Skin excluding Basal and Squamous | PFOS | 0.96 [0.85, 1.08] | 0.49 | 1.00 | UCMR3 |
| Respiratory System | PFOS | 1.02 [0.96, 1.08] | 0.50 | 1.00 | UCMR5 |
| Leukemia | PFPEA | 0.98 [0.93, 1.03] | 0.50 | 1.00 | UCMR5 |
| Brain and Other Nervous System | PFNA | 1.1 [0.82, 1.47] | 0.52 | 1.00 | UCMR3 |
| Myeloma | PFHxS | 1.07 [0.88, 1.29] | 0.52 | 1.00 | UCMR5 |
| Lymphoma | PFHpA | 1.03 [0.95, 1.11] | 0.53 | 1.00 | UCMR3 |
| Brain and Other Nervous System | PFOA | 1.04 [0.92, 1.18] | 0.53 | 1.00 | UCMR5 |
| Endocrine System | PFHxS | 1.06 [0.89, 1.25] | 0.53 | 1.00 | UCMR5 |
| Leukemia | PFOS | 1.03 [0.94, 1.12] | 0.55 | 1.00 | UCMR5 |
| Digestive System | PFNA | 1.03 [0.94, 1.13] | 0.55 | 1.00 | UCMR3 |
| Soft Tissue including Heart | PFBS | 1.03 [0.93, 1.16] | 0.55 | 1.00 | UCMR5 |
| Myeloma | PFBA | 1.02 [0.95, 1.09] | 0.56 | 1.00 | UCMR5 |
| Urinary System | PFBS | 0.96 [0.82, 1.12] | 0.57 | 1.00 | UCMR3 |
| Soft Tissue including Heart | PFOS | 1.06 [0.87, 1.28] | 0.57 | 1.00 | UCMR5 |
| Lymphoma | PFHXA | 0.99 [0.94, 1.03] | 0.57 | 1.00 | UCMR5 |
| Lymphoma | PFBA | 0.99 [0.95, 1.03] | 0.57 | 1.00 | UCMR5 |
| Leukemia | PFBS | 0.99 [0.94, 1.04] | 0.59 | 1.00 | UCMR5 |
| Leukemia | PFHpA | 1.02 [0.95, 1.1] | 0.59 | 1.00 | UCMR5 |
| Soft Tissue including Heart | PFHXA | 1.03 [0.92, 1.16] | 0.59 | 1.00 | UCMR5 |
| Bones and Joints | PFHpA | 0.91 [0.62, 1.32] | 0.61 | 1.00 | UCMR3 |
| Oral Cavity and Pharynx | PFOA | 1.02 [0.93, 1.13] | 0.61 | 1.00 | UCMR3 |
| Skin excluding Basal and Squamous | PFPEA | 1.02 [0.96, 1.08] | 0.61 | 1.00 | UCMR5 |
| Urinary System | PFHxS | 0.98 [0.89, 1.07] | 0.63 | 1.00 | UCMR5 |
| Lymphoma | PFOA | 1.02 [0.95, 1.09] | 0.64 | 1.00 | UCMR5 |
| Skin excluding Basal and Squamous | PFHpA | 0.97 [0.87, 1.09] | 0.65 | 1.00 | UCMR3 |
| Breast | PFOS | 1.01 [0.96, 1.06] | 0.65 | 1.00 | UCMR3 |
| Myeloma | PFBS | 1.02 [0.94, 1.1] | 0.65 | 1.00 | UCMR5 |
| Oral Cavity and Pharynx | PFHpA | 1.02 [0.94, 1.11] | 0.65 | 1.00 | UCMR5 |
| Digestive System | PFHpA | 1.01 [0.97, 1.06] | 0.65 | 1.00 | UCMR3 |
| Breast | PFBS | 1.01 [0.98, 1.03] | 0.66 | 1.00 | UCMR5 |
| Lymphoma | PFBS | 0.95 [0.76, 1.19] | 0.66 | 1.00 | UCMR3 |
| Bones and Joints | PFBA | 0.96 [0.8, 1.15] | 0.66 | 1.00 | UCMR5 |
| Soft Tissue including Heart | PFOA | 1.04 [0.87, 1.23] | 0.67 | 1.00 | UCMR5 |
| Leukemia | PFHXA | 0.99 [0.94, 1.04] | 0.67 | 1.00 | UCMR5 |
| Leukemia | PFHxS | 1.02 [0.91, 1.15] | 0.68 | 1.00 | UCMR3 |
| Soft Tissue including Heart | PFOA | 1.04 [0.87, 1.25] | 0.68 | 1.00 | UCMR3 |
| Skin excluding Basal and Squamous | PFOA | 1.02 [0.93, 1.12] | 0.69 | 1.00 | UCMR5 |
| Bones and Joints | PFHxS | 0.92 [0.59, 1.43] | 0.70 | 1.00 | UCMR3 |
| Soft Tissue including Heart | PFHpA | 0.96 [0.78, 1.18] | 0.70 | 1.00 | UCMR3 |
| Urinary System | PFHpA | 1.01 [0.96, 1.06] | 0.70 | 1.00 | UCMR5 |
| Leukemia | PFOA | 0.98 [0.9, 1.07] | 0.71 | 1.00 | UCMR3 |
| Brain and Other Nervous System | PFBS | 1.01 [0.94, 1.1] | 0.72 | 1.00 | UCMR5 |
| Myeloma | PFPEA | 0.99 [0.92, 1.06] | 0.72 | 1.00 | UCMR5 |
| All Sites | PFHXA | 1 [0.99, 1.02] | 0.72 | 1.00 | UCMR5 |
| Urinary System | PFHpA | 1.01 [0.95, 1.07] | 0.73 | 1.00 | UCMR3 |
| Leukemia | PFBS | 1.04 [0.82, 1.34] | 0.73 | 1.00 | UCMR3 |
| Myeloma | PFHXA | 1.01 [0.94, 1.1] | 0.73 | 1.00 | UCMR5 |
| Leukemia | PFNA | 0.97 [0.79, 1.18] | 0.74 | 1.00 | UCMR3 |
| Lymphoma | PFPEA | 1.01 [0.96, 1.05] | 0.74 | 1.00 | UCMR5 |
| Myeloma | PFOS | 0.98 [0.86, 1.11] | 0.74 | 1.00 | UCMR5 |
| Bones and Joints | PFNA | 0.88 [0.41, 1.9] | 0.75 | 1.00 | UCMR3 |
| Lymphoma | PFOS | 0.99 [0.91, 1.07] | 0.75 | 1.00 | UCMR5 |
| Breast | PFHxS | 0.99 [0.92, 1.06] | 0.75 | 1.00 | UCMR5 |
| Myeloma | PFNA | 0.96 [0.73, 1.26] | 0.75 | 1.00 | UCMR3 |
| Urinary System | PFOS | 1.01 [0.95, 1.07] | 0.76 | 1.00 | UCMR3 |
| Brain and Other Nervous System | PFPEA | 1.01 [0.94, 1.09] | 0.76 | 1.00 | UCMR5 |
| Lymphoma | PFHxS | 0.98 [0.86, 1.11] | 0.76 | 1.00 | UCMR5 |
| Bones and Joints | PFOA | 0.95 [0.68, 1.33] | 0.77 | 1.00 | UCMR3 |
| Oral Cavity and Pharynx | PFNA | 0.97 [0.78, 1.21] | 0.77 | 1.00 | UCMR3 |
| Digestive System | PFOS | 0.99 [0.95, 1.04] | 0.78 | 1.00 | UCMR3 |
| Myeloma | PFHpA | 0.99 [0.89, 1.1] | 0.79 | 1.00 | UCMR5 |
| Bones and Joints | PFPEA | 0.97 [0.8, 1.19] | 0.79 | 1.00 | UCMR5 |
| Breast | PFHxS | 1.01 [0.95, 1.06] | 0.79 | 1.00 | UCMR3 |
| Brain and Other Nervous System | PFOS | 0.98 [0.85, 1.13] | 0.79 | 1.00 | UCMR5 |
| Oral Cavity and Pharynx | PFOS | 0.99 [0.88, 1.1] | 0.80 | 1.00 | UCMR3 |
| Soft Tissue including Heart | PFHxS | 0.97 [0.76, 1.24] | 0.81 | 1.00 | UCMR3 |
| Bones and Joints | PFOS | 0.95 [0.65, 1.4] | 0.81 | 1.00 | UCMR3 |
| Oral Cavity and Pharynx | PFHxS | 0.98 [0.87, 1.12] | 0.81 | 1.00 | UCMR3 |
| Digestive System | PFHxS | 1.01 [0.95, 1.06] | 0.81 | 1.00 | UCMR3 |
| Urinary System | PFHxS | 1.01 [0.94, 1.08] | 0.82 | 1.00 | UCMR3 |
| Bones and Joints | PFOS | 0.96 [0.68, 1.37] | 0.83 | 1.00 | UCMR5 |
| Bones and Joints | PFOA | 0.97 [0.7, 1.33] | 0.83 | 1.00 | UCMR5 |
| Urinary System | PFNA | 1.01 [0.9, 1.14] | 0.84 | 1.00 | UCMR3 |
| Leukemia | PFHpA | 1.01 [0.92, 1.11] | 0.84 | 1.00 | UCMR3 |
| Myeloma | PFBS | 1.04 [0.7, 1.54] | 0.84 | 1.00 | UCMR3 |
| Oral Cavity and Pharynx | PFOA | 0.99 [0.91, 1.08] | 0.87 | 1.00 | UCMR5 |
| Soft Tissue including Heart | PFHpA | 1.01 [0.86, 1.19] | 0.87 | 1.00 | UCMR5 |
| Urinary System | PFOS | 1 [0.95, 1.06] | 0.88 | 1.00 | UCMR5 |
| Bones and Joints | PFHpA | 1.02 [0.77, 1.36] | 0.88 | 1.00 | UCMR5 |
| Soft Tissue including Heart | PFNA | 0.97 [0.64, 1.47] | 0.89 | 1.00 | UCMR3 |
| Oral Cavity and Pharynx | PFHpA | 1.01 [0.91, 1.12] | 0.89 | 1.00 | UCMR3 |
| Breast | PFHpA | 1 [0.96, 1.03] | 0.89 | 1.00 | UCMR5 |
| Leukemia | PFOS | 1.01 [0.91, 1.11] | 0.90 | 1.00 | UCMR3 |
| Urinary System | PFOA | 1 [0.95, 1.05] | 0.90 | 1.00 | UCMR3 |
| Myeloma | PFOA | 1.01 [0.9, 1.13] | 0.91 | 1.00 | UCMR5 |
| Brain and Other Nervous System | PFBS | 1.02 [0.7, 1.5] | 0.91 | 1.00 | UCMR3 |
| All Sites | PFPEA | 1 [0.98, 1.02] | 0.92 | 1.00 | UCMR5 |
| Lymphoma | PFNA | 1.01 [0.86, 1.18] | 0.93 | 1.00 | UCMR3 |
| Soft Tissue including Heart | PFBA | 1 [0.9, 1.1] | 0.93 | 1.00 | UCMR5 |
| Skin excluding Basal and Squamous | PFNA | 1.01 [0.8, 1.27] | 0.94 | 1.00 | UCMR3 |
| All Sites | PFBS | 1 [0.92, 1.08] | 0.95 | 1.00 | UCMR3 |
| Breast | PFPEA | 1 [0.97, 1.02] | 0.95 | 1.00 | UCMR5 |
| Bones and Joints | PFHXA | 1 [0.81, 1.23] | 0.97 | 1.00 | UCMR5 |
| Soft Tissue including Heart | PFOS | 1 [0.81, 1.24] | 0.98 | 1.00 | UCMR3 |
| Brain and Other Nervous System | PFHXA | 1 [0.92, 1.09] | 0.98 | 1.00 | UCMR5 |
| Leukemia | PFOA | 1 [0.92, 1.09] | 0.99 | 1.00 | UCMR5 |
| Digestive System | PFOA | 1 [0.96, 1.04] | 0.99 | 1.00 | UCMR3 |
| Bones and Joints | PFBS | 1 [0.82, 1.22] | 1.00 | 1.00 | UCMR5 |
| 1. All models were adjusted for county-level SES variables, urbanicity, smoking rate and air pollution. | | | | | |
| 2. p values were adjusted using false discovery rate method and were adjusted by PFAS chemical. | | | | | |
| 3. cells were highlighted if crude or adjusted p values were less than 0.05. | | | | | |
| 4. All PFAS exposure in UCMR3 were categorized as detected/non-detected and in UCMR5, PFOA, PFOS, PFHxS were categorized as MCL violation and the rest of PFAS was categorized as detected/non-detected. | | | | | |

| **Supplemental Table 15.** Sensitivity analysis of additionally controlling for number of potential PFAS polluting facilities in the county. | | | | | |
| --- | --- | --- | --- | --- | --- |
| **Cancers** | **Exposures^4^** | **IRR [95% CI]** | **p value^1,2,3^** | **adjusted p value^1,2,3^** | **UCMR** |
| Digestive System | PFHxS | 1.14 [1.06, 1.21] | 0.00 | 0.00 | UCMR5 |
| Skin excluding Basal and Squamous | PFBA | 0.91 [0.86, 0.95] | 0.00 | 0.00 | UCMR5 |
| Respiratory System | PFOA | 1.09 [1.04, 1.15] | 0.00 | 0.02 | UCMR3 |
| Endocrine System | PFBA | 0.93 [0.88, 0.98] | 0.01 | 0.08 | UCMR5 |
| Endocrine System | PFNA | 1.3 [1.06, 1.58] | 0.01 | 0.16 | UCMR3 |
| Endocrine System | PFHpA | 1.11 [1.02, 1.21] | 0.02 | 0.23 | UCMR5 |
| All Sites | PFOA | 1.03 [1.01, 1.06] | 0.02 | 0.22 | UCMR3 |
| Digestive System | PFBA | 1.03 [1, 1.05] | 0.02 | 0.23 | UCMR5 |
| Respiratory System | PFHpA | 1.05 [1.01, 1.1] | 0.02 | 0.31 | UCMR5 |
| Respiratory System | PFBA | 1.03 [1, 1.06] | 0.03 | 0.35 | UCMR5 |
| Oral Cavity and Pharynx | PFBS | 1.31 [1.02, 1.69] | 0.03 | 0.46 | UCMR3 |
| Endocrine System | PFOS | 1.13 [1.01, 1.26] | 0.03 | 0.48 | UCMR3 |
| Digestive System | PFBS | 0.88 [0.79, 0.99] | 0.04 | 0.50 | UCMR3 |
| Digestive System | PFBS | 1.03 [1, 1.05] | 0.04 | 0.59 | UCMR5 |
| Breast | PFOA | 1.04 [1, 1.09] | 0.04 | 0.51 | UCMR3 |
| Leukemia | PFBA | 0.95 [0.91, 1] | 0.05 | 0.48 | UCMR5 |
| Respiratory System | PFHpA | 1.06 [1, 1.13] | 0.05 | 0.68 | UCMR3 |
| Breast | PFBA | 0.98 [0.96, 1] | 0.05 | 0.48 | UCMR5 |
| Endocrine System | PFOA | 1.1 [1, 1.21] | 0.05 | 0.55 | UCMR3 |
| All Sites | PFHpA | 1.03 [1, 1.06] | 0.06 | 0.79 | UCMR3 |
| Skin excluding Basal and Squamous | PFHpA | 0.92 [0.84, 1] | 0.06 | 0.75 | UCMR5 |
| All Sites | PFOS | 1.03 [1, 1.06] | 0.07 | 0.89 | UCMR3 |
| Endocrine System | PFHxS | 1.12 [0.99, 1.28] | 0.07 | 0.97 | UCMR3 |
| Oral Cavity and Pharynx | PFBS | 1.05 [0.99, 1.11] | 0.08 | 1.00 | UCMR5 |
| Respiratory System | PFBS | 1.14 [0.99, 1.32] | 0.08 | 0.95 | UCMR3 |
| All Sites | PFHpA | 1.02 [1, 1.05] | 0.08 | 0.89 | UCMR5 |
| Urinary System | PFPEA | 0.97 [0.94, 1] | 0.09 | 1.00 | UCMR5 |
| Respiratory System | PFOS | 1.06 [0.99, 1.13] | 0.10 | 1.00 | UCMR3 |
| Oral Cavity and Pharynx | PFHxS | 1.14 [0.97, 1.33] | 0.10 | 1.00 | UCMR5 |
| Skin excluding Basal and Squamous | PFOS | 1.09 [0.98, 1.21] | 0.11 | 1.00 | UCMR5 |
| Respiratory System | PFHXA | 1.03 [0.99, 1.06] | 0.11 | 1.00 | UCMR5 |
| All Sites | PFHxS | 1.03 [0.99, 1.07] | 0.12 | 1.00 | UCMR3 |
| Endocrine System | PFPEA | 0.95 [0.9, 1.01] | 0.12 | 1.00 | UCMR5 |
| Respiratory System | PFPEA | 1.02 [0.99, 1.06] | 0.12 | 1.00 | UCMR5 |
| Urinary System | PFBA | 1.02 [0.99, 1.05] | 0.13 | 1.00 | UCMR5 |
| Respiratory System | PFHxS | 1.06 [0.98, 1.14] | 0.14 | 1.00 | UCMR3 |
| Brain and Other Nervous System | PFHpA | 1.11 [0.96, 1.29] | 0.16 | 1.00 | UCMR3 |
| Brain and Other Nervous System | PFHpA | 1.09 [0.97, 1.22] | 0.16 | 1.00 | UCMR5 |
| Breast | PFBS | 1.09 [0.96, 1.24] | 0.16 | 1.00 | UCMR3 |
| Endocrine System | PFBS | 0.8 [0.58, 1.1] | 0.17 | 1.00 | UCMR3 |
| All Sites | PFNA | 1.04 [0.98, 1.11] | 0.17 | 1.00 | UCMR3 |
| Endocrine System | PFBS | 0.96 [0.9, 1.02] | 0.17 | 1.00 | UCMR5 |
| Digestive System | PFHpA | 1.02 [0.99, 1.06] | 0.18 | 1.00 | UCMR5 |
| Oral Cavity and Pharynx | PFOS | 1.07 [0.97, 1.17] | 0.19 | 1.00 | UCMR5 |
| Oral Cavity and Pharynx | PFPEA | 1.04 [0.98, 1.1] | 0.20 | 1.00 | UCMR5 |
| Lymphoma | PFHpA | 1.04 [0.98, 1.11] | 0.20 | 1.00 | UCMR5 |
| Digestive System | PFHXA | 1.02 [0.99, 1.04] | 0.20 | 1.00 | UCMR5 |
| Urinary System | PFOA | 0.97 [0.92, 1.02] | 0.20 | 1.00 | UCMR5 |
| Respiratory System | PFBS | 1.02 [0.99, 1.05] | 0.20 | 1.00 | UCMR5 |
| Urinary System | PFHXA | 0.98 [0.95, 1.01] | 0.20 | 1.00 | UCMR5 |
| Endocrine System | PFOA | 1.06 [0.97, 1.17] | 0.21 | 1.00 | UCMR5 |
| Brain and Other Nervous System | PFOA | 1.09 [0.95, 1.24] | 0.22 | 1.00 | UCMR3 |
| Skin excluding Basal and Squamous | PFHxS | 1.11 [0.94, 1.32] | 0.22 | 1.00 | UCMR5 |
| Oral Cavity and Pharynx | PFBA | 1.03 [0.98, 1.08] | 0.23 | 1.00 | UCMR5 |
| Digestive System | PFOA | 1.02 [0.99, 1.06] | 0.23 | 1.00 | UCMR5 |
| Urinary System | PFHxS | 0.94 [0.86, 1.04] | 0.24 | 1.00 | UCMR5 |
| Lymphoma | PFOS | 1.05 [0.97, 1.14] | 0.26 | 1.00 | UCMR3 |
| Endocrine System | PFHXA | 0.96 [0.9, 1.03] | 0.27 | 1.00 | UCMR5 |
| Endocrine System | PFHpA | 1.06 [0.95, 1.19] | 0.27 | 1.00 | UCMR3 |
| Leukemia | PFHxS | 0.92 [0.78, 1.07] | 0.27 | 1.00 | UCMR5 |
| Urinary System | PFBS | 0.98 [0.95, 1.01] | 0.28 | 1.00 | UCMR5 |
| Lymphoma | PFBS | 0.98 [0.93, 1.02] | 0.28 | 1.00 | UCMR5 |
| Leukemia | PFPEA | 0.97 [0.92, 1.02] | 0.29 | 1.00 | UCMR5 |
| Lymphoma | PFOA | 1.04 [0.97, 1.12] | 0.30 | 1.00 | UCMR3 |
| Digestive System | PFPEA | 1.01 [0.99, 1.04] | 0.30 | 1.00 | UCMR5 |
| Breast | PFHpA | 1.02 [0.98, 1.07] | 0.31 | 1.00 | UCMR3 |
| Soft Tissue including Heart | PFBS | 1.3 [0.78, 2.17] | 0.31 | 1.00 | UCMR3 |
| Endocrine System | PFOS | 0.95 [0.85, 1.06] | 0.32 | 1.00 | UCMR5 |
| All Sites | PFHxS | 1.02 [0.98, 1.07] | 0.35 | 1.00 | UCMR5 |
| Myeloma | PFOA | 0.94 [0.83, 1.07] | 0.36 | 1.00 | UCMR3 |
| Bones and Joints | PFBS | 1.47 [0.64, 3.39] | 0.37 | 1.00 | UCMR3 |
| Respiratory System | PFHxS | 1.04 [0.95, 1.14] | 0.37 | 1.00 | UCMR5 |
| Oral Cavity and Pharynx | PFHXA | 1.03 [0.97, 1.09] | 0.37 | 1.00 | UCMR5 |
| Skin excluding Basal and Squamous | PFBS | 1.15 [0.84, 1.57] | 0.37 | 1.00 | UCMR3 |
| Oral Cavity and Pharynx | PFOA | 1.04 [0.95, 1.15] | 0.38 | 1.00 | UCMR3 |
| Breast | PFHXA | 0.99 [0.96, 1.02] | 0.39 | 1.00 | UCMR5 |
| Myeloma | PFOS | 0.94 [0.81, 1.09] | 0.39 | 1.00 | UCMR3 |
| Myeloma | PFHpA | 0.94 [0.82, 1.08] | 0.39 | 1.00 | UCMR3 |
| Lymphoma | PFBA | 0.98 [0.94, 1.02] | 0.40 | 1.00 | UCMR5 |
| Digestive System | PFOS | 1.02 [0.98, 1.06] | 0.40 | 1.00 | UCMR5 |
| Brain and Other Nervous System | PFBA | 0.97 [0.9, 1.04] | 0.41 | 1.00 | UCMR5 |
| Respiratory System | PFNA | 1.05 [0.93, 1.19] | 0.41 | 1.00 | UCMR3 |
| All Sites | PFOA | 1.01 [0.98, 1.04] | 0.41 | 1.00 | UCMR5 |
| Breast | PFOS | 1.02 [0.97, 1.06] | 0.41 | 1.00 | UCMR5 |
| Soft Tissue including Heart | PFPEA | 1.05 [0.94, 1.17] | 0.42 | 1.00 | UCMR5 |
| Leukemia | PFHpA | 1.03 [0.96, 1.11] | 0.43 | 1.00 | UCMR5 |
| Lymphoma | PFHxS | 0.95 [0.83, 1.08] | 0.43 | 1.00 | UCMR5 |
| Digestive System | PFHpA | 1.02 [0.97, 1.07] | 0.43 | 1.00 | UCMR3 |
| Brain and Other Nervous System | PFOS | 1.06 [0.91, 1.24] | 0.44 | 1.00 | UCMR3 |
| Myeloma | PFHxS | 0.94 [0.79, 1.11] | 0.44 | 1.00 | UCMR3 |
| Breast | PFOA | 1.02 [0.98, 1.06] | 0.44 | 1.00 | UCMR5 |
| Bones and Joints | PFHxS | 1.22 [0.73, 2.04] | 0.45 | 1.00 | UCMR5 |
| Digestive System | PFNA | 1.03 [0.95, 1.13] | 0.46 | 1.00 | UCMR3 |
| Skin excluding Basal and Squamous | PFOA | 0.96 [0.87, 1.07] | 0.47 | 1.00 | UCMR3 |
| Soft Tissue including Heart | PFOA | 1.07 [0.89, 1.29] | 0.48 | 1.00 | UCMR3 |
| Myeloma | PFOS | 0.95 [0.84, 1.09] | 0.48 | 1.00 | UCMR5 |
| Leukemia | PFBS | 0.98 [0.93, 1.03] | 0.48 | 1.00 | UCMR5 |
| Lymphoma | PFHxS | 1.03 [0.94, 1.14] | 0.48 | 1.00 | UCMR3 |
| Brain and Other Nervous System | PFHxS | 0.92 [0.72, 1.17] | 0.48 | 1.00 | UCMR5 |
| Respiratory System | PFOA | 1.02 [0.97, 1.07] | 0.49 | 1.00 | UCMR5 |
| Breast | PFNA | 1.03 [0.94, 1.13] | 0.49 | 1.00 | UCMR3 |
| Soft Tissue including Heart | PFHxS | 1.11 [0.81, 1.53] | 0.51 | 1.00 | UCMR5 |
| Urinary System | PFHpA | 1.02 [0.96, 1.08] | 0.52 | 1.00 | UCMR3 |
| Urinary System | PFOS | 1.02 [0.96, 1.09] | 0.52 | 1.00 | UCMR3 |
| Breast | PFOS | 1.02 [0.97, 1.07] | 0.53 | 1.00 | UCMR3 |
| Digestive System | PFHxS | 1.02 [0.96, 1.07] | 0.54 | 1.00 | UCMR3 |
| All Sites | PFOS | 1.01 [0.98, 1.04] | 0.54 | 1.00 | UCMR5 |
| Skin excluding Basal and Squamous | PFBS | 1.02 [0.96, 1.08] | 0.54 | 1.00 | UCMR5 |
| All Sites | PFBA | 1 [0.98, 1.01] | 0.55 | 1.00 | UCMR5 |
| Leukemia | PFHxS | 1.04 [0.92, 1.16] | 0.55 | 1.00 | UCMR3 |
| Skin excluding Basal and Squamous | PFHXA | 1.02 [0.96, 1.09] | 0.56 | 1.00 | UCMR5 |
| Urinary System | PFBS | 0.96 [0.82, 1.12] | 0.56 | 1.00 | UCMR3 |
| Urinary System | PFHpA | 1.01 [0.97, 1.06] | 0.56 | 1.00 | UCMR5 |
| Lymphoma | PFHpA | 1.02 [0.94, 1.11] | 0.57 | 1.00 | UCMR3 |
| Breast | PFHxS | 0.98 [0.91, 1.06] | 0.58 | 1.00 | UCMR5 |
| Urinary System | PFHxS | 1.02 [0.95, 1.1] | 0.60 | 1.00 | UCMR3 |
| Lymphoma | PFOS | 0.98 [0.91, 1.06] | 0.62 | 1.00 | UCMR5 |
| Brain and Other Nervous System | PFNA | 1.08 [0.81, 1.44] | 0.62 | 1.00 | UCMR3 |
| Oral Cavity and Pharynx | PFHpA | 1.02 [0.94, 1.11] | 0.63 | 1.00 | UCMR5 |
| Lymphoma | PFHXA | 0.99 [0.94, 1.04] | 0.63 | 1.00 | UCMR5 |
| Myeloma | PFBA | 1.02 [0.95, 1.09] | 0.63 | 1.00 | UCMR5 |
| Brain and Other Nervous System | PFHxS | 1.04 [0.87, 1.25] | 0.63 | 1.00 | UCMR3 |
| Respiratory System | PFOS | 1.01 [0.96, 1.07] | 0.64 | 1.00 | UCMR5 |
| Lymphoma | PFBS | 0.95 [0.76, 1.18] | 0.64 | 1.00 | UCMR3 |
| Brain and Other Nervous System | PFOA | 1.03 [0.91, 1.17] | 0.65 | 1.00 | UCMR5 |
| Soft Tissue including Heart | PFHXA | 1.03 [0.91, 1.16] | 0.65 | 1.00 | UCMR5 |
| Leukemia | PFOS | 1.02 [0.93, 1.12] | 0.65 | 1.00 | UCMR5 |
| Digestive System | PFOA | 1.01 [0.97, 1.05] | 0.65 | 1.00 | UCMR3 |
| Myeloma | PFPEA | 0.98 [0.91, 1.06] | 0.66 | 1.00 | UCMR5 |
| Skin excluding Basal and Squamous | PFHxS | 0.97 [0.85, 1.11] | 0.66 | 1.00 | UCMR3 |
| Leukemia | PFHXA | 0.99 [0.94, 1.04] | 0.67 | 1.00 | UCMR5 |
| Soft Tissue including Heart | PFBS | 1.02 [0.91, 1.15] | 0.68 | 1.00 | UCMR5 |
| Skin excluding Basal and Squamous | PFOS | 0.97 [0.86, 1.1] | 0.68 | 1.00 | UCMR3 |
| Bones and Joints | PFBA | 0.96 [0.8, 1.16] | 0.68 | 1.00 | UCMR5 |
| Lymphoma | PFOA | 1.01 [0.95, 1.09] | 0.68 | 1.00 | UCMR5 |
| Breast | PFPEA | 0.99 [0.97, 1.02] | 0.69 | 1.00 | UCMR5 |
| Breast | PFHxS | 1.01 [0.96, 1.07] | 0.70 | 1.00 | UCMR3 |
| Bones and Joints | PFHpA | 0.93 [0.64, 1.36] | 0.71 | 1.00 | UCMR3 |
| Soft Tissue including Heart | PFOA | 1.03 [0.87, 1.23] | 0.71 | 1.00 | UCMR5 |
| Soft Tissue including Heart | PFBA | 0.98 [0.89, 1.09] | 0.71 | 1.00 | UCMR5 |
| All Sites | PFBS | 1 [0.99, 1.02] | 0.71 | 1.00 | UCMR5 |
| Oral Cavity and Pharynx | PFHpA | 1.02 [0.92, 1.14] | 0.72 | 1.00 | UCMR3 |
| Leukemia | PFHpA | 1.02 [0.92, 1.12] | 0.73 | 1.00 | UCMR3 |
| Leukemia | PFBS | 1.04 [0.81, 1.34] | 0.73 | 1.00 | UCMR3 |
| Soft Tissue including Heart | PFOS | 1.03 [0.85, 1.26] | 0.73 | 1.00 | UCMR5 |
| Myeloma | PFHpA | 0.98 [0.88, 1.09] | 0.74 | 1.00 | UCMR5 |
| Leukemia | PFNA | 0.97 [0.79, 1.18] | 0.74 | 1.00 | UCMR3 |
| Brain and Other Nervous System | PFBS | 1.01 [0.93, 1.1] | 0.74 | 1.00 | UCMR5 |
| Leukemia | PFOS | 1.02 [0.92, 1.13] | 0.74 | 1.00 | UCMR3 |
| Urinary System | PFNA | 1.02 [0.9, 1.15] | 0.75 | 1.00 | UCMR3 |
| Myeloma | PFNA | 0.96 [0.73, 1.26] | 0.75 | 1.00 | UCMR3 |
| Bones and Joints | PFHpA | 1.05 [0.78, 1.4] | 0.76 | 1.00 | UCMR5 |
| Brain and Other Nervous System | PFOS | 0.98 [0.85, 1.13] | 0.76 | 1.00 | UCMR5 |
| Endocrine System | PFHxS | 1.03 [0.86, 1.23] | 0.76 | 1.00 | UCMR5 |
| Myeloma | PFBS | 1.01 [0.94, 1.09] | 0.78 | 1.00 | UCMR5 |
| Skin excluding Basal and Squamous | PFHpA | 0.98 [0.88, 1.1] | 0.78 | 1.00 | UCMR3 |
| Bones and Joints | PFNA | 0.9 [0.42, 1.94] | 0.78 | 1.00 | UCMR3 |
| Soft Tissue including Heart | PFHpA | 0.97 [0.79, 1.2] | 0.80 | 1.00 | UCMR3 |
| Myeloma | PFBS | 1.05 [0.71, 1.55] | 0.81 | 1.00 | UCMR3 |
| Brain and Other Nervous System | PFPEA | 1.01 [0.93, 1.09] | 0.81 | 1.00 | UCMR5 |
| Bones and Joints | PFPEA | 0.98 [0.8, 1.19] | 0.81 | 1.00 | UCMR5 |
| Oral Cavity and Pharynx | PFOA | 0.99 [0.9, 1.08] | 0.82 | 1.00 | UCMR5 |
| Breast | PFHpA | 1 [0.97, 1.04] | 0.82 | 1.00 | UCMR5 |
| All Sites | PFPEA | 1 [0.98, 1.01] | 0.82 | 1.00 | UCMR5 |
| Soft Tissue including Heart | PFOS | 1.03 [0.82, 1.27] | 0.82 | 1.00 | UCMR3 |
| Bones and Joints | PFHxS | 0.95 [0.61, 1.49] | 0.83 | 1.00 | UCMR3 |
| Oral Cavity and Pharynx | PFNA | 0.98 [0.78, 1.22] | 0.84 | 1.00 | UCMR3 |
| Skin excluding Basal and Squamous | PFPEA | 1.01 [0.95, 1.07] | 0.85 | 1.00 | UCMR5 |
| All Sites | PFHXA | 1 [0.98, 1.02] | 0.85 | 1.00 | UCMR5 |
| Bones and Joints | PFOS | 0.97 [0.68, 1.38] | 0.86 | 1.00 | UCMR5 |
| Myeloma | PFHXA | 1.01 [0.93, 1.09] | 0.86 | 1.00 | UCMR5 |
| Breast | PFBS | 1 [0.98, 1.03] | 0.86 | 1.00 | UCMR5 |
| Soft Tissue including Heart | PFHpA | 1.01 [0.86, 1.19] | 0.87 | 1.00 | UCMR5 |
| Brain and Other Nervous System | PFHXA | 0.99 [0.91, 1.08] | 0.87 | 1.00 | UCMR5 |
| Urinary System | PFOS | 1 [0.94, 1.05] | 0.88 | 1.00 | UCMR5 |
| Myeloma | PFOA | 0.99 [0.88, 1.11] | 0.88 | 1.00 | UCMR5 |
| Urinary System | PFOA | 1 [0.95, 1.06] | 0.88 | 1.00 | UCMR3 |
| Bones and Joints | PFOA | 0.98 [0.7, 1.37] | 0.89 | 1.00 | UCMR3 |
| Skin excluding Basal and Squamous | PFOA | 1.01 [0.91, 1.11] | 0.90 | 1.00 | UCMR5 |
| Digestive System | PFOS | 1 [0.96, 1.05] | 0.91 | 1.00 | UCMR3 |
| Lymphoma | PFPEA | 1 [0.96, 1.05] | 0.91 | 1.00 | UCMR5 |
| Leukemia | PFOA | 1 [0.92, 1.08] | 0.91 | 1.00 | UCMR5 |
| Bones and Joints | PFOA | 0.98 [0.71, 1.35] | 0.91 | 1.00 | UCMR5 |
| Oral Cavity and Pharynx | PFHxS | 1.01 [0.88, 1.15] | 0.93 | 1.00 | UCMR3 |
| Skin excluding Basal and Squamous | PFNA | 1.01 [0.81, 1.27] | 0.93 | 1.00 | UCMR3 |
| Soft Tissue including Heart | PFNA | 0.98 [0.65, 1.49] | 0.93 | 1.00 | UCMR3 |
| Oral Cavity and Pharynx | PFOS | 1 [0.9, 1.13] | 0.94 | 1.00 | UCMR3 |
| Bones and Joints | PFHXA | 1.01 [0.82, 1.24] | 0.95 | 1.00 | UCMR5 |
| Soft Tissue including Heart | PFHxS | 0.99 [0.77, 1.27] | 0.95 | 1.00 | UCMR3 |
| Myeloma | PFHxS | 1.01 [0.82, 1.24] | 0.95 | 1.00 | UCMR5 |
| Brain and Other Nervous System | PFBS | 1.01 [0.69, 1.49] | 0.95 | 1.00 | UCMR3 |
| All Sites | PFBS | 1 [0.92, 1.08] | 0.96 | 1.00 | UCMR3 |
| Bones and Joints | PFOS | 0.99 [0.67, 1.47] | 0.96 | 1.00 | UCMR3 |
| Lymphoma | PFNA | 1 [0.85, 1.18] | 0.96 | 1.00 | UCMR3 |
| Leukemia | PFOA | 1 [0.92, 1.09] | 0.98 | 1.00 | UCMR3 |
| Bones and Joints | PFBS | 1 [0.81, 1.23] | 0.99 | 1.00 | UCMR5 |
| 1. All models were adjusted for county-level SES variables, urbanicity, smoking rate, obesity, air pollution and number of potential PFAS polluting facilities. | | | | | |
| 2. p values were adjusted using false discovery rate method and were adjusted by PFAS chemical. | | | | | |
| 3. cells were highlighted if crude or adjusted p values were less than 0.05. | | | | | |
| 4. All PFAS exposure in UCMR3 were categorized as detected/non-detected and in UCMR5, PFOA, PFOS, PFHxS were categorized as MCL violation and the rest of PFAS was categorized as detected/non-detected. | | | | | |
