# Supplementary material for: Associations between per-and polyfluoroalkyl substances (PFAS) and county-level cancer incidence between 2016 and 2021 and incident cancer burden attributable to PFAS in drinking water in the United States
Source: J Expo Sci Environ Epidemiol. 2025 Jan 9;35(3):425–36. doi: 10.1038/s41370-024-00742-2 (PMC12069088; doi:10.1038/s41370-024-00742-2)
Supplement: Supplementary file 5 — Supplemental Table 4 [file 41370_2024_742_MOESM5_ESM.docx]

| **Supplemental Table 16.** Stratified analysis by sex for the associations between detection of PFAS in drinking water using UCMR3 data and cancer incidence between 2016 and 2021. | | | | | | |
| --- | --- | --- | --- | --- | --- | --- |
|  |  | **Male** | | **Female** | |  |
| **Cancers** | **Exposures** | **IRR [95% CI]** | **p value^1,2^** | **IRR [95% CI]** | **p value^1,2^** | **Indicator of Significance^3,4^** |
| Hepatic Flexure | PFBS | 2.69 [1.23, 5.91] | 0.01 | 3.01 [1.41, 6.42] | 0.00 | ++ |
| Trachea Mediastinum and Other Respiratory Organs | PFOS | 1.97 [1.04, 3.74] | 0.04 | 2.61 [1.19, 5.71] | 0.02 | ++ |
| Splenic Flexure | PFHpA | 0.63 [0.4, 0.99] | 0.04 | 0.54 [0.3, 0.97] | 0.04 | ++ |
| Acute Monocytic Leukemia | PFBS | 8.22 [1.84, 36.83] | 0.01 | NA | NA | NA |
| Hodgkin Extranodal | PFHpA | 6.22 [1.83, 21.15] | 0.00 | 0.9 [0.1, 7.94] | 0.92 | + |
| Retroperitoneum | PFNA | 2.74 [1.2, 6.22] | 0.02 | 0.46 [0.05, 4.16] | 0.49 | + |
| Trachea Mediastinum and Other Respiratory Organs | PFHxS | 2.43 [1.24, 4.74] | 0.01 | 1.2 [0.43, 3.35] | 0.73 | + |
| Other Biliary | PFBS | 2.12 [1.04, 4.31] | 0.04 | 0.95 [0.37, 2.46] | 0.91 | + |
| Other Digestive Organs | PFHpA | 1.59 [1.07, 2.37] | 0.02 | 1.37 [0.9, 2.09] | 0.15 | + |
| Small Intestine | PFOA | 1.36 [1.1, 1.68] | 0.01 | 1.05 [0.82, 1.34] | 0.73 | + |
| Cecum | PFHxS | 1.27 [1.02, 1.57] | 0.03 | 0.9 [0.73, 1.12] | 0.36 | + |
| Brain | PFHpA | 1.25 [1.03, 1.52] | 0.02 | 0.86 [0.69, 1.06] | 0.15 | + |
| Acute Myeloid Leukemia | PFOS | 1.23 [1.01, 1.49] | 0.04 | 1.18 [0.93, 1.51] | 0.17 | + |
| Brain and Other Nervous System | PFHpA | 1.22 [1.02, 1.47] | 0.03 | 1.01 [0.82, 1.24] | 0.93 | + |
| NHL Extranodal | PFOA | 1.2 [1.02, 1.41] | 0.03 | 0.96 [0.8, 1.15] | 0.68 | + |
| Kidney and Renal Pelvis | PFHxS | 1.13 [1.01, 1.27] | 0.03 | 0.92 [0.78, 1.09] | 0.32 | + |
| Kidney and Renal Pelvis | PFOS | 1.12 [1.01, 1.24] | 0.04 | 0.98 [0.84, 1.14] | 0.78 | + |
| Urinary System | PFHxS | 1.11 [1, 1.22] | 0.04 | 0.94 [0.83, 1.06] | 0.32 | + |
| Urinary System | PFOS | 1.1 [1.01, 1.2] | 0.03 | 0.97 [0.86, 1.08] | 0.54 | + |
| Stomach | PFOA | 0.83 [0.69, 1] | 0.05 | 0.98 [0.79, 1.2] | 0.82 | + |
| Myeloma | PFOS | 0.83 [0.7, 0.99] | 0.03 | 1 [0.82, 1.22] | 0.98 | + |
| Tongue | PFHpA | 0.73 [0.6, 0.89] | 0.00 | 0.96 [0.72, 1.28] | 0.77 | + |
| Other Non Epithelial Skin | PFHpA | 0.65 [0.47, 0.89] | 0.01 | 1.03 [0.72, 1.48] | 0.86 | + |
| Nose Nasal Cavity and Middle Ear | PFOA | 0.6 [0.39, 0.93] | 0.02 | 1.22 [0.78, 1.88] | 0.38 | + |
| Nose Nasal Cavity and Middle Ear | PFHpA | 0.58 [0.35, 0.95] | 0.03 | 1.01 [0.6, 1.68] | 0.98 | + |
| Splenic Flexure | PFHxS | 0.57 [0.34, 0.96] | 0.03 | 0.93 [0.54, 1.6] | 0.80 | + |
| Stomach | PFBS | 0.52 [0.27, 1] | 0.05 | 1.54 [0.84, 2.82] | 0.17 | + |
| Testis | PFBS | 0.3 [0.12, 0.74] | 0.01 | NA | NA | NA |
| Pleura | PFHpA | 1.96 [0.32, 11.79] | 0.46 | 7.15 [1.8, 28.42] | 0.01 | + |
| Pleura | PFOS | 1.4 [0.22, 8.69] | 0.72 | 6.14 [1.73, 21.77] | 0.00 | + |
| Pleura | PFOA | 1.91 [0.41, 8.89] | 0.41 | 5.32 [1.53, 18.45] | 0.01 | + |
| Pleura | PFHxS | 1.76 [0.28, 10.98] | 0.55 | 4.69 [1.31, 16.81] | 0.02 | + |
| Acute Monocytic Leukemia | PFOA | 1.12 [0.55, 2.29] | 0.75 | 3.48 [1.78, 6.82] | 0.00 | + |
| Oropharynx | PFNA | 0.93 [0.4, 2.14] | 0.87 | 2.78 [1.1, 7.02] | 0.03 | + |
| Other Oral Cavity and Pharynx | PFHxS | 0.8 [0.4, 1.6] | 0.54 | 2.74 [1.2, 6.26] | 0.02 | + |
| Cranial Nerves Other Nervous System | PFHpA | 0.81 [0.44, 1.5] | 0.50 | 2.51 [1.51, 4.17] | 0.00 | + |
| Other Oral Cavity and Pharynx | PFHpA | 0.87 [0.47, 1.6] | 0.64 | 2.39 [1.07, 5.35] | 0.03 | + |
| Other Endocrine including Thymus | PFHpA | 1 [0.63, 1.57] | 0.99 | 2.28 [1.5, 3.49] | 0.00 | + |
| Hypopharynx | PFHxS | 0.72 [0.44, 1.18] | 0.19 | 2.25 [1.19, 4.27] | 0.01 | + |
| Oropharynx | PFHxS | 0.87 [0.57, 1.33] | 0.52 | 2.15 [1.18, 3.92] | 0.01 | + |
| Cranial Nerves Other Nervous System | PFOA | 1.26 [0.78, 2.04] | 0.35 | 2.14 [1.34, 3.42] | 0.00 | + |
| Other Endocrine including Thymus | PFHxS | 0.63 [0.35, 1.12] | 0.12 | 2.06 [1.28, 3.31] | 0.00 | + |
| Hypopharynx | PFOS | 0.79 [0.51, 1.21] | 0.28 | 2.03 [1.11, 3.73] | 0.02 | + |
| Floor of Mouth | PFHpA | 0.9 [0.55, 1.47] | 0.68 | 1.9 [1.1, 3.3] | 0.02 | + |
| Oropharynx | PFHpA | 0.8 [0.54, 1.18] | 0.26 | 1.8 [1.02, 3.19] | 0.04 | + |
| Oropharynx | PFOS | 0.88 [0.6, 1.29] | 0.52 | 1.79 [1, 3.21] | 0.05 | + |
| Oropharynx | PFOA | 0.78 [0.55, 1.11] | 0.16 | 1.77 [1.05, 2.98] | 0.03 | + |
| Other Endocrine including Thymus | PFOS | 1.07 [0.68, 1.7] | 0.77 | 1.73 [1.1, 2.7] | 0.02 | + |
| Other Digestive Organs | PFOA | 1.1 [0.74, 1.61] | 0.64 | 1.7 [1.18, 2.45] | 0.00 | + |
| Ureter | PFOA | 0.85 [0.57, 1.29] | 0.45 | 1.6 [1.02, 2.5] | 0.04 | + |
| Descending Colon | PFOA | 0.96 [0.73, 1.27] | 0.77 | 1.47 [1.1, 1.97] | 0.01 | + |
| Descending Colon | PFHpA | 0.91 [0.67, 1.25] | 0.57 | 1.46 [1.05, 2.02] | 0.02 | + |
| Acute Myeloid Leukemia | PFHxS | 1.15 [0.92, 1.42] | 0.21 | 1.43 [1.11, 1.86] | 0.01 | + |
| Large Intestine NOS | PFHxS | 0.89 [0.6, 1.32] | 0.55 | 1.4 [1.01, 1.95] | 0.05 | + |
| Myeloid and Monocytic Leukemia | PFHxS | 1.11 [0.92, 1.35] | 0.26 | 1.27 [1.01, 1.59] | 0.04 | + |
| Oral Cavity and Pharynx | PFOA | 0.96 [0.86, 1.07] | 0.46 | 1.2 [1.03, 1.39] | 0.02 | + |
| Breast | PFHpA | 0.85 [0.56, 1.28] | 0.43 | 0.94 [0.88, 1] | 0.05 | + |
| Rectum | PFOA | 1.02 [0.89, 1.17] | 0.75 | 0.85 [0.73, 1] | 0.04 | + |
| Skin excluding Basal and Squamous | PFOA | 1.08 [0.96, 1.22] | 0.20 | 0.85 [0.73, 0.98] | 0.03 | + |
| Melanoma of the Skin | PFOA | 1.09 [0.96, 1.24] | 0.19 | 0.84 [0.72, 0.98] | 0.03 | + |
| Rectum and Rectosigmoid Junction | PFOA | 1.02 [0.91, 1.16] | 0.72 | 0.84 [0.72, 0.97] | 0.02 | + |
| Melanoma of the Skin | PFHpA | 0.96 [0.83, 1.11] | 0.58 | 0.84 [0.7, 0.99] | 0.04 | + |
| Stomach | PFHpA | 0.84 [0.68, 1.04] | 0.11 | 0.78 [0.61, 0.99] | 0.04 | + |
| Corpus Uteri | PFBS | NA | NA | 0.68 [0.48, 0.95] | 0.02 | NA |
| Corpus and Uterus NOS | PFBS | NA | NA | 0.66 [0.47, 0.93] | 0.02 | NA |
| Nose Nasal Cavity and Middle Ear | PFBS | 0.51 [0.11, 2.28] | 0.38 | 2.97 [1, 8.85] | 0.05 | - |
| Skin excluding Basal and Squamous | PFHpA | 0.93 [0.81, 1.07] | 0.33 | 0.85 [0.72, 1] | 0.05 | - |
| Trachea Mediastinum and Other Respiratory Organs | PFHpA | 1.58 [0.8, 3.09] | 0.18 | 2.25 [0.99, 5.13] | 0.05 | - |
| Other Endocrine including Thymus | PFOA | 1.08 [0.73, 1.62] | 0.69 | 1.48 [0.98, 2.23] | 0.06 | - |
| Other Acute Leukemia | PFNA | 0.5 [0.07, 3.54] | 0.48 | 3.13 [0.95, 10.34] | 0.06 | - |
| Larynx | PFHpA | 1.05 [0.85, 1.29] | 0.68 | 0.69 [0.47, 1.02] | 0.06 | - |
| Oral Cavity and Pharynx | PFHpA | 0.9 [0.8, 1.01] | 0.08 | 1.17 [0.99, 1.39] | 0.06 | - |
| Cervix Uteri | PFBS | NA | NA | 1.73 [0.96, 3.12] | 0.07 | NA |
| Myeloma | PFOA | 1.09 [0.94, 1.26] | 0.27 | 0.84 [0.7, 1.01] | 0.07 | - |
| Pancreas | PFOA | 1 [0.89, 1.12] | 0.96 | 0.9 [0.8, 1.01] | 0.07 | - |
| Salivary Gland | PFOA | 0.97 [0.73, 1.29] | 0.83 | 1.36 [0.97, 1.91] | 0.07 | - |
| Hypopharynx | PFHpA | 0.87 [0.57, 1.33] | 0.51 | 1.77 [0.95, 3.3] | 0.07 | - |
| Soft Tissue including Heart | PFOS | 1.01 [0.77, 1.32] | 0.96 | 1.28 [0.98, 1.69] | 0.07 | - |
| Large Intestine NOS | PFOA | 0.96 [0.71, 1.31] | 0.80 | 1.28 [0.97, 1.68] | 0.08 | - |
| Other Biliary | PFHpA | 0.93 [0.67, 1.28] | 0.64 | 1.32 [0.97, 1.8] | 0.08 | - |
| Endocrine System | PFBS | 1.21 [0.69, 2.13] | 0.50 | 0.66 [0.41, 1.05] | 0.08 | - |
| Other Female Genital Organs | PFBS | NA | NA | 1.97 [0.92, 4.2] | 0.08 | NA |
| Gum and Other Mouth | PFOA | 0.92 [0.71, 1.21] | 0.56 | 1.28 [0.97, 1.7] | 0.08 | - |
| Large Intestine NOS | PFHpA | 1.14 [0.81, 1.6] | 0.46 | 1.31 [0.97, 1.77] | 0.08 | - |
| Rectum and Rectosigmoid Junction | PFNA | 0.97 [0.71, 1.31] | 0.83 | 0.71 [0.49, 1.04] | 0.08 | - |
| Other Oral Cavity and Pharynx | PFOS | 0.74 [0.4, 1.39] | 0.35 | 2.05 [0.92, 4.61] | 0.08 | - |
| Acute Lymphocytic Leukemia | PFNA | 0.6 [0.26, 1.37] | 0.23 | 1.93 [0.92, 4.06] | 0.08 | - |
| Tongue | PFOA | 1.06 [0.9, 1.24] | 0.51 | 1.24 [0.97, 1.59] | 0.08 | - |
| Thyroid | PFBS | 1.29 [0.71, 2.35] | 0.41 | 0.65 [0.4, 1.06] | 0.08 | - |
| Aleukemic Subleukemic and NOS | PFOS | 1.08 [0.66, 1.78] | 0.75 | 1.5 [0.94, 2.4] | 0.09 | - |
| Other Digestive Organs | PFHxS | 1.33 [0.84, 2.11] | 0.22 | 1.49 [0.94, 2.35] | 0.09 | - |
| Acute Myeloid Leukemia | PFHpA | 1.05 [0.86, 1.28] | 0.64 | 1.23 [0.97, 1.57] | 0.09 | - |
| Rectosigmoid Junction | PFOA | 1.03 [0.82, 1.3] | 0.80 | 0.78 [0.58, 1.04] | 0.09 | - |
| Uterus NOS | PFNA | NA | NA | 1.96 [0.89, 4.29] | 0.09 | NA |
| Ovary | PFOA | NA | NA | 1.13 [0.98, 1.29] | 0.09 | NA |
| Chronic Lymphocytic Leukemia | PFHxS | 1.09 [0.85, 1.39] | 0.51 | 0.8 [0.61, 1.04] | 0.09 | - |
| Breast | PFOS | 1.01 [0.68, 1.52] | 0.95 | 0.95 [0.89, 1.01] | 0.10 | - |
| Bones and Joints | PFHxS | 0.97 [0.62, 1.53] | 0.91 | 0.63 [0.37, 1.09] | 0.10 | - |
| Hepatic Flexure | PFOS | 1.2 [0.85, 1.69] | 0.30 | 1.33 [0.94, 1.87] | 0.10 | - |
| Peritoneum Omentum and Mesentery | PFHpA | 0.22 [0.03, 1.64] | 0.14 | 0.66 [0.4, 1.09] | 0.10 | - |
| Esophagus | PFHxS | 0.92 [0.76, 1.1] | 0.35 | 0.74 [0.52, 1.07] | 0.11 | - |
| Skin excluding Basal and Squamous | PFHxS | 0.89 [0.76, 1.03] | 0.12 | 0.86 [0.71, 1.03] | 0.11 | - |
| Other Urinary Organs | PFHpA | 0.64 [0.36, 1.12] | 0.12 | 0.5 [0.21, 1.18] | 0.11 | - |
| Skin excluding Basal and Squamous | PFNA | 1.09 [0.81, 1.47] | 0.56 | 0.74 [0.51, 1.08] | 0.11 | - |
| Esophagus | PFHpA | 1.01 [0.85, 1.19] | 0.95 | 0.77 [0.56, 1.07] | 0.12 | - |
| Lung and Bronchus | PFHxS | 1.02 [0.9, 1.15] | 0.80 | 0.9 [0.8, 1.03] | 0.12 | - |
| Larynx | PFOA | 1.07 [0.88, 1.29] | 0.50 | 1.28 [0.94, 1.74] | 0.12 | - |
| Other Urinary Organs | PFOS | 0.75 [0.44, 1.27] | 0.28 | 0.5 [0.21, 1.2] | 0.12 | - |
| Salivary Gland | PFNA | 0.97 [0.48, 1.96] | 0.93 | 0.41 [0.13, 1.26] | 0.12 | - |
| Other Biliary | PFNA | 0.84 [0.41, 1.72] | 0.63 | 0.44 [0.16, 1.24] | 0.12 | - |
| Breast | PFOA | 0.99 [0.69, 1.41] | 0.94 | 0.96 [0.91, 1.01] | 0.12 | - |
| Melanoma of the Skin | PFHxS | 0.9 [0.77, 1.06] | 0.21 | 0.86 [0.71, 1.04] | 0.12 | - |
| Large Intestine NOS | PFOS | 0.9 [0.63, 1.27] | 0.54 | 1.27 [0.94, 1.73] | 0.12 | - |
| Respiratory System | PFHxS | 1.01 [0.9, 1.14] | 0.87 | 0.91 [0.8, 1.03] | 0.13 | - |
| Sigmoid Colon | PFOA | 0.96 [0.81, 1.14] | 0.64 | 0.87 [0.72, 1.04] | 0.13 | - |
| Gum and Other Mouth | PFHpA | 0.94 [0.7, 1.27] | 0.69 | 1.28 [0.93, 1.76] | 0.13 | - |
| Stomach | PFHxS | 0.79 [0.63, 1] | 0.05 | 0.81 [0.62, 1.06] | 0.13 | - |
| Kidney and Renal Pelvis | PFNA | 0.98 [0.77, 1.23] | 0.84 | 1.27 [0.93, 1.75] | 0.13 | - |
| Vagina | PFHpA | NA | NA | 1.45 [0.89, 2.37] | 0.13 | NA |
| Rectum | PFNA | 0.93 [0.66, 1.3] | 0.67 | 0.73 [0.49, 1.1] | 0.13 | - |
| Brain | PFBS | 0.96 [0.54, 1.68] | 0.88 | 0.59 [0.29, 1.19] | 0.14 | - |
| Descending Colon | PFBS | 1 [0.4, 2.52] | 0.99 | 1.94 [0.8, 4.69] | 0.14 | - |
| Brain and Other Nervous System | PFBS | 0.92 [0.54, 1.6] | 0.78 | 0.59 [0.3, 1.2] | 0.14 | - |
| Other Leukemia | PFNA | 0.49 [0.15, 1.65] | 0.25 | 1.85 [0.81, 4.24] | 0.15 | - |
| Chronic Lymphocytic Leukemia | PFBS | 0.86 [0.44, 1.69] | 0.66 | 1.54 [0.86, 2.74] | 0.15 | - |
| Melanoma of the Skin | PFNA | 1.1 [0.8, 1.51] | 0.55 | 0.75 [0.51, 1.11] | 0.15 | - |
| Cecum | PFHpA | 1.2 [0.99, 1.46] | 0.07 | 0.87 [0.71, 1.05] | 0.15 | - |
| Soft Tissue including Heart | PFHxS | 0.93 [0.69, 1.25] | 0.63 | 1.25 [0.92, 1.69] | 0.15 | - |
| Hypopharynx | PFOA | 0.91 [0.63, 1.32] | 0.63 | 0.56 [0.25, 1.24] | 0.15 | - |
| Stomach | PFOS | 0.84 [0.69, 1.04] | 0.11 | 0.84 [0.66, 1.07] | 0.16 | - |
| Chronic Myeloid Leukemia | PFOA | 1.04 [0.8, 1.35] | 0.76 | 0.79 [0.57, 1.09] | 0.16 | - |
| Large Intestine NOS | PFBS | 0.57 [0.2, 1.67] | 0.31 | 0.41 [0.12, 1.4] | 0.16 | - |
| Lymphocytic Leukemia | PFHxS | 1.08 [0.87, 1.35] | 0.47 | 0.84 [0.66, 1.07] | 0.16 | - |
| Melanoma of the Skin | PFOS | 1.03 [0.89, 1.19] | 0.68 | 0.88 [0.74, 1.05] | 0.16 | - |
| Ascending Colon | PFNA | 1.12 [0.73, 1.7] | 0.61 | 1.32 [0.89, 1.95] | 0.17 | - |
| Trachea Mediastinum and Other Respiratory Organs | PFOA | 1.52 [0.83, 2.76] | 0.17 | 1.74 [0.79, 3.84] | 0.17 | - |
| Other Oral Cavity and Pharynx | PFOA | 0.65 [0.36, 1.16] | 0.15 | 1.73 [0.79, 3.79] | 0.17 | - |
| Other Myeloid Monocytic Leukemia | PFNA | 0.51 [0.03, 7.58] | 0.62 | 2.9 [0.63, 13.4] | 0.17 | - |
| Colon and Rectum | PFOA | 1 [0.92, 1.08] | 0.91 | 0.94 [0.86, 1.03] | 0.18 | - |
| Liver | PFOA | 1.04 [0.89, 1.21] | 0.66 | 1.17 [0.93, 1.46] | 0.18 | - |
| Urinary Bladder | PFOA | 1.06 [0.95, 1.18] | 0.30 | 0.9 [0.78, 1.05] | 0.18 | - |
| Myeloid and Monocytic Leukemia | PFBS | 1.32 [0.82, 2.11] | 0.25 | 0.63 [0.32, 1.24] | 0.18 | - |
| Acute Monocytic Leukemia | PFHxS | 0.94 [0.36, 2.42] | 0.90 | 0.4 [0.1, 1.53] | 0.18 | - |
| Lung and Bronchus | PFNA | 0.95 [0.74, 1.2] | 0.65 | 1.18 [0.92, 1.51] | 0.18 | - |
| Gallbladder | PFNA | 1.22 [0.5, 2.97] | 0.66 | 0.49 [0.17, 1.4] | 0.19 | - |
| Bones and Joints | PFHpA | 0.9 [0.59, 1.36] | 0.61 | 0.73 [0.45, 1.17] | 0.19 | - |
| Rectum and Rectosigmoid Junction | PFHpA | 1.05 [0.92, 1.21] | 0.48 | 0.9 [0.76, 1.05] | 0.19 | - |
| Liver and Intrahepatic Bile Duct | PFOA | 1.05 [0.91, 1.21] | 0.53 | 1.14 [0.94, 1.37] | 0.19 | - |
| Acute Lymphocytic Leukemia | PFBS | 0.72 [0.24, 2.18] | 0.56 | 0.38 [0.09, 1.64] | 0.19 | - |
| Rectum | PFHpA | 1.06 [0.91, 1.23] | 0.44 | 0.89 [0.75, 1.06] | 0.19 | - |
| Skin excluding Basal and Squamous | PFOS | 1.02 [0.89, 1.17] | 0.79 | 0.9 [0.76, 1.06] | 0.20 | - |
| Respiratory System | PFNA | 0.93 [0.74, 1.18] | 0.56 | 1.17 [0.92, 1.5] | 0.20 | - |
| Chronic Myeloid Leukemia | PFBS | 1.08 [0.47, 2.47] | 0.85 | 0.43 [0.12, 1.55] | 0.20 | - |
| Other Urinary Organs | PFHxS | 0.65 [0.35, 1.2] | 0.17 | 0.54 [0.21, 1.38] | 0.20 | - |
| Aleukemic Subleukemic and NOS | PFOA | 0.74 [0.46, 1.19] | 0.22 | 1.32 [0.86, 2.02] | 0.20 | - |
| Lung and Bronchus | PFOS | 1.05 [0.94, 1.17] | 0.40 | 0.93 [0.83, 1.04] | 0.20 | - |
| Hepatic Flexure | PFHpA | 1.18 [0.84, 1.67] | 0.34 | 1.26 [0.89, 1.78] | 0.20 | - |
| Soft Tissue including Heart | PFOA | 1.01 [0.79, 1.28] | 0.96 | 1.17 [0.92, 1.5] | 0.20 | - |
| Hodgkin Nodal | PFOS | 1.18 [0.88, 1.58] | 0.26 | 1.24 [0.89, 1.74] | 0.21 | - |
| All Sites | PFHxS | 1 [0.95, 1.05] | 0.93 | 0.97 [0.92, 1.02] | 0.21 | - |
| Sigmoid Colon | PFHxS | 0.94 [0.76, 1.17] | 0.60 | 0.86 [0.69, 1.08] | 0.21 | - |
| Breast | PFHxS | 1.19 [0.77, 1.83] | 0.43 | 0.96 [0.89, 1.02] | 0.21 | - |
| Hodgkin Lymphoma | PFOS | 1.16 [0.87, 1.55] | 0.32 | 1.24 [0.88, 1.73] | 0.21 | - |
| Corpus and Uterus NOS | PFNA | NA | NA | 1.16 [0.92, 1.46] | 0.21 | NA |
| Salivary Gland | PFHpA | 1.12 [0.82, 1.53] | 0.49 | 1.27 [0.87, 1.85] | 0.21 | - |
| Uterus NOS | PFBS | NA | NA | 0.29 [0.04, 2.09] | 0.22 | NA |
| Salivary Gland | PFOS | 1.06 [0.77, 1.45] | 0.74 | 0.77 [0.5, 1.17] | 0.22 | - |
| Myeloid and Monocytic Leukemia | PFOS | 1.13 [0.95, 1.34] | 0.16 | 1.14 [0.92, 1.4] | 0.22 | - |
| Other Non Epithelial Skin | PFNA | 1.01 [0.54, 1.88] | 0.98 | 0.55 [0.21, 1.44] | 0.22 | - |
| Pancreas | PFOS | 1.02 [0.9, 1.16] | 0.76 | 0.92 [0.81, 1.05] | 0.23 | - |
| Aleukemic Subleukemic and NOS | PFHxS | 1.07 [0.62, 1.86] | 0.80 | 1.38 [0.82, 2.34] | 0.23 | - |
| Hodgkin Lymphoma | PFBS | 1.31 [0.59, 2.9] | 0.51 | 1.74 [0.71, 4.28] | 0.23 | - |
| Appendix | PFOS | 1.33 [0.95, 1.86] | 0.10 | 0.8 [0.56, 1.15] | 0.23 | - |
| Ureter | PFBS | 0.41 [0.06, 2.98] | 0.38 | 2.15 [0.62, 7.51] | 0.23 | - |
| Other Biliary | PFHxS | 0.84 [0.58, 1.2] | 0.34 | 1.24 [0.87, 1.75] | 0.23 | - |
| Other Leukemia | PFBS | 1.55 [0.52, 4.59] | 0.43 | 0.31 [0.05, 2.12] | 0.23 | - |
| Cecum | PFOA | 1.11 [0.93, 1.32] | 0.26 | 0.9 [0.76, 1.07] | 0.23 | - |
| Vagina | PFNA | NA | NA | 1.83 [0.68, 4.97] | 0.23 | NA |
| Urinary System | PFOA | 1.07 [0.99, 1.15] | 0.11 | 0.94 [0.85, 1.04] | 0.24 | - |
| Soft Tissue including Heart | PFBS | 1.85 [0.92, 3.74] | 0.09 | 0.56 [0.22, 1.46] | 0.24 | - |
| Transverse Colon | PFBS | 1.15 [0.55, 2.4] | 0.71 | 0.57 [0.22, 1.47] | 0.24 | - |
| Other Acute Leukemia | PFOS | 0.83 [0.39, 1.8] | 0.64 | 0.6 [0.25, 1.43] | 0.25 | - |
| Other Digestive Organs | PFNA | 0.69 [0.23, 2.02] | 0.50 | 1.64 [0.71, 3.78] | 0.25 | - |
| Hodgkin Nodal | PFBS | 1.32 [0.59, 2.93] | 0.50 | 1.7 [0.69, 4.24] | 0.25 | - |
| Other Myeloid Monocytic Leukemia | PFHpA | 1.17 [0.47, 2.91] | 0.73 | 0.43 [0.1, 1.84] | 0.26 | - |
| All Sites | PFOA | 1.03 [0.99, 1.07] | 0.21 | 0.98 [0.94, 1.02] | 0.26 | - |
| Ascending Colon | PFOS | 0.92 [0.75, 1.12] | 0.40 | 1.11 [0.92, 1.35] | 0.26 | - |
| Rectosigmoid Junction | PFNA | 1.1 [0.63, 1.93] | 0.74 | 0.64 [0.3, 1.39] | 0.26 | - |
| Intrahepatic Bile Duct | PFNA | 1.12 [0.57, 2.23] | 0.74 | 0.61 [0.26, 1.45] | 0.26 | - |
| Appendix | PFHpA | 1.01 [0.71, 1.44] | 0.94 | 0.82 [0.57, 1.16] | 0.26 | - |
| Digestive System | PFOA | 0.99 [0.93, 1.06] | 0.86 | 0.96 [0.9, 1.03] | 0.27 | - |
| Aleukemic Subleukemic and NOS | PFHpA | 0.83 [0.48, 1.44] | 0.52 | 1.31 [0.81, 2.11] | 0.27 | - |
| Gallbladder | PFBS | 1 [0.27, 3.77] | 1.00 | 0.48 [0.13, 1.77] | 0.27 | - |
| Vulva | PFHxS | NA | NA | 1.16 [0.89, 1.53] | 0.27 | NA |
| Urinary System | PFBS | 0.97 [0.75, 1.26] | 0.82 | 0.83 [0.6, 1.16] | 0.27 | - |
| Respiratory System | PFOS | 1.05 [0.94, 1.17] | 0.36 | 0.94 [0.84, 1.05] | 0.28 | - |
| Peritoneum Omentum and Mesentery | PFHxS | 0.19 [0.02, 2.24] | 0.19 | 0.75 [0.45, 1.26] | 0.28 | - |
| Pleura | PFNA | 1.05 [0.01, 84.84] | 0.98 | 2.82 [0.42, 18.85] | 0.28 | - |
| Appendix | PFBS | 0.98 [0.34, 2.81] | 0.97 | 0.53 [0.16, 1.7] | 0.28 | - |
| Splenic Flexure | PFOA | 0.81 [0.56, 1.17] | 0.26 | 0.78 [0.49, 1.23] | 0.29 | - |
| Female Genital System | PFHpA | NA | NA | 1.05 [0.96, 1.14] | 0.29 | NA |
| Other Urinary Organs | PFNA | 0.82 [0.27, 2.52] | 0.73 | 0.2 [0.01, 3.97] | 0.29 | - |
| Corpus Uteri | PFNA | NA | NA | 1.13 [0.9, 1.43] | 0.29 | NA |
| Gallbladder | PFOA | 1.17 [0.8, 1.71] | 0.41 | 0.83 [0.59, 1.17] | 0.30 | - |
| Ascending Colon | PFBS | 1.49 [0.9, 2.47] | 0.12 | 1.31 [0.78, 2.2] | 0.30 | - |
| Leukemia | PFOS | 1.02 [0.89, 1.18] | 0.74 | 1.08 [0.93, 1.25] | 0.30 | - |
| Liver | PFHpA | 1.11 [0.93, 1.31] | 0.24 | 1.14 [0.89, 1.48] | 0.30 | - |
| Liver and Intrahepatic Bile Duct | PFOS | 1 [0.85, 1.17] | 0.98 | 0.89 [0.71, 1.11] | 0.30 | - |
| Lymphocytic Leukemia | PFNA | 1.04 [0.68, 1.6] | 0.86 | 1.26 [0.81, 1.95] | 0.31 | - |
| Bones and Joints | PFNA | 0.76 [0.28, 2.01] | 0.57 | 0.55 [0.17, 1.77] | 0.32 | - |
| Esophagus | PFOS | 0.98 [0.83, 1.15] | 0.80 | 0.85 [0.62, 1.16] | 0.32 | - |
| Leukemia | PFNA | 0.99 [0.72, 1.35] | 0.93 | 1.18 [0.86, 1.62] | 0.32 | - |
| Female Genital System | PFNA | NA | NA | 1.1 [0.91, 1.33] | 0.32 | NA |
| Peritoneum Omentum and Mesentery | PFOS | 0.35 [0.07, 1.83] | 0.21 | 0.79 [0.5, 1.25] | 0.32 | - |
| Urinary Bladder | PFBS | 1 [0.71, 1.42] | 0.99 | 0.77 [0.46, 1.29] | 0.32 | - |
| Rectum and Rectosigmoid Junction | PFBS | 0.91 [0.61, 1.35] | 0.63 | 0.78 [0.48, 1.27] | 0.32 | - |
| Urinary System | PFNA | 1.01 [0.83, 1.23] | 0.94 | 1.13 [0.89, 1.43] | 0.33 | - |
| Soft Tissue including Heart | PFHpA | 0.84 [0.64, 1.11] | 0.22 | 1.15 [0.87, 1.52] | 0.33 | - |
| Floor of Mouth | PFOS | 1.19 [0.75, 1.88] | 0.46 | 1.34 [0.75, 2.39] | 0.33 | - |
| Leukemia | PFHpA | 0.94 [0.81, 1.08] | 0.36 | 1.08 [0.93, 1.25] | 0.33 | - |
| Vulva | PFOA | NA | NA | 0.89 [0.71, 1.12] | 0.34 | NA |
| Trachea Mediastinum and Other Respiratory Organs | PFBS | 0.19 [0, 12.68] | 0.44 | 2.7 [0.35, 21.02] | 0.34 | - |
| Appendix | PFOA | 0.79 [0.57, 1.09] | 0.15 | 0.86 [0.63, 1.18] | 0.35 | - |
| Other Leukemia | PFOA | 0.79 [0.53, 1.18] | 0.25 | 1.2 [0.82, 1.76] | 0.35 | - |
| Other Acute Leukemia | PFHpA | 1.49 [0.77, 2.87] | 0.24 | 0.63 [0.24, 1.64] | 0.35 | - |
| Corpus Uteri | PFHpA | NA | NA | 1.05 [0.94, 1.18] | 0.35 | NA |
| Hypopharynx | PFNA | 0.83 [0.32, 2.16] | 0.70 | 0.2 [0.01, 5.81] | 0.35 | - |
| Pancreas | PFHpA | 1.04 [0.92, 1.18] | 0.54 | 0.94 [0.83, 1.07] | 0.35 | - |
| Other Leukemia | PFHxS | 1.01 [0.62, 1.63] | 0.98 | 1.25 [0.78, 1.99] | 0.36 | - |
| Transverse Colon | PFHpA | 0.81 [0.61, 1.07] | 0.14 | 1.14 [0.87, 1.49] | 0.36 | - |
| Other Leukemia | PFOS | 1.01 [0.65, 1.56] | 0.96 | 1.22 [0.8, 1.87] | 0.36 | - |
| All Sites | PFOS | 1.02 [0.98, 1.07] | 0.34 | 0.98 [0.94, 1.02] | 0.36 | - |
| Corpus and Uterus NOS | PFHpA | NA | NA | 1.05 [0.94, 1.17] | 0.37 | NA |
| Soft Tissue including Heart | PFNA | 0.98 [0.55, 1.76] | 0.95 | 0.73 [0.38, 1.44] | 0.37 | - |
| Tonsil | PFOA | 0.96 [0.78, 1.18] | 0.71 | 0.82 [0.53, 1.27] | 0.38 | - |
| Digestive System | PFHxS | 0.98 [0.91, 1.06] | 0.66 | 0.96 [0.89, 1.05] | 0.38 | - |
| Liver | PFOS | 1.01 [0.85, 1.2] | 0.89 | 0.89 [0.68, 1.16] | 0.38 | - |
| Intrahepatic Bile Duct | PFHpA | 0.99 [0.72, 1.38] | 0.96 | 0.85 [0.6, 1.21] | 0.38 | - |
| NHL Extranodal | PFNA | 0.77 [0.49, 1.19] | 0.23 | 0.81 [0.51, 1.29] | 0.38 | - |
| Digestive System | PFHpA | 1.02 [0.95, 1.09] | 0.61 | 0.97 [0.9, 1.04] | 0.38 | - |
| Sigmoid Colon | PFBS | 1.25 [0.74, 2.12] | 0.40 | 0.76 [0.41, 1.41] | 0.38 | - |
| Non Hodgkin Lymphoma | PFOA | 1.1 [1, 1.21] | 0.06 | 0.94 [0.82, 1.08] | 0.39 | - |
| Liver and Intrahepatic Bile Duct | PFHxS | 1.08 [0.9, 1.29] | 0.41 | 0.9 [0.7, 1.15] | 0.39 | - |
| Acute Monocytic Leukemia | PFNA | 0.81 [0.11, 5.77] | 0.83 | 0.23 [0.01, 6.51] | 0.39 | - |
| Cranial Nerves Other Nervous System | PFNA | 1.21 [0.39, 3.72] | 0.74 | 0.53 [0.12, 2.29] | 0.39 | - |
| Uterus NOS | PFOA | NA | NA | 1.18 [0.81, 1.71] | 0.40 | NA |
| Hodgkin Nodal | PFHxS | 1.11 [0.8, 1.53] | 0.54 | 1.18 [0.81, 1.72] | 0.40 | - |
| Rectosigmoid Junction | PFHxS | 1.02 [0.76, 1.37] | 0.89 | 0.86 [0.6, 1.23] | 0.40 | - |
| Acute Monocytic Leukemia | PFHpA | 0.76 [0.31, 1.89] | 0.56 | 0.65 [0.23, 1.8] | 0.40 | - |
| Kidney and Renal Pelvis | PFOA | 1.09 [0.99, 1.19] | 0.08 | 0.94 [0.83, 1.08] | 0.41 | - |
| NHL Nodal | PFOA | 1.05 [0.94, 1.18] | 0.37 | 0.93 [0.79, 1.1] | 0.41 | - |
| Endocrine System | PFOA | 1.04 [0.87, 1.25] | 0.64 | 1.06 [0.92, 1.21] | 0.41 | - |
| Ovary | PFHxS | NA | NA | 1.08 [0.9, 1.28] | 0.41 | NA |
| Hodgkin Nodal | PFOA | 1.06 [0.81, 1.38] | 0.69 | 1.14 [0.84, 1.54] | 0.41 | - |
| Floor of Mouth | PFNA | 0.73 [0.23, 2.32] | 0.60 | 0.43 [0.06, 3.18] | 0.41 | - |
| Gum and Other Mouth | PFBS | 0.71 [0.26, 1.94] | 0.51 | 1.44 [0.6, 3.44] | 0.41 | - |
| Rectum and Rectosigmoid Junction | PFHxS | 0.99 [0.85, 1.16] | 0.91 | 0.93 [0.77, 1.11] | 0.41 | - |
| Rectosigmoid Junction | PFOS | 1 [0.77, 1.3] | 0.99 | 0.87 [0.63, 1.21] | 0.41 | - |
| Leukemia | PFHxS | 1.1 [0.94, 1.28] | 0.24 | 1.07 [0.91, 1.26] | 0.42 | - |
| All Sites | PFHpA | 1 [0.95, 1.05] | 1.00 | 0.98 [0.94, 1.03] | 0.42 | - |
| Hodgkin Lymphoma | PFHxS | 1.09 [0.79, 1.5] | 0.62 | 1.17 [0.8, 1.7] | 0.42 | - |
| Myeloid and Monocytic Leukemia | PFHpA | 1.02 [0.85, 1.21] | 0.85 | 1.09 [0.88, 1.35] | 0.42 | - |
| Pancreas | PFBS | 1.13 [0.79, 1.62] | 0.51 | 0.85 [0.58, 1.26] | 0.42 | - |
| Hodgkin Lymphoma | PFOA | 1.04 [0.8, 1.35] | 0.78 | 1.13 [0.84, 1.53] | 0.43 | - |
| Kidney and Renal Pelvis | PFHpA | 1.11 [1, 1.23] | 0.06 | 0.94 [0.81, 1.09] | 0.43 | - |
| Aleukemic Subleukemic and NOS | PFBS | 2.04 [0.65, 6.37] | 0.22 | 0.44 [0.06, 3.36] | 0.43 | - |
| Breast | PFNA | 1.71 [0.78, 3.71] | 0.18 | 0.95 [0.83, 1.08] | 0.43 | - |
| Small Intestine | PFHpA | 0.91 [0.7, 1.18] | 0.49 | 0.89 [0.66, 1.19] | 0.43 | - |
| Rectum | PFBS | 0.87 [0.56, 1.34] | 0.53 | 0.81 [0.48, 1.37] | 0.43 | - |
| Hepatic Flexure | PFOA | 1.15 [0.85, 1.57] | 0.36 | 1.13 [0.83, 1.56] | 0.44 | - |
| Rectosigmoid Junction | PFBS | 1.07 [0.51, 2.24] | 0.86 | 0.67 [0.24, 1.84] | 0.44 | - |
| Endocrine System | PFHpA | 1.07 [0.88, 1.31] | 0.51 | 1.06 [0.91, 1.24] | 0.44 | - |
| Gum and Other Mouth | PFNA | 0.99 [0.52, 1.87] | 0.97 | 0.72 [0.32, 1.64] | 0.44 | - |
| Corpus Uteri | PFOA | NA | NA | 0.96 [0.87, 1.06] | 0.44 | NA |
| Acute Myeloid Leukemia | PFBS | 1.33 [0.77, 2.27] | 0.30 | 0.74 [0.34, 1.6] | 0.44 | - |
| Other Non Epithelial Skin | PFHxS | 0.72 [0.51, 1.01] | 0.06 | 0.85 [0.56, 1.29] | 0.44 | - |
| Female Genital System | PFHxS | NA | NA | 1.04 [0.94, 1.14] | 0.45 | NA |
| Tongue | PFNA | 0.99 [0.66, 1.49] | 0.98 | 1.25 [0.7, 2.25] | 0.45 | - |
| Salivary Gland | PFHxS | 1.15 [0.82, 1.62] | 0.42 | 0.84 [0.53, 1.33] | 0.45 | - |
| Oral Cavity and Pharynx | PFHxS | 0.93 [0.82, 1.07] | 0.31 | 1.08 [0.89, 1.3] | 0.45 | - |
| Kidney and Renal Pelvis | PFBS | 0.94 [0.69, 1.28] | 0.70 | 0.84 [0.54, 1.31] | 0.45 | - |
| Other Acute Leukemia | PFBS | 0.35 [0.01, 8.5] | 0.52 | 0.11 [0, 35.77] | 0.45 | - |
| Larynx | PFBS | 1.57 [0.9, 2.73] | 0.11 | 1.46 [0.54, 3.98] | 0.46 | - |
| Chronic Myeloid Leukemia | PFOS | 0.93 [0.69, 1.25] | 0.64 | 1.14 [0.81, 1.59] | 0.46 | - |
| Brain and Other Nervous System | PFOA | 1.11 [0.94, 1.31] | 0.24 | 1.07 [0.89, 1.28] | 0.46 | - |
| Nasopharynx | PFOA | 0.71 [0.43, 1.18] | 0.19 | 0.77 [0.39, 1.53] | 0.46 | - |
| Anus Anal Canal and Anorectum | PFOS | 1.06 [0.74, 1.52] | 0.75 | 1.1 [0.85, 1.42] | 0.46 | - |
| NHL Extranodal | PFHxS | 0.85 [0.68, 1.05] | 0.14 | 0.92 [0.73, 1.15] | 0.47 | - |
| Chronic Myeloid Leukemia | PFNA | 0.84 [0.43, 1.64] | 0.60 | 1.3 [0.64, 2.61] | 0.47 | - |
| Floor of Mouth | PFOA | 0.71 [0.45, 1.13] | 0.15 | 1.22 [0.71, 2.1] | 0.47 | - |
| Other Digestive Organs | PFOS | 1.35 [0.89, 2.04] | 0.16 | 1.18 [0.76, 1.84] | 0.47 | - |
| Other Myeloid Monocytic Leukemia | PFHxS | 1.01 [0.34, 2.96] | 0.99 | 0.57 [0.12, 2.66] | 0.47 | - |
| Tonsil | PFHpA | 1 [0.79, 1.25] | 0.97 | 0.84 [0.52, 1.36] | 0.47 | - |
| Other Biliary | PFOS | 1.03 [0.75, 1.4] | 0.87 | 1.12 [0.81, 1.55] | 0.48 | - |
| Hodgkin Nodal | PFHpA | 0.97 [0.72, 1.32] | 0.87 | 1.13 [0.8, 1.59] | 0.48 | - |
| Ovary | PFHpA | NA | NA | 1.06 [0.9, 1.24] | 0.48 | NA |
| Urinary System | PFHpA | 1.06 [0.97, 1.16] | 0.23 | 0.96 [0.86, 1.07] | 0.48 | - |
| Hodgkin Nodal | PFNA | 1.08 [0.57, 2.08] | 0.81 | 1.29 [0.63, 2.65] | 0.48 | - |
| Other Female Genital Organs | PFHxS | NA | NA | 1.12 [0.81, 1.56] | 0.49 | NA |
| Peritoneum Omentum and Mesentery | PFNA | 1.25 [0.15, 10.76] | 0.84 | 1.33 [0.59, 2.98] | 0.49 | - |
| Ureter | PFHpA | 0.89 [0.56, 1.41] | 0.61 | 1.21 [0.7, 2.09] | 0.49 | - |
| Retroperitoneum | PFBS | 0.29 [0.02, 4.99] | 0.39 | 0.44 [0.04, 4.52] | 0.49 | - |
| Cecum | PFNA | 0.89 [0.56, 1.39] | 0.60 | 1.15 [0.77, 1.71] | 0.49 | - |
| Anus Anal Canal and Anorectum | PFHpA | 1 [0.69, 1.44] | 0.98 | 1.09 [0.85, 1.41] | 0.49 | - |
| Tonsil | PFBS | 1.48 [0.8, 2.77] | 0.21 | 1.53 [0.46, 5.11] | 0.49 | - |
| Liver | PFHxS | 1.06 [0.87, 1.28] | 0.58 | 0.9 [0.67, 1.21] | 0.50 | - |
| Other Non Epithelial Skin | PFOA | 1 [0.77, 1.28] | 0.97 | 0.89 [0.64, 1.24] | 0.50 | - |
| Leukemia | PFOA | 1.01 [0.89, 1.15] | 0.86 | 1.05 [0.92, 1.2] | 0.50 | - |
| Other Myeloid Monocytic Leukemia | PFOA | 0.91 [0.37, 2.23] | 0.84 | 0.68 [0.23, 2.05] | 0.50 | - |
| Lip | PFNA | 0.52 [0.16, 1.72] | 0.28 | 0.48 [0.06, 4.05] | 0.50 | - |
| Vulva | PFNA | NA | NA | 1.2 [0.71, 2.02] | 0.50 | NA |
| Hodgkin Lymphoma | PFHpA | 1.03 [0.76, 1.39] | 0.84 | 1.12 [0.8, 1.57] | 0.50 | - |
| Nasopharynx | PFBS | 0.97 [0.22, 4.35] | 0.97 | 0.39 [0.02, 6.16] | 0.50 | - |
| Hodgkin Lymphoma | PFNA | 1.07 [0.56, 2.03] | 0.84 | 1.28 [0.62, 2.6] | 0.50 | - |
| Acute Monocytic Leukemia | PFOS | 1.46 [0.68, 3.13] | 0.33 | 0.7 [0.25, 2.01] | 0.51 | - |
| Nose Nasal Cavity and Middle Ear | PFOS | 0.75 [0.48, 1.19] | 0.22 | 1.18 [0.72, 1.94] | 0.51 | - |
| Digestive System | PFOS | 0.99 [0.93, 1.07] | 0.84 | 0.98 [0.91, 1.05] | 0.51 | - |
| Anus Anal Canal and Anorectum | PFBS | 1.42 [0.56, 3.6] | 0.46 | 1.27 [0.62, 2.62] | 0.51 | - |
| Leukemia | PFBS | 1.07 [0.71, 1.61] | 0.74 | 0.86 [0.56, 1.34] | 0.51 | - |
| Cecum | PFBS | 0.93 [0.52, 1.67] | 0.81 | 0.82 [0.46, 1.47] | 0.51 | - |
| Aleukemic Subleukemic and NOS | PFNA | 0.47 [0.11, 2.1] | 0.33 | 1.39 [0.51, 3.77] | 0.51 | - |
| Corpus and Uterus NOS | PFOA | NA | NA | 0.97 [0.88, 1.07] | 0.52 | NA |
| Intrahepatic Bile Duct | PFOS | 0.91 [0.65, 1.27] | 0.56 | 0.89 [0.63, 1.26] | 0.52 | - |
| Ureter | PFHxS | 0.87 [0.52, 1.45] | 0.59 | 1.22 [0.67, 2.22] | 0.52 | - |
| Colon excluding Rectum | PFNA | 0.91 [0.71, 1.18] | 0.49 | 1.08 [0.85, 1.39] | 0.52 | - |
| Ascending Colon | PFHxS | 0.87 [0.69, 1.08] | 0.21 | 1.07 [0.87, 1.32] | 0.53 | - |
| Esophagus | PFBS | 1.39 [0.89, 2.15] | 0.15 | 1.3 [0.58, 2.88] | 0.53 | - |
| Colon and Rectum | PFBS | 1.08 [0.83, 1.41] | 0.58 | 0.91 [0.68, 1.22] | 0.53 | - |
| Sigmoid Colon | PFHpA | 0.99 [0.82, 1.2] | 0.91 | 0.94 [0.76, 1.15] | 0.53 | - |
| Ureter | PFNA | 0.74 [0.25, 2.15] | 0.58 | 0.61 [0.13, 2.86] | 0.53 | - |
| Vagina | PFHxS | NA | NA | 1.2 [0.68, 2.12] | 0.53 | NA |
| Nasopharynx | PFHpA | 0.73 [0.41, 1.29] | 0.28 | 0.78 [0.36, 1.71] | 0.53 | - |
| Lymphocytic Leukemia | PFBS | 0.82 [0.45, 1.48] | 0.51 | 1.2 [0.67, 2.13] | 0.54 | - |
| Transverse Colon | PFOA | 0.93 [0.73, 1.18] | 0.54 | 0.92 [0.72, 1.19] | 0.54 | - |
| Intrahepatic Bile Duct | PFHxS | 1.19 [0.84, 1.69] | 0.32 | 0.89 [0.61, 1.3] | 0.55 | - |
| Liver and Intrahepatic Bile Duct | PFNA | 1.24 [0.88, 1.74] | 0.22 | 0.86 [0.52, 1.42] | 0.55 | - |
| Nasopharynx | PFNA | 0.6 [0.15, 2.37] | 0.47 | 1.51 [0.39, 5.84] | 0.55 | - |
| Thyroid | PFOA | 1.04 [0.86, 1.25] | 0.72 | 1.04 [0.91, 1.2] | 0.55 | - |
| Anus Anal Canal and Anorectum | PFNA | 0.94 [0.41, 2.13] | 0.87 | 1.18 [0.69, 2.01] | 0.55 | - |
| Other Leukemia | PFHpA | 1.05 [0.68, 1.63] | 0.81 | 1.14 [0.74, 1.78] | 0.55 | - |
| Larynx | PFHxS | 0.92 [0.72, 1.17] | 0.50 | 0.88 [0.59, 1.33] | 0.55 | - |
| Vagina | PFBS | NA | NA | 0.6 [0.11, 3.27] | 0.56 | NA |
| Hepatic Flexure | PFNA | 0.6 [0.24, 1.51] | 0.28 | 0.77 [0.32, 1.84] | 0.56 | - |
| Brain | PFNA | 0.9 [0.58, 1.41] | 0.65 | 1.14 [0.74, 1.77] | 0.56 | - |
| Oral Cavity and Pharynx | PFOS | 0.94 [0.83, 1.06] | 0.31 | 1.05 [0.89, 1.25] | 0.56 | - |
| Myeloma | PFHpA | 0.87 [0.74, 1.04] | 0.13 | 0.94 [0.77, 1.15] | 0.56 | - |
| Non Hodgkin Lymphoma | PFNA | 1.07 [0.84, 1.36] | 0.57 | 0.91 [0.64, 1.27] | 0.57 | - |
| Appendix | PFHxS | 1.2 [0.83, 1.75] | 0.34 | 0.89 [0.61, 1.32] | 0.57 | - |
| Lymphoma | PFOS | 1.04 [0.93, 1.15] | 0.51 | 1.04 [0.9, 1.21] | 0.57 | - |
| Splenic Flexure | PFBS | 0.57 [0.13, 2.51] | 0.46 | 0.64 [0.14, 3] | 0.57 | - |
| Chronic Lymphocytic Leukemia | PFHpA | 0.83 [0.66, 1.04] | 0.11 | 1.07 [0.85, 1.34] | 0.57 | - |
| Oral Cavity and Pharynx | PFNA | 0.87 [0.67, 1.14] | 0.31 | 0.89 [0.61, 1.32] | 0.57 | - |
| Chronic Myeloid Leukemia | PFHxS | 1.07 [0.77, 1.47] | 0.70 | 1.11 [0.76, 1.62] | 0.58 | - |
| Rectum | PFHxS | 0.98 [0.83, 1.16] | 0.84 | 0.95 [0.78, 1.15] | 0.58 | - |
| Urinary Bladder | PFOS | 1.1 [0.98, 1.24] | 0.11 | 0.95 [0.81, 1.13] | 0.58 | - |
| Other Female Genital Organs | PFOA | NA | NA | 1.08 [0.82, 1.41] | 0.59 | NA |
| Cervix Uteri | PFOA | NA | NA | 1.06 [0.87, 1.28] | 0.59 | NA |
| Rectosigmoid Junction | PFHpA | 1.02 [0.78, 1.32] | 0.91 | 0.92 [0.67, 1.26] | 0.59 | - |
| Respiratory System | PFBS | 1.16 [0.85, 1.57] | 0.34 | 1.09 [0.79, 1.51] | 0.59 | - |
| Acute Myeloid Leukemia | PFOA | 1.12 [0.94, 1.33] | 0.22 | 1.06 [0.85, 1.32] | 0.59 | - |
| Lymphoma | PFOA | 1.09 [0.99, 1.2] | 0.06 | 0.97 [0.85, 1.1] | 0.60 | - |
| Liver and Intrahepatic Bile Duct | PFHpA | 1.1 [0.93, 1.28] | 0.26 | 1.06 [0.85, 1.32] | 0.60 | - |
| Large Intestine NOS | PFNA | 0.63 [0.27, 1.46] | 0.28 | 1.19 [0.61, 2.32] | 0.61 | - |
| Myeloma | PFBS | 1.08 [0.66, 1.75] | 0.76 | 0.86 [0.47, 1.55] | 0.61 | - |
| Cervix Uteri | PFNA | NA | NA | 0.88 [0.54, 1.44] | 0.61 | NA |
| Hypopharynx | PFBS | 0.88 [0.27, 2.91] | 0.84 | 0.45 [0.02, 10.06] | 0.62 | - |
| Transverse Colon | PFNA | 0.78 [0.42, 1.44] | 0.42 | 0.85 [0.45, 1.62] | 0.63 | - |
| Lung and Bronchus | PFOA | 1.05 [0.95, 1.16] | 0.31 | 0.98 [0.88, 1.08] | 0.63 | - |
| Myeloma | PFHxS | 0.89 [0.73, 1.08] | 0.23 | 1.06 [0.85, 1.31] | 0.63 | - |
| Corpus and Uterus NOS | PFHxS | NA | NA | 1.03 [0.91, 1.16] | 0.63 | NA |
| Brain | PFOA | 1.1 [0.92, 1.3] | 0.30 | 0.96 [0.79, 1.15] | 0.63 | - |
| Descending Colon | PFOS | 1.02 [0.75, 1.4] | 0.89 | 0.92 [0.64, 1.31] | 0.64 | - |
| All Sites | PFBS | 1.09 [0.95, 1.24] | 0.23 | 0.97 [0.85, 1.1] | 0.64 | - |
| Pancreas | PFHxS | 1.07 [0.93, 1.23] | 0.36 | 0.97 [0.84, 1.11] | 0.64 | - |
| Pancreas | PFNA | 1.25 [0.96, 1.63] | 0.09 | 1.07 [0.81, 1.4] | 0.64 | - |
| Floor of Mouth | PFHxS | 1.19 [0.72, 1.98] | 0.50 | 0.84 [0.4, 1.76] | 0.64 | - |
| Respiratory System | PFHpA | 1.02 [0.92, 1.14] | 0.72 | 0.97 [0.87, 1.09] | 0.64 | - |
| Other Lymphocytic Leukemia | PFBS | 0.57 [0.09, 3.67] | 0.55 | 1.67 [0.19, 14.52] | 0.64 | - |
| Corpus Uteri | PFHxS | NA | NA | 1.03 [0.91, 1.16] | 0.64 | NA |
| Colon and Rectum | PFHpA | 1.03 [0.94, 1.13] | 0.54 | 0.98 [0.88, 1.08] | 0.65 | - |
| Nose Nasal Cavity and Middle Ear | PFHxS | 0.77 [0.46, 1.28] | 0.31 | 0.87 [0.48, 1.58] | 0.65 | - |
| Other Urinary Organs | PFBS | 1 [0.23, 4.36] | 1.00 | 0.52 [0.03, 8.89] | 0.65 | - |
| Other Acute Leukemia | PFHxS | 0.83 [0.35, 1.95] | 0.67 | 0.82 [0.34, 1.97] | 0.65 | - |
| Other Non Epithelial Skin | PFOS | 0.89 [0.66, 1.19] | 0.43 | 1.08 [0.76, 1.55] | 0.65 | - |
| Acute Myeloid Leukemia | PFNA | 1.07 [0.69, 1.64] | 0.76 | 0.88 [0.5, 1.55] | 0.66 | - |
| Retroperitoneum | PFHpA | 1.45 [0.88, 2.39] | 0.14 | 0.84 [0.4, 1.78] | 0.66 | - |
| Chronic Lymphocytic Leukemia | PFOA | 0.98 [0.8, 1.2] | 0.82 | 1.05 [0.86, 1.28] | 0.66 | - |
| Lymphoma | PFHpA | 1.05 [0.95, 1.17] | 0.34 | 1.03 [0.89, 1.19] | 0.67 | - |
| Brain | PFOS | 1.11 [0.92, 1.35] | 0.28 | 0.95 [0.77, 1.18] | 0.67 | - |
| Other Digestive Organs | PFBS | 0.7 [0.16, 2.96] | 0.63 | 1.32 [0.37, 4.78] | 0.67 | - |
| Descending Colon | PFHxS | 0.96 [0.68, 1.36] | 0.82 | 1.09 [0.74, 1.6] | 0.67 | - |
| Colon and Rectum | PFHxS | 0.98 [0.88, 1.09] | 0.70 | 0.98 [0.87, 1.09] | 0.67 | - |
| Floor of Mouth | PFBS | 1.21 [0.32, 4.64] | 0.78 | 0.6 [0.06, 6.48] | 0.67 | - |
| Other Biliary | PFOA | 0.97 [0.74, 1.28] | 0.84 | 1.06 [0.8, 1.42] | 0.67 | - |
| Hepatic Flexure | PFHxS | 0.92 [0.62, 1.38] | 0.70 | 1.09 [0.73, 1.61] | 0.68 | - |
| Bones and Joints | PFBS | 0.68 [0.18, 2.65] | 0.58 | 0.77 [0.22, 2.7] | 0.68 | - |
| Acute Lymphocytic Leukemia | PFHxS | 1.04 [0.72, 1.5] | 0.84 | 0.91 [0.59, 1.42] | 0.68 | - |
| Esophagus | PFNA | 0.84 [0.58, 1.22] | 0.36 | 1.14 [0.61, 2.12] | 0.68 | - |
| Brain and Other Nervous System | PFOS | 1.09 [0.9, 1.32] | 0.36 | 0.96 [0.78, 1.18] | 0.68 | - |
| Lymphocytic Leukemia | PFHpA | 0.86 [0.7, 1.05] | 0.13 | 1.04 [0.85, 1.29] | 0.69 | - |
| Vulva | PFBS | NA | NA | 1.16 [0.57, 2.35] | 0.69 | NA |
| Cervix Uteri | PFOS | NA | NA | 1.05 [0.84, 1.3] | 0.69 | NA |
| Colon excluding Rectum | PFOA | 0.98 [0.89, 1.09] | 0.73 | 0.98 [0.88, 1.08] | 0.69 | - |
| Lung and Bronchus | PFHpA | 1.02 [0.92, 1.14] | 0.69 | 0.98 [0.87, 1.09] | 0.69 | - |
| Cranial Nerves Other Nervous System | PFBS | 0.24 [0.01, 7.73] | 0.42 | 0.53 [0.02, 12.54] | 0.69 | - |
| Cervix Uteri | PFHpA | NA | NA | 1.05 [0.84, 1.3] | 0.69 | NA |
| Lung and Bronchus | PFBS | 1.13 [0.83, 1.55] | 0.43 | 1.07 [0.77, 1.48] | 0.69 | - |
| Gallbladder | PFHxS | 1.06 [0.66, 1.7] | 0.82 | 1.08 [0.72, 1.62] | 0.70 | - |
| Endocrine System | PFOS | 1.15 [0.95, 1.4] | 0.16 | 1.03 [0.88, 1.2] | 0.70 | - |
| Vagina | PFOS | NA | NA | 1.11 [0.66, 1.87] | 0.70 | NA |
| Other Myeloid Monocytic Leukemia | PFOS | 1.04 [0.4, 2.7] | 0.94 | 0.79 [0.24, 2.65] | 0.70 | - |
| Female Genital System | PFOA | NA | NA | 1.02 [0.94, 1.1] | 0.70 | NA |
| Small Intestine | PFNA | 1.04 [0.6, 1.8] | 0.89 | 1.12 [0.62, 2.05] | 0.70 | - |
| Lip | PFBS | 0.81 [0.22, 3.05] | 0.76 | 0.57 [0.03, 10.19] | 0.70 | - |
| Chronic Myeloid Leukemia | PFHpA | 0.97 [0.72, 1.31] | 0.86 | 0.94 [0.66, 1.33] | 0.71 | - |
| Other Acute Leukemia | PFOA | 0.91 [0.47, 1.75] | 0.78 | 0.87 [0.42, 1.82] | 0.71 | - |
| Stomach | PFNA | 0.98 [0.63, 1.53] | 0.93 | 1.1 [0.67, 1.8] | 0.71 | - |
| Peritoneum Omentum and Mesentery | PFBS | 0.18 [0, 172.84] | 0.63 | 0.73 [0.13, 3.93] | 0.71 | - |
| Myeloid and Monocytic Leukemia | PFOA | 1.09 [0.93, 1.27] | 0.28 | 1.04 [0.86, 1.25] | 0.71 | - |
| Urinary Bladder | PFNA | 1.04 [0.8, 1.35] | 0.78 | 0.93 [0.65, 1.34] | 0.71 | - |
| Bones and Joints | PFOA | 1.09 [0.77, 1.55] | 0.64 | 0.93 [0.63, 1.37] | 0.72 | - |
| Other Endocrine including Thymus | PFBS | 0.44 [0.06, 3.17] | 0.41 | 0.76 [0.17, 3.47] | 0.72 | - |
| Ovary | PFNA | NA | NA | 1.06 [0.75, 1.5] | 0.72 | NA |
| Cervix Uteri | PFHxS | NA | NA | 0.96 [0.75, 1.22] | 0.73 | NA |
| Sigmoid Colon | PFOS | 0.97 [0.8, 1.18] | 0.80 | 0.96 [0.79, 1.18] | 0.73 | - |
| Gallbladder | PFOS | 1.27 [0.84, 1.92] | 0.25 | 1.07 [0.74, 1.53] | 0.73 | - |
| All Sites | PFNA | 1.02 [0.92, 1.14] | 0.64 | 1.02 [0.92, 1.12] | 0.73 | - |
| NHL Extranodal | PFOS | 1.06 [0.87, 1.28] | 0.58 | 1.04 [0.85, 1.27] | 0.73 | - |
| Hodgkin Extranodal | PFHxS | 0.28 [0.02, 4.84] | 0.38 | 0.61 [0.03, 10.51] | 0.73 | - |
| Thyroid | PFHxS | 1.01 [0.8, 1.28] | 0.93 | 0.97 [0.81, 1.16] | 0.73 | - |
| Other Lymphocytic Leukemia | PFNA | 0.98 [0.32, 2.99] | 0.97 | 0.55 [0.02, 17.47] | 0.73 | - |
| Thyroid | PFNA | 0.83 [0.51, 1.34] | 0.44 | 1.06 [0.75, 1.49] | 0.73 | - |
| Transverse Colon | PFHxS | 0.84 [0.62, 1.14] | 0.27 | 1.05 [0.78, 1.43] | 0.74 | - |
| Endocrine System | PFNA | 0.88 [0.56, 1.37] | 0.57 | 1.06 [0.76, 1.48] | 0.74 | - |
| Cecum | PFOS | 1.12 [0.92, 1.37] | 0.26 | 0.97 [0.8, 1.17] | 0.74 | - |
| Brain and Other Nervous System | PFHxS | 1.09 [0.88, 1.34] | 0.44 | 0.96 [0.77, 1.21] | 0.74 | - |
| NHL Extranodal | PFHpA | 1.15 [0.95, 1.39] | 0.14 | 1.03 [0.85, 1.27] | 0.74 | - |
| Brain | PFHxS | 1.11 [0.9, 1.38] | 0.32 | 0.96 [0.76, 1.21] | 0.74 | - |
| Vagina | PFOA | NA | NA | 1.08 [0.68, 1.72] | 0.75 | NA |
| Non Hodgkin Lymphoma | PFHxS | 0.98 [0.86, 1.11] | 0.71 | 0.97 [0.82, 1.15] | 0.75 | - |
| Lymphocytic Leukemia | PFOA | 0.97 [0.81, 1.15] | 0.71 | 1.03 [0.85, 1.24] | 0.76 | - |
| Tongue | PFHxS | 0.86 [0.7, 1.06] | 0.17 | 1.05 [0.76, 1.44] | 0.76 | - |
| Brain and Other Nervous System | PFNA | 0.92 [0.6, 1.41] | 0.71 | 1.07 [0.69, 1.66] | 0.77 | - |
| Uterus NOS | PFHpA | NA | NA | 0.93 [0.59, 1.47] | 0.77 | NA |
| Small Intestine | PFHxS | 0.95 [0.72, 1.26] | 0.73 | 0.95 [0.7, 1.3] | 0.77 | - |
| Lip | PFHpA | 1.3 [0.84, 2.01] | 0.25 | 0.9 [0.43, 1.88] | 0.77 | - |
| Tongue | PFOS | 0.94 [0.78, 1.13] | 0.50 | 1.04 [0.78, 1.39] | 0.77 | - |
| Ureter | PFOS | 0.79 [0.49, 1.28] | 0.34 | 1.09 [0.62, 1.91] | 0.78 | - |
| Colon excluding Rectum | PFOS | 1.01 [0.9, 1.13] | 0.92 | 1.02 [0.91, 1.14] | 0.78 | - |
| Nose Nasal Cavity and Middle Ear | PFNA | 0.92 [0.35, 2.41] | 0.87 | 0.85 [0.27, 2.65] | 0.78 | - |
| NHL Extranodal | PFBS | 0.87 [0.48, 1.56] | 0.63 | 0.92 [0.5, 1.69] | 0.78 | - |
| Intrahepatic Bile Duct | PFOA | 1.11 [0.84, 1.47] | 0.47 | 1.04 [0.78, 1.4] | 0.78 | - |
| Digestive System | PFBS | 1.09 [0.89, 1.33] | 0.40 | 0.97 [0.78, 1.2] | 0.78 | - |
| Oral Cavity and Pharynx | PFBS | 1.11 [0.79, 1.55] | 0.56 | 1.07 [0.65, 1.78] | 0.78 | - |
| Lymphoma | PFNA | 1.07 [0.85, 1.35] | 0.56 | 0.96 [0.69, 1.31] | 0.78 | - |
| Vulva | PFOS | NA | NA | 1.04 [0.81, 1.33] | 0.78 | NA |
| Intrahepatic Bile Duct | PFBS | 0.96 [0.38, 2.41] | 0.92 | 0.87 [0.33, 2.32] | 0.78 | - |
| Salivary Gland | PFBS | 1.09 [0.45, 2.67] | 0.85 | 1.18 [0.35, 4] | 0.79 | - |
| Female Genital System | PFOS | NA | NA | 1.01 [0.93, 1.1] | 0.79 | NA |
| Anus Anal Canal and Anorectum | PFOA | 0.91 [0.65, 1.27] | 0.59 | 0.97 [0.77, 1.22] | 0.79 | - |
| Ascending Colon | PFHpA | 1.03 [0.85, 1.26] | 0.73 | 1.03 [0.85, 1.25] | 0.79 | - |
| Esophagus | PFOA | 0.96 [0.83, 1.12] | 0.63 | 0.96 [0.74, 1.26] | 0.79 | - |
| Vulva | PFHpA | NA | NA | 0.97 [0.75, 1.25] | 0.80 | NA |
| Lip | PFHxS | 1.51 [0.95, 2.4] | 0.08 | 0.9 [0.39, 2.05] | 0.80 | - |
| Splenic Flexure | PFOS | 0.83 [0.55, 1.26] | 0.39 | 0.94 [0.58, 1.52] | 0.80 | - |
| Urinary Bladder | PFHxS | 1.1 [0.96, 1.26] | 0.15 | 0.98 [0.81, 1.17] | 0.80 | - |
| Lymphoma | PFBS | 0.97 [0.71, 1.33] | 0.86 | 1.05 [0.69, 1.6] | 0.80 | - |
| Colon excluding Rectum | PFBS | 1.16 [0.84, 1.61] | 0.36 | 0.96 [0.69, 1.34] | 0.81 | - |
| Thyroid | PFHpA | 1.08 [0.87, 1.34] | 0.48 | 1.02 [0.87, 1.19] | 0.81 | - |
| Liver | PFBS | 1.22 [0.76, 1.96] | 0.41 | 1.09 [0.54, 2.23] | 0.81 | - |
| Non Hodgkin Lymphoma | PFHpA | 1.06 [0.95, 1.18] | 0.33 | 1.02 [0.87, 1.19] | 0.81 | - |
| Small Intestine | PFBS | 1.14 [0.56, 2.32] | 0.72 | 1.1 [0.5, 2.42] | 0.81 | - |
| Acute Lymphocytic Leukemia | PFOA | 0.86 [0.63, 1.17] | 0.33 | 0.96 [0.68, 1.36] | 0.81 | - |
| NHL Nodal | PFNA | 1.22 [0.92, 1.6] | 0.17 | 0.95 [0.63, 1.44] | 0.82 | - |
| Gallbladder | PFHpA | 1.06 [0.69, 1.64] | 0.78 | 0.96 [0.66, 1.4] | 0.82 | - |
| Peritoneum Omentum and Mesentery | PFOA | 0.63 [0.2, 1.97] | 0.43 | 0.96 [0.65, 1.41] | 0.82 | - |
| Tonsil | PFOS | 0.93 [0.73, 1.17] | 0.53 | 0.95 [0.59, 1.53] | 0.84 | - |
| Anus Anal Canal and Anorectum | PFHxS | 1.11 [0.74, 1.65] | 0.61 | 1.03 [0.77, 1.37] | 0.84 | - |
| Lip | PFOS | 1.28 [0.83, 1.96] | 0.26 | 0.93 [0.44, 1.94] | 0.84 | - |
| Non Hodgkin Lymphoma | PFBS | 0.93 [0.67, 1.29] | 0.66 | 0.96 [0.61, 1.5] | 0.85 | - |
| Rectum | PFOS | 1.01 [0.87, 1.17] | 0.91 | 1.02 [0.86, 1.21] | 0.85 | - |
| Respiratory System | PFOA | 1.05 [0.95, 1.15] | 0.33 | 0.99 [0.9, 1.1] | 0.85 | - |
| Chronic Lymphocytic Leukemia | PFOS | 0.95 [0.76, 1.2] | 0.67 | 0.98 [0.78, 1.23] | 0.85 | - |
| Other Non Epithelial Skin | PFBS | 1.27 [0.6, 2.67] | 0.53 | 0.9 [0.28, 2.86] | 0.86 | - |
| Acute Lymphocytic Leukemia | PFHpA | 0.89 [0.63, 1.26] | 0.52 | 0.96 [0.65, 1.44] | 0.86 | - |
| Skin excluding Basal and Squamous | PFBS | 1.37 [0.93, 2.01] | 0.11 | 0.96 [0.59, 1.54] | 0.86 | - |
| Lip | PFOA | 1.26 [0.86, 1.85] | 0.24 | 0.94 [0.49, 1.8] | 0.86 | - |
| Colon and Rectum | PFOS | 1.01 [0.92, 1.1] | 0.91 | 1.01 [0.91, 1.11] | 0.86 | - |
| Melanoma of the Skin | PFBS | 1.38 [0.92, 2.07] | 0.12 | 0.96 [0.58, 1.58] | 0.87 | - |
| Other Female Genital Organs | PFOS | NA | NA | 0.97 [0.71, 1.33] | 0.87 | NA |
| Oropharynx | PFBS | 0.27 [0.05, 1.54] | 0.14 | 0.81 [0.06, 10.13] | 0.87 | - |
| Splenic Flexure | PFNA | 0.75 [0.29, 1.92] | 0.55 | 0.91 [0.31, 2.72] | 0.87 | - |
| Gum and Other Mouth | PFHxS | 0.75 [0.52, 1.06] | 0.10 | 0.97 [0.67, 1.41] | 0.87 | - |
| Colon and Rectum | PFNA | 0.93 [0.76, 1.15] | 0.50 | 0.98 [0.79, 1.22] | 0.87 | - |
| Non Hodgkin Lymphoma | PFOS | 1.02 [0.91, 1.14] | 0.70 | 1.01 [0.87, 1.18] | 0.87 | - |
| Nasopharynx | PFOS | 0.7 [0.39, 1.26] | 0.24 | 0.94 [0.45, 1.97] | 0.87 | - |
| Ovary | PFBS | NA | NA | 1.04 [0.66, 1.63] | 0.88 | NA |
| Larynx | PFNA | 0.78 [0.47, 1.28] | 0.32 | 1.06 [0.49, 2.28] | 0.88 | - |
| Uterus NOS | PFHxS | NA | NA | 1.04 [0.64, 1.68] | 0.88 | NA |
| Rectum and Rectosigmoid Junction | PFOS | 1.01 [0.88, 1.15] | 0.93 | 0.99 [0.84, 1.16] | 0.88 | - |
| Ascending Colon | PFOA | 1.02 [0.86, 1.21] | 0.82 | 1.01 [0.85, 1.2] | 0.89 | - |
| Descending Colon | PFNA | 1.22 [0.64, 2.33] | 0.55 | 1.06 [0.5, 2.23] | 0.89 | - |
| Liver | PFNA | 1.24 [0.86, 1.78] | 0.25 | 0.96 [0.53, 1.72] | 0.89 | - |
| Corpus and Uterus NOS | PFOS | NA | NA | 1.01 [0.9, 1.12] | 0.89 | NA |
| Tonsil | PFNA | 0.8 [0.47, 1.35] | 0.40 | 0.93 [0.33, 2.63] | 0.89 | - |
| Hodgkin Extranodal | PFOS | 0.29 [0.02, 3.58] | 0.33 | 1.16 [0.14, 9.91] | 0.89 | - |
| Bones and Joints | PFOS | 0.9 [0.59, 1.36] | 0.60 | 1.03 [0.67, 1.58] | 0.90 | - |
| Corpus Uteri | PFOS | NA | NA | 1.01 [0.9, 1.12] | 0.90 | NA |
| Colon excluding Rectum | PFHpA | 1.02 [0.91, 1.14] | 0.74 | 1.01 [0.9, 1.13] | 0.91 | - |
| Endocrine System | PFHxS | 0.97 [0.77, 1.21] | 0.76 | 1.01 [0.85, 1.2] | 0.91 | - |
| Other Endocrine including Thymus | PFNA | 1.35 [0.54, 3.38] | 0.53 | 0.94 [0.31, 2.9] | 0.91 | - |
| Liver and Intrahepatic Bile Duct | PFBS | 1.19 [0.76, 1.85] | 0.45 | 1.03 [0.56, 1.9] | 0.92 | - |
| Ovary | PFOS | NA | NA | 0.99 [0.85, 1.16] | 0.92 | NA |
| NHL Nodal | PFHpA | 1.01 [0.89, 1.16] | 0.83 | 1.01 [0.84, 1.22] | 0.92 | - |
| Appendix | PFNA | 0.61 [0.26, 1.46] | 0.27 | 1.03 [0.5, 2.16] | 0.93 | - |
| Chronic Lymphocytic Leukemia | PFNA | 1.16 [0.71, 1.87] | 0.56 | 1.02 [0.63, 1.67] | 0.93 | - |
| Colon excluding Rectum | PFHxS | 0.97 [0.86, 1.11] | 0.69 | 0.99 [0.88, 1.13] | 0.93 | - |
| Other Lymphocytic Leukemia | PFOA | 1.16 [0.75, 1.8] | 0.51 | 0.96 [0.36, 2.54] | 0.93 | - |
| NHL Nodal | PFBS | 0.96 [0.66, 1.4] | 0.82 | 0.98 [0.57, 1.69] | 0.94 | - |
| Other Lymphocytic Leukemia | PFHpA | 1.13 [0.68, 1.87] | 0.63 | 1.04 [0.35, 3.14] | 0.94 | - |
| Retroperitoneum | PFHxS | 0.96 [0.52, 1.78] | 0.91 | 1.03 [0.47, 2.25] | 0.94 | - |
| Female Genital System | PFBS | NA | NA | 1.01 [0.79, 1.3] | 0.94 | NA |
| Cranial Nerves Other Nervous System | PFHxS | 0.64 [0.3, 1.34] | 0.23 | 0.98 [0.51, 1.89] | 0.94 | - |
| Nasopharynx | PFHxS | 0.55 [0.27, 1.12] | 0.10 | 0.97 [0.43, 2.19] | 0.95 | - |
| Myeloma | PFNA | 1 [0.69, 1.44] | 1.00 | 0.99 [0.64, 1.52] | 0.95 | - |
| Other Female Genital Organs | PFHpA | NA | NA | 0.99 [0.73, 1.35] | 0.95 | NA |
| Digestive System | PFNA | 1.02 [0.88, 1.19] | 0.76 | 1 [0.86, 1.18] | 0.95 | - |
| Transverse Colon | PFOS | 0.98 [0.76, 1.28] | 0.90 | 1.01 [0.76, 1.33] | 0.95 | - |
| NHL Nodal | PFHxS | 1.04 [0.9, 1.2] | 0.61 | 0.99 [0.81, 1.23] | 0.96 | - |
| Sigmoid Colon | PFNA | 1.01 [0.66, 1.54] | 0.96 | 0.99 [0.64, 1.53] | 0.96 | - |
| Other Urinary Organs | PFOA | 0.96 [0.62, 1.49] | 0.87 | 0.98 [0.53, 1.83] | 0.96 | - |
| Other Lymphocytic Leukemia | PFHxS | 1.35 [0.79, 2.29] | 0.27 | 1.03 [0.3, 3.58] | 0.96 | - |
| Lymphocytic Leukemia | PFOS | 0.94 [0.77, 1.14] | 0.51 | 0.99 [0.8, 1.23] | 0.96 | - |
| Hodgkin Extranodal | PFOA | 0.29 [0.03, 2.87] | 0.29 | 1.05 [0.14, 7.66] | 0.96 | - |
| Other Female Genital Organs | PFNA | NA | NA | 0.98 [0.51, 1.9] | 0.96 | NA |
| Thyroid | PFOS | 1.16 [0.94, 1.44] | 0.17 | 1 [0.86, 1.18] | 0.96 | - |
| Tonsil | PFHxS | 0.98 [0.76, 1.27] | 0.87 | 1.01 [0.6, 1.7] | 0.97 | - |
| Larynx | PFOS | 1.11 [0.9, 1.37] | 0.32 | 0.99 [0.69, 1.43] | 0.97 | - |
| Uterus NOS | PFOS | NA | NA | 1.01 [0.65, 1.56] | 0.97 | NA |
| Lymphoma | PFHxS | 0.99 [0.88, 1.11] | 0.84 | 1 [0.85, 1.17] | 0.97 | - |
| Breast | PFBS | 1.91 [0.67, 5.44] | 0.23 | 1 [0.84, 1.2] | 0.97 | - |
| Myeloid and Monocytic Leukemia | PFNA | 0.98 [0.67, 1.44] | 0.92 | 1.01 [0.63, 1.6] | 0.98 | - |
| Retroperitoneum | PFOS | 1.09 [0.63, 1.85] | 0.77 | 1.01 [0.5, 2.05] | 0.98 | - |
| Cranial Nerves Other Nervous System | PFOS | 0.73 [0.39, 1.39] | 0.34 | 1.01 [0.55, 1.85] | 0.98 | - |
| Acute Lymphocytic Leukemia | PFOS | 0.78 [0.55, 1.12] | 0.18 | 1 [0.68, 1.49] | 0.98 | - |
| Gum and Other Mouth | PFOS | 0.84 [0.61, 1.14] | 0.26 | 1 [0.72, 1.4] | 0.98 | - |
| Small Intestine | PFOS | 0.96 [0.74, 1.24] | 0.76 | 1 [0.75, 1.32] | 0.99 | - |
| Retroperitoneum | PFOA | 0.93 [0.56, 1.53] | 0.78 | 1 [0.53, 1.89] | 0.99 | - |
| Other Oral Cavity and Pharynx | PFBS | NA | NA | 0.99 [0.05, 19.84] | 0.99 | NA |
| Urinary Bladder | PFHpA | 1.03 [0.91, 1.16] | 0.65 | 1 [0.85, 1.18] | 0.99 | - |
| Other Lymphocytic Leukemia | PFOS | 1.29 [0.79, 2.09] | 0.31 | 1 [0.32, 3.07] | 1.00 | - |
| NHL Nodal | PFOS | 1.01 [0.88, 1.15] | 0.91 | 1 [0.83, 1.21] | 1.00 | - |
| Tongue | PFBS | 1.4 [0.86, 2.29] | 0.18 | 1 [0.43, 2.32] | 1.00 | - |
| Prostate | PFNA | 1.14 [0.98, 1.32] | 0.10 | NA | NA | NA |
| Male Genital System | PFNA | 1.12 [0.97, 1.3] | 0.12 | NA | NA | NA |
| Other Male Genital Organs | PFHpA | 0.52 [0.19, 1.41] | 0.20 | NA | NA | NA |
| Other Male Genital Organs | PFOA | 0.6 [0.26, 1.39] | 0.23 | NA | NA | NA |
| Other Male Genital Organs | PFOS | 0.57 [0.21, 1.53] | 0.26 | NA | NA | NA |
| Penis | PFNA | 0.53 [0.17, 1.67] | 0.28 | NA | NA | NA |
| Other Male Genital Organs | PFHxS | 0.57 [0.19, 1.69] | 0.31 | NA | NA | NA |
| Prostate | PFBS | 1.1 [0.9, 1.34] | 0.36 | NA | NA | NA |
| Prostate | PFOS | 1.03 [0.96, 1.1] | 0.47 | NA | NA | NA |
| Other Oral Cavity and Pharynx | PFNA | 0.56 [0.11, 2.78] | 0.48 | NA | NA | NA |
| Penis | PFOA | 0.89 [0.6, 1.3] | 0.54 | NA | NA | NA |
| Testis | PFOS | 0.93 [0.72, 1.19] | 0.54 | NA | NA | NA |
| Prostate | PFOA | 1.02 [0.96, 1.08] | 0.56 | NA | NA | NA |
| Male Genital System | PFOS | 1.02 [0.95, 1.09] | 0.57 | NA | NA | NA |
| Testis | PFHxS | 1.08 [0.82, 1.42] | 0.57 | NA | NA | NA |
| Penis | PFHxS | 1.14 [0.72, 1.8] | 0.58 | NA | NA | NA |
| Penis | PFHpA | 1.11 [0.73, 1.69] | 0.62 | NA | NA | NA |
| Penis | PFBS | 0.72 [0.18, 2.81] | 0.63 | NA | NA | NA |
| Male Genital System | PFBS | 1.05 [0.86, 1.27] | 0.64 | NA | NA | NA |
| Hodgkin Extranodal | PFNA | 0.31 [0, 41.82] | 0.64 | NA | NA | NA |
| Other Male Genital Organs | PFBS | 0.37 [0, 27.35] | 0.65 | NA | NA | NA |
| Trachea Mediastinum and Other Respiratory Organs | PFNA | 0.64 [0.09, 4.4] | 0.65 | NA | NA | NA |
| Male Genital System | PFOA | 1.01 [0.95, 1.08] | 0.67 | NA | NA | NA |
| Testis | PFOA | 0.95 [0.77, 1.19] | 0.68 | NA | NA | NA |
| Other Male Genital Organs | PFNA | 1.35 [0.27, 6.63] | 0.71 | NA | NA | NA |
| Prostate | PFHxS | 0.99 [0.91, 1.07] | 0.74 | NA | NA | NA |
| Male Genital System | PFHxS | 0.99 [0.92, 1.07] | 0.85 | NA | NA | NA |
| Male Genital System | PFHpA | 0.99 [0.93, 1.06] | 0.87 | NA | NA | NA |
| Prostate | PFHpA | 0.99 [0.93, 1.07] | 0.88 | NA | NA | NA |
| Testis | PFHpA | 0.98 [0.77, 1.26] | 0.89 | NA | NA | NA |
| Penis | PFOS | 0.98 [0.64, 1.5] | 0.93 | NA | NA | NA |
| Testis | PFNA | 0.99 [0.58, 1.72] | 0.98 | NA | NA | NA |
| Hodgkin Extranodal | PFBS | NA | NA | NA | NA | NA |
| Other Myeloid Monocytic Leukemia | PFBS | NA | NA | NA | NA | NA |
| Pleura | PFBS | NA | NA | NA | NA | NA |
| 1. All models were adjusted for county-level SES variables, urbanicity, smoking rate, obesity, and air pollution. | | | | | | |
| 2. cells were highlighted if crude p values were less than 0.05. | | | | | | |
| 3. NAs were produced with limited sample size or when we assessed cancers in male reproductive system among females or vice versa. | | | | | | |
| 4. ++ indicates cancers were significant in both sexes, + indicates cancers were siginificant only in one sex, - indicates cancers were not significant in either sex, NA indicates analysis was done only in one sex or none. | | | | | | |

| **Supplemental Table 17.** Stratified analysis by sex for the associations between detection of PFAS in drinking water using UCMR5 data and cancer incidence between 2016 and 2021. | | | | | | |
| --- | --- | --- | --- | --- | --- | --- |
|  |  | **Male** | | **Female** | |  |
| **Cancers** | **Exposures** | **IRR [95% CI]** | **p value^1,2^** | **IRR [95% CI]** | **p value^1,2^** | **Indicator of Significance^3,4^** |
| Rectum | PFHpA | 1.12 [1, 1.24] | 0.04 | 1.17 [1.02, 1.34] | 0.03 | ++ |
| Hodgkin Nodal | PFBS | 0.81 [0.68, 0.96] | 0.02 | 1.21 [1.01, 1.45] | 0.04 | ++ |
| Hodgkin Extranodal | PFHpA | 3.9 [1.45, 10.48] | 0.01 | 0.68 [0.12, 3.71] | 0.65 | + |
| Cranial Nerves Other Nervous System | PFHpA | 1.77 [1.19, 2.64] | 0.00 | 1.43 [0.93, 2.19] | 0.10 | + |
| Large Intestine NOS | PFBA | 1.31 [1.12, 1.54] | 0.00 | 1.1 [0.94, 1.29] | 0.22 | + |
| Floor of Mouth | PFHXA | 1.29 [1, 1.66] | 0.05 | 1.16 [0.79, 1.69] | 0.45 | + |
| Soft Tissue including Heart | PFHXA | 1.28 [1.1, 1.48] | 0.00 | 1.04 [0.88, 1.22] | 0.64 | + |
| Soft Tissue including Heart | PFPEA | 1.24 [1.07, 1.43] | 0.00 | 1.05 [0.9, 1.23] | 0.50 | + |
| Hepatic Flexure | PFBA | 1.21 [1.02, 1.44] | 0.03 | 0.95 [0.79, 1.14] | 0.56 | + |
| Gum and Other Mouth | PFBA | 1.2 [1.03, 1.4] | 0.02 | 0.89 [0.75, 1.05] | 0.15 | + |
| Soft Tissue including Heart | PFBS | 1.19 [1.02, 1.37] | 0.02 | 1.17 [1, 1.37] | 0.05 | + |
| Chronic Lymphocytic Leukemia | PFHXA | 1.18 [1.05, 1.34] | 0.01 | 1 [0.88, 1.14] | 0.97 | + |
| Sigmoid Colon | PFBS | 1.18 [1.07, 1.3] | 0.00 | 0.95 [0.85, 1.06] | 0.38 | + |
| Chronic Lymphocytic Leukemia | PFPEA | 1.17 [1.04, 1.32] | 0.01 | 1.05 [0.93, 1.19] | 0.42 | + |
| Sigmoid Colon | PFPEA | 1.17 [1.06, 1.29] | 0.00 | 0.94 [0.84, 1.04] | 0.24 | + |
| Sigmoid Colon | PFHXA | 1.16 [1.05, 1.29] | 0.00 | 1.01 [0.9, 1.13] | 0.84 | + |
| Brain and Other Nervous System | PFHpA | 1.15 [1, 1.32] | 0.04 | 1.03 [0.88, 1.2] | 0.72 | + |
| Lymphocytic Leukemia | PFHXA | 1.14 [1.03, 1.27] | 0.02 | 0.98 [0.87, 1.1] | 0.75 | + |
| Cecum | PFBS | 1.13 [1.02, 1.27] | 0.03 | 1.07 [0.96, 1.18] | 0.21 | + |
| Chronic Lymphocytic Leukemia | PFBS | 1.13 [1, 1.28] | 0.05 | 0.91 [0.8, 1.03] | 0.15 | + |
| Lymphocytic Leukemia | PFPEA | 1.12 [1.01, 1.24] | 0.04 | 1.04 [0.92, 1.16] | 0.54 | + |
| Lymphocytic Leukemia | PFBS | 1.11 [1, 1.24] | 0.05 | 0.95 [0.84, 1.06] | 0.36 | + |
| Leukemia | PFPEA | 1.09 [1.01, 1.17] | 0.03 | 0.99 [0.91, 1.08] | 0.84 | + |
| Urinary Bladder | PFPEA | 1.08 [1.01, 1.15] | 0.02 | 1 [0.91, 1.09] | 0.97 | + |
| Colon excluding Rectum | PFBS | 1.07 [1.01, 1.14] | 0.02 | 1.01 [0.94, 1.07] | 0.84 | + |
| Urinary Bladder | PFHXA | 1.07 [1, 1.15] | 0.05 | 0.97 [0.88, 1.06] | 0.51 | + |
| Colon and Rectum | PFBS | 1.05 [1, 1.11] | 0.04 | 1 [0.94, 1.06] | 0.96 | + |
| Chronic Lymphocytic Leukemia | PFBA | 0.88 [0.79, 0.99] | 0.03 | 0.97 [0.87, 1.09] | 0.64 | + |
| Stomach | PFPEA | 0.86 [0.77, 0.96] | 0.01 | 1.13 [0.99, 1.28] | 0.06 | + |
| Other Non Epithelial Skin | PFPEA | 0.86 [0.73, 1] | 0.05 | 1.09 [0.89, 1.33] | 0.39 | + |
| Other Biliary | PFBA | 0.85 [0.73, 0.99] | 0.04 | 1.05 [0.89, 1.23] | 0.56 | + |
| Other Biliary | PFHXA | 0.84 [0.7, 1] | 0.05 | 0.96 [0.79, 1.15] | 0.65 | + |
| Hodgkin Lymphoma | PFBS | 0.82 [0.7, 0.98] | 0.03 | 1.2 [1, 1.43] | 0.05 | + |
| Other Biliary | PFPEA | 0.82 [0.69, 0.97] | 0.02 | 0.94 [0.79, 1.13] | 0.51 | + |
| Oropharynx | PFBS | 0.79 [0.65, 0.97] | 0.02 | 1.12 [0.75, 1.66] | 0.59 | + |
| Other Biliary | PFBS | 0.79 [0.66, 0.94] | 0.01 | 0.99 [0.83, 1.19] | 0.95 | + |
| Oropharynx | PFHXA | 0.76 [0.61, 0.93] | 0.01 | 0.93 [0.61, 1.4] | 0.71 | + |
| Splenic Flexure | PFBS | 0.73 [0.58, 0.92] | 0.01 | 0.8 [0.61, 1.05] | 0.10 | + |
| Other Oral Cavity and Pharynx | PFBA | 0.62 [0.46, 0.84] | 0.00 | 1.09 [0.66, 1.79] | 0.74 | + |
| Other Male Genital Organs | PFHpA | 0.43 [0.22, 0.87] | 0.02 | NA | NA | NA |
| Acute Monocytic Leukemia | PFHpA | 1.04 [0.56, 1.94] | 0.90 | 3.24 [1.79, 5.86] | 0.00 | + |
| Trachea Mediastinum and Other Respiratory Organs | PFHpA | 0.95 [0.55, 1.64] | 0.85 | 2.28 [1.18, 4.4] | 0.01 | + |
| Other Myeloid Monocytic Leukemia | PFBA | 0.82 [0.51, 1.32] | 0.42 | 1.84 [1.06, 3.19] | 0.03 | + |
| Other Myeloid Monocytic Leukemia | PFPEA | 0.83 [0.49, 1.39] | 0.48 | 1.81 [1.01, 3.24] | 0.05 | + |
| Hypopharynx | PFHpA | 0.95 [0.7, 1.29] | 0.73 | 1.67 [1.02, 2.71] | 0.04 | + |
| Hypopharynx | PFBS | 1 [0.8, 1.25] | 0.98 | 1.64 [1.11, 2.44] | 0.01 | + |
| Other Urinary Organs | PFBA | 1.01 [0.79, 1.29] | 0.95 | 1.64 [1.16, 2.32] | 0.01 | + |
| Oropharynx | PFBA | 1.01 [0.85, 1.2] | 0.90 | 1.63 [1.16, 2.3] | 0.01 | + |
| Other Digestive Organs | PFHpA | 1.22 [0.88, 1.67] | 0.23 | 1.63 [1.19, 2.24] | 0.00 | + |
| Retroperitoneum | PFHpA | 1.27 [0.86, 1.88] | 0.23 | 1.62 [1.01, 2.61] | 0.05 | + |
| Retroperitoneum | PFPEA | 1.05 [0.77, 1.44] | 0.75 | 1.59 [1.1, 2.28] | 0.01 | + |
| Other Endocrine including Thymus | PFHpA | 0.78 [0.55, 1.12] | 0.18 | 1.53 [1.1, 2.13] | 0.01 | + |
| Intrahepatic Bile Duct | PFHpA | 1.09 [0.88, 1.36] | 0.42 | 1.5 [1.18, 1.9] | 0.00 | + |
| Cranial Nerves Other Nervous System | PFHXA | 1.2 [0.86, 1.67] | 0.29 | 1.47 [1.04, 2.08] | 0.03 | + |
| Cranial Nerves Other Nervous System | PFPEA | 1.23 [0.89, 1.69] | 0.22 | 1.44 [1.02, 2.02] | 0.04 | + |
| Ureter | PFBA | 1.18 [0.94, 1.47] | 0.15 | 1.43 [1.07, 1.91] | 0.02 | + |
| Salivary Gland | PFHpA | 1.09 [0.86, 1.38] | 0.47 | 1.35 [1.01, 1.8] | 0.04 | + |
| Intrahepatic Bile Duct | PFPEA | 0.93 [0.79, 1.1] | 0.40 | 1.25 [1.03, 1.5] | 0.02 | + |
| Stomach | PFHXA | 0.92 [0.82, 1.03] | 0.15 | 1.15 [1.01, 1.31] | 0.03 | + |
| Endocrine System | PFHpA | 1.07 [0.93, 1.23] | 0.32 | 1.15 [1.03, 1.29] | 0.02 | + |
| Thyroid | PFHpA | 1.12 [0.96, 1.3] | 0.15 | 1.13 [1.01, 1.28] | 0.04 | + |
| Endocrine System | PFHXA | 1.02 [0.92, 1.14] | 0.68 | 1.11 [1.01, 1.21] | 0.02 | + |
| Thyroid | PFHXA | 1.04 [0.93, 1.17] | 0.50 | 1.1 [1.01, 1.21] | 0.03 | + |
| Endocrine System | PFPEA | 1.05 [0.95, 1.17] | 0.33 | 1.09 [1, 1.19] | 0.04 | + |
| Colon excluding Rectum | PFHpA | 1 [0.92, 1.08] | 1.00 | 0.91 [0.83, 0.99] | 0.03 | + |
| Transverse Colon | PFBA | 0.99 [0.86, 1.13] | 0.85 | 0.85 [0.75, 0.98] | 0.02 | + |
| Appendix | PFHpA | 1.03 [0.79, 1.34] | 0.85 | 0.77 [0.59, 1] | 0.05 | + |
| Cranial Nerves Other Nervous System | PFBA | 1.24 [0.93, 1.67] | 0.15 | 0.73 [0.52, 1.01] | 0.05 | - |
| Lip | PFPEA | 1.08 [0.85, 1.38] | 0.53 | 1.45 [0.99, 2.11] | 0.06 | - |
| Thyroid | PFPEA | 1.06 [0.94, 1.18] | 0.35 | 1.09 [1, 1.19] | 0.06 | - |
| Cecum | PFPEA | 1.05 [0.94, 1.17] | 0.42 | 1.1 [1, 1.22] | 0.06 | - |
| Lung and Bronchus | PFHpA | 1.05 [0.97, 1.13] | 0.25 | 0.92 [0.84, 1] | 0.06 | - |
| Bones and Joints | PFBS | 0.81 [0.64, 1.03] | 0.09 | 0.79 [0.61, 1.01] | 0.06 | - |
| Gallbladder | PFBS | 1.07 [0.84, 1.37] | 0.59 | 0.82 [0.67, 1.01] | 0.06 | - |
| Larynx | PFPEA | 1.06 [0.94, 1.19] | 0.34 | 1.2 [0.99, 1.46] | 0.06 | - |
| Gallbladder | PFPEA | 0.91 [0.71, 1.17] | 0.47 | 0.83 [0.67, 1.02] | 0.07 | - |
| Hodgkin Nodal | PFHpA | 0.88 [0.69, 1.11] | 0.27 | 1.25 [0.98, 1.59] | 0.07 | - |
| Lip | PFHXA | 1.1 [0.85, 1.41] | 0.47 | 1.43 [0.97, 2.12] | 0.07 | - |
| Nose Nasal Cavity and Middle Ear | PFHXA | 0.79 [0.62, 1.01] | 0.06 | 0.75 [0.55, 1.03] | 0.07 | - |
| Hodgkin Nodal | PFPEA | 0.99 [0.84, 1.17] | 0.88 | 1.18 [0.98, 1.41] | 0.08 | - |
| Pancreas | PFHpA | 1 [0.91, 1.1] | 0.92 | 0.92 [0.83, 1.01] | 0.08 | - |
| Hodgkin Lymphoma | PFHpA | 0.91 [0.73, 1.15] | 0.44 | 1.24 [0.97, 1.57] | 0.08 | - |
| Rectum and Rectosigmoid Junction | PFHpA | 1.09 [0.99, 1.2] | 0.09 | 1.12 [0.99, 1.27] | 0.08 | - |
| Floor of Mouth | PFBS | 1 [0.78, 1.28] | 0.97 | 1.37 [0.96, 1.96] | 0.09 | - |
| Retroperitoneum | PFHXA | 1.15 [0.83, 1.58] | 0.39 | 1.39 [0.95, 2.03] | 0.09 | - |
| Pancreas | PFBA | 1.01 [0.95, 1.08] | 0.76 | 0.95 [0.89, 1.01] | 0.09 | - |
| Rectum | PFBA | 1.04 [0.97, 1.12] | 0.29 | 1.08 [0.99, 1.19] | 0.09 | - |
| Ascending Colon | PFBS | 0.97 [0.87, 1.08] | 0.60 | 1.1 [0.98, 1.23] | 0.10 | - |
| Melanoma of the Skin | PFHpA | 1.06 [0.95, 1.18] | 0.27 | 0.9 [0.79, 1.02] | 0.10 | - |
| Respiratory System | PFHpA | 1.04 [0.97, 1.13] | 0.28 | 0.93 [0.85, 1.01] | 0.10 | - |
| Gallbladder | PFHXA | 0.79 [0.6, 1.02] | 0.07 | 0.84 [0.67, 1.03] | 0.10 | - |
| Urinary Bladder | PFBA | 0.98 [0.92, 1.03] | 0.41 | 0.93 [0.86, 1.01] | 0.10 | - |
| Hodgkin Lymphoma | PFPEA | 1 [0.85, 1.18] | 0.99 | 1.16 [0.97, 1.39] | 0.10 | - |
| Chronic Lymphocytic Leukemia | PFHpA | 1.03 [0.87, 1.21] | 0.75 | 0.87 [0.73, 1.03] | 0.10 | - |
| Retroperitoneum | PFBS | 1.04 [0.76, 1.43] | 0.79 | 1.36 [0.94, 1.96] | 0.11 | - |
| Other Female Genital Organs | PFBS | NA | NA | 0.86 [0.72, 1.03] | 0.11 | NA |
| Nose Nasal Cavity and Middle Ear | PFHpA | 0.92 [0.67, 1.26] | 0.59 | 0.71 [0.46, 1.08] | 0.11 | - |
| Hypopharynx | PFBA | 0.94 [0.77, 1.15] | 0.55 | 1.35 [0.94, 1.94] | 0.11 | - |
| Ovary | PFPEA | NA | NA | 0.93 [0.85, 1.02] | 0.12 | NA |
| Cranial Nerves Other Nervous System | PFBS | 0.95 [0.68, 1.32] | 0.75 | 1.31 [0.93, 1.84] | 0.12 | - |
| Other Myeloid Monocytic Leukemia | PFHXA | 0.71 [0.41, 1.24] | 0.23 | 1.59 [0.88, 2.88] | 0.12 | - |
| Melanoma of the Skin | PFBS | 0.98 [0.91, 1.06] | 0.65 | 0.93 [0.84, 1.02] | 0.12 | - |
| NHL Nodal | PFPEA | 1.01 [0.95, 1.09] | 0.70 | 0.92 [0.83, 1.02] | 0.12 | - |
| Hodgkin Extranodal | PFHXA | 1.54 [0.59, 4.01] | 0.38 | 0.31 [0.07, 1.39] | 0.13 | - |
| Splenic Flexure | PFPEA | 0.97 [0.78, 1.21] | 0.79 | 0.81 [0.62, 1.06] | 0.13 | - |
| Descending Colon | PFBA | 1.05 [0.91, 1.22] | 0.50 | 0.87 [0.73, 1.04] | 0.13 | - |
| Vagina | PFPEA | NA | NA | 0.81 [0.61, 1.07] | 0.13 | NA |
| Respiratory System | PFBS | 1.03 [0.97, 1.09] | 0.35 | 1.05 [0.99, 1.12] | 0.13 | - |
| Larynx | PFHpA | 1.03 [0.88, 1.2] | 0.75 | 1.22 [0.94, 1.58] | 0.13 | - |
| Endocrine System | PFBS | 1.07 [0.97, 1.19] | 0.18 | 1.07 [0.98, 1.16] | 0.13 | - |
| Lung and Bronchus | PFBS | 1.03 [0.97, 1.09] | 0.38 | 1.05 [0.98, 1.12] | 0.13 | - |
| Acute Monocytic Leukemia | PFHXA | 1.32 [0.83, 2.09] | 0.24 | 1.47 [0.89, 2.42] | 0.13 | - |
| Acute Monocytic Leukemia | PFBA | 0.75 [0.5, 1.14] | 0.18 | 1.41 [0.9, 2.22] | 0.14 | - |
| Skin excluding Basal and Squamous | PFHpA | 1.05 [0.94, 1.16] | 0.38 | 0.91 [0.8, 1.03] | 0.14 | - |
| Myeloma | PFHpA | 0.99 [0.87, 1.11] | 0.82 | 0.89 [0.77, 1.04] | 0.14 | - |
| Rectum and Rectosigmoid Junction | PFBA | 1.03 [0.96, 1.1] | 0.42 | 1.07 [0.98, 1.16] | 0.14 | - |
| Vagina | PFBS | NA | NA | 0.81 [0.61, 1.08] | 0.14 | NA |
| Vagina | PFBA | NA | NA | 0.83 [0.64, 1.07] | 0.15 | NA |
| Vulva | PFHXA | NA | NA | 1.11 [0.96, 1.29] | 0.15 | NA |
| Skin excluding Basal and Squamous | PFBS | 0.98 [0.91, 1.06] | 0.63 | 0.93 [0.85, 1.03] | 0.15 | - |
| Splenic Flexure | PFHXA | 0.92 [0.73, 1.15] | 0.46 | 0.82 [0.62, 1.08] | 0.15 | - |
| Non Hodgkin Lymphoma | PFPEA | 1 [0.94, 1.07] | 0.91 | 0.94 [0.86, 1.02] | 0.15 | - |
| Trachea Mediastinum and Other Respiratory Organs | PFHXA | 1.25 [0.82, 1.89] | 0.30 | 1.53 [0.85, 2.75] | 0.15 | - |
| Rectosigmoid Junction | PFHXA | 0.94 [0.81, 1.09] | 0.39 | 0.88 [0.73, 1.05] | 0.16 | - |
| Bones and Joints | PFHXA | 0.83 [0.65, 1.06] | 0.13 | 0.83 [0.64, 1.07] | 0.16 | - |
| Breast | PFBA | 1.16 [0.94, 1.42] | 0.17 | 0.98 [0.95, 1.01] | 0.16 | - |
| NHL Extranodal | PFHXA | 0.97 [0.87, 1.09] | 0.64 | 0.92 [0.81, 1.03] | 0.16 | - |
| Acute Monocytic Leukemia | PFPEA | 1.13 [0.72, 1.77] | 0.59 | 1.42 [0.87, 2.32] | 0.16 | - |
| Other Oral Cavity and Pharynx | PFBS | 0.75 [0.54, 1.04] | 0.09 | 1.45 [0.86, 2.45] | 0.17 | - |
| Larynx | PFHXA | 1.03 [0.92, 1.16] | 0.61 | 1.15 [0.94, 1.41] | 0.17 | - |
| Uterus NOS | PFHpA | NA | NA | 1.24 [0.91, 1.69] | 0.17 | NA |
| Non Hodgkin Lymphoma | PFBS | 1.01 [0.95, 1.07] | 0.82 | 0.94 [0.86, 1.03] | 0.17 | - |
| Thyroid | PFBS | 1.09 [0.97, 1.22] | 0.14 | 1.06 [0.97, 1.16] | 0.17 | - |
| Kidney and Renal Pelvis | PFPEA | 0.99 [0.93, 1.04] | 0.61 | 0.94 [0.87, 1.03] | 0.18 | - |
| Other Endocrine including Thymus | PFPEA | 1.04 [0.81, 1.33] | 0.78 | 1.19 [0.92, 1.54] | 0.18 | - |
| Acute Monocytic Leukemia | PFBS | 0.92 [0.58, 1.45] | 0.72 | 1.4 [0.86, 2.3] | 0.18 | - |
| Tonsil | PFBS | 1.04 [0.92, 1.18] | 0.55 | 0.83 [0.64, 1.09] | 0.18 | - |
| Melanoma of the Skin | PFHXA | 0.98 [0.9, 1.06] | 0.55 | 0.93 [0.85, 1.03] | 0.18 | - |
| Esophagus | PFHpA | 1.04 [0.92, 1.18] | 0.49 | 0.86 [0.69, 1.07] | 0.18 | - |
| Lymphocytic Leukemia | PFHpA | 1.02 [0.88, 1.18] | 0.79 | 0.9 [0.77, 1.05] | 0.18 | - |
| Gallbladder | PFHpA | 0.93 [0.67, 1.31] | 0.69 | 0.82 [0.62, 1.1] | 0.19 | - |
| Melanoma of the Skin | PFBA | 0.96 [0.89, 1.03] | 0.26 | 0.94 [0.87, 1.03] | 0.19 | - |
| Skin excluding Basal and Squamous | PFBA | 0.95 [0.89, 1.02] | 0.17 | 0.95 [0.87, 1.03] | 0.20 | - |
| Bones and Joints | PFPEA | 0.82 [0.65, 1.03] | 0.09 | 0.85 [0.66, 1.09] | 0.20 | - |
| Myeloma | PFBS | 1 [0.91, 1.1] | 0.98 | 0.93 [0.84, 1.04] | 0.22 | - |
| Aleukemic Subleukemic and NOS | PFHpA | 0.78 [0.52, 1.17] | 0.23 | 1.26 [0.86, 1.84] | 0.23 | - |
| Cecum | PFHpA | 1.14 [0.98, 1.32] | 0.09 | 0.92 [0.8, 1.06] | 0.23 | - |
| Brain and Other Nervous System | PFBA | 1.04 [0.95, 1.14] | 0.44 | 0.94 [0.85, 1.04] | 0.23 | - |
| Oral Cavity and Pharynx | PFHpA | 0.98 [0.9, 1.07] | 0.66 | 1.08 [0.95, 1.24] | 0.23 | - |
| Anus Anal Canal and Anorectum | PFHXA | 0.99 [0.81, 1.22] | 0.95 | 1.09 [0.94, 1.27] | 0.23 | - |
| Hypopharynx | PFPEA | 0.95 [0.76, 1.19] | 0.68 | 1.27 [0.85, 1.9] | 0.24 | - |
| Salivary Gland | PFBA | 1.06 [0.9, 1.24] | 0.48 | 0.88 [0.72, 1.09] | 0.24 | - |
| Other Non Epithelial Skin | PFHXA | 0.89 [0.76, 1.05] | 0.17 | 1.13 [0.92, 1.39] | 0.24 | - |
| Sigmoid Colon | PFHpA | 1.01 [0.88, 1.16] | 0.85 | 0.91 [0.79, 1.06] | 0.24 | - |
| Large Intestine NOS | PFHpA | 0.92 [0.72, 1.18] | 0.50 | 0.86 [0.67, 1.11] | 0.25 | - |
| Skin excluding Basal and Squamous | PFHXA | 0.97 [0.89, 1.05] | 0.42 | 0.95 [0.86, 1.04] | 0.25 | - |
| Tongue | PFHpA | 0.98 [0.85, 1.13] | 0.81 | 1.13 [0.92, 1.38] | 0.25 | - |
| Salivary Gland | PFBS | 1.09 [0.92, 1.3] | 0.33 | 1.14 [0.91, 1.43] | 0.25 | - |
| Vulva | PFHpA | NA | NA | 1.12 [0.92, 1.35] | 0.25 | NA |
| Appendix | PFBA | 0.95 [0.8, 1.14] | 0.59 | 0.91 [0.76, 1.07] | 0.26 | - |
| Ascending Colon | PFPEA | 0.95 [0.85, 1.06] | 0.36 | 1.06 [0.95, 1.19] | 0.26 | - |
| Urinary System | PFHXA | 1.03 [0.98, 1.08] | 0.24 | 0.97 [0.91, 1.03] | 0.27 | - |
| Esophagus | PFBA | 1.02 [0.94, 1.11] | 0.58 | 1.09 [0.94, 1.25] | 0.27 | - |
| Acute Myeloid Leukemia | PFHpA | 1.06 [0.92, 1.24] | 0.41 | 0.9 [0.74, 1.09] | 0.27 | - |
| Lip | PFBA | 0.85 [0.68, 1.06] | 0.16 | 1.22 [0.86, 1.72] | 0.27 | - |
| Bones and Joints | PFHpA | 1.09 [0.8, 1.5] | 0.58 | 0.83 [0.59, 1.16] | 0.27 | - |
| Aleukemic Subleukemic and NOS | PFBS | 0.79 [0.6, 1.05] | 0.10 | 0.85 [0.63, 1.14] | 0.27 | - |
| Other Acute Leukemia | PFBA | 0.98 [0.69, 1.39] | 0.91 | 1.24 [0.84, 1.82] | 0.27 | - |
| NHL Extranodal | PFBS | 1.02 [0.91, 1.14] | 0.75 | 0.94 [0.83, 1.05] | 0.27 | - |
| NHL Nodal | PFBS | 1 [0.94, 1.07] | 0.94 | 0.94 [0.85, 1.05] | 0.28 | - |
| Other Female Genital Organs | PFPEA | NA | NA | 1.1 [0.92, 1.32] | 0.28 | NA |
| Urinary System | PFPEA | 1.04 [0.99, 1.09] | 0.11 | 0.97 [0.91, 1.03] | 0.28 | - |
| Liver | PFHpA | 1.03 [0.9, 1.17] | 0.69 | 0.9 [0.73, 1.09] | 0.28 | - |
| Pancreas | PFHXA | 1 [0.93, 1.07] | 0.91 | 0.96 [0.89, 1.03] | 0.29 | - |
| Tongue | PFBS | 0.94 [0.84, 1.04] | 0.24 | 0.92 [0.78, 1.07] | 0.29 | - |
| Other Endocrine including Thymus | PFHXA | 0.9 [0.7, 1.17] | 0.44 | 1.15 [0.89, 1.5] | 0.29 | - |
| Nasopharynx | PFHXA | 0.83 [0.61, 1.13] | 0.24 | 0.79 [0.52, 1.22] | 0.29 | - |
| Hypopharynx | PFHXA | 0.98 [0.78, 1.24] | 0.87 | 1.25 [0.83, 1.88] | 0.29 | - |
| Hepatic Flexure | PFHpA | 1.17 [0.91, 1.51] | 0.22 | 0.86 [0.65, 1.14] | 0.29 | - |
| Other Leukemia | PFHpA | 0.88 [0.63, 1.23] | 0.45 | 1.19 [0.86, 1.66] | 0.30 | - |
| Sigmoid Colon | PFBA | 0.99 [0.9, 1.09] | 0.86 | 0.95 [0.86, 1.05] | 0.30 | - |
| Hepatic Flexure | PFBS | 1.14 [0.95, 1.38] | 0.17 | 1.11 [0.91, 1.36] | 0.30 | - |
| Soft Tissue including Heart | PFHpA | 0.98 [0.8, 1.19] | 0.83 | 0.89 [0.72, 1.11] | 0.30 | - |
| Other Endocrine including Thymus | PFBS | 0.98 [0.76, 1.26] | 0.86 | 1.15 [0.88, 1.49] | 0.30 | - |
| Other Biliary | PFHpA | 0.91 [0.72, 1.16] | 0.45 | 1.13 [0.89, 1.43] | 0.30 | - |
| Endocrine System | PFBA | 1.07 [0.98, 1.17] | 0.15 | 1.04 [0.96, 1.12] | 0.30 | - |
| Ureter | PFHpA | 1.08 [0.77, 1.5] | 0.66 | 1.24 [0.82, 1.88] | 0.31 | - |
| Floor of Mouth | PFPEA | 1.18 [0.92, 1.51] | 0.19 | 1.21 [0.84, 1.74] | 0.31 | - |
| Urinary System | PFBA | 1 [0.96, 1.05] | 0.85 | 0.97 [0.92, 1.03] | 0.31 | - |
| All Sites | PFHpA | 1.03 [1, 1.06] | 0.09 | 0.98 [0.95, 1.02] | 0.31 | - |
| Transverse Colon | PFHXA | 0.87 [0.75, 1.01] | 0.08 | 0.92 [0.79, 1.08] | 0.31 | - |
| Chronic Myeloid Leukemia | PFBA | 1.14 [0.98, 1.32] | 0.09 | 0.92 [0.77, 1.09] | 0.32 | - |
| Appendix | PFHXA | 1.05 [0.86, 1.29] | 0.63 | 0.91 [0.75, 1.1] | 0.32 | - |
| Thyroid | PFBA | 1.1 [1, 1.22] | 0.06 | 1.04 [0.96, 1.13] | 0.33 | - |
| Kidney and Renal Pelvis | PFHXA | 0.98 [0.92, 1.04] | 0.51 | 0.96 [0.88, 1.04] | 0.33 | - |
| Ureter | PFHXA | 1.09 [0.84, 1.4] | 0.53 | 1.18 [0.85, 1.64] | 0.33 | - |
| Other Female Genital Organs | PFBA | NA | NA | 0.92 [0.78, 1.09] | 0.33 | NA |
| Liver and Intrahepatic Bile Duct | PFBA | 1.03 [0.95, 1.12] | 0.43 | 0.95 [0.85, 1.06] | 0.33 | - |
| Nasopharynx | PFBS | 0.85 [0.63, 1.15] | 0.29 | 0.81 [0.54, 1.24] | 0.33 | - |
| Descending Colon | PFHpA | 1.03 [0.82, 1.29] | 0.78 | 0.88 [0.68, 1.14] | 0.34 | - |
| Bones and Joints | PFBA | 1.04 [0.85, 1.29] | 0.69 | 1.11 [0.89, 1.39] | 0.34 | - |
| Tonsil | PFHpA | 0.94 [0.79, 1.11] | 0.44 | 0.84 [0.58, 1.21] | 0.34 | - |
| Acute Myeloid Leukemia | PFPEA | 1.01 [0.9, 1.13] | 0.86 | 0.93 [0.81, 1.08] | 0.34 | - |
| Large Intestine NOS | PFHXA | 0.88 [0.73, 1.06] | 0.18 | 1.09 [0.91, 1.31] | 0.34 | - |
| Tongue | PFBA | 0.97 [0.89, 1.07] | 0.59 | 0.93 [0.81, 1.08] | 0.35 | - |
| Small Intestine | PFBA | 0.96 [0.85, 1.1] | 0.58 | 1.07 [0.93, 1.22] | 0.35 | - |
| Other Urinary Organs | PFBS | 0.97 [0.74, 1.27] | 0.82 | 1.21 [0.82, 1.78] | 0.35 | - |
| Salivary Gland | PFHXA | 1.1 [0.92, 1.32] | 0.29 | 1.12 [0.89, 1.41] | 0.35 | - |
| Kidney and Renal Pelvis | PFBS | 1.04 [0.98, 1.1] | 0.16 | 0.96 [0.89, 1.04] | 0.35 | - |
| Other Myeloid Monocytic Leukemia | PFBS | 0.88 [0.52, 1.48] | 0.63 | 1.32 [0.73, 2.37] | 0.36 | - |
| Female Genital System | PFBS | NA | NA | 0.98 [0.93, 1.03] | 0.36 | NA |
| Liver | PFBA | 1.03 [0.94, 1.12] | 0.55 | 0.94 [0.83, 1.07] | 0.36 | - |
| Lung and Bronchus | PFBA | 1.02 [0.97, 1.07] | 0.50 | 1.03 [0.97, 1.09] | 0.36 | - |
| Acute Lymphocytic Leukemia | PFHXA | 0.99 [0.81, 1.21] | 0.90 | 0.9 [0.72, 1.13] | 0.37 | - |
| Colon and Rectum | PFHpA | 1.03 [0.96, 1.1] | 0.41 | 0.97 [0.9, 1.04] | 0.37 | - |
| Hodgkin Extranodal | PFPEA | 2.09 [0.85, 5.14] | 0.11 | 0.58 [0.17, 1.94] | 0.37 | - |
| Hodgkin Extranodal | PFBS | 1.72 [0.71, 4.17] | 0.23 | 0.56 [0.16, 2.01] | 0.38 | - |
| Gum and Other Mouth | PFBS | 1.05 [0.88, 1.25] | 0.56 | 1.09 [0.9, 1.3] | 0.38 | - |
| Respiratory System | PFHXA | 1.01 [0.95, 1.07] | 0.71 | 1.03 [0.96, 1.1] | 0.38 | - |
| Liver and Intrahepatic Bile Duct | PFPEA | 0.94 [0.86, 1.03] | 0.19 | 1.06 [0.94, 1.19] | 0.38 | - |
| Pancreas | PFPEA | 0.96 [0.9, 1.03] | 0.29 | 0.97 [0.9, 1.04] | 0.38 | - |
| Cecum | PFHXA | 1.01 [0.9, 1.14] | 0.82 | 1.05 [0.94, 1.16] | 0.39 | - |
| Aleukemic Subleukemic and NOS | PFBA | 1.01 [0.8, 1.29] | 0.91 | 0.89 [0.69, 1.16] | 0.39 | - |
| Breast | PFPEA | 1.07 [0.85, 1.34] | 0.58 | 1.01 [0.98, 1.05] | 0.39 | - |
| Cervix Uteri | PFHpA | NA | NA | 1.07 [0.91, 1.26] | 0.39 | NA |
| Liver | PFBS | 1.05 [0.95, 1.15] | 0.34 | 0.94 [0.81, 1.08] | 0.39 | - |
| Respiratory System | PFBA | 1.01 [0.96, 1.07] | 0.65 | 1.03 [0.97, 1.09] | 0.39 | - |
| Other Non Epithelial Skin | PFHpA | 0.89 [0.72, 1.1] | 0.28 | 1.12 [0.86, 1.47] | 0.39 | - |
| Appendix | PFBS | 0.93 [0.76, 1.14] | 0.48 | 0.92 [0.76, 1.11] | 0.40 | - |
| Descending Colon | PFPEA | 1 [0.85, 1.18] | 0.99 | 0.92 [0.76, 1.12] | 0.40 | - |
| Non Hodgkin Lymphoma | PFHXA | 1 [0.94, 1.06] | 0.98 | 0.96 [0.88, 1.05] | 0.40 | - |
| Corpus Uteri | PFHpA | NA | NA | 0.96 [0.89, 1.05] | 0.41 | NA |
| Cervix Uteri | PFBA | NA | NA | 1.05 [0.94, 1.16] | 0.41 | NA |
| Retroperitoneum | PFBA | 0.97 [0.73, 1.29] | 0.83 | 1.15 [0.83, 1.6] | 0.41 | - |
| Colon excluding Rectum | PFBA | 1.05 [1, 1.11] | 0.06 | 0.98 [0.92, 1.03] | 0.41 | - |
| Lymphoma | PFPEA | 1 [0.95, 1.06] | 0.90 | 0.97 [0.89, 1.05] | 0.41 | - |
| Tonsil | PFPEA | 1.03 [0.91, 1.17] | 0.62 | 0.9 [0.69, 1.17] | 0.42 | - |
| Gum and Other Mouth | PFHXA | 1.02 [0.85, 1.22] | 0.82 | 1.08 [0.89, 1.3] | 0.43 | - |
| Lung and Bronchus | PFHXA | 1.01 [0.95, 1.07] | 0.70 | 1.03 [0.96, 1.1] | 0.43 | - |
| Acute Myeloid Leukemia | PFBS | 0.98 [0.88, 1.1] | 0.74 | 0.94 [0.82, 1.09] | 0.43 | - |
| Myeloma | PFPEA | 0.99 [0.9, 1.08] | 0.78 | 0.96 [0.86, 1.07] | 0.43 | - |
| Other Digestive Organs | PFPEA | 1.23 [0.97, 1.57] | 0.09 | 1.11 [0.86, 1.44] | 0.43 | - |
| Brain | PFHXA | 1.02 [0.92, 1.15] | 0.68 | 0.95 [0.85, 1.07] | 0.43 | - |
| Soft Tissue including Heart | PFBA | 0.91 [0.8, 1.04] | 0.19 | 1.06 [0.92, 1.22] | 0.43 | - |
| Liver and Intrahepatic Bile Duct | PFHpA | 1.04 [0.92, 1.17] | 0.55 | 1.07 [0.91, 1.26] | 0.43 | - |
| Transverse Colon | PFBS | 1 [0.87, 1.16] | 0.96 | 0.94 [0.81, 1.09] | 0.44 | - |
| Trachea Mediastinum and Other Respiratory Organs | PFPEA | 0.94 [0.63, 1.42] | 0.78 | 1.26 [0.7, 2.25] | 0.44 | - |
| Gallbladder | PFBA | 0.94 [0.75, 1.18] | 0.58 | 0.93 [0.77, 1.12] | 0.44 | - |
| Corpus Uteri | PFPEA | NA | NA | 1.03 [0.96, 1.09] | 0.44 | NA |
| Breast | PFHXA | 1.11 [0.88, 1.41] | 0.38 | 0.99 [0.95, 1.02] | 0.44 | - |
| Urinary System | PFBS | 1.05 [1, 1.1] | 0.07 | 0.98 [0.92, 1.04] | 0.45 | - |
| Other Oral Cavity and Pharynx | PFHpA | 0.67 [0.42, 1.07] | 0.10 | 0.75 [0.35, 1.6] | 0.45 | - |
| Leukemia | PFHpA | 1.03 [0.93, 1.15] | 0.53 | 0.96 [0.85, 1.07] | 0.45 | - |
| Lymphoma | PFHXA | 1 [0.94, 1.06] | 0.92 | 0.97 [0.89, 1.05] | 0.45 | - |
| Esophagus | PFHXA | 1.04 [0.94, 1.14] | 0.44 | 0.94 [0.8, 1.11] | 0.46 | - |
| Leukemia | PFBS | 1.07 [0.99, 1.16] | 0.08 | 0.97 [0.89, 1.05] | 0.46 | - |
| Corpus Uteri | PFBS | NA | NA | 0.98 [0.92, 1.04] | 0.46 | NA |
| Corpus and Uterus NOS | PFPEA | NA | NA | 1.02 [0.96, 1.09] | 0.47 | NA |
| Tongue | PFHXA | 0.95 [0.85, 1.06] | 0.33 | 0.94 [0.8, 1.11] | 0.47 | - |
| Ascending Colon | PFHXA | 0.99 [0.89, 1.11] | 0.87 | 1.04 [0.93, 1.17] | 0.47 | - |
| Urinary Bladder | PFHpA | 1 [0.91, 1.09] | 0.95 | 0.95 [0.84, 1.08] | 0.47 | - |
| Stomach | PFBA | 1.02 [0.92, 1.13] | 0.66 | 1.04 [0.93, 1.17] | 0.47 | - |
| Lymphocytic Leukemia | PFBA | 0.94 [0.85, 1.03] | 0.18 | 0.96 [0.87, 1.07] | 0.48 | - |
| Other Digestive Organs | PFBA | 0.98 [0.78, 1.22] | 0.84 | 1.09 [0.86, 1.38] | 0.48 | - |
| Gum and Other Mouth | PFPEA | 1.07 [0.9, 1.27] | 0.44 | 1.07 [0.89, 1.28] | 0.48 | - |
| Other Leukemia | PFPEA | 0.91 [0.72, 1.15] | 0.43 | 0.92 [0.71, 1.18] | 0.49 | - |
| Small Intestine | PFHpA | 0.98 [0.8, 1.18] | 0.80 | 1.07 [0.88, 1.31] | 0.50 | - |
| Ascending Colon | PFBA | 1.02 [0.92, 1.12] | 0.73 | 1.04 [0.94, 1.14] | 0.50 | - |
| Peritoneum Omentum and Mesentery | PFBS | 0.66 [0.35, 1.28] | 0.22 | 0.92 [0.72, 1.17] | 0.50 | - |
| Corpus and Uterus NOS | PFBS | NA | NA | 0.98 [0.92, 1.04] | 0.51 | NA |
| Other Urinary Organs | PFPEA | 1.25 [0.95, 1.63] | 0.11 | 1.14 [0.77, 1.69] | 0.51 | - |
| Other Non Epithelial Skin | PFBS | 0.98 [0.84, 1.14] | 0.80 | 1.07 [0.88, 1.31] | 0.51 | - |
| Kidney and Renal Pelvis | PFBA | 1.03 [0.98, 1.09] | 0.20 | 0.98 [0.91, 1.05] | 0.51 | - |
| Corpus and Uterus NOS | PFHpA | NA | NA | 0.97 [0.89, 1.06] | 0.51 | NA |
| Hodgkin Extranodal | PFBA | 1.39 [0.62, 3.13] | 0.42 | 0.7 [0.24, 2.02] | 0.51 | - |
| Melanoma of the Skin | PFPEA | 1.01 [0.93, 1.09] | 0.80 | 0.97 [0.88, 1.07] | 0.51 | - |
| Transverse Colon | PFHpA | 0.86 [0.7, 1.06] | 0.15 | 0.93 [0.76, 1.15] | 0.52 | - |
| Other Female Genital Organs | PFHpA | NA | NA | 0.92 [0.72, 1.18] | 0.52 | NA |
| Brain | PFBA | 1.03 [0.93, 1.13] | 0.60 | 0.97 [0.87, 1.07] | 0.52 | - |
| Lymphoma | PFBS | 0.99 [0.93, 1.05] | 0.63 | 0.97 [0.9, 1.06] | 0.52 | - |
| Acute Myeloid Leukemia | PFHXA | 1 [0.89, 1.12] | 0.98 | 0.95 [0.82, 1.1] | 0.52 | - |
| Other Urinary Organs | PFHpA | 0.94 [0.65, 1.35] | 0.72 | 0.83 [0.48, 1.46] | 0.52 | - |
| Rectosigmoid Junction | PFPEA | 0.9 [0.78, 1.04] | 0.15 | 0.94 [0.79, 1.13] | 0.52 | - |
| Liver and Intrahepatic Bile Duct | PFBS | 1.05 [0.96, 1.15] | 0.27 | 0.96 [0.85, 1.09] | 0.52 | - |
| Acute Lymphocytic Leukemia | PFBA | 1.14 [0.95, 1.36] | 0.15 | 0.94 [0.77, 1.14] | 0.53 | - |
| All Sites | PFBA | 1.01 [0.99, 1.03] | 0.41 | 0.99 [0.97, 1.01] | 0.53 | - |
| Respiratory System | PFPEA | 1.04 [0.98, 1.1] | 0.16 | 1.02 [0.96, 1.09] | 0.54 | - |
| Descending Colon | PFHXA | 0.99 [0.83, 1.17] | 0.86 | 0.94 [0.77, 1.15] | 0.54 | - |
| Liver | PFHXA | 0.99 [0.9, 1.1] | 0.88 | 0.96 [0.82, 1.11] | 0.54 | - |
| Nasopharynx | PFPEA | 0.96 [0.72, 1.29] | 0.80 | 0.88 [0.59, 1.32] | 0.54 | - |
| Ureter | PFPEA | 0.89 [0.7, 1.15] | 0.38 | 1.11 [0.8, 1.52] | 0.54 | - |
| Myeloma | PFHXA | 0.99 [0.9, 1.08] | 0.76 | 0.97 [0.86, 1.08] | 0.54 | - |
| Other Lymphocytic Leukemia | PFPEA | 0.76 [0.58, 1] | 0.05 | 0.85 [0.5, 1.43] | 0.54 | - |
| Other Digestive Organs | PFBS | 1.07 [0.84, 1.37] | 0.56 | 1.08 [0.84, 1.4] | 0.55 | - |
| Digestive System | PFHpA | 1.01 [0.96, 1.06] | 0.64 | 0.98 [0.93, 1.04] | 0.55 | - |
| Other Acute Leukemia | PFBS | 1.25 [0.85, 1.82] | 0.26 | 1.14 [0.75, 1.74] | 0.55 | - |
| Urinary System | PFHpA | 1.03 [0.96, 1.09] | 0.46 | 0.98 [0.9, 1.06] | 0.55 | - |
| Trachea Mediastinum and Other Respiratory Organs | PFBS | 0.85 [0.56, 1.29] | 0.44 | 1.18 [0.67, 2.09] | 0.56 | - |
| Rectosigmoid Junction | PFHpA | 0.98 [0.8, 1.19] | 0.82 | 0.93 [0.73, 1.19] | 0.56 | - |
| Cecum | PFBA | 1.06 [0.96, 1.18] | 0.23 | 1.03 [0.94, 1.13] | 0.56 | - |
| Small Intestine | PFBS | 1.06 [0.92, 1.23] | 0.41 | 1.04 [0.9, 1.21] | 0.57 | - |
| Oral Cavity and Pharynx | PFHXA | 0.98 [0.92, 1.05] | 0.65 | 1.03 [0.93, 1.14] | 0.57 | - |
| Ovary | PFHpA | NA | NA | 0.97 [0.86, 1.09] | 0.57 | NA |
| Other Endocrine including Thymus | PFBA | 0.84 [0.67, 1.06] | 0.14 | 1.07 [0.85, 1.35] | 0.57 | - |
| Myeloid and Monocytic Leukemia | PFPEA | 1.04 [0.94, 1.14] | 0.46 | 0.97 [0.86, 1.09] | 0.58 | - |
| Larynx | PFBS | 1.08 [0.96, 1.21] | 0.21 | 1.06 [0.87, 1.29] | 0.59 | - |
| Non Hodgkin Lymphoma | PFBA | 1.05 [0.99, 1.11] | 0.10 | 0.98 [0.91, 1.06] | 0.59 | - |
| Lymphoma | PFBA | 1.05 [1, 1.11] | 0.07 | 0.98 [0.91, 1.05] | 0.59 | - |
| Skin excluding Basal and Squamous | PFPEA | 1 [0.92, 1.08] | 0.93 | 0.98 [0.89, 1.07] | 0.59 | - |
| Pancreas | PFBS | 1.01 [0.94, 1.08] | 0.82 | 0.98 [0.91, 1.05] | 0.60 | - |
| Liver and Intrahepatic Bile Duct | PFHXA | 0.99 [0.9, 1.08] | 0.80 | 0.97 [0.85, 1.1] | 0.61 | - |
| NHL Extranodal | PFBA | 1.03 [0.94, 1.14] | 0.51 | 0.97 [0.88, 1.08] | 0.61 | - |
| Uterus NOS | PFBS | NA | NA | 1.06 [0.84, 1.35] | 0.61 | NA |
| Rectum and Rectosigmoid Junction | PFBS | 1.01 [0.94, 1.09] | 0.73 | 0.98 [0.89, 1.07] | 0.61 | - |
| Rectum and Rectosigmoid Junction | PFHXA | 1.02 [0.95, 1.1] | 0.58 | 0.98 [0.88, 1.08] | 0.62 | - |
| Intrahepatic Bile Duct | PFBA | 1.07 [0.92, 1.24] | 0.41 | 0.96 [0.81, 1.14] | 0.62 | - |
| Vulva | PFPEA | NA | NA | 1.04 [0.9, 1.19] | 0.63 | NA |
| Small Intestine | PFHXA | 1.05 [0.91, 1.22] | 0.49 | 1.04 [0.89, 1.21] | 0.63 | - |
| Rectosigmoid Junction | PFBS | 0.89 [0.77, 1.03] | 0.13 | 0.96 [0.8, 1.14] | 0.64 | - |
| Vagina | PFHpA | NA | NA | 0.91 [0.62, 1.34] | 0.64 | NA |
| Rectum | PFPEA | 1.05 [0.97, 1.14] | 0.22 | 1.03 [0.92, 1.14] | 0.64 | - |
| Small Intestine | PFPEA | 1.06 [0.92, 1.22] | 0.44 | 0.97 [0.83, 1.12] | 0.64 | - |
| Ovary | PFBA | NA | NA | 0.98 [0.91, 1.06] | 0.64 | NA |
| Transverse Colon | PFPEA | 0.99 [0.85, 1.14] | 0.85 | 0.97 [0.83, 1.12] | 0.65 | - |
| Floor of Mouth | PFHpA | 1.05 [0.75, 1.47] | 0.76 | 0.89 [0.54, 1.47] | 0.65 | - |
| Acute Lymphocytic Leukemia | PFHpA | 0.93 [0.71, 1.22] | 0.61 | 0.93 [0.69, 1.26] | 0.66 | - |
| Aleukemic Subleukemic and NOS | PFPEA | 0.83 [0.63, 1.1] | 0.20 | 0.94 [0.7, 1.25] | 0.66 | - |
| Acute Myeloid Leukemia | PFBA | 0.99 [0.9, 1.1] | 0.89 | 1.03 [0.91, 1.17] | 0.66 | - |
| Other Acute Leukemia | PFPEA | 1.1 [0.75, 1.61] | 0.61 | 0.91 [0.59, 1.4] | 0.66 | - |
| Other Leukemia | PFBS | 0.92 [0.72, 1.17] | 0.49 | 0.94 [0.73, 1.22] | 0.66 | - |
| Uterus NOS | PFBA | NA | NA | 1.05 [0.85, 1.3] | 0.66 | NA |
| All Sites | PFPEA | 1.02 [0.99, 1.04] | 0.13 | 1.01 [0.98, 1.03] | 0.66 | - |
| Oral Cavity and Pharynx | PFBS | 0.97 [0.91, 1.04] | 0.44 | 1.02 [0.92, 1.13] | 0.66 | - |
| Other Lymphocytic Leukemia | PFHXA | 0.85 [0.64, 1.13] | 0.26 | 1.12 [0.66, 1.9] | 0.67 | - |
| Anus Anal Canal and Anorectum | PFHpA | 1.24 [0.96, 1.61] | 0.10 | 1.04 [0.86, 1.27] | 0.67 | - |
| Lung and Bronchus | PFPEA | 1.04 [0.98, 1.11] | 0.15 | 1.01 [0.95, 1.08] | 0.67 | - |
| Uterus NOS | PFHXA | NA | NA | 1.05 [0.82, 1.34] | 0.69 | NA |
| Chronic Myeloid Leukemia | PFPEA | 1.1 [0.93, 1.3] | 0.25 | 0.96 [0.79, 1.17] | 0.69 | - |
| Rectum | PFBS | 1.05 [0.97, 1.13] | 0.25 | 0.98 [0.88, 1.09] | 0.69 | - |
| Oral Cavity and Pharynx | PFBA | 1 [0.94, 1.06] | 0.93 | 0.98 [0.9, 1.08] | 0.69 | - |
| Cervix Uteri | PFBS | NA | NA | 0.98 [0.87, 1.1] | 0.70 | NA |
| NHL Nodal | PFBA | 1.05 [0.99, 1.12] | 0.10 | 0.98 [0.89, 1.08] | 0.71 | - |
| Breast | PFHpA | 0.86 [0.63, 1.18] | 0.35 | 0.99 [0.95, 1.04] | 0.71 | - |
| Brain | PFBS | 1.04 [0.93, 1.16] | 0.46 | 0.98 [0.87, 1.1] | 0.71 | - |
| Oral Cavity and Pharynx | PFPEA | 0.99 [0.93, 1.06] | 0.81 | 1.02 [0.92, 1.13] | 0.71 | - |
| Floor of Mouth | PFBA | 1.05 [0.84, 1.31] | 0.66 | 0.94 [0.67, 1.32] | 0.71 | - |
| Oropharynx | PFHpA | 0.81 [0.62, 1.08] | 0.15 | 1.1 [0.65, 1.89] | 0.72 | - |
| Trachea Mediastinum and Other Respiratory Organs | PFBA | 0.98 [0.68, 1.42] | 0.92 | 1.1 [0.65, 1.87] | 0.72 | - |
| Stomach | PFBS | 0.96 [0.86, 1.08] | 0.52 | 1.02 [0.9, 1.16] | 0.72 | - |
| Uterus NOS | PFPEA | NA | NA | 0.96 [0.76, 1.21] | 0.72 | NA |
| Peritoneum Omentum and Mesentery | PFBA | 1.09 [0.64, 1.89] | 0.75 | 0.96 [0.77, 1.2] | 0.73 | - |
| Lip | PFBS | 0.99 [0.77, 1.26] | 0.93 | 0.93 [0.63, 1.38] | 0.73 | - |
| Digestive System | PFPEA | 1 [0.96, 1.03] | 0.86 | 1.01 [0.97, 1.05] | 0.73 | - |
| Other Oral Cavity and Pharynx | PFHXA | 0.96 [0.69, 1.34] | 0.81 | 1.1 [0.63, 1.92] | 0.73 | - |
| Larynx | PFBA | 0.96 [0.86, 1.06] | 0.42 | 0.97 [0.81, 1.16] | 0.73 | - |
| Breast | PFBS | 1.03 [0.82, 1.29] | 0.82 | 0.99 [0.96, 1.03] | 0.73 | - |
| Other Leukemia | PFHXA | 0.92 [0.72, 1.17] | 0.49 | 1.05 [0.81, 1.35] | 0.74 | - |
| Brain and Other Nervous System | PFPEA | 0.95 [0.86, 1.05] | 0.33 | 1.02 [0.91, 1.14] | 0.74 | - |
| Splenic Flexure | PFHpA | 0.82 [0.6, 1.13] | 0.23 | 0.94 [0.66, 1.35] | 0.74 | - |
| Corpus Uteri | PFBA | NA | NA | 0.99 [0.94, 1.05] | 0.75 | NA |
| Vagina | PFHXA | NA | NA | 0.95 [0.72, 1.27] | 0.75 | NA |
| Anus Anal Canal and Anorectum | PFBA | 0.98 [0.82, 1.17] | 0.79 | 0.98 [0.86, 1.12] | 0.75 | - |
| Lip | PFHpA | 1.33 [0.97, 1.84] | 0.08 | 0.92 [0.53, 1.58] | 0.75 | - |
| Ovary | PFHXA | NA | NA | 0.99 [0.9, 1.08] | 0.75 | NA |
| Brain | PFHpA | 1.12 [0.96, 1.29] | 0.14 | 0.98 [0.83, 1.14] | 0.76 | - |
| Non Hodgkin Lymphoma | PFHpA | 1.04 [0.96, 1.13] | 0.33 | 0.98 [0.87, 1.1] | 0.76 | - |
| Digestive System | PFBS | 1.03 [0.99, 1.07] | 0.14 | 0.99 [0.95, 1.03] | 0.76 | - |
| NHL Nodal | PFHXA | 1.01 [0.94, 1.09] | 0.74 | 0.98 [0.88, 1.1] | 0.76 | - |
| Nose Nasal Cavity and Middle Ear | PFPEA | 0.88 [0.7, 1.11] | 0.27 | 1.05 [0.78, 1.41] | 0.76 | - |
| NHL Extranodal | PFPEA | 0.98 [0.88, 1.1] | 0.75 | 0.98 [0.88, 1.1] | 0.76 | - |
| Esophagus | PFBS | 1 [0.91, 1.09] | 0.93 | 0.98 [0.83, 1.15] | 0.77 | - |
| Nose Nasal Cavity and Middle Ear | PFBS | 0.86 [0.68, 1.09] | 0.22 | 0.96 [0.71, 1.29] | 0.77 | - |
| Myeloid and Monocytic Leukemia | PFHpA | 1.07 [0.94, 1.22] | 0.28 | 0.98 [0.83, 1.15] | 0.78 | - |
| Corpus and Uterus NOS | PFBA | NA | NA | 0.99 [0.94, 1.05] | 0.78 | NA |
| Tonsil | PFBA | 0.99 [0.89, 1.11] | 0.91 | 0.97 [0.76, 1.23] | 0.79 | - |
| Lymphoma | PFHpA | 1.03 [0.95, 1.11] | 0.50 | 1.02 [0.91, 1.13] | 0.79 | - |
| Stomach | PFHpA | 0.89 [0.77, 1.04] | 0.15 | 1.02 [0.86, 1.21] | 0.79 | - |
| Myeloid and Monocytic Leukemia | PFBS | 1.01 [0.91, 1.11] | 0.88 | 0.98 [0.87, 1.11] | 0.79 | - |
| Ovary | PFBS | NA | NA | 1.01 [0.93, 1.1] | 0.79 | NA |
| Myeloid and Monocytic Leukemia | PFBA | 1.03 [0.94, 1.12] | 0.55 | 1.01 [0.91, 1.13] | 0.79 | - |
| Digestive System | PFBA | 1.03 [0.99, 1.06] | 0.13 | 1 [0.96, 1.03] | 0.80 | - |
| Oropharynx | PFPEA | 0.84 [0.69, 1.03] | 0.09 | 1.05 [0.71, 1.55] | 0.80 | - |
| NHL Nodal | PFHpA | 1.06 [0.96, 1.16] | 0.25 | 0.98 [0.85, 1.13] | 0.80 | - |
| Kidney and Renal Pelvis | PFHpA | 1.06 [0.98, 1.15] | 0.13 | 0.99 [0.88, 1.1] | 0.80 | - |
| Other Digestive Organs | PFHXA | 1.16 [0.91, 1.49] | 0.24 | 1.04 [0.79, 1.35] | 0.80 | - |
| Other Urinary Organs | PFHXA | 0.78 [0.58, 1.03] | 0.08 | 1.05 [0.7, 1.59] | 0.80 | - |
| Peritoneum Omentum and Mesentery | PFHpA | 0.42 [0.14, 1.24] | 0.12 | 0.96 [0.69, 1.33] | 0.81 | - |
| Brain | PFPEA | 0.94 [0.84, 1.04] | 0.23 | 0.99 [0.88, 1.11] | 0.81 | - |
| Vulva | PFBS | NA | NA | 1.02 [0.88, 1.17] | 0.82 | NA |
| Other Acute Leukemia | PFHXA | 0.99 [0.67, 1.48] | 0.97 | 1.05 [0.68, 1.63] | 0.82 | - |
| Vulva | PFBA | NA | NA | 1.01 [0.89, 1.15] | 0.82 | NA |
| NHL Extranodal | PFHpA | 1.01 [0.87, 1.17] | 0.89 | 0.98 [0.84, 1.15] | 0.83 | - |
| Leukemia | PFBA | 0.98 [0.91, 1.05] | 0.54 | 0.99 [0.92, 1.07] | 0.83 | - |
| Female Genital System | PFBA | NA | NA | 1 [0.95, 1.04] | 0.83 | NA |
| Appendix | PFPEA | 1.08 [0.89, 1.31] | 0.44 | 0.98 [0.81, 1.18] | 0.83 | - |
| Hodgkin Lymphoma | PFBA | 1.07 [0.92, 1.24] | 0.36 | 0.98 [0.83, 1.16] | 0.83 | - |
| Nasopharynx | PFHpA | 1.16 [0.79, 1.72] | 0.45 | 0.94 [0.54, 1.64] | 0.84 | - |
| Cervix Uteri | PFHXA | NA | NA | 1.01 [0.9, 1.14] | 0.85 | NA |
| Other Myeloid Monocytic Leukemia | PFHpA | 1.17 [0.6, 2.28] | 0.65 | 0.92 [0.39, 2.15] | 0.85 | - |
| Other Lymphocytic Leukemia | PFBS | 0.82 [0.62, 1.08] | 0.16 | 1.05 [0.62, 1.78] | 0.85 | - |
| Rectum and Rectosigmoid Junction | PFPEA | 1.02 [0.94, 1.09] | 0.67 | 1.01 [0.92, 1.11] | 0.85 | - |
| Female Genital System | PFHpA | NA | NA | 0.99 [0.93, 1.06] | 0.85 | NA |
| Nose Nasal Cavity and Middle Ear | PFBA | 0.9 [0.73, 1.11] | 0.33 | 0.97 [0.74, 1.28] | 0.85 | - |
| Liver | PFPEA | 0.94 [0.86, 1.04] | 0.24 | 0.99 [0.86, 1.14] | 0.85 | - |
| Ascending Colon | PFHpA | 0.96 [0.83, 1.11] | 0.56 | 0.99 [0.85, 1.15] | 0.86 | - |
| Chronic Myeloid Leukemia | PFHpA | 1.09 [0.87, 1.36] | 0.46 | 0.98 [0.75, 1.27] | 0.86 | - |
| Hodgkin Nodal | PFHXA | 0.97 [0.82, 1.15] | 0.74 | 1.02 [0.84, 1.23] | 0.86 | - |
| Aleukemic Subleukemic and NOS | PFHXA | 0.89 [0.67, 1.19] | 0.44 | 1.03 [0.76, 1.38] | 0.86 | - |
| Myeloma | PFBA | 1.03 [0.95, 1.12] | 0.41 | 1.01 [0.91, 1.11] | 0.86 | - |
| Brain and Other Nervous System | PFBS | 1.04 [0.93, 1.15] | 0.50 | 1.01 [0.9, 1.13] | 0.87 | - |
| Hepatic Flexure | PFPEA | 1.17 [0.97, 1.42] | 0.10 | 1.02 [0.83, 1.25] | 0.87 | - |
| Colon and Rectum | PFHXA | 1.03 [0.98, 1.08] | 0.28 | 1 [0.94, 1.05] | 0.88 | - |
| Other Oral Cavity and Pharynx | PFPEA | 0.86 [0.62, 1.2] | 0.38 | 0.96 [0.56, 1.65] | 0.88 | - |
| Descending Colon | PFBS | 0.96 [0.81, 1.14] | 0.63 | 1.01 [0.84, 1.23] | 0.88 | - |
| Colon and Rectum | PFPEA | 1.04 [0.99, 1.09] | 0.12 | 1 [0.95, 1.06] | 0.88 | - |
| Rectosigmoid Junction | PFBA | 0.99 [0.87, 1.12] | 0.82 | 0.99 [0.84, 1.16] | 0.88 | - |
| Nasopharynx | PFBA | 1.07 [0.83, 1.38] | 0.60 | 1.03 [0.72, 1.46] | 0.88 | - |
| Myeloid and Monocytic Leukemia | PFHXA | 0.97 [0.88, 1.08] | 0.59 | 0.99 [0.88, 1.12] | 0.89 | - |
| Hodgkin Nodal | PFBA | 1.07 [0.92, 1.24] | 0.41 | 0.99 [0.84, 1.17] | 0.89 | - |
| Leukemia | PFHXA | 1.07 [0.99, 1.16] | 0.09 | 0.99 [0.91, 1.08] | 0.89 | - |
| Splenic Flexure | PFBA | 1.17 [0.96, 1.43] | 0.12 | 1.02 [0.8, 1.29] | 0.89 | - |
| Salivary Gland | PFPEA | 1.08 [0.91, 1.29] | 0.39 | 0.99 [0.79, 1.24] | 0.90 | - |
| Brain and Other Nervous System | PFHXA | 1.03 [0.93, 1.15] | 0.55 | 0.99 [0.88, 1.12] | 0.91 | - |
| Acute Lymphocytic Leukemia | PFBS | 1.04 [0.85, 1.26] | 0.71 | 1.01 [0.81, 1.26] | 0.91 | - |
| Anus Anal Canal and Anorectum | PFBS | 1.02 [0.84, 1.25] | 0.82 | 0.99 [0.86, 1.15] | 0.91 | - |
| Other Leukemia | PFBA | 1.01 [0.82, 1.25] | 0.93 | 0.99 [0.79, 1.24] | 0.91 | - |
| All Sites | PFBS | 1.02 [0.99, 1.04] | 0.13 | 1 [0.98, 1.02] | 0.92 | - |
| Tongue | PFPEA | 0.93 [0.84, 1.04] | 0.19 | 0.99 [0.85, 1.16] | 0.92 | - |
| Peritoneum Omentum and Mesentery | PFPEA | 1.06 [0.58, 1.94] | 0.85 | 1.01 [0.8, 1.29] | 0.92 | - |
| Other Lymphocytic Leukemia | PFBA | 1.11 [0.87, 1.42] | 0.42 | 0.98 [0.62, 1.54] | 0.92 | - |
| Large Intestine NOS | PFBS | 1.05 [0.87, 1.26] | 0.62 | 1.01 [0.84, 1.21] | 0.93 | - |
| Female Genital System | PFHXA | NA | NA | 1 [0.95, 1.05] | 0.93 | NA |
| Anus Anal Canal and Anorectum | PFPEA | 1.04 [0.85, 1.27] | 0.71 | 1.01 [0.87, 1.16] | 0.93 | - |
| Acute Lymphocytic Leukemia | PFPEA | 0.94 [0.77, 1.14] | 0.54 | 1.01 [0.81, 1.26] | 0.93 | - |
| Colon excluding Rectum | PFHXA | 1.03 [0.97, 1.1] | 0.35 | 1 [0.94, 1.07] | 0.93 | - |
| Corpus Uteri | PFHXA | NA | NA | 1 [0.93, 1.06] | 0.94 | NA |
| Colon excluding Rectum | PFPEA | 1.05 [0.99, 1.11] | 0.11 | 1 [0.94, 1.07] | 0.94 | - |
| Other Lymphocytic Leukemia | PFHpA | 1.19 [0.84, 1.69] | 0.32 | 1.03 [0.48, 2.19] | 0.94 | - |
| Intrahepatic Bile Duct | PFBS | 1.07 [0.9, 1.26] | 0.44 | 1.01 [0.83, 1.22] | 0.94 | - |
| Tonsil | PFHXA | 1 [0.88, 1.14] | 0.95 | 0.99 [0.76, 1.3] | 0.95 | - |
| Cervix Uteri | PFPEA | NA | NA | 1 [0.89, 1.13] | 0.95 | NA |
| Other Acute Leukemia | PFHpA | 1.07 [0.64, 1.79] | 0.81 | 0.98 [0.55, 1.75] | 0.95 | - |
| Peritoneum Omentum and Mesentery | PFHXA | 0.65 [0.33, 1.25] | 0.19 | 1.01 [0.78, 1.29] | 0.96 | - |
| Hepatic Flexure | PFHXA | 1.06 [0.87, 1.29] | 0.59 | 1.01 [0.81, 1.24] | 0.96 | - |
| All Sites | PFHXA | 1.01 [0.99, 1.04] | 0.30 | 1 [0.98, 1.02] | 0.96 | - |
| Colon and Rectum | PFBA | 1.04 [1, 1.09] | 0.05 | 1 [0.95, 1.05] | 0.97 | - |
| Gum and Other Mouth | PFHpA | 0.93 [0.73, 1.18] | 0.55 | 0.99 [0.78, 1.27] | 0.97 | - |
| Intrahepatic Bile Duct | PFHXA | 0.95 [0.8, 1.13] | 0.57 | 1 [0.82, 1.21] | 0.97 | - |
| Esophagus | PFPEA | 1.06 [0.97, 1.16] | 0.21 | 1 [0.86, 1.18] | 0.97 | - |
| Hodgkin Lymphoma | PFHXA | 0.98 [0.82, 1.16] | 0.81 | 1 [0.83, 1.21] | 0.97 | - |
| Other Female Genital Organs | PFHXA | NA | NA | 1 [0.83, 1.21] | 0.98 | NA |
| Chronic Myeloid Leukemia | PFHXA | 0.92 [0.77, 1.09] | 0.33 | 1 [0.82, 1.22] | 0.98 | - |
| Female Genital System | PFPEA | NA | NA | 1 [0.95, 1.05] | 0.98 | NA |
| Corpus and Uterus NOS | PFHXA | NA | NA | 1 [0.94, 1.06] | 0.98 | NA |
| Urinary Bladder | PFBS | 1.05 [0.98, 1.12] | 0.16 | 1 [0.91, 1.1] | 0.99 | - |
| Other Non Epithelial Skin | PFBA | 0.88 [0.77, 1.01] | 0.08 | 1 [0.83, 1.2] | 0.99 | - |
| Rectum | PFHXA | 1.05 [0.96, 1.14] | 0.28 | 1 [0.9, 1.11] | 0.99 | - |
| Ureter | PFBS | 1.04 [0.81, 1.33] | 0.79 | 1 [0.72, 1.39] | 0.99 | - |
| Digestive System | PFHXA | 1 [0.97, 1.04] | 0.82 | 1 [0.96, 1.04] | 1.00 | - |
| Chronic Myeloid Leukemia | PFBS | 1.07 [0.91, 1.26] | 0.42 | 1 [0.82, 1.22] | 1.00 | - |
| Large Intestine NOS | PFPEA | 0.86 [0.72, 1.03] | 0.10 | 1 [0.84, 1.19] | 1.00 | - |
| Testis | PFPEA | 0.89 [0.78, 1.01] | 0.06 | NA | NA | NA |
| Prostate | PFHpA | 1.04 [0.99, 1.1] | 0.11 | NA | NA | NA |
| Male Genital System | PFHpA | 1.04 [0.99, 1.09] | 0.12 | NA | NA | NA |
| Pleura | PFBA | 1.98 [0.7, 5.61] | 0.20 | NA | NA | NA |
| Prostate | PFHXA | 1.03 [0.99, 1.07] | 0.20 | NA | NA | NA |
| Pleura | PFBS | 0.43 [0.11, 1.59] | 0.20 | NA | NA | NA |
| Penis | PFBS | 1.16 [0.92, 1.46] | 0.22 | NA | NA | NA |
| Testis | PFBA | 0.93 [0.83, 1.04] | 0.22 | NA | NA | NA |
| Testis | PFHXA | 0.93 [0.81, 1.06] | 0.25 | NA | NA | NA |
| Male Genital System | PFHXA | 1.02 [0.98, 1.06] | 0.26 | NA | NA | NA |
| Penis | PFHpA | 1.19 [0.88, 1.63] | 0.26 | NA | NA | NA |
| Testis | PFBS | 0.93 [0.82, 1.06] | 0.29 | NA | NA | NA |
| Other Male Genital Organs | PFBA | 0.82 [0.56, 1.2] | 0.30 | NA | NA | NA |
| Penis | PFPEA | 1.13 [0.89, 1.42] | 0.31 | NA | NA | NA |
| Prostate | PFPEA | 1.02 [0.98, 1.06] | 0.35 | NA | NA | NA |
| Penis | PFBA | 1.1 [0.89, 1.36] | 0.36 | NA | NA | NA |
| Other Male Genital Organs | PFBS | 0.83 [0.55, 1.26] | 0.38 | NA | NA | NA |
| Penis | PFHXA | 1.1 [0.87, 1.4] | 0.43 | NA | NA | NA |
| Male Genital System | PFPEA | 1.01 [0.98, 1.05] | 0.51 | NA | NA | NA |
| Pleura | PFHpA | 0.55 [0.1, 3.22] | 0.51 | NA | NA | NA |
| Pleura | PFPEA | 0.74 [0.23, 2.39] | 0.61 | NA | NA | NA |
| Other Male Genital Organs | PFHXA | 1.11 [0.73, 1.69] | 0.63 | NA | NA | NA |
| Other Male Genital Organs | PFPEA | 0.91 [0.6, 1.37] | 0.64 | NA | NA | NA |
| Prostate | PFBA | 1 [0.97, 1.04] | 0.82 | NA | NA | NA |
| Pleura | PFHXA | 0.88 [0.27, 2.85] | 0.83 | NA | NA | NA |
| Testis | PFHpA | 0.99 [0.83, 1.17] | 0.89 | NA | NA | NA |
| Male Genital System | PFBS | 1 [0.96, 1.04] | 0.95 | NA | NA | NA |
| Prostate | PFBS | 1 [0.96, 1.04] | 0.95 | NA | NA | NA |
| Male Genital System | PFBA | 1 [0.97, 1.03] | 0.98 | NA | NA | NA |
| 1. All models were adjusted for county-level SES variables, urbanicity, smoking rate, obesity, and air pollution. | | | | | | |
| 2. cells were highlighted if crude p values were less than 0.05. | | | | | | |
| 3. NAs were produced with limited sample size or when we assessed cancers in male reproductive system among females or vice versa. | | | | | | |
| 4. ++ indicates cancers were significant in both sexes, + indicates cancers were siginificant only in one sex, - indicates cancers were not significant in either sex, NA indicates analysis was done only in one sex or none. | | | | | | |

| **Supplemental Table 18.** Stratified analysis by sex for the associations between MCL violation of PFAS in drinking water using UCMR5 data and cancer incidence between 2016 and 2021. | | | | | | |
| --- | --- | --- | --- | --- | --- | --- |
|  |  | **Male** | | **Female** | |  |
| **Cancers** | **Exposures** | **IRR [95% CI]** | **p value^1,2^** | **IRR [95% CI]** | **p value^1,2^** | **Indicator of Significance^3,4^** |
| Soft Tissue including Heart | PFOA | 1.75 [1.42, 2.16] | 0.00 | 1.32 [1.05, 1.67] | 0.02 | ++ |
| Soft Tissue including Heart | PFOS | 1.66 [1.33, 2.06] | 0.00 | 1.56 [1.24, 1.97] | 0.00 | ++ |
| Other Endocrine including Thymus | PFHxS | 0.22 [0.06, 0.83] | 0.03 | 2.11 [1.11, 4] | 0.02 | ++ |
| Soft Tissue including Heart | PFHxS | 4.13 [2.93, 5.82] | 0.00 | 1.03 [0.64, 1.63] | 0.91 | + |
| Trachea Mediastinum and Other Respiratory Organs | PFHxS | 3.87 [1.66, 9.04] | 0.00 | NA | NA | NA |
| Chronic Lymphocytic Leukemia | PFHxS | 3.32 [2.46, 4.48] | 0.00 | 0.79 [0.54, 1.17] | 0.25 | + |
| Lymphocytic Leukemia | PFHxS | 2.83 [2.17, 3.7] | 0.00 | 0.77 [0.53, 1.1] | 0.15 | + |
| Sigmoid Colon | PFHxS | 2.3 [1.78, 2.97] | 0.00 | 1.05 [0.76, 1.44] | 0.78 | + |
| Leukemia | PFHxS | 1.86 [1.5, 2.29] | 0.00 | 0.83 [0.64, 1.08] | 0.17 | + |
| Urinary Bladder | PFHxS | 1.61 [1.34, 1.93] | 0.00 | 0.84 [0.63, 1.11] | 0.22 | + |
| Chronic Lymphocytic Leukemia | PFOA | 1.53 [1.28, 1.83] | 0.00 | 0.99 [0.81, 1.2] | 0.92 | + |
| Chronic Lymphocytic Leukemia | PFOS | 1.46 [1.22, 1.76] | 0.00 | 0.94 [0.77, 1.16] | 0.58 | + |
| Lymphocytic Leukemia | PFOS | 1.41 [1.2, 1.65] | 0.00 | 0.93 [0.77, 1.12] | 0.45 | + |
| Lymphocytic Leukemia | PFOA | 1.4 [1.2, 1.64] | 0.00 | 0.95 [0.79, 1.14] | 0.61 | + |
| Colon excluding Rectum | PFHxS | 1.39 [1.17, 1.64] | 0.00 | 1.05 [0.87, 1.27] | 0.59 | + |
| Sigmoid Colon | PFOS | 1.38 [1.18, 1.61] | 0.00 | 1.04 [0.87, 1.24] | 0.68 | + |
| Sigmoid Colon | PFOA | 1.37 [1.18, 1.59] | 0.00 | 0.89 [0.75, 1.06] | 0.20 | + |
| Small Intestine | PFOS | 1.29 [1.04, 1.61] | 0.02 | 1.21 [0.96, 1.52] | 0.10 | + |
| Urinary System | PFHxS | 1.28 [1.12, 1.47] | 0.00 | 0.84 [0.7, 1.01] | 0.07 | + |
| Colon and Rectum | PFHxS | 1.22 [1.06, 1.4] | 0.01 | 1.01 [0.85, 1.18] | 0.95 | + |
| Leukemia | PFOS | 1.2 [1.06, 1.36] | 0.00 | 1 [0.87, 1.15] | 0.98 | + |
| Brain and Other Nervous System | PFOA | 1.2 [1.02, 1.4] | 0.03 | 0.95 [0.8, 1.14] | 0.62 | + |
| Brain | PFOA | 1.19 [1.01, 1.41] | 0.04 | 1 [0.84, 1.2] | 0.98 | + |
| Leukemia | PFOA | 1.19 [1.05, 1.34] | 0.01 | 1.07 [0.94, 1.22] | 0.30 | + |
| Urinary Bladder | PFOA | 1.14 [1.03, 1.27] | 0.01 | 1.12 [0.97, 1.29] | 0.12 | + |
| Urinary Bladder | PFOS | 1.14 [1.03, 1.26] | 0.01 | 1.04 [0.9, 1.21] | 0.58 | + |
| Colon excluding Rectum | PFOS | 1.12 [1.02, 1.23] | 0.02 | 1.05 [0.95, 1.17] | 0.35 | + |
| Prostate | PFHxS | 0.87 [0.77, 0.97] | 0.01 | NA | NA | NA |
| Male Genital System | PFHxS | 0.86 [0.77, 0.96] | 0.01 | NA | NA | NA |
| Non Hodgkin Lymphoma | PFHxS | 0.8 [0.66, 0.97] | 0.02 | 1.04 [0.81, 1.34] | 0.75 | + |
| Lymphoma | PFHxS | 0.75 [0.63, 0.91] | 0.00 | 1.11 [0.87, 1.4] | 0.41 | + |
| Aleukemic Subleukemic and NOS | PFOS | 0.54 [0.31, 0.93] | 0.03 | 0.61 [0.35, 1.06] | 0.08 | + |
| Anus Anal Canal and Anorectum | PFHxS | 0.43 [0.18, 0.99] | 0.05 | 1.11 [0.73, 1.7] | 0.61 | + |
| Hodgkin Nodal | PFHxS | 0.37 [0.19, 0.71] | 0.00 | 1.54 [0.93, 2.55] | 0.09 | + |
| Hodgkin Lymphoma | PFHxS | 0.36 [0.19, 0.7] | 0.00 | 1.52 [0.92, 2.5] | 0.10 | + |
| Splenic Flexure | PFHxS | 0.26 [0.09, 0.78] | 0.02 | 0.38 [0.12, 1.21] | 0.10 | + |
| Aleukemic Subleukemic and NOS | PFHxS | 0.22 [0.05, 0.96] | 0.04 | 0.77 [0.31, 1.95] | 0.59 | + |
| Nasopharynx | PFHxS | 0.14 [0.02, 0.96] | 0.05 | 0.44 [0.08, 2.56] | 0.36 | + |
| Hypopharynx | PFHxS | 0.44 [0.19, 1.04] | 0.06 | 6.47 [3.37, 12.43] | 0.00 | + |
| Acute Monocytic Leukemia | PFOS | 0.7 [0.3, 1.61] | 0.40 | 4.21 [2.25, 7.88] | 0.00 | + |
| Acute Monocytic Leukemia | PFOA | 0.38 [0.13, 1.05] | 0.06 | 3.96 [2.15, 7.3] | 0.00 | + |
| Hypopharynx | PFOS | 0.93 [0.65, 1.35] | 0.71 | 2.92 [1.82, 4.69] | 0.00 | + |
| Other Oral Cavity and Pharynx | PFOS | 0.84 [0.49, 1.46] | 0.54 | 2.71 [1.41, 5.19] | 0.00 | + |
| Hypopharynx | PFOA | 1.06 [0.75, 1.5] | 0.75 | 2.55 [1.57, 4.13] | 0.00 | + |
| Cranial Nerves Other Nervous System | PFOS | 1.34 [0.82, 2.18] | 0.24 | 1.97 [1.23, 3.15] | 0.00 | + |
| Ureter | PFOA | 1 [0.68, 1.48] | 1.00 | 1.62 [1.05, 2.5] | 0.03 | + |
| Other Digestive Organs | PFOA | 1.03 [0.7, 1.51] | 0.88 | 1.6 [1.12, 2.28] | 0.01 | + |
| Intrahepatic Bile Duct | PFOS | 0.92 [0.7, 1.21] | 0.53 | 1.5 [1.14, 1.98] | 0.00 | + |
| Salivary Gland | PFOS | 0.88 [0.65, 1.18] | 0.39 | 1.45 [1.04, 2.02] | 0.03 | + |
| Intrahepatic Bile Duct | PFOA | 0.88 [0.67, 1.15] | 0.34 | 1.34 [1.01, 1.77] | 0.04 | + |
| Tongue | PFOA | 1.07 [0.91, 1.26] | 0.42 | 1.28 [1.02, 1.61] | 0.03 | + |
| Oral Cavity and Pharynx | PFOS | 0.92 [0.83, 1.03] | 0.14 | 1.25 [1.07, 1.46] | 0.00 | + |
| Myeloid and Monocytic Leukemia | PFOA | 0.9 [0.77, 1.06] | 0.20 | 1.22 [1.02, 1.46] | 0.03 | + |
| Ovary | PFOA | NA | NA | 0.84 [0.73, 0.97] | 0.02 | NA |
| Cervix Uteri | PFOS | NA | NA | 0.82 [0.67, 1] | 0.05 | NA |
| Cervix Uteri | PFHxS | NA | NA | 0.62 [0.43, 0.91] | 0.01 | NA |
| Splenic Flexure | PFOA | 0.81 [0.56, 1.18] | 0.27 | 0.6 [0.36, 0.97] | 0.04 | + |
| Other Leukemia | PFOS | 0.75 [0.49, 1.14] | 0.18 | 0.52 [0.32, 0.87] | 0.01 | + |
| Esophagus | PFHxS | 0.84 [0.63, 1.11] | 0.23 | 0.46 [0.24, 0.86] | 0.02 | + |
| Other Acute Leukemia | PFOS | 1.25 [0.69, 2.27] | 0.47 | 0.26 [0.08, 0.83] | 0.02 | + |
| Thyroid | PFHxS | 1.27 [0.92, 1.76] | 0.15 | 0.77 [0.58, 1] | 0.05 | - |
| Endocrine System | PFOS | 0.96 [0.81, 1.14] | 0.63 | 1.14 [1, 1.31] | 0.05 | - |
| Nasopharynx | PFOA | 0.76 [0.46, 1.27] | 0.30 | 1.69 [0.99, 2.89] | 0.05 | - |
| Oral Cavity and Pharynx | PFOA | 0.96 [0.86, 1.06] | 0.42 | 1.16 [1, 1.35] | 0.06 | - |
| Cranial Nerves Other Nervous System | PFOA | 1.19 [0.72, 1.94] | 0.50 | 0.55 [0.3, 1.03] | 0.06 | - |
| Vagina | PFOA | NA | NA | 0.62 [0.38, 1.03] | 0.06 | NA |
| Cervix Uteri | PFOA | NA | NA | 0.83 [0.69, 1.01] | 0.06 | NA |
| Respiratory System | PFHxS | 1.04 [0.88, 1.24] | 0.62 | 0.84 [0.69, 1.01] | 0.07 | - |
| Other Oral Cavity and Pharynx | PFOA | 1.3 [0.81, 2.08] | 0.27 | 1.9 [0.95, 3.78] | 0.07 | - |
| Other Biliary | PFOA | 0.82 [0.62, 1.09] | 0.18 | 0.75 [0.55, 1.02] | 0.07 | - |
| Pancreas | PFOA | 0.95 [0.84, 1.06] | 0.32 | 0.9 [0.8, 1.01] | 0.07 | - |
| Vagina | PFOS | NA | NA | 0.63 [0.38, 1.06] | 0.08 | NA |
| Thyroid | PFOS | 1 [0.83, 1.2] | 0.98 | 1.13 [0.98, 1.31] | 0.08 | - |
| Trachea Mediastinum and Other Respiratory Organs | PFOS | 0.94 [0.48, 1.84] | 0.86 | 0.21 [0.04, 1.22] | 0.08 | - |
| Ascending Colon | PFHxS | 0.93 [0.67, 1.3] | 0.68 | 1.3 [0.96, 1.77] | 0.09 | - |
| Lung and Bronchus | PFHxS | 1.04 [0.87, 1.23] | 0.69 | 0.85 [0.7, 1.03] | 0.09 | - |
| Acute Myeloid Leukemia | PFOA | 0.96 [0.81, 1.15] | 0.68 | 1.2 [0.97, 1.49] | 0.09 | - |
| Vagina | PFHxS | NA | NA | 0.42 [0.15, 1.16] | 0.09 | NA |
| Female Genital System | PFHxS | NA | NA | 0.88 [0.76, 1.02] | 0.10 | NA |
| Cecum | PFHxS | 0.96 [0.69, 1.34] | 0.81 | 1.27 [0.96, 1.69] | 0.10 | - |
| NHL Nodal | PFOA | 1.03 [0.93, 1.15] | 0.55 | 0.87 [0.73, 1.03] | 0.11 | - |
| Intrahepatic Bile Duct | PFHxS | 0.79 [0.45, 1.4] | 0.42 | 1.5 [0.92, 2.45] | 0.11 | - |
| Other Endocrine including Thymus | PFOA | 0.93 [0.62, 1.39] | 0.71 | 1.37 [0.93, 2.01] | 0.11 | - |
| Hodgkin Nodal | PFOS | 0.82 [0.61, 1.09] | 0.16 | 1.25 [0.94, 1.67] | 0.13 | - |
| Cranial Nerves Other Nervous System | PFHxS | 0.63 [0.19, 2.03] | 0.44 | 0.25 [0.04, 1.55] | 0.13 | - |
| Nose Nasal Cavity and Middle Ear | PFHxS | 0.91 [0.46, 1.81] | 0.79 | 0.34 [0.08, 1.4] | 0.14 | - |
| Liver and Intrahepatic Bile Duct | PFOS | 0.99 [0.85, 1.14] | 0.84 | 1.16 [0.95, 1.4] | 0.14 | - |
| Endocrine System | PFHxS | 1.13 [0.83, 1.53] | 0.44 | 0.82 [0.64, 1.07] | 0.14 | - |
| Hodgkin Lymphoma | PFOS | 0.82 [0.62, 1.09] | 0.17 | 1.23 [0.93, 1.64] | 0.15 | - |
| Other Non Epithelial Skin | PFOS | 0.94 [0.73, 1.21] | 0.63 | 1.25 [0.92, 1.7] | 0.16 | - |
| Retroperitoneum | PFHxS | 1.19 [0.47, 2.98] | 0.72 | 0.33 [0.07, 1.55] | 0.16 | - |
| Skin excluding Basal and Squamous | PFHxS | 0.86 [0.69, 1.09] | 0.22 | 1.21 [0.93, 1.58] | 0.16 | - |
| Cecum | PFOS | 1 [0.84, 1.2] | 0.99 | 1.12 [0.96, 1.32] | 0.16 | - |
| Myeloid and Monocytic Leukemia | PFOS | 0.94 [0.8, 1.1] | 0.45 | 1.14 [0.95, 1.37] | 0.17 | - |
| Chronic Myeloid Leukemia | PFOS | 0.99 [0.76, 1.3] | 0.97 | 1.24 [0.91, 1.67] | 0.17 | - |
| Nose Nasal Cavity and Middle Ear | PFOS | 0.74 [0.49, 1.11] | 0.15 | 0.68 [0.39, 1.19] | 0.18 | - |
| Other Lymphocytic Leukemia | PFOA | 1.06 [0.7, 1.62] | 0.78 | 0.45 [0.14, 1.45] | 0.18 | - |
| Rectosigmoid Junction | PFOS | 1.08 [0.86, 1.36] | 0.52 | 0.82 [0.61, 1.1] | 0.19 | - |
| Stomach | PFHxS | 1 [0.71, 1.4] | 0.99 | 0.76 [0.51, 1.14] | 0.19 | - |
| Other Lymphocytic Leukemia | PFOS | 1.4 [0.94, 2.09] | 0.10 | 0.46 [0.15, 1.45] | 0.19 | - |
| Melanoma of the Skin | PFHxS | 0.88 [0.7, 1.12] | 0.31 | 1.21 [0.91, 1.59] | 0.19 | - |
| Larynx | PFOS | 0.99 [0.82, 1.19] | 0.91 | 0.8 [0.57, 1.12] | 0.19 | - |
| Other Endocrine including Thymus | PFOS | 0.71 [0.45, 1.11] | 0.13 | 1.3 [0.87, 1.93] | 0.20 | - |
| Rectum and Rectosigmoid Junction | PFOS | 0.99 [0.88, 1.12] | 0.87 | 0.9 [0.77, 1.06] | 0.20 | - |
| Endocrine System | PFOA | 1.04 [0.88, 1.22] | 0.66 | 1.09 [0.95, 1.24] | 0.21 | - |
| Acute Lymphocytic Leukemia | PFHxS | 0.98 [0.54, 1.75] | 0.94 | 0.62 [0.3, 1.31] | 0.21 | - |
| Large Intestine NOS | PFOA | 0.85 [0.63, 1.14] | 0.28 | 1.19 [0.91, 1.56] | 0.21 | - |
| Respiratory System | PFOS | 0.97 [0.89, 1.07] | 0.57 | 0.94 [0.84, 1.04] | 0.21 | - |
| Hepatic Flexure | PFOS | 1.27 [0.95, 1.7] | 0.11 | 1.22 [0.89, 1.65] | 0.22 | - |
| All Sites | PFHxS | 1.06 [0.99, 1.14] | 0.12 | 0.96 [0.89, 1.03] | 0.22 | - |
| Tongue | PFHxS | 0.79 [0.57, 1.11] | 0.17 | 0.73 [0.45, 1.21] | 0.22 | - |
| Ureter | PFOS | 1.05 [0.71, 1.56] | 0.80 | 1.34 [0.84, 2.15] | 0.22 | - |
| NHL Extranodal | PFOA | 0.84 [0.71, 1.01] | 0.06 | 1.12 [0.93, 1.33] | 0.22 | - |
| Brain and Other Nervous System | PFOS | 1.04 [0.88, 1.23] | 0.67 | 1.12 [0.93, 1.34] | 0.23 | - |
| Other Female Genital Organs | PFHxS | NA | NA | 0.7 [0.4, 1.25] | 0.23 | NA |
| Corpus and Uterus NOS | PFOS | NA | NA | 1.06 [0.96, 1.17] | 0.23 | NA |
| Bones and Joints | PFOA | 1.16 [0.81, 1.66] | 0.42 | 0.78 [0.51, 1.18] | 0.23 | - |
| Vulva | PFOS | NA | NA | 1.15 [0.92, 1.43] | 0.23 | NA |
| Ovary | PFHxS | NA | NA | 0.85 [0.65, 1.11] | 0.24 | NA |
| Nose Nasal Cavity and Middle Ear | PFOA | 0.88 [0.6, 1.28] | 0.50 | 0.74 [0.44, 1.23] | 0.24 | - |
| Liver | PFHxS | 1.01 [0.76, 1.34] | 0.96 | 0.76 [0.48, 1.2] | 0.24 | - |
| Appendix | PFOA | 0.73 [0.52, 1.02] | 0.06 | 1.19 [0.89, 1.58] | 0.24 | - |
| Larynx | PFHxS | 1.08 [0.77, 1.5] | 0.66 | 0.68 [0.35, 1.31] | 0.24 | - |
| Uterus NOS | PFHxS | NA | NA | 0.6 [0.25, 1.43] | 0.25 | NA |
| Stomach | PFOA | 0.88 [0.73, 1.05] | 0.16 | 1.12 [0.92, 1.36] | 0.25 | - |
| Kidney and Renal Pelvis | PFHxS | 0.9 [0.76, 1.07] | 0.22 | 0.87 [0.68, 1.11] | 0.26 | - |
| Female Genital System | PFOA | NA | NA | 0.96 [0.88, 1.03] | 0.26 | NA |
| Corpus Uteri | PFOS | NA | NA | 1.06 [0.96, 1.17] | 0.26 | NA |
| NHL Nodal | PFOS | 0.96 [0.86, 1.08] | 0.52 | 0.91 [0.76, 1.08] | 0.27 | - |
| Rectum and Rectosigmoid Junction | PFOA | 1.01 [0.9, 1.13] | 0.93 | 0.92 [0.79, 1.07] | 0.27 | - |
| Oropharynx | PFOA | 0.89 [0.65, 1.22] | 0.47 | 1.38 [0.78, 2.44] | 0.27 | - |
| Appendix | PFHxS | 0.61 [0.31, 1.19] | 0.15 | 0.72 [0.4, 1.3] | 0.28 | - |
| Esophagus | PFOS | 0.88 [0.75, 1.02] | 0.09 | 0.86 [0.66, 1.13] | 0.28 | - |
| Ureter | PFHxS | 1.43 [0.74, 2.73] | 0.28 | 0.46 [0.11, 1.91] | 0.28 | - |
| Lung and Bronchus | PFOS | 0.98 [0.89, 1.07] | 0.60 | 0.95 [0.85, 1.05] | 0.29 | - |
| Hepatic Flexure | PFHxS | 0.97 [0.55, 1.71] | 0.92 | 1.34 [0.78, 2.29] | 0.29 | - |
| Thyroid | PFOA | 1.06 [0.89, 1.26] | 0.55 | 1.08 [0.94, 1.24] | 0.30 | - |
| Myeloma | PFHxS | 0.81 [0.61, 1.07] | 0.13 | 1.17 [0.87, 1.59] | 0.30 | - |
| Tonsil | PFOA | 0.89 [0.73, 1.08] | 0.23 | 0.79 [0.51, 1.23] | 0.30 | - |
| Rectum | PFHxS | 0.9 [0.7, 1.14] | 0.36 | 0.85 [0.62, 1.16] | 0.30 | - |
| Acute Monocytic Leukemia | PFHxS | 0.27 [0.02, 3.34] | 0.31 | 0.09 [0, 9.2] | 0.31 | - |
| Myeloma | PFOS | 1.07 [0.93, 1.24] | 0.33 | 0.91 [0.76, 1.09] | 0.31 | - |
| Retroperitoneum | PFOS | 1.02 [0.62, 1.69] | 0.92 | 0.71 [0.36, 1.38] | 0.31 | - |
| Kidney and Renal Pelvis | PFOA | 0.96 [0.88, 1.05] | 0.39 | 0.94 [0.82, 1.07] | 0.32 | - |
| Ascending Colon | PFOA | 0.89 [0.75, 1.06] | 0.18 | 1.09 [0.92, 1.29] | 0.32 | - |
| Other Leukemia | PFOA | 0.86 [0.58, 1.27] | 0.45 | 0.81 [0.53, 1.24] | 0.34 | - |
| Bones and Joints | PFHxS | 0.91 [0.45, 1.85] | 0.79 | 0.66 [0.29, 1.54] | 0.34 | - |
| Tongue | PFOS | 1.01 [0.85, 1.2] | 0.92 | 1.12 [0.88, 1.43] | 0.34 | - |
| Lung and Bronchus | PFOA | 0.95 [0.87, 1.05] | 0.31 | 0.95 [0.86, 1.05] | 0.35 | - |
| Ascending Colon | PFOS | 0.97 [0.81, 1.16] | 0.73 | 1.09 [0.91, 1.29] | 0.35 | - |
| Nasopharynx | PFOS | 0.76 [0.45, 1.29] | 0.31 | 1.33 [0.73, 2.4] | 0.35 | - |
| Respiratory System | PFOA | 0.95 [0.87, 1.04] | 0.30 | 0.95 [0.86, 1.05] | 0.35 | - |
| Other Female Genital Organs | PFOS | NA | NA | 0.87 [0.65, 1.17] | 0.36 | NA |
| Lip | PFOS | 1.27 [0.87, 1.86] | 0.22 | 1.3 [0.74, 2.28] | 0.36 | - |
| Corpus and Uterus NOS | PFOA | NA | NA | 1.05 [0.95, 1.15] | 0.37 | NA |
| Gum and Other Mouth | PFOS | 0.86 [0.64, 1.15] | 0.30 | 1.14 [0.86, 1.51] | 0.37 | - |
| Large Intestine NOS | PFOS | 0.91 [0.68, 1.24] | 0.56 | 0.87 [0.65, 1.18] | 0.37 | - |
| Rectum | PFOA | 1.01 [0.89, 1.15] | 0.85 | 0.93 [0.79, 1.09] | 0.37 | - |
| Digestive System | PFHxS | 1.09 [0.98, 1.22] | 0.12 | 0.95 [0.84, 1.07] | 0.37 | - |
| Rectum | PFOS | 0.97 [0.85, 1.1] | 0.60 | 0.93 [0.78, 1.1] | 0.38 | - |
| Descending Colon | PFHxS | 1.01 [0.62, 1.65] | 0.97 | 0.75 [0.4, 1.41] | 0.38 | - |
| Rectum and Rectosigmoid Junction | PFHxS | 0.88 [0.71, 1.1] | 0.27 | 0.88 [0.66, 1.17] | 0.38 | - |
| Corpus Uteri | PFOA | NA | NA | 1.04 [0.95, 1.15] | 0.38 | NA |
| Hodgkin Nodal | PFOA | 0.79 [0.6, 1.05] | 0.10 | 1.13 [0.85, 1.51] | 0.39 | - |
| Esophagus | PFOA | 0.97 [0.84, 1.12] | 0.72 | 0.89 [0.69, 1.16] | 0.39 | - |
| Other Digestive Organs | PFHxS | 1.59 [0.86, 2.95] | 0.14 | 0.68 [0.28, 1.65] | 0.39 | - |
| Hodgkin Extranodal | PFOA | 0.71 [0.15, 3.34] | 0.67 | 0.32 [0.02, 4.39] | 0.39 | - |
| Other Biliary | PFOS | 0.85 [0.63, 1.13] | 0.27 | 0.88 [0.65, 1.19] | 0.40 | - |
| NHL Extranodal | PFOS | 1.11 [0.93, 1.32] | 0.25 | 1.08 [0.9, 1.3] | 0.40 | - |
| Other Acute Leukemia | PFOA | 1.3 [0.73, 2.29] | 0.37 | 0.72 [0.33, 1.57] | 0.40 | - |
| Liver | PFOA | 1.07 [0.92, 1.24] | 0.39 | 0.91 [0.72, 1.14] | 0.40 | - |
| Other Non Epithelial Skin | PFHxS | 0.65 [0.38, 1.1] | 0.11 | 1.27 [0.72, 2.23] | 0.40 | - |
| Gum and Other Mouth | PFOA | 0.87 [0.65, 1.15] | 0.33 | 1.13 [0.85, 1.49] | 0.41 | - |
| Other Myeloid Monocytic Leukemia | PFHxS | 2.61 [0.94, 7.24] | 0.06 | 0.18 [0, 10.66] | 0.41 | - |
| Rectosigmoid Junction | PFOA | 0.98 [0.78, 1.23] | 0.89 | 0.89 [0.67, 1.18] | 0.41 | - |
| Hodgkin Extranodal | PFOS | 1.32 [0.34, 5.15] | 0.69 | 0.31 [0.02, 5.24] | 0.41 | - |
| Other Lymphocytic Leukemia | PFHxS | 0.64 [0.23, 1.77] | 0.39 | 1.64 [0.5, 5.39] | 0.41 | - |
| Myeloma | PFOA | 0.96 [0.83, 1.11] | 0.59 | 1.07 [0.91, 1.27] | 0.42 | - |
| Aleukemic Subleukemic and NOS | PFOA | 0.67 [0.41, 1.09] | 0.11 | 0.82 [0.51, 1.32] | 0.42 | - |
| Non Hodgkin Lymphoma | PFOA | 0.98 [0.89, 1.08] | 0.63 | 0.95 [0.83, 1.08] | 0.43 | - |
| Hodgkin Lymphoma | PFOA | 0.79 [0.6, 1.04] | 0.09 | 1.12 [0.84, 1.48] | 0.43 | - |
| Anus Anal Canal and Anorectum | PFOS | 1.05 [0.77, 1.45] | 0.74 | 1.1 [0.87, 1.38] | 0.44 | - |
| Skin excluding Basal and Squamous | PFOS | 0.98 [0.87, 1.11] | 0.78 | 1.06 [0.91, 1.23] | 0.45 | - |
| Transverse Colon | PFOA | 0.85 [0.67, 1.07] | 0.17 | 1.09 [0.87, 1.37] | 0.47 | - |
| Other Non Epithelial Skin | PFOA | 0.8 [0.62, 1.04] | 0.09 | 1.12 [0.82, 1.53] | 0.47 | - |
| Pancreas | PFHxS | 1.04 [0.85, 1.28] | 0.69 | 0.93 [0.75, 1.15] | 0.49 | - |
| Other Urinary Organs | PFHxS | 0.96 [0.41, 2.24] | 0.93 | 0.62 [0.15, 2.48] | 0.50 | - |
| Peritoneum Omentum and Mesentery | PFOS | 0.45 [0.11, 1.75] | 0.25 | 0.87 [0.58, 1.31] | 0.50 | - |
| Other Acute Leukemia | PFHxS | 0.84 [0.26, 2.73] | 0.77 | 0.56 [0.1, 3.15] | 0.51 | - |
| Acute Myeloid Leukemia | PFHxS | 0.81 [0.57, 1.15] | 0.25 | 0.87 [0.56, 1.33] | 0.51 | - |
| Pancreas | PFOS | 0.92 [0.82, 1.04] | 0.17 | 0.96 [0.86, 1.08] | 0.52 | - |
| Gallbladder | PFOA | 1.29 [0.89, 1.86] | 0.18 | 0.9 [0.64, 1.25] | 0.52 | - |
| Stomach | PFOS | 0.88 [0.73, 1.06] | 0.18 | 1.07 [0.87, 1.31] | 0.52 | - |
| Uterus NOS | PFOS | NA | NA | 1.13 [0.78, 1.63] | 0.52 | NA |
| Tonsil | PFOS | 0.84 [0.69, 1.04] | 0.11 | 0.86 [0.55, 1.35] | 0.52 | - |
| Oropharynx | PFOS | 0.81 [0.58, 1.14] | 0.23 | 1.22 [0.66, 2.26] | 0.52 | - |
| Floor of Mouth | PFOS | 0.72 [0.46, 1.13] | 0.16 | 1.19 [0.69, 2.08] | 0.53 | - |
| Transverse Colon | PFHxS | 0.7 [0.43, 1.15] | 0.16 | 0.87 [0.55, 1.37] | 0.54 | - |
| Oropharynx | PFHxS | 0.7 [0.37, 1.31] | 0.27 | 0.68 [0.2, 2.3] | 0.54 | - |
| Myeloid and Monocytic Leukemia | PFHxS | 0.82 [0.61, 1.11] | 0.20 | 0.9 [0.63, 1.28] | 0.54 | - |
| Other Leukemia | PFHxS | 0.44 [0.18, 1.11] | 0.08 | 0.78 [0.34, 1.77] | 0.55 | - |
| Acute Lymphocytic Leukemia | PFOA | 0.8 [0.58, 1.11] | 0.19 | 0.9 [0.63, 1.28] | 0.55 | - |
| Hepatic Flexure | PFOA | 1.24 [0.93, 1.65] | 0.15 | 0.91 [0.66, 1.26] | 0.56 | - |
| Melanoma of the Skin | PFOS | 0.99 [0.87, 1.12] | 0.82 | 1.05 [0.9, 1.22] | 0.56 | - |
| Salivary Gland | PFOA | 0.74 [0.55, 1] | 0.05 | 0.9 [0.62, 1.3] | 0.57 | - |
| Ovary | PFOS | NA | NA | 0.96 [0.83, 1.11] | 0.57 | NA |
| Other Myeloid Monocytic Leukemia | PFOA | 0.75 [0.31, 1.81] | 0.52 | 1.28 [0.54, 3.05] | 0.57 | - |
| Digestive System | PFOA | 1.01 [0.95, 1.07] | 0.74 | 0.98 [0.92, 1.05] | 0.58 | - |
| All Sites | PFOS | 1.01 [0.97, 1.05] | 0.53 | 1.01 [0.97, 1.05] | 0.58 | - |
| Non Hodgkin Lymphoma | PFOS | 1.01 [0.91, 1.11] | 0.89 | 0.96 [0.84, 1.11] | 0.59 | - |
| Lip | PFOA | 0.94 [0.64, 1.39] | 0.77 | 1.17 [0.66, 2.1] | 0.59 | - |
| Trachea Mediastinum and Other Respiratory Organs | PFOA | 0.92 [0.48, 1.78] | 0.81 | 0.76 [0.27, 2.12] | 0.60 | - |
| Descending Colon | PFOA | 1.06 [0.81, 1.37] | 0.68 | 1.08 [0.81, 1.44] | 0.60 | - |
| Other Biliary | PFHxS | 0.65 [0.36, 1.17] | 0.15 | 0.87 [0.5, 1.52] | 0.62 | - |
| NHL Nodal | PFHxS | 0.84 [0.68, 1.04] | 0.11 | 1.08 [0.79, 1.47] | 0.62 | - |
| Other Urinary Organs | PFOA | 0.85 [0.54, 1.33] | 0.48 | 0.85 [0.44, 1.64] | 0.63 | - |
| Lymphoma | PFOA | 0.96 [0.87, 1.05] | 0.34 | 0.97 [0.85, 1.1] | 0.63 | - |
| Breast | PFHxS | 1.3 [0.69, 2.44] | 0.42 | 1.02 [0.93, 1.13] | 0.64 | - |
| Digestive System | PFOS | 1.01 [0.95, 1.07] | 0.76 | 1.02 [0.95, 1.08] | 0.65 | - |
| Bones and Joints | PFOS | 1.05 [0.72, 1.53] | 0.78 | 0.91 [0.61, 1.38] | 0.67 | - |
| Vulva | PFHxS | NA | NA | 0.91 [0.59, 1.41] | 0.68 | NA |
| Hodgkin Extranodal | PFHxS | NA | NA | 0.51 [0.02, 14.34] | 0.69 | NA |
| Salivary Gland | PFHxS | 1.07 [0.64, 1.78] | 0.80 | 1.13 [0.59, 2.18] | 0.71 | - |
| Liver and Intrahepatic Bile Duct | PFOA | 1.04 [0.91, 1.2] | 0.54 | 1.04 [0.85, 1.25] | 0.72 | - |
| Uterus NOS | PFOA | NA | NA | 1.07 [0.74, 1.54] | 0.73 | NA |
| Urinary System | PFOS | 1.04 [0.97, 1.13] | 0.28 | 1.02 [0.92, 1.12] | 0.74 | - |
| Acute Lymphocytic Leukemia | PFOS | 0.94 [0.68, 1.3] | 0.70 | 0.94 [0.66, 1.35] | 0.74 | - |
| Large Intestine NOS | PFHxS | 1.26 [0.75, 2.11] | 0.38 | 0.91 [0.53, 1.56] | 0.74 | - |
| Vulva | PFOA | NA | NA | 1.04 [0.83, 1.3] | 0.74 | NA |
| Skin excluding Basal and Squamous | PFOA | 0.94 [0.83, 1.06] | 0.29 | 1.03 [0.89, 1.19] | 0.74 | - |
| Other Female Genital Organs | PFOA | NA | NA | 0.95 [0.72, 1.27] | 0.74 | NA |
| Acute Myeloid Leukemia | PFOS | 0.92 [0.76, 1.1] | 0.36 | 0.96 [0.76, 1.21] | 0.75 | - |
| Colon and Rectum | PFOA | 1.04 [0.97, 1.13] | 0.26 | 0.99 [0.9, 1.08] | 0.75 | - |
| Corpus Uteri | PFHxS | NA | NA | 1.03 [0.86, 1.24] | 0.76 | NA |
| Small Intestine | PFHxS | 0.86 [0.55, 1.33] | 0.49 | 0.93 [0.59, 1.47] | 0.76 | - |
| Cecum | PFOA | 0.92 [0.77, 1.1] | 0.38 | 0.97 [0.83, 1.15] | 0.76 | - |
| Tonsil | PFHxS | 0.84 [0.57, 1.25] | 0.40 | 1.12 [0.53, 2.39] | 0.76 | - |
| Peritoneum Omentum and Mesentery | PFHxS | 0.39 [0.04, 3.71] | 0.41 | 0.88 [0.4, 1.95] | 0.76 | - |
| Breast | PFOA | 1.29 [0.92, 1.82] | 0.15 | 1.01 [0.96, 1.06] | 0.77 | - |
| Chronic Myeloid Leukemia | PFHxS | 0.76 [0.45, 1.28] | 0.31 | 1.09 [0.62, 1.89] | 0.77 | - |
| NHL Extranodal | PFHxS | 0.72 [0.51, 1.03] | 0.07 | 0.95 [0.68, 1.34] | 0.77 | - |
| Brain | PFHxS | 1.1 [0.8, 1.51] | 0.56 | 1.05 [0.75, 1.48] | 0.77 | - |
| Floor of Mouth | PFHxS | 0.42 [0.15, 1.17] | 0.10 | 0.84 [0.26, 2.77] | 0.78 | - |
| Female Genital System | PFOS | NA | NA | 0.99 [0.91, 1.07] | 0.78 | NA |
| Gallbladder | PFHxS | 1.3 [0.65, 2.61] | 0.45 | 0.92 [0.49, 1.71] | 0.79 | - |
| Gum and Other Mouth | PFHxS | 1.04 [0.62, 1.74] | 0.88 | 0.93 [0.53, 1.61] | 0.79 | - |
| Other Myeloid Monocytic Leukemia | PFOS | 1.12 [0.51, 2.43] | 0.78 | 1.14 [0.44, 2.92] | 0.79 | - |
| Peritoneum Omentum and Mesentery | PFOA | 0.45 [0.12, 1.66] | 0.23 | 1.05 [0.73, 1.52] | 0.79 | - |
| Descending Colon | PFOS | 0.92 [0.7, 1.21] | 0.55 | 1.04 [0.77, 1.41] | 0.80 | - |
| Colon excluding Rectum | PFOA | 1.06 [0.97, 1.17] | 0.21 | 1.01 [0.91, 1.12] | 0.81 | - |
| Melanoma of the Skin | PFOA | 0.95 [0.84, 1.08] | 0.42 | 1.02 [0.88, 1.19] | 0.81 | - |
| Floor of Mouth | PFOA | 1.25 [0.87, 1.8] | 0.22 | 0.94 [0.52, 1.7] | 0.84 | - |
| Colon and Rectum | PFOS | 1.08 [1, 1.17] | 0.06 | 1.01 [0.92, 1.1] | 0.85 | - |
| Corpus and Uterus NOS | PFHxS | NA | NA | 1.02 [0.85, 1.22] | 0.85 | NA |
| Brain | PFOS | 1.02 [0.85, 1.21] | 0.84 | 1.02 [0.84, 1.23] | 0.85 | - |
| Splenic Flexure | PFOS | 0.93 [0.64, 1.34] | 0.70 | 0.96 [0.63, 1.48] | 0.86 | - |
| Other Digestive Organs | PFOS | 1.03 [0.69, 1.52] | 0.89 | 1.04 [0.69, 1.56] | 0.86 | - |
| Lip | PFHxS | 1.14 [0.57, 2.3] | 0.70 | 1.09 [0.37, 3.24] | 0.87 | - |
| Breast | PFOS | 1.16 [0.81, 1.67] | 0.41 | 1 [0.95, 1.06] | 0.88 | - |
| Urinary System | PFOA | 1.06 [0.98, 1.14] | 0.12 | 1.01 [0.92, 1.11] | 0.89 | - |
| Larynx | PFOA | 0.96 [0.8, 1.15] | 0.66 | 0.98 [0.72, 1.33] | 0.89 | - |
| Oral Cavity and Pharynx | PFHxS | 0.84 [0.68, 1.02] | 0.08 | 1.02 [0.76, 1.37] | 0.91 | - |
| Rectosigmoid Junction | PFHxS | 0.83 [0.53, 1.31] | 0.44 | 1.03 [0.62, 1.7] | 0.92 | - |
| Chronic Myeloid Leukemia | PFOA | 0.85 [0.65, 1.11] | 0.23 | 1.01 [0.75, 1.38] | 0.94 | - |
| Liver | PFOS | 1 [0.85, 1.17] | 0.98 | 1.01 [0.8, 1.27] | 0.94 | - |
| Anus Anal Canal and Anorectum | PFOA | 1.02 [0.75, 1.4] | 0.88 | 0.99 [0.79, 1.25] | 0.95 | - |
| Kidney and Renal Pelvis | PFOS | 0.92 [0.84, 1.01] | 0.09 | 1 [0.87, 1.14] | 0.95 | - |
| Brain and Other Nervous System | PFHxS | 1.07 [0.79, 1.45] | 0.66 | 0.99 [0.7, 1.39] | 0.95 | - |
| Lymphoma | PFOS | 0.99 [0.9, 1.09] | 0.79 | 1 [0.87, 1.14] | 0.97 | - |
| Other Oral Cavity and Pharynx | PFHxS | 1.56 [0.69, 3.52] | 0.28 | 0.97 [0.17, 5.45] | 0.97 | - |
| All Sites | PFOA | 1.01 [0.98, 1.05] | 0.48 | 1 [0.96, 1.04] | 0.97 | - |
| Appendix | PFOS | 0.82 [0.59, 1.15] | 0.25 | 1.01 [0.74, 1.36] | 0.97 | - |
| Transverse Colon | PFOS | 1 [0.79, 1.27] | 0.99 | 1 [0.78, 1.27] | 0.98 | - |
| Retroperitoneum | PFOA | 1.44 [0.93, 2.23] | 0.10 | 1.01 [0.55, 1.84] | 0.99 | - |
| Gallbladder | PFOS | 0.8 [0.52, 1.22] | 0.30 | 1 [0.72, 1.39] | 0.99 | - |
| Other Urinary Organs | PFOS | 1.02 [0.66, 1.59] | 0.91 | 1 [0.53, 1.87] | 0.99 | - |
| Liver and Intrahepatic Bile Duct | PFHxS | 0.98 [0.76, 1.28] | 0.90 | 1 [0.7, 1.43] | 0.99 | - |
| Small Intestine | PFOA | 1.07 [0.86, 1.34] | 0.54 | 1 [0.79, 1.26] | 1.00 | - |
| Penis | PFHxS | 0.54 [0.24, 1.21] | 0.13 | NA | NA | NA |
| Testis | PFOS | 0.87 [0.7, 1.07] | 0.18 | NA | NA | NA |
| Testis | PFOA | 0.87 [0.71, 1.07] | 0.19 | NA | NA | NA |
| Testis | PFHxS | 0.82 [0.55, 1.22] | 0.32 | NA | NA | NA |
| Other Male Genital Organs | PFOS | 0.69 [0.32, 1.47] | 0.33 | NA | NA | NA |
| Penis | PFOA | 1.12 [0.78, 1.61] | 0.54 | NA | NA | NA |
| Other Male Genital Organs | PFOA | 0.88 [0.44, 1.74] | 0.71 | NA | NA | NA |
| Pleura | PFOS | 0.72 [0.09, 5.96] | 0.76 | NA | NA | NA |
| Prostate | PFOA | 1.01 [0.95, 1.07] | 0.82 | NA | NA | NA |
| Pleura | PFOA | 0.82 [0.11, 5.89] | 0.84 | NA | NA | NA |
| Penis | PFOS | 1.03 [0.71, 1.51] | 0.87 | NA | NA | NA |
| Male Genital System | PFOS | 1 [0.94, 1.06] | 0.88 | NA | NA | NA |
| Other Male Genital Organs | PFHxS | 1.08 [0.34, 3.41] | 0.90 | NA | NA | NA |
| Prostate | PFOS | 1 [0.94, 1.07] | 0.93 | NA | NA | NA |
| Male Genital System | PFOA | 1 [0.94, 1.06] | 0.98 | NA | NA | NA |
| Pleura | PFHxS | NA | NA | NA | NA | NA |
| 1. All models were adjusted for county-level SES variables, urbanicity, smoking rate, obesity, and air pollution. | | | | | | |
| 2. cells were highlighted if crude p values were less than 0.05. | | | | | | |
| 3. NAs were produced with limited sample size or when we assessed cancers in male reproductive system among females or vice versa. | | | | | | |
| 4. ++ indicates cancers were significant in both sexes, + indicates cancers were siginificant only in one sex, - indicates cancers were not significant in either sex, NA indicates analysis was done only in one sex or none. | | | | | | |
